# Lewis Base-Enhanced C–H Bond Functionalization Mediated by a Diiron Imido Complex

**DOI:** 10.1021/acs.inorgchem.4c03922

**Published:** 2025-01-24

**Authors:** Reilly K. Gwinn, Trevor P. Latendresse, Owen N. Beck, Carla Slebodnick, Nicholas J. Mayhall, Claire E. Casaday, Diana A. Thornton

**Affiliations:** † Department of Chemistry, Virginia Tech, Blacksburg, Virginia 24061, United States; ‡ Department of Chemistry and Chemical Biology, Harvard University, Cambridge, Massachusetts 02138, United States

## Abstract

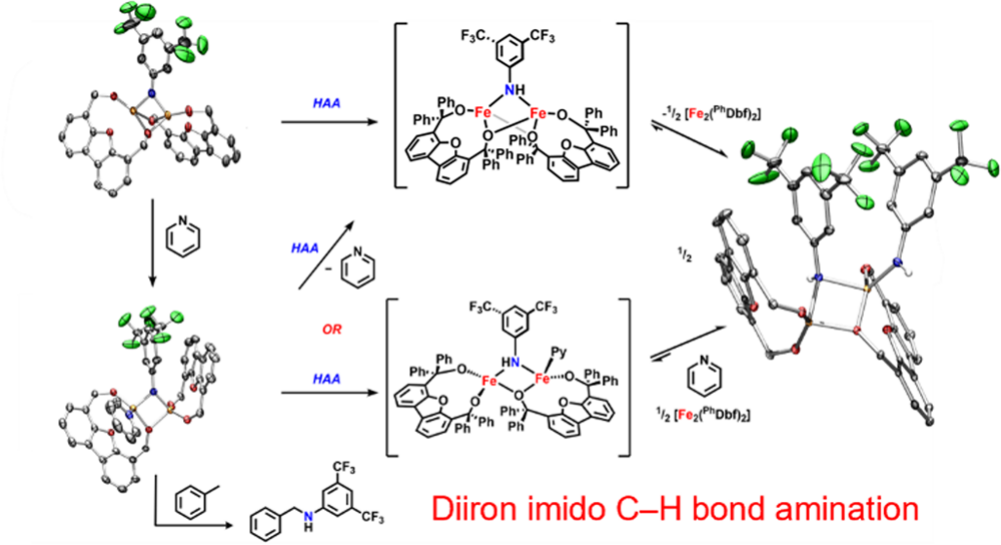

Herein, we investigate the effects of ligand design on the nuclearity and reactivity of metal–ligand multiply bonded (MLMB) complexes to access an exclusively bimetallic reaction pathway for C–H bond functionalization. To this end, the diiron alkoxide [Fe_2_(^Ph^Dbf)_2_] (**1**) was treated with 3,5-bis(trifluoromethyl)phenyl azide to access the diiron imido complex [Fe_2_(^Ph^Dbf)_2_(μ-NC_8_H_3_F_6_)] (**2a**) that promotes hydrogen atom abstraction (HAA) from a variety of C–H and O–H bond containing substrates. A diiron bis(amide) complex [Fe_2_(^Ph^Dbf)_2_(μ-NHC_8_H_3_F_6_)(NHC_8_H_3_F_6_)] (**3**) was generated, prompting the isolation of the analogous bridging amide terminal alkoxide [Fe_2_(^Ph^Dbf)_2_(μ-NHC_8_H_3_F_6_)(OC_19_H_15_)] (**4**) and the asymmetric pyridine-bound diiron imido [Fe_2_(^Ph^Dbf)_2_(μ-NC_8_H_3_F_6_)(NC_5_H_5_)] (**6a**). We found that **6a** is competent for toluene amination, indicating the effect of Lewis base-enhanced C–H bond functionalization. Mechanistic investigations suggest that the bimetallic bridging imido complex is the reactive intermediate as no monometallic species is detected during the time course of the reaction.

## Abstract 2

The effect of ligand design on the nuclearity and reactivity of metal–ligand multiply bonded complexes to access an exclusively bimetallic reaction pathway for C–H bond functionalization was investigated. Lewis base-enhanced C–H bond functionalization meditated by a diiron imido complex was observed as toluene amination only occurred in the presence of pyridine. As no monometallic species were detected, we propose that alkoxide ligands can facilitate C–H bond functionalization via bimetallic reaction pathways.

## 1 Introduction

C–H bond functionalization is an important transformation that enables the conversion of unreactive alkanes into synthetically useful materials. This reactivity offers streamlining potential in complex syntheses by eliminating complicated prefunctionalization and purification processes.^1^ However, the stability and strength of aliphatic C–H bonds (BDE ∼ 104 kcal mol^–1^)^2^ render this chemical transformation difficult. For this reason, transition metal catalysts capable of selectively promoting C–H bond functionalization have been widely explored. While many 4d and 5d transition metal complexes promote C–H bond functionalization,^3−5^ increasing demand for economically and environmentally benign systems has led researchers to explore systems utilizing 3d transition metal centers due to their high natural abundance and relatively diminished toxicity.^6^ In response, numerous methodologies for activating C–H bonds to construct new C–X bonds (e.g., X = O, N, C) using earth-abundant, late 3d transition metals have emerged.^7−11^ Owing to their preference for one-electron chemistry, many late first-row transition metal catalysts, in the presence of an external oxidant (e.g., O_2_, N_3_), operate in oxidative processes commonly involving the formation of a metal–ligand multiply bonded (MLMB) intermediate (e.g., metal imido, oxo, carbene),^6^ much like that of the enzyme Cytochrome P450.^12^ Unfortunately, the high reactivity of late transition 3d MLMB complexes can also pose limitations, including (1) fast decomposition that inhibits their direct isolation and (2) degradation during catalysis, which decreases turnover rates. To address these shortcomings, bimetallic complexes offer the possibility of redox-load sharing across two metals to enhance their stability and prevent premature decomposition. However, designing bimetallic MLMB systems to promote C–H bond functionalization has proved challenging, as the enhanced stability of these complexes often significantly dampened reactivity.

While several diiron imido complexes have been characterized,^13−22^ only one dimeric dipyrrin bridging imido complex was observed to promote C–H bond functionalization.^18^ Mechanistically, the diiron imido complex promotes C–H functionalization at the dinuclear site; however, the analogous monometallic iminyl species exists in equilibrium, promoting C–H functionalization through a competing mononuclear pathway.^18^ The existence of both species in solution presents a significant barrier to understanding properties that would aid in the future design of bimetallic MLMB systems. Therefore, in this report, we investigate the effect of ligand design on the nuclearity of iron imido systems to promote reactivity through bimetallic pathways.

Overall, we hypothesize that alkoxide ligands will facilitate C–H bond functionalization through a dinuclear reaction pathway as their enhanced π-donicity facilitates the formation of multimetallic complexes and their weak-field character engenders high-spin states that are necessary for the desired reactivity.^23,24^ Additionally, auxiliary ligand effects on the structure and reactivity of alkoxide-supported iron imido complexes are explored with Lewis bases. We hypothesize that Lewis base coordination will provide a means of assessing the alkoxide ligand’s ability to maintain dinuclearity in unfavorable environments, as well as exploring reactivity control. Herein, we report the isolation, characterization, and reactivity of alkoxide-supported diiron imido species, [Fe_2_(^Ph^Dbf)_2_(μ-NC_8_H_3_F_6_)] (**2a**) as well as the effect of the addition of Lewis base on the structure and reactivity of this species.

### 1.1 Isolation and Characterization of a Diiron Imido Complex: Fe_2_(^Ph^Dbf)_2_(μ-NC_8_H_3_F_6_) (**2a**)

**Scheme 1:**
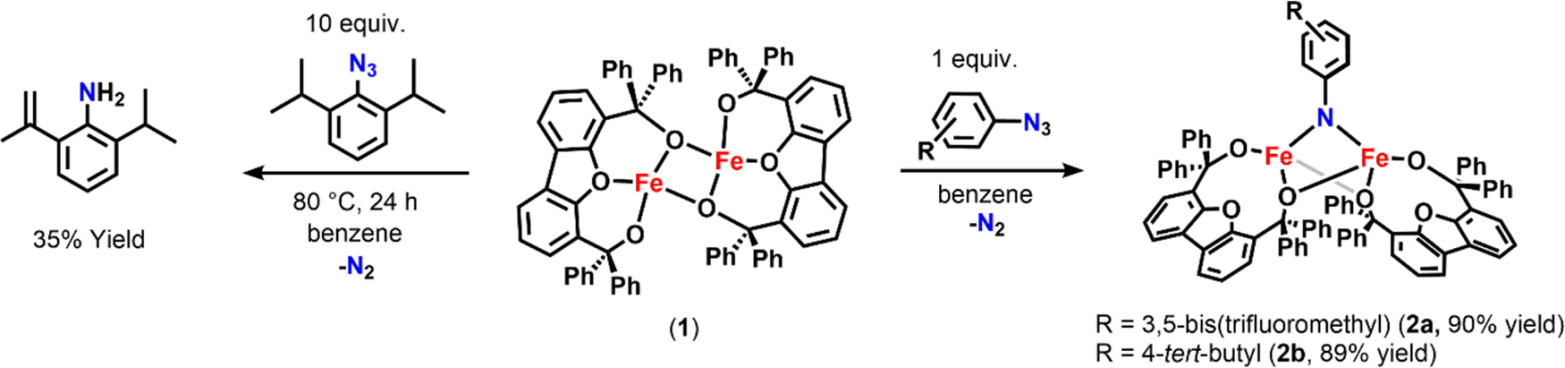
Synthesis of Diiron Imido Complexes **2a** and **2b** (Right) and the Intramolecular Dehydrogenation Reaction of **1** with 2,6-Diisopropylphenyl Azide (Left)

Initially, the previously reported diiron alkoxide^25^ [Fe_2_(^Ph^Dbf_2_)] (**1**) was exposed to a stoichiometric amount of 3,5-bis(trifluoromethyl)phenyl azide. Rapid consumption of the starting materials in ^1^H and ^19^F NMR spectra and the disappearance of the N–N bond stretch at 2108 cm^–1^ in the infrared (IR) spectrum indicated that azide activation had occurred (Figures S-9, S-10, and S-34). Upon azide activation, a single paramagnetic species was identified in the ^19^F NMR spectrum (**2a**, Scheme 1 and Figure S-10). Similar spectral changes in the ^1^H NMR and IR spectra were noted for several aromatic azides, including 4-*tert*-butylphenyl azide (**2b**), 4-nitrophenyl azide, and 2,4,6-trimethylphenyl azide, although reactions with *ortho*-substituted aryl azides required 24 h (Figures S-11, S-36, S-43, and S-44). Interestingly, the addition of 2,6-diisopropylphenyl azide to **1** did not afford a new species at room temperature, likely due to the increased steric bulk in the *ortho* position (Figure S-50). However, upon heating at 80 °C overnight, the resulting ^1^H NMR spectrum indicated the presence of dehydrogenated 2-isopropyl-6-(prop-1-en-2-yl)aniline (Scheme 1, Figures S-51 and S-53). Using 10 equiv of 2,6-diisopropylphenyl azide, 2-isopropyl-6-(prop-1-en-2-yl)aniline was observed in 35% yield (Scheme 1). Notably, under catalytic conditions, the ^1^H NMR spectrum revealed the formation of new paramagnetic species (Figure S-52). Although this species could not be isolated, intramolecular dehydrogenation of this substrate has been previously reported for other isolated iron imido complexes.^18,26,27^ Therefore, we propose that an iron imido species is indeed formed upon heating of **1** in the presence of 2,6-diisopropylphenyl azide, which then reacts rapidly at 80 °C to afford the dehydrogenated product. Encouraged by these results, we sought to identify the species **2a** formed upon azide activation with 3,5-bis(trifluoromethyl)phenyl azide.

**Figure 1:**
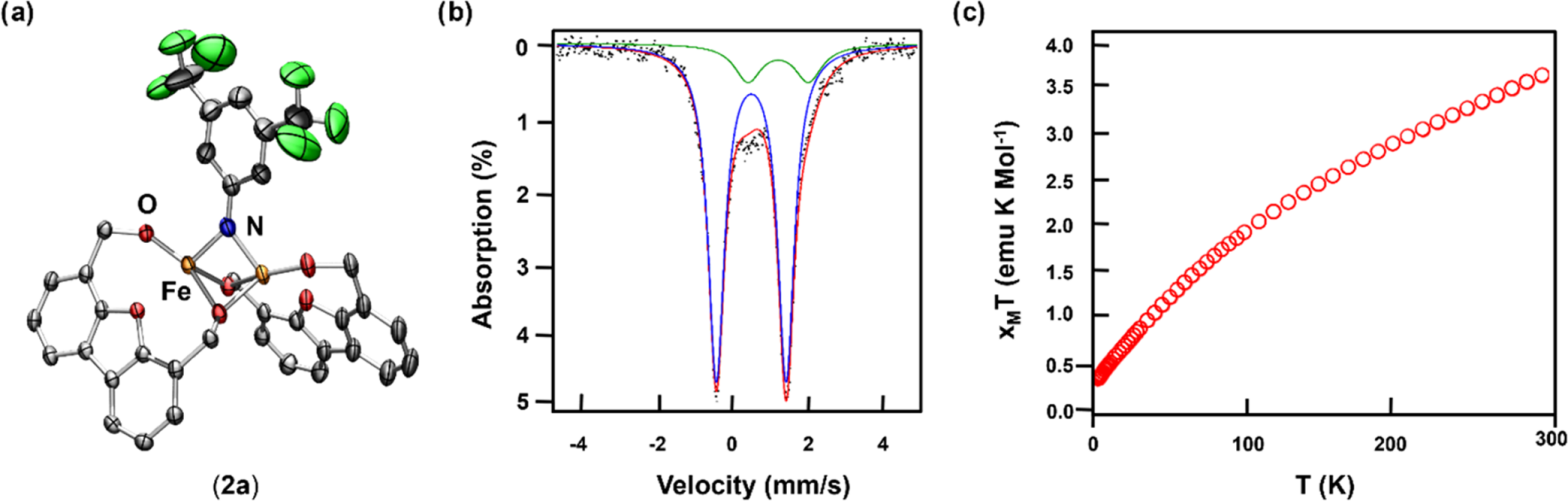
Truncated solid-state molecular structure of (a) [Fe_2_(^Ph^Dbf)_2_(μ-NC_8_H_3_F_6_)] (**2a**), with anisotropic displacement ellipsoids at 50% probability level; Color scheme: Fe, orange; O, red; N, blue; C, gray; F, green. Hydrogen atoms and phenyl groups on the ligand are excluded for clarity. (b) Zero-field ^57^Fe Mössbauer spectra of Fe_2_(^Ph^Dbf)2(μ-NC_8_H_3_F_6_) (**2a**; δ = 0.49 mm s^–1^; |Δ*E*_Q_| = 1.85 mm s^–1^) collected at 90 K. (c) Variable-temperature susceptibility data for Fe_2_(^Ph^Dbf)_2_(μ-NC_8_H_3_F_6_) (**2a**): χ_M_*T* vs *T* collected at 1.0 T.

Gratifyingly, product **2a** was isolated in 90% yield as a dark blue powder and identified as the bridging diiron imido [Fe_2_(^Ph^Dbf)_2_(μ-NC_6_H_3_F_3_)] (**2a**) via X-ray crystallography (Figure 1a). The iron centers adopt a *pseudo* tetrahedral geometry with only slight deviations between coordination environments. Likely these small perturbations are a result of crystal packing or the large steric profile of the ^Ph^Dbf ligand that results in a slight tilting of one ligand. As expected, the Fe–N_imido_ bond lengths of 1.905(2) and 1.887(2) Å are in good agreement with other reported bridging diiron imido species (Tables S-5 and S-7).^13−20^ Interestingly, both bridging alkoxide units remained intact upon azide addition, facilitating a short Fe–Fe distance of 2.5180(4) Å and an acute Fe–N_imido_–Fe angle of 83.20(9)°, which are unusual among other diiron bridging imides. Indeed, the short Fe–Fe distance is indicative of a weak interaction between the metal centers such that **2a** is best described as Fe^III^/Fe^III^ edge-sharing tetrahedra (Figure S-87). Additionally, **2b** was isolated from the reaction of **1** with 4-*tert*-butylphenyl azide in 89% yield as a blue powder and identified as the analogous diiron bridging imido [Fe_2_(^Ph^Dbf)_2_(μ-NC_10_H_13_)] (**2b**) via X-ray crystallography (Scheme 1, Figure S-78, and Table S-5). Indeed, the bond metrics obtained for **2b** were in good agreement with those of **2a**. Thus, we propose that the unique structural properties, such as the short Fe–Fe distance and acute Fe–N_imido_–Fe angle, are a direct result of the increased π-donicity of the supporting alkoxide ligand (^Ph^Dbf) that sustains the bimetallic structure through its bridging units.

**Figure 2:**
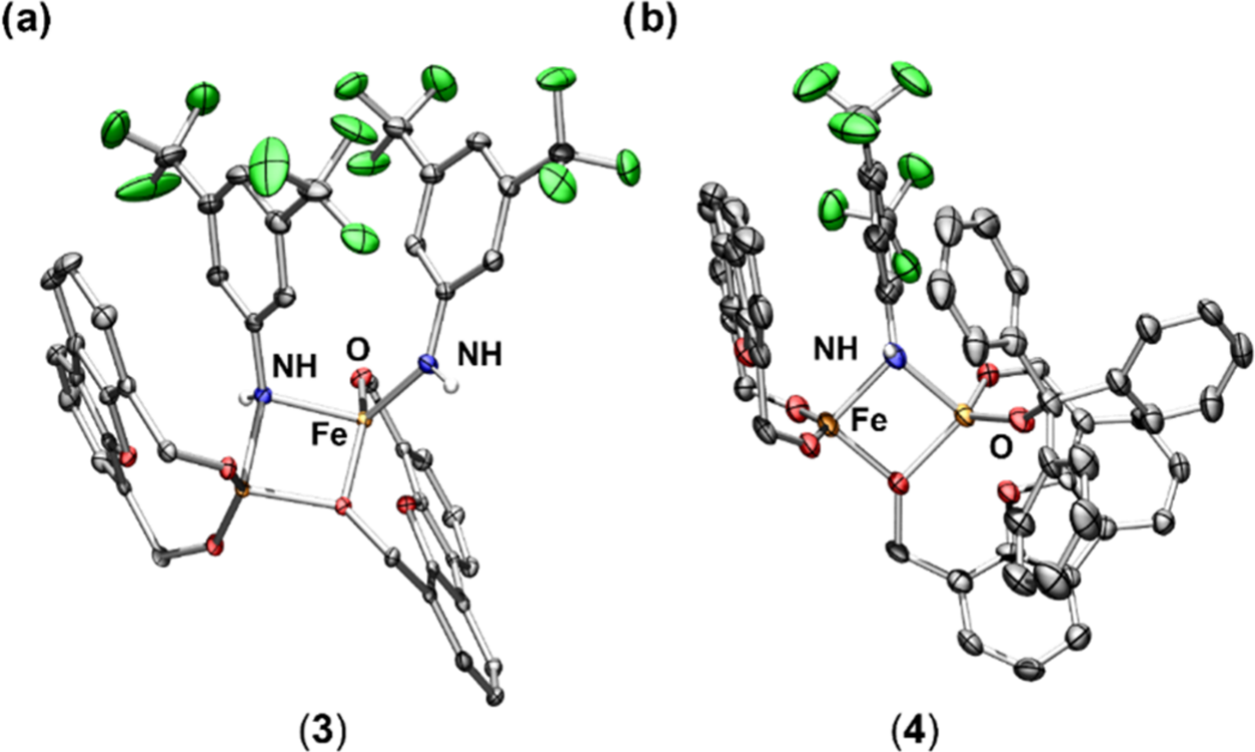
Truncated solid-state molecular structure of (a) Fe_2_(^Ph^Dbf)_2_(μ-NHC_8_H_3_F_6_)(NHC_8_H_3_F_6_) (**3**) and (b) Fe_2_(^Ph^Dbf)_2_(μ-NHC_8_H_3_F_6_)(OC_19_H_15_) (**4**), with anisotropic displacement ellipsoids at 50% probability level. Color scheme: Fe, orange; O, red; N, blue; C, gray; F, green. Hydrogen atoms (except NH) and phenyl groups on the ligand are excluded for clarity.

**Figure 3:**
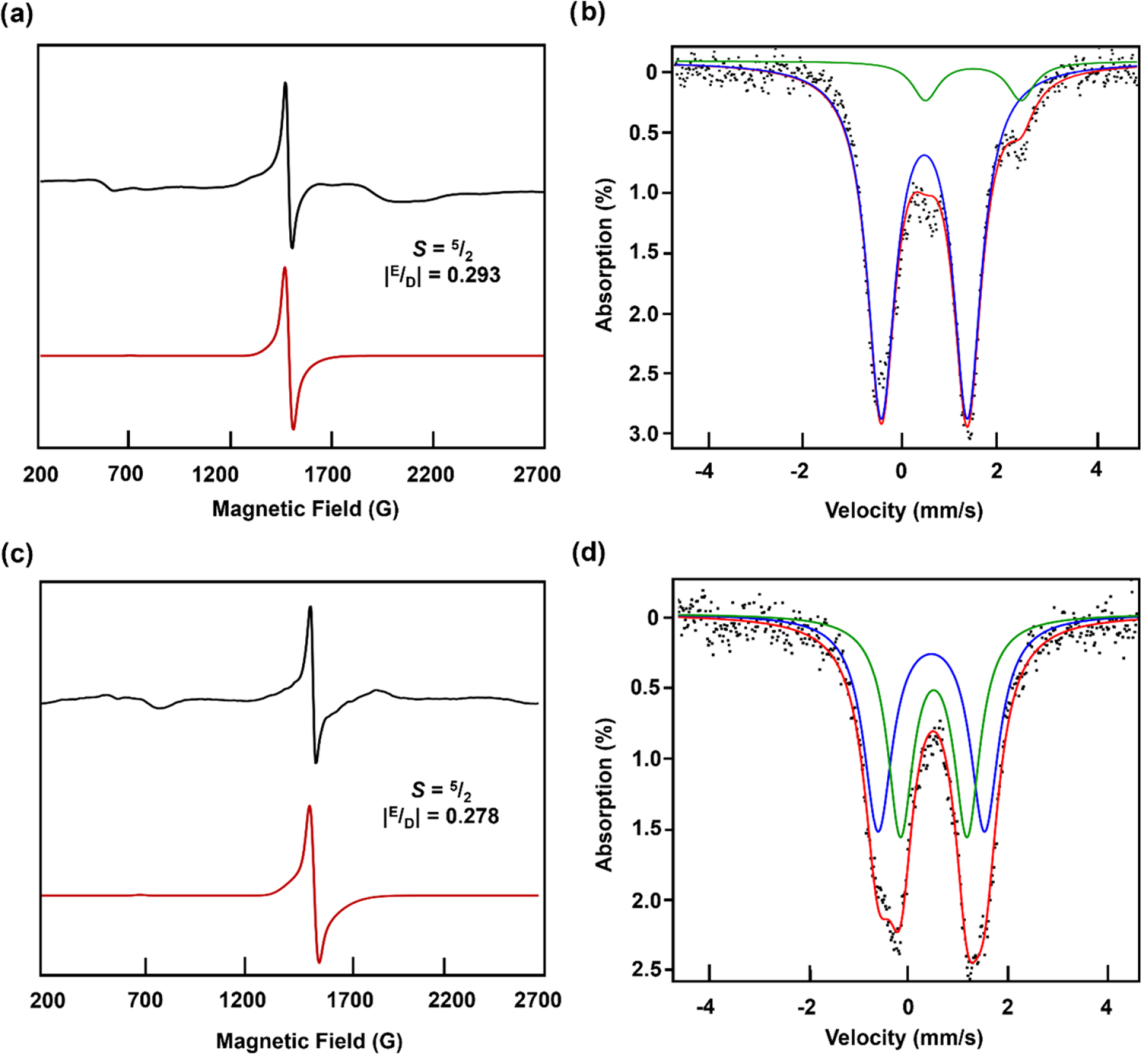
Frozen toluene EPR spectra for (a) Fe_2_(^Ph^Dbf)_2_(μ-NHC_8_H_3_F_6_)(NHC_8_H_3_F_6_) (**3**; *g*_eff_ = 4.25) and (c) Fe_2_(^Ph^Dbf)_2_(μ-NHC_8_H_3_F_6_)(OCPh_3_) (**4**; *g*_eff_ = 4.26) collected at 80 K. The black line is the experimental spectra, the red line is the simulation, and the simulation parameters obtained from VisualRhombo^39^ are in bolded font on the spectra. Zero-field ^57^Fe Mössbauer spectra of (b) Fe_2_(^Ph^Dbf)_2_(μ-NHC_8_H_3_F_6_)(NHC_8_H_3_F_6_) (**3**; δ = 0.49 mm s^–1^; |Δ*E*_Q_| = 1.78 mm s^–1^) and (d) Fe_2_(^Ph^Dbf)_2_(μ-NHC_8_H_3_F_6_)(OCPh_3_) (**4**; 50% δ = 0.47 mm s^–1^; |Δ*E*_Q_| = 2.15 mm s^–1^ and 50% δ = 0.51 mm s^–1^; |Δ*E*_Q_| = 1.34 mm s^–1^), collected at 90 K.

**Figure 4:**
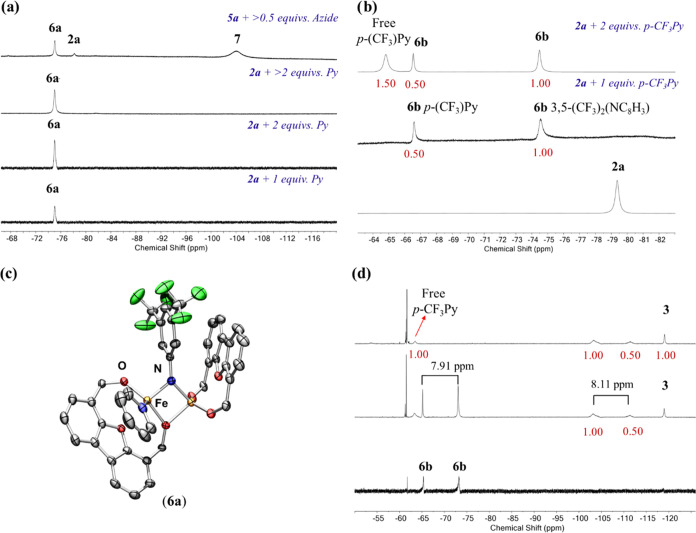
^19^F NMR spectra of the reactions of (a) **2a** with 1 equiv of pyridine (bottom), 2 equiv of pyridine (middle), >2 equiv of pyridine (middle), and **5a** with 0.5 equiv of 3,5-bis(trifluoromethyl)phenyl azide (top); (b) **2a** + 1 equiv (middle) and 2 equiv of 4-trifluoromethylpyridine (top); (c) the truncated solid-state molecular structure of Fe_2_(^Ph^Dbf)_2_(μ-NC_8_H_3_F_6_)(NC_5_H_5_) (**6a**), with anisotropic displacement ellipsoids at 50% probability level. Color scheme: Fe, orange; O, red; N, blue; C, gray; F, green. Hydrogen atoms and phenyl groups on the ligand are excluded for clarity. (d) ^19^F NMR spectra of **6a** with 1,4-cyclohexadiene at 90 °C for 24 h (middle) and 36 h (top). All integrations are rounded to the nearest 0.50, and relative stoichiometries are listed in purple.

Successful isolation of **2a** prompted the study of the electronic properties of this diiron imido species to assess the potential of **2a** to promote C–H bond functionalization. Encouragingly, ^57^Fe Mössbauer analysis of **2a** indicated a single Fe^III^ environment (δ = 0.49 mm s^–1^; |Δ*E*_Q_| = 1.85 mm s^–1^, Figure 2a), alongside an Fe^II^ impurity (10%), with parameters in line with previously reported high-spin Fe^III^-imido species and calculated values (Figure 1b and Table S-1).^18,24,28,29^ The high-spin nature of **2a** was further investigated by measuring the temperature dependence of the molar magnetic susceptibility (χ_M_) via superconducting quantum interference device (SQUID) magnetometry under a 1.0 T external magnetic field between *T* = 300–2 K (Figure 1c). The room temperature value of the molar magnetic susceptibility temperature product (χ_M_*T*) of **2a** was found to be 3.06 emu·K mol^–1^ (at 300 K). This value is significantly lower than the expected value of 8.754 emu·K mol^–1^ for two noninteracting high-spin *S* = 5/2 Fe^III^ sites and indicates the presence of antiferromagnetic coupling between the Fe^III^ centers. Upon cooling, the χ_M_*T* value gradually decreases to a minimum value of 0.223 emu·K mol^–1^ at 2 K (Figures 2b, S-6, and S-7). The nonzero χ_M_*T* value at 2 K can be attributed to partial population of higher-spin excited states of **2a** or the presence of a paramagnetic impurity—possibly the high-spin Fe^II^ impurity observed via ^57^Fe Mössbauer spectrum mentioned above. Unfortunately, attempts to model the static magnetic properties of **2a** have been unsuccessful, resulting in unreasonable fitting parameters. We note that the χ_M_*T* data is reproducible and χ_M_*T* curves at variable fields show only slight deviations at high temperatures (Figure S-8). Nevertheless, theoretical investigations suggest weak antiferromagnetic coupling between *S* = 5/2 Fe centers of **2a** (Figure 2c), predicting a calculated exchange coupling constant of −54.2 cm^–1^ (B3LYP/LANL2DZ, Table S-9) when the Yamaguchi approach for high-spin and broken symmetry solutions (*H* = −2*JS*_1_·*S*_2_) was employed.^30^ The presence of an antiferromagnetically coupled, *S* = 0 ground state, of **2a** was further supported by low-temperature electron paramagnetic resonance (EPR) spectroscopy in which a discernible EPR signal was not observed below 80 K (Figure S-24). Remarkably, the high-spin nature of **2a**, as well as the weak antiferromagnetic coupling between Fe centers was reminiscent of the reactive dipyrrin diiron imido complex.^18,21,22^ In fact, these properties were proposed to be responsible for the observed HAA reactivity at the dinuclear site.^18^ Thus, we hypothesized that **2a** can promote nitrene group transfer reactivity as a bimetallic species.

### 1.2 Reactivity of the Imido Complex

#### 1.2.1 Styrene Aziridination and O–H Bond Activation Reactivity

**Scheme 2:**
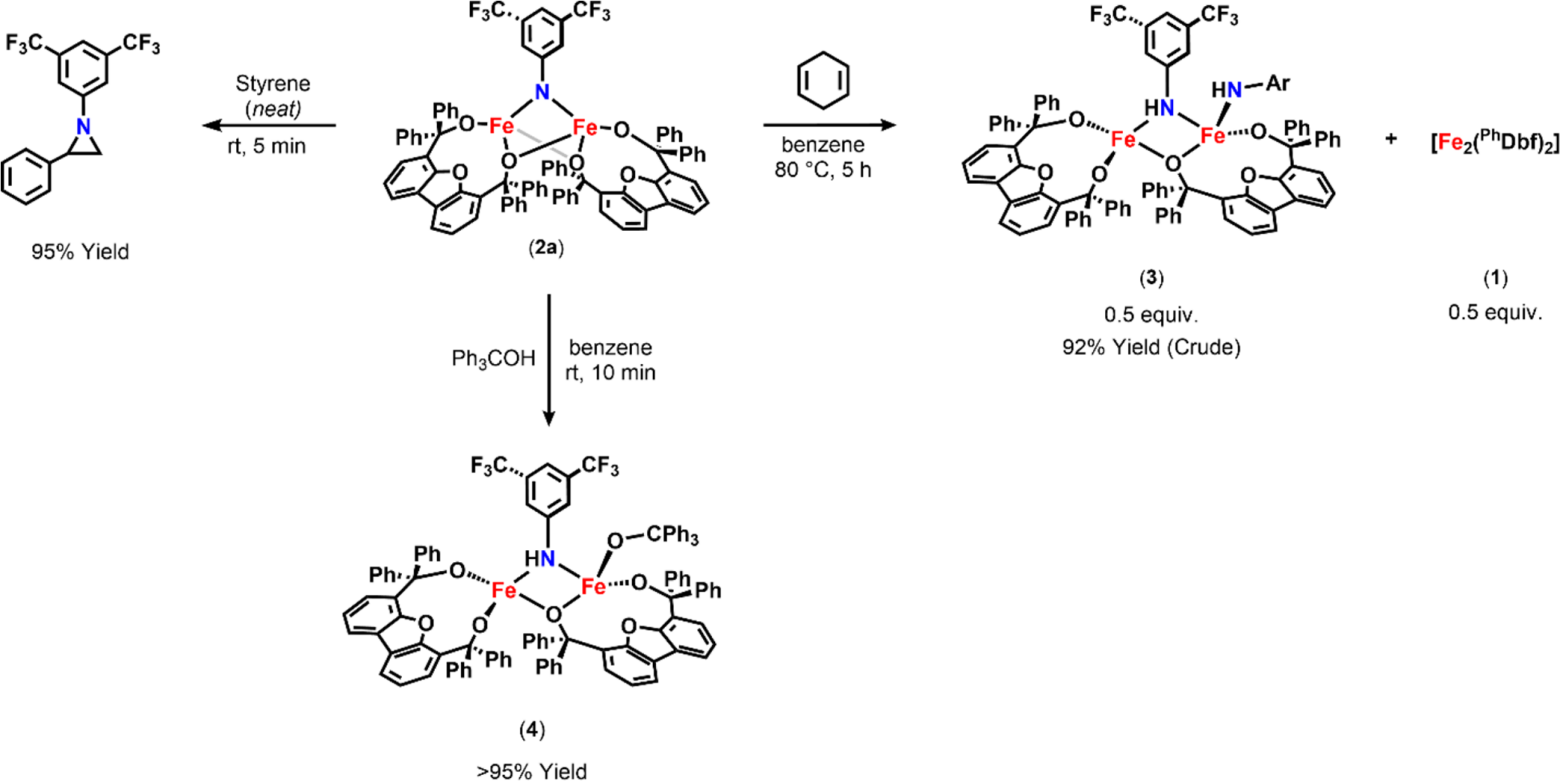
Reactivity of **2a**

Initially, **2a**′s ability to promote nitrene group transfer to unsaturated carbon–carbon bonds was examined. As such, **2a** was stirred in neat styrene at room temperature. The corresponding 1-(3,5-bis(trifluoromethyl)phenyl)-2-phenylaziridine was detected as the major organic product after 1 h, suggesting that **2a** was capable of group transfer reactivity (Scheme 2). Akin to the reaction of **1** with 2,6-diisopropylphenyl azide, styrene aziridination was performed catalytically using 10 mol % of **1** to afford the corresponding aziridine in quantitative yield (>95% yield). This observed reactivity encouraged further investigations of whether **2a** could promote more challenging transformations.

To probe whether **2a** could promote HAA, its reactivity with the weak O–H-bond-containing substrates was investigated. Thus, **2a** was exposed to stoichiometric 2-hydroxy-2-azaadamaantane (AdNOH; BDE = 76 kcal mol^–1^; Scheme 2).^2^ Indeed, the generation of the corresponding AdNO^•^ radical in the X-band EPR spectrum indicated that HAA had occurred (Figures S-30 and S-31). Unexpectedly, multiple paramagnetic species were noted by ^19^F NMR spectroscopy (δ −71.70 and −119.97 ppm; Figure S-55). However, upon addition of a stoichiometric amount of commercially purchased AdNO^•^ radical to **2a**, the species corresponding to the signal at δ −71.70 ppm (^19^F NMR) was identified as a byproduct resulting from subsequent binding of the AdNO^•^ radical to unreacted **2a** in solution (Figure S-56). Therefore, a reaction with **2a** and the O–H bond containing substrate 2,4,6-tri-*tert*-butylphenol butylphenol (BDE = 83 kcal mol^–1^; Scheme 2)^2^ was explored, as the bulky phenol radical is less likely to react with **2a** due to steric restraints. Indeed, heating this reaction at 80 °C overnight resulted in the formation of **3** in the ^19^F NMR spectrum (δ −119.97 ppm; Figure S-57), along with the respective phenol radical identified by EPR spectroscopy (Figure S-32). Together, these results indicated that **2a** is competent for HAA from O–H-bond-containing substrates to afford a new paramagnetic species (**3**), as identified in the ^19^F NMR spectra.

#### 1.2.2 C–H Bond Activation Reactivity

Encouraged by the observed HAA from O–H-bond-containing substrates, C–H bond activation was explored. To this end, the addition of excess 1,4-cyclohexadiene (BDE ∼ 76 kcal mol^–1^)^2^ to **2a** afforded **3** upon heating at 80 °C for 5 h (Scheme 2). More interestingly, when **2a** was dissolved in toluene (BDE ∼ 89 kcal/mol),^2^**3** was generated at room temperature, although no further reactivity was observed. When the reaction was quenched, 3,5-bis(trifluoromethyl)aniline and bibenzyl were identified, confirming that HAA from toluene had occurred. While encouraging, these results were peculiar considering radical recombination to form the aminated product typically occurs rapidly following HAA. More interestingly, only trace *N*-benzyl-3,5-bis(trifluoromethyl)aniline was observed upon quenching the reaction of **2a** in toluene after extensive heating (Figures S-61, S-63, and S-88), indicating radical recombination can be promoted, although it is largely inhibited under the current reaction conditions. Thus, to better understand this behavior, we sought to identify compound **3**.

Notably, the stability of **3** was unusual as the expected product of HAA from **2a** was the corresponding diiron bridging amide (Fe^II^/Fe^III^) complex. Fe^II^/Fe^III^ bridging diiron-amide species are often transient intermediates that rapidly undergo radical recombination or decomposition that in turn prevents their isolation.^18^ With this in mind, we hypothesized that **3** was a species other than the expected bridging amide complex, such as a stable Fe^II^/Fe^II^ diiron-aniline, as similar species have been reported in the literature.^18,31−34^ Nevertheless, **3** was isolated in 92% yield as a dark blue powder via the reaction of **2a** with excess 1,4-cyclohexadiene and identified as an asymmetric Fe^III^/Fe^III^ bis(amide) [Fe_2_(^Ph^Dbf)_2_(μ-NHC_8_H_3_F_6_)(NHC_8_H_3_F_6_)] (**3**) via X-ray crystallography (Figure 2, Scheme 2, and Table S-3). Unexpectedly, **3** featured two amide ligands—one terminal and one bridging—despite only one bridging imido ligand being bound to **2a**. Comparatively, the dipyrrin diiron system reported by Betley noted that no Fe^II^/Fe^III^ bridging amide could be isolated in solution or via retrosynthesis; however, the corresponding stable diferrous bis(amide) could be.^18^ Thus, our system may be exhibiting a similar behavior, in which upon HAA by **2a**, a reactive Fe^II^/Fe^III^ bridging amide undergoes a rapid rearrangement, generating equimolar amounts of **3** and **1**. Interestingly, one of the alkoxide units remained bridging, and the geometry of the Fe centers is best described as *pseudo* tetrahedral and distorted seesaw. As a result, the Fe–Fe distance was significantly elongated (3.061(3) Å), such that there is no longer a weak Fe–Fe interaction. These observed differences in structure prompted further investigation concerning the electronic properties of compound **3**.

As expected, the Fe^III^/Fe^III^ assignment inferred from the crystallographic data was further corroborated by EPR (80 K), as the spectrum of **3** revealed an isotropic signal indicative of a high-spin Fe^III^ complex with high rhombicity (*g*_eff_ = 4.27; |*E*/*D*| = 0.293, Figure 3a) consistent with the calculated coupling constant of −3.8 cm^–1^ (B3LYP/LANL2DZ). However, the ^57^Fe Mössbauer spectrum of **3** showed a single high-spin Fe^III^ species (δ = 0.49 mm s^–1^; |Δ*E*_Q_| = 1.78 mm s^–1^, Figure 3b), despite the asymmetry between the two iron centers. Likely, the observed broadened quadrupole doublet results from the overlap of two Fe^III^ signals with isomer shifts indistinguishable by the modeling software, as the experimental parameters are in line with previously reported high-spin Fe^III^ species and calculations (δ = 0.44 mm s^–1^, |Δ*E*_Q_| = 1.556 mm s^–1^ and δ = 0.43 mm s^–1^, |Δ*E*_Q_| = −1.811 mm s^–1^).^18,24,28,29^ Regardless, this unusual spectroscopic signature warranted further investigation of related asymmetric Fe^III^/Fe^III^ bridging amide species.

Thus, triphenylmethanol (BDE ∼ 87 kcal mol^–1^)^2^ was used to access an isoelectronic diiron bridging amide with a terminal alkoxide ligand. Upon addition of triphenylmethanol to **2a**, instantaneous conversion into a new species (**4**) was observed by ^19^F NMR spectroscopy (Scheme 2 and Figure S-14). Product **4** was isolated in 95% yield as a brown powder and identified as the targeted asymmetric Fe^III^/Fe^III^ terminal alkoxide bridging amide [Fe_2_(^Ph^Dbf)_2_(μ-NHC_8_H_3_F_6_)(OC_19_H_15_)] (**4**) via X-ray crystallography (Figure 2). The bond metrics for **4** are in good agreement with those found for **3** (Table S-6) and the EPR spectrum reveals a similar isotropic signal (*g*_eff_ = 4.28, |*E*/*D*| = 0.278, Figure 3c) consistent with the calculated coupling constant of −8.1 cm^–1^ (B3LYP/LANL2DZ). Notably, ^57^Fe Mössbauer analysis of **4** revealed two quadrupole doublets indicative of high-spin Fe^III^ centers (δ = 0.47 mm s^–1^; |Δ*E*_Q_| = 2.15 mm s^–1^; δ = 0.51 mm s^–1^; |Δ*E*_Q_| = 1.34 mm s^–1^, Figure 3d), as would be expected for an asymmetric species. However, these signals are closely related, suggesting that it is possible that the coordinatively distinct amide groups in **3** are not sufficient to distinguish the two iron centers in the ^57^Fe Mössbauer spectrum, as proposed. Nevertheless, the isolation of both bridging amide species (**3** and **4**) suggests that the alkoxide ligands may be capable of supporting a C–H functionalization pathway via exclusively bimetallic species as proposed, under the correct conditions.

#### 1.2.3 Radical Recombination Capabilities of **3**

To better understand the lack of observed radical recombination, we investigated whether complex **3** could promote radical recombination in the presence of an organic radical. Previously, Betley reported treatment of an isolated monomeric iron-amide species with the triphenylmethyl radical (Ph_3_C^•^), to afford the corresponding ArNCPh_3_ compound, featuring a new C–N bond, indicating that the monomeric iron-amide species underwent radical recombination. Inspired by this work, the reaction of **3** with the triphenylmethyl radical (Ph_3_C^•^) was examined.^18,35^ As expected, no radical recombination was observed, suggesting that complex **3** is not competent for radical recombination and, thus, not responsible for the formation of the trace amounts of aminated product observed via gas chromatography–mass spectrometry (GC–MS) analysis. These results indicate that the observed radical recombination is promoted by another species in solution that is likely in equilibrium with and therefore is limited by **3**.

**Scheme 3:**
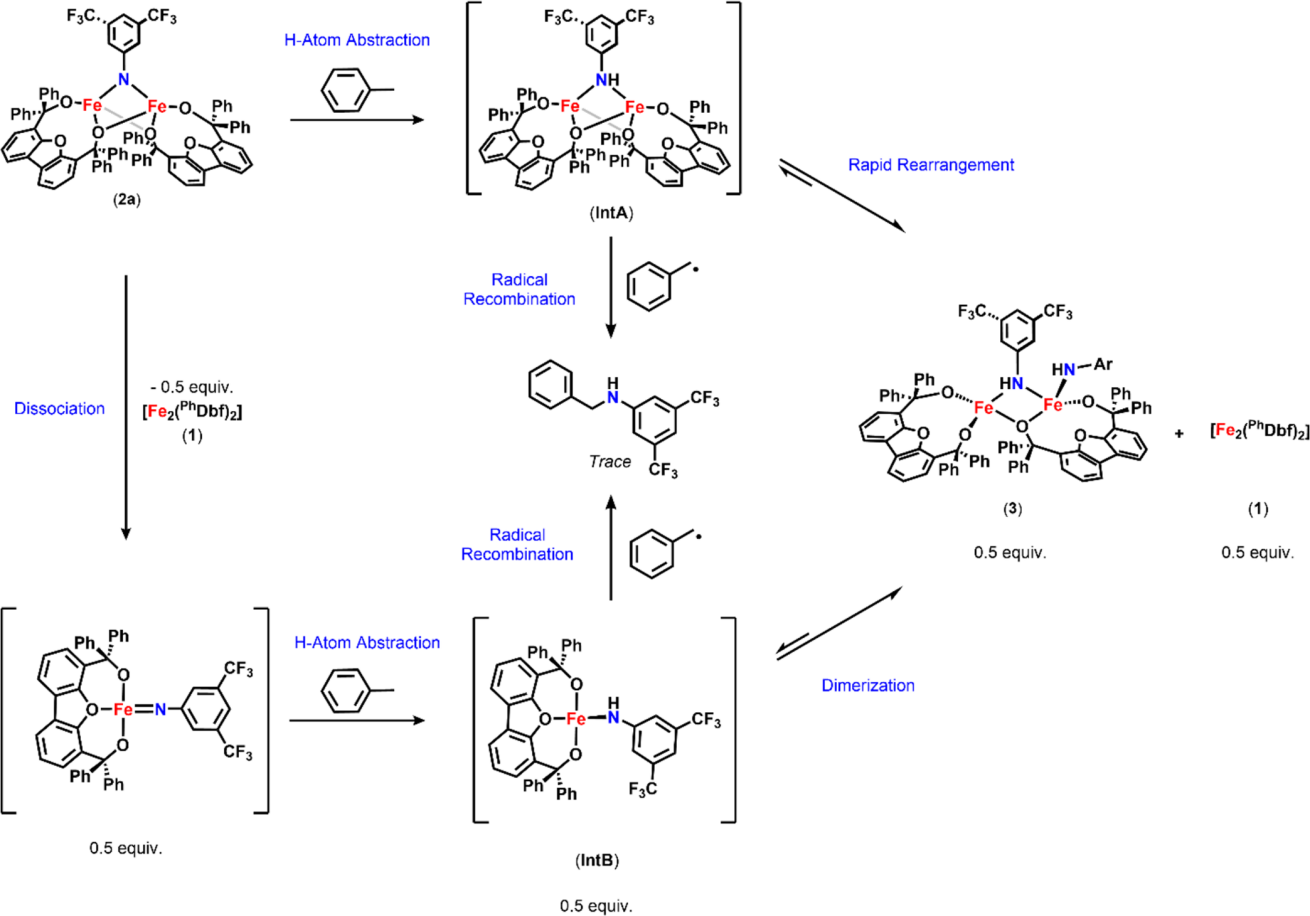
Possible Reaction Pathways for the Formation of **3** via HAA from Toluene Mediated by **2a** and Radical Recombination to Access Trace Amounts of *N*-Benzyl-3-5-bis(trifluoromethyl)aniline

Examining previously reported reactive bridging imides, we note that the reactive dipyrrin diiron imido complex published by Betley promoted C–H bond functionalization at both the mono- and dinuclear site, as both bimetallic and monomeric imido species exist in equilibrium.^18^ Additionally, studies of dinickel species reported by the Warren group revealed that the reactive monometallic metal imido species were responsible for C–H bond functionalization.^36^ In the presence of bulky substrates, the monometallic nickel imido species converted into the analogous, unreactive dinickel species, requiring dissociation back into the monomeric species for C–H bond functionalization to occur. These literature precedents along with our experimental results led us to rationalize the reactivity of **2a** and formation of **3** via either (1) dissociation of **2a** into a monomeric imido that undergoes HAA to afford a ferric amide (Scheme 3; Intermediate B; **IntB**) which dimerizes to afford **3**, similar to the previously reported examples, or (2) direct HAA by **2a** to generate a diiron bridging amide ([Fe_2_(^Ph^Dbf)_2_(N**H**C_8_H_3_F_6_)], Intermediate A; **IntA**; Scheme 3), which undergoes a rapid rearrangement to give **3**. In the latter pathway, the proposed **IntA** is highly unstable, as the reactive nature of Fe^II^/Fe^III^ bridging amide species has been reported to prevent their isolation. This instability in combination with the steric encumbrance introduced by the bridging bulky alkoxide and fluorinated aryl imido ligands likely results in the preferential rearrangement to **3** under the current conditions (Scheme 3). To gain insight into the reaction pathway, further mechanistic investigations were conducted.

### 1.3 C–H Bond Activation Reaction Pathway

#### 1.3.1 Lewis Base Effects on Structure and Electronics

To probe whether dissociation of **2a** into a monometallic iron imide is likely, we sought to test how the reactivity is altered in the presence of Lewis bases. We previously reported a series of high-spin iron alkoxide complexes that displayed nuclearity control via the addition of substituted pyridines.^25^ It was noted that in the presence of ethereal solvents (i.e., tetrahydrofuran and diethyl ether), **1** maintained dinuclearity, despite the highly electrophilic metal centers and unsaturated coordination sphere. However, upon addition of the more basic substituted pyridines, analogous monomeric species and asymmetric diiron complexes were isolated selectively, dependent on the electron richness of the pyridine ligand. With this in mind, we proposed that Lewis bases could be utilized to assess the ability of the alkoxide ligands to maintain nuclearity, as well as provide us a means of isolating monomeric imido and amide species for characterization.

**Scheme 4:**
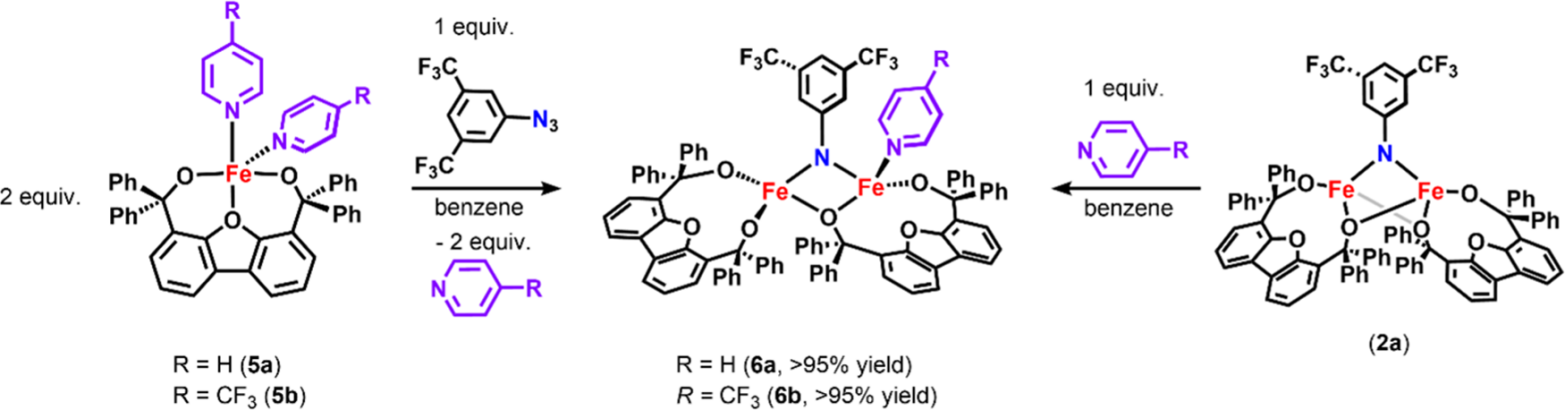
Synthesis of Pyridine-Bound Diiron Imido Complexes **6a** and **6b** Reported yields for **6a** and **6b** are for the reaction of **2a** with the appropriate Lewis base. Complex **7** was omitted for clarity, as it could not be identified and **6a** can be isolated via reaction of **2a** with pyridine. Thus, the reaction of **2a** with Lewis base was the preferred synthetic pathway to access **6a** and **6b**.

Initially, we examined the previously reported alkoxide iron pyridine complexes [Fe(^Ph^Dbf)(NC_5_H_5_)_2_] (**5a**) and [Fe(^Ph^Dbf)(NC_5_H_4_F_3_)_2_] (**5b**) under similar conditions (Scheme 4 and Figure 4a,b).^25^ Unexpectedly, the reaction with monomeric **5a** and 3,5-bis(trifluoromethyl)phenyl azide generated two species in the ^19^F NMR spectrum tentatively assigned as **6a** and an unknown compound **7** (Figure 4a). However, the reaction with **5b** resulted in a single paramagnetic species (**6b**) in quantitative yield (>95% yield, Scheme 4). Notably, the reaction with **5b** and 3,5-bis(trifluoromethylphenyl) azide required only a half equivalent of azide (relative to **5b**) to consume the starting material, suggesting that **6b** was a bimetallic species. Furthermore, the ^19^F NMR spectrum supports the addition of only one *p*-CF_3_Py ligand (**6b**), as indicated by the observed integration of 1:2 of the ^19^F signals corresponding to the bound *p*-CF_3_Py and the imido unit, respectively (Figures 4b, S-22 and S-23).

To probe the identity of **6a** and **7**, we attempted to access these species via the direct addition of pyridine to **2a**. Treatment of **2a** with a stoichiometric amount of pyridine generated **6a** in quantitative yield (>95% yield, Scheme 4 and Figure 4a) as a blue-green powder. Satisfyingly, no additional species were observed upon addition of excess pyridine (>2 equiv, Figures 4a and S-21), suggesting that the secondary species (**7**) formed in the reaction of aryl azide with **5a** was not a monomeric imido complex. Unfortunately, complex **7** could not be identified. Therefore, the addition of stoichiometric pyridine to **2a** was the preferred pathway to access **6a** cleanly. Thus, all further studies were conducted in the presence of 1 equiv of pyridine via direct addition to **2a**.

**6a** was identified as the asymmetric pyridine-bound bridging diiron imido [Fe_2_(^Ph^Dbf)_2_(μ-NC_8_H_3_F_6_)(NC_5_H_5_)] (**6a**) via X-ray crystallography (Figure 4c). Akin to the structures of **3** and **4**, upon coordination of an additional ligand, one of the bridging alkoxide units dissociated. Thus, the metal centers adopt pseudo tetrahedral and distorted seesaw geometries, respectively. As a result, the Fe–Fe distance [2.8117(6) Å] and Fe–N_imido_–Fe bond angle [97.10(12)°] increased significantly. The Fe–N_imido_ bond lengths (1.8615(17), 1.8927(17) Å) remained in good agreement with other diiron imido complexes.^13−20^ However, these Fe–N_imido_ bond lengths were shorter than those of **2a** (1.887(2), 1.905(2) Å) and **2b** (1.884(4), 1.898(4) Å), due to the decreased steric hindrance at the diiron core that facilitates a wider Fe–N_imido_–Fe angle and enhances the interaction between the metal d-orbitals and nitrogen p-orbitals to affect a greater multiple bond character. Computational analysis supports the proposed stronger interactions as the coupling between Fe atoms increased upon pyridine addition to −79.2 cm^–1^ (B3LYP/LANL2DZ, Table S-9). Further, the silent EPR spectrum (80 K) further supports the proposed antiferromagnetic coupling of the iron centers (Figure S-29). Interestingly, ^57^Fe Mössbauer analysis of **6a** indicated a single high-spin Fe^III^ environment (δ = 0.49 mm s^–1^; |Δ*E*_Q_| = 1.78 mm s^–1^, Figure S-4),^18,24,28,29^ with parameters almost identical to those of **2a** and **3**, as well as a broadened signal reminiscent of those observed for **3** (Figures S-22 and S-23).

Altogether, the isolation of **6a** and **6b** in the presence of Lewis bases—even in the presence of higher than 2 equiv—suggests that the formation of a monometallic iron imido complex is unfavorable and that HAA by **2a** is likely occurring at the dinuclear site (Scheme 3). To probe this hypothesis, we examined **6a**’s ability to promote HAA at the dinuclear site and the effects of Lewis base coordination on the reactivity.

#### 1.3.2 Lewis Base Effects on Reactivity

**Scheme 5:**
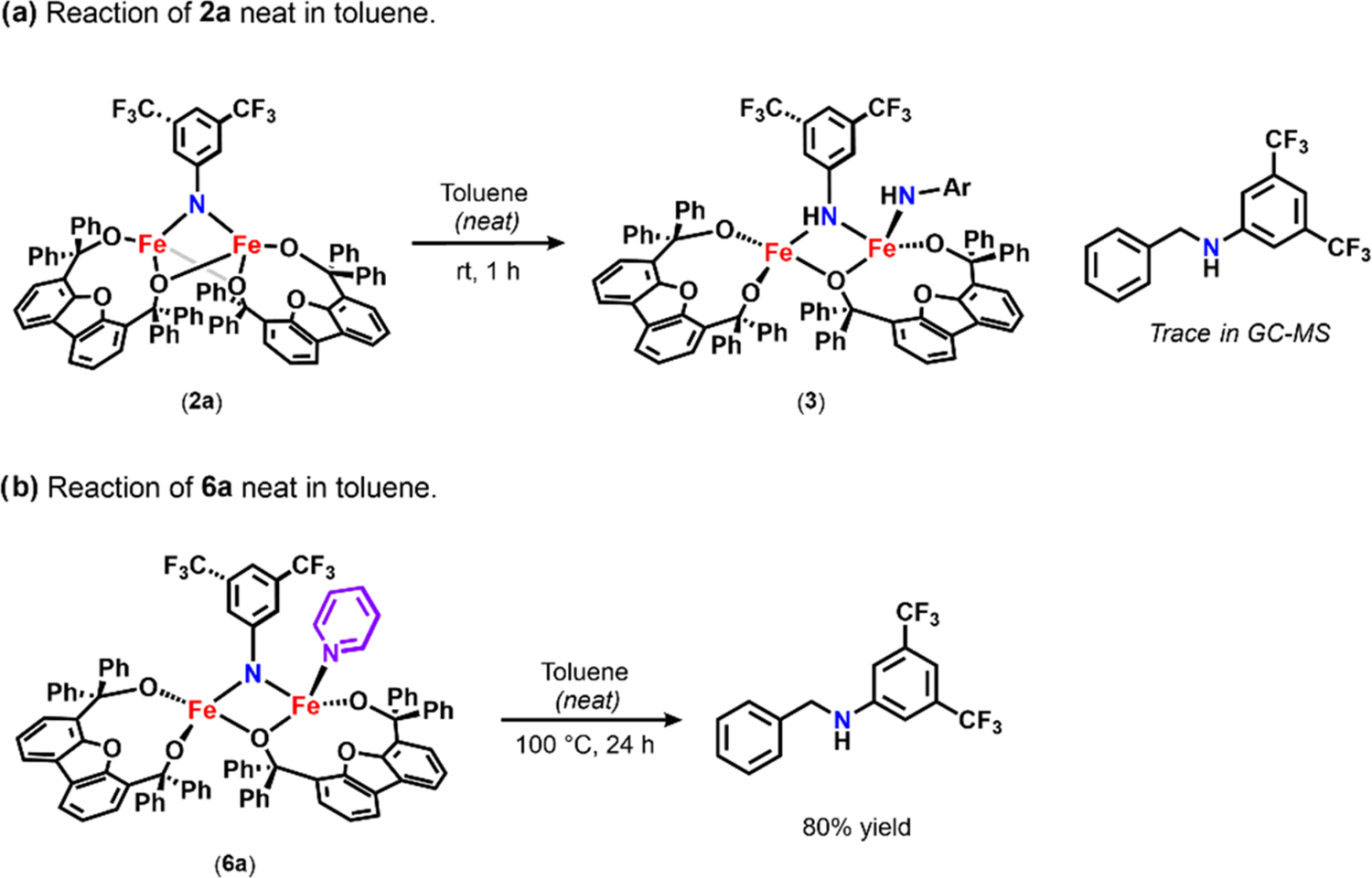
Lewis Base Effect on Toluene Amination Reactivity Mediated by (a) **2a** and (b) **6a**

Interestingly, upon the addition of excess 1,4-cyclohexadiene to **6a** at 90 °C, **3** was observed in the ^19^F NMR spectrum, indicating that the pyridine ligand had dissociated (Figure 4d). Likewise, free *p*-CF_3_Py was observed in the ^19^F NMR spectrum of the reaction of **6b** and 1,4-cyclohexadiene under the same conditions (Figure S-66). Surprisingly, unlike **2a**, reactions with **6a** or **6b** and 1,4-cyclohexadiene consumed all starting material and **3** and afforded the corresponding 3,5-bis(trifluoromethyl)phenyl aniline upon continued heating at 90 °C for 24 h. Similarly unexpected, the reaction of **6a** with toluene at 100 °C afforded the aminated product *N*-benzyl-3-5-bis(trifluoromethyl)aniline in 80% yield (Scheme 5).

Lewis base-mediated C–H bond functionalization has been previously demonstrated. A recently published monomeric cobalt-imido system^37^ was shown to manifest enhanced reactivity in the presence of pyridine, which was attributed to pyridine coordination to slow down the formation of an unreactive cobalt-tetrazido complex. Likewise, a dicobalt nitride complex was reported to require the addition of pyridine to activate the nitride unit and form the respective bridged imido species competent for HAA.^38^ Both cobalt-imido systems are prime examples of how auxiliary ligands can be leveraged to effect structural changes that enhance reactivity and provide invaluable insight into the effect of pyridine on the reactivity of our system.

To better understand the increased reactivity of **2a** upon Lewis base addition, the reaction of **6b** with 1,4-cyclohexadiene was further analyzed. Interestingly, **3** was observed in the ^19^F NMR spectrum upon HAA, as well as two additional broad peaks (Figure S-67). The generation of two new peaks in the ^19^F NMR spectrum could correspond to either (1) two new paramagnetic species or (2) a single paramagnetic species bound to both the fluorinated imido ligand and one 4-trifluoromethylpyridine ligand. Notably, the distance between the peaks corresponding to the fluorine atoms of the 4-trifluoromethylpyridine and the imido ligand of **6b** (7.91 ppm) is close to the difference in chemical shift observed between the new signals (8.11 ppm) (Figure 4d). Additionally, these signals integrate in a 2:1 ratio, as seen for **6b** and as expected for a species that contains both a single fluorinated imido and fluorinated pyridine ligand (Figure 4d). Furthermore, the chemical shifts and broadness of these peaks are reminiscent of the signal observed for asymmetric complex **4** (Figure S-15). Thus, we hypothesize that this species could be an asymmetric bridging amide complex with one Fe center bound terminally to a 4-trifluoromethylpyridine ligand. Unfortunately, this species could not be isolated to confirm its identity. Therefore, the reaction of **3** with 1 equiv of 4-trifluoromethylpyridine was attempted to further probe the identity of this complex. Gratifyingly, the same paramagnetic species was identified in the ^19^F NMR spectrum, providing additional support for the proposed bimetallic structure.

#### 1.3.3 Proposed Reaction Pathway

**Scheme 6:**
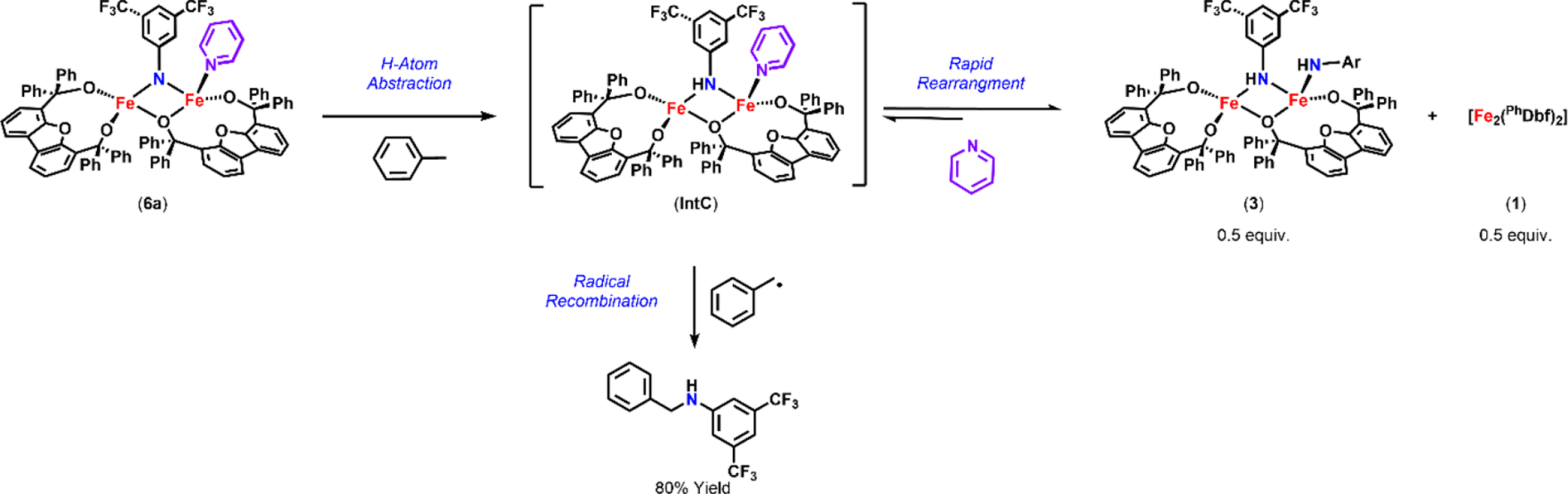
Proposed Reaction Pathway for Toluene Amination via **6a**

In light of the above results, we propose that **3** is in equilibrium with a reactive amide intermediate (Scheme 6). In the absence of Lewis base, this equilibrium favors formation of complex **3** due to its increased stability in comparison to that of the proposed symmetrical bridging amide in Scheme 4, **IntA**. As **3** has been shown to be incompetent for radical recombination, if the proposed amide intermediate does not exist in significant quantities in solution, radical recombination would not be observed. This is consistent with the observed reactivity of **2a** with toluene, where only trace amounts of aminated product can be identified in GC–MS. In comparison, the reaction of toluene with **6a** likely affords organic product in significant quantities due to pyridine shifting the equilibrium and/or aiding in the stabilization of the bridging amide intermediate (**IntC**; Scheme 6). Ultimately, this would enhance the reactive intermediate’s life span in solution and promote radical recombination, thus explaining the observed difference in reactivity. Additionally, increased concentrations of pyridine were observed to enhance the reactivity of **2a**, decreasing reaction times to 10 min (>10 equiv) or 1 h (2 equiv). However, these studies were performed qualitatively via ^19^F NMR spectroscopy and GC–MS and quantitative studies will be required to draw more significant conclusions concerning the Lewis base effects on the reaction rate.

As such, we propose that the intermediate responsible for radical recombination is a bimetallic bridging amide species (**IntA**, Scheme 4; **IntC**, Scheme 6). This is consistent with the isolation of bridging amide species **4**, the failure to isolate monomeric species via retrosynthetic methods or otherwise in the presence of excess Lewis base, and the spectroscopic features of the species formed during the reaction of **6b** with 1,4-cyclohexadiene suggestive of an asymmetric bridging amide species. Overall, our results suggest that both HAA and radical recombination occur at the dinuclear site.

## 2 Conclusions

In this report, we investigate the effect of ligand design on the nuclearity of MLMB species competent for C–H bond functionalization. We hypothesized that alkoxide ligands were suitable for the design of bimetallic iron imido systems, as they have enhanced bridging capabilities in comparison to the previously employed dipyrrin ligand. Additionally, the weak-field properties of alkoxide ligands promote high-spin states necessary for the desired reactivity.

The supporting alkoxide ligands facilitated the formation of reactive diiron bridging imido species in both the absence (**2a**) and presence of Lewis base (**6a** and **6b**). Furthermore, the addition of excess pyridine to **2a** did not afford a monomeric imido species, suggesting that the alkoxide ligands can maintain dinuclearity in environments that typically favor the formation of monometallic complexes. Additionally, when **2a** and **6a** promoted HAA from toluene, inert bis(amide) complex **3** was observed by ^19^F NMR spectroscopy. However, the presence of pyridine promoted the consumption of **3** and radical recombination to afford the aminated product *N*-benzyl-3-5-bis(trifluoromethyl)aniline in good yield, while in the absence of pyridine, the reaction did not consume **3** in significant quantities, affording only trace aminated product. Considering the observed reactivity, literature precedence for Lewis base-enhanced reactivity of MLMB species, and our inability to isolate monomeric iron imido and amide species, our studies suggest that the reactive intermediate responsible for the radical recombination is a diiron bridging amide species that required pyridine coordination to effectively promote C–N bond formation. Overall, our findings illustrate the potential for alkoxide ligands to support bimetallic MLMB systems competent for nitrene group transfer as well as provide insight into the properties of the bimetallic iron imido complexes that promote reactivity that will aid in the design of future dinuclear MLMB systems.

## 3 Experimental Section

### 3.1 General Considerations

All manipulations of metal complexes were carried out in the absence of water and dioxygen using standard Schlenk techniques or in a Vigor inert atmosphere dry box under a dinitrogen atmosphere. The bis-alkoxide ligand was synthesized as previously reported.^39^ All glassware was oven-dried for a minimum of 1 h and cooled in an evacuated antechamber prior to use in the dry box. Benzene, diethyl ether, hexane, pentane, toluene, tetrahydrofuran, 1,2-difluorobenzene, and trifluorotoluene were dried over 4 Å molecular sieves (Research Catalysts) prior to use. Chloroform-*d* was purchased from Cambridge Isotope Laboratories and used as received. Benzene-*d*_6_ was purchased from Cambridge Isotope Laboratories and was degassed and stored over 4 Å molecular sieves prior to use. Pyridine, 3,5-bis(trifluoromethyl)aniline, 9-azabicyclo[3.1.3]Nonane-*N*-Oxyl, *n*-butyllithium, and benzophenone were purchased from Aldrich. Dibenzofuran, 4-(trifluoromethyl)pyridine, 2,4,6-trimethylaniline, 2,6-diisopropylaniline, triphenylmethanol, fluorene, 2,4,6-tri-*tert*-butylphenol, 1,4-cyclohexadiene, and 4-*tert*-butylaniline were purchased from Oakwood Chemical. 1,4-Cyclohexadiene was distilled to remove radical stabilizer, degassed, and stored over 4 Å molecular sieves (Research Catalysts) prior to use. Aniline and *N*,*N*,*N*′,*N*′-tetramethylethylethylenediamine were purchased from Sigma. 2-Hydroxy-2-azaadamantane was purchased from Tokyo Chemical Industry (TCI). 34 Celite 545 (J. T. Baker) was dried in a Schlenk flask for 24 h under dynamic vacuum while heating to at least 150 °C prior to use in a dry box. Alumina gel 32–63 μ (AIC, Framingham, MA) was used as received. All aryl azides^40−43^ and the diiron starting material were [Fe_2_(Dbf)_2_] (1)^25^ were synthesized as previously reported. Yields of the metal complexes were measured as isolated yields. The yield reactions producing organic reagents were determined via an internal standard of ferrocene (dehydrogenation reaction) or 1,2-difluorobenzene (**2a** styrene aziridination/**6a** toluene amination).

### 3.2 Characterization and Physical Methods

^1^H NMR spectra were recorded on a Bruker Avance III 600 MHz with a TCI LN2 Prodigy probe, a Bruker Avance II 500 MHz with a BBO LN2 Prodigy probe, or a JEOL ECZL400S 400 MHz with a Royal HFX probe system. ^19^F NMR spectra were recorded on a Bruker Avance III 600 MHz with a TCI LN2 Prodigy probe or a JEOL ECZL400S 400 MHz with a Royal HFX probe system. ^1^H and ^13^C NMR chemical shifts are reported relative to SiMe_4_ using the chemical shift of residual solvent peaks as reference. ^19^F NMR chemical shifts are reported relative to an internal standard of trifluorotoluene and were all referenced. ^19^F spectra were recorded on a JEOL ECZL400S 400 MHz with a Royal HFX probe system.^44^ All NMR spectra were collected at room temperature, unless otherwise stated. Elemental analyses were carried out by Midwest Microlab (Indianapolis, IN). Zero-field ^57^Fe Mössbauer spectra were measured with a constant acceleration spectrometer (SEE Co, Minneapolis, MN) at 90 K. Isomer shifts are quoted relative to Fe foil at room temperature. Data was analyzed and simulated with Igor Pro 6 software (WaveMetrics, Portland, OR) using Lorentzian fitting functions. Samples were prepared by suspending 25–50 mg of the compound in sufficient Paratone oil and immobilizing it by rapid freezing in liquid nitrogen. EPR spectra were obtained on a Bruker EMX-Plus CW-EPR or Bruker EleXsys E-500 CW-EPR spectrometer at 80 K unless otherwise stated. Spectra were measured as frozen toluene glasses at a microwave power of 0.6325–2 mW unless otherwise noted (Parameters: Modulation Amplitude: 4 G, Scans: 5, Attenuation: 35 dB, Center Field: 3000 G; Sweep Width: 6000 G or Modulation Amplitude:10 G, Scans: 5, Attenuation: 20 dB, Center Field: 3200 G; Sweep Width: 6000 G). Spectral simulations incorporating spin state and rhombicity were performed using VisualRhombo.^45^ Fourier transform infrared (FTIR) spectra were collected on a Bruker Alpha II-Platinum FT-IR Spectrometer with Platinum Diamond-ATR. GC–MS experiments were performed using an Agilent 7890 Series GC system coupled to an HP 5975 mass selective detector.

### 3.3 X-ray Diffraction Techniques

All structures were collected on a Rigaku Oxford Diffraction Synergy-S diffractometer equipped with a HyPix6000HE detector and operating with Cu Kα radiation or Mo Kα source. Data collection, unit cell refinement, and data processing were carried out with CrysAlisPro,^46^ while structures were solved utilizing SHELXT^47^ and refined using SHELXL^48^ or OLEX2.refine^49^ via Olex2.^49^ Olex2, PovRay,^50^ and ORTEP^51^ applications were used to generate structure graphics. Crystals were mounted on a cryoloop or glass fiber pin by using Paratone N oil. Structures were collected at 100 K. All nonhydrogen atoms were refined anisotropically. Hydrogen atoms were placed at idealized positions and refined by using a riding model. The isotropic displacement parameters of all hydrogen atoms were fixed to 1.2 times the atoms to which they are linked (1.5 times for methyl groups). Further details on structures are noted in the Supporting Information.

### 3.4 Computational Methods

Computations for imido complexes **2a** and **6a** and for complexes **3** and **4** were carried out with the QChem software package.^52^ Several basis sets were considered, and the results are reported in Table S-9. J coupling constants are computed using the Yamaguchi projection formula:^30^
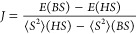


Computations were carried out utilizing the ORCA 4.2.1^53^ program package for all Mössbauer calculations. The B3LYP^54,55^ functional was used with the def2-TZVP (Fe, O, N, Cl) and def2-SV(P) (C, H) basis sets.^56−58^ For single-point calculations and property calculations, the def2-TZVP/J (Fe, O, N, Cl) and def2-SVP/J (C, H) auxiliary basis sets^59^ were employed to utilize the RIJCOSX^60^ approximation for accelerating the calculation. All coordinates were taken from X-ray structures.

### 3.5 Mössbauer

Mössbauer parameters were obtained from additional single-point calculations, following methods described by F. Neese.^61,62^ Quadrupole splittings (Δ*E*_Q_) were calculated from electric field gradient, eq S.1.
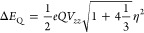
S.1

The nuclear quadrupole moment *Q*(^57^Fe) was taken to be 0.16 barn.^61^ The principal tensor components of the EFG are *V*_*xx*_, *V*_*yy*_, and *V*_*zz*_, from which the asymmetry parameter η = (*V*_*xx*_ – *V*_*yy*_)/*V*_*zz*_ can be defined.

Isomer shifts (δ) were calculated from the electron density at the nucleus ρ_0_, using a linear equation, eq S.2,^61^ with constants determined by fitting the calculated densities to experimental isomer shifts for a series of iron alkoxide complexes synthesized in the lab (the basis sets and functional described above were used for all structures. X-ray coordinates were used, and spin states were assigned based on experimental Mössbauer data).
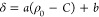
S.2

For this series of compounds, the parameters were determined to be *C* = 11,580 au^–3^, *a* = −0.359 au^3^ mm s^–1^, and *b* = 1.295 mm.

### 3.6 SQUID Magnetometry

Preparation of sample used for magnetic characterization was performed in an air/moisture-free environment inside a N_2_ filled glovebox and using Standard Schlenk techniques. Material used for magnetic characterization was used as received (coarse powder) and was subsequently ground into a fine powder using a plastic spatula. The ground material (20–30 mg) was then placed into the bottom of high purity glass NMR tube along with eicosane (40–60 mg). The NMR tube containing the sample/eicosane mixture was then equipped with a glass Schlenk line adapter and was sealed into a ∼4 cm tube under vacuum on the Schlenk line. The solid eicosane was melted over the paramagnetic sample between 38 and 43 °C while being agitated to avoid the isolation of air bubbles within the solid matrix.

Magnetic characterization was carried out using a Quantum Design MPMS 3 SQUID Magnetometer. The direct current (dc) magnetic susceptibility was collected using an external 1.0 T magnetic field between 300 and 2 K. A diamagnetic correction was calculated using Pascal’s Constants^63^ and was included in the calculation of the magnetic susceptibility to account for the diamagnetism of the compound core electrons and eicosane. The field dependence of the magnetization was collected between 0 and 7 T at 2 K, 4 K, 6 K, and 8 T and 0 and 4 T at 100 K.

### 3.7 Safety Statement

Caution! Azides are incompatible with acids and metals. During synthesis do not expose sodium azide (NaN_3_) to any metal, including metal spatulas.

Caution! Starting material Fe_2_Mes_4_ is pyrophoric and should be handled with caution under inert conditions. All excess Fe_2_Mes_4_ and glassware used during synthesis should be quenched with acetonitrile before removal from the glovebox to avoid ignition upon exposure to air.

### 3.8 Metal Complex Syntheses

#### 3.8.1 [Fe_2_(^Ph^Dbf)_2_(μ-NAr)] (**2a**) and (**2b**)

##### 3.8.1.1 General Procedure A

**1** (100.0 mg, 0.085 mmol) was dissolved in minimal benzene. An aliquot of azide stock solution (1 equiv, 0.085 mmol) in benzene was added to **1a** and stirred for 5 min at room temperature until bubbling was no longer observed and complete consumption of **1** was confirmed via NMR. The complex was lyophilized to afford a colored powder.

#### 3.8.2 [Fe_2_(^Ph^Dbf)_2_(μ-NC_8_H_3_F_6_)] (**2a**)

Upon lyophilization, a blue powder was obtained (**2a**) in 90% yield (72.9 mg). Crystals suitable for X-ray diffraction were grown from a hexane solution with drops of trifluorotoluene at −35 °C. ^**1**^**H NMR** (600 MHz, C_6_D_6_): δ 10.06 (br s), 8.70 (br s), 8.03 (br s), 6.73 (br s), −46.20 (br s), −49.37 (br.s). ^**19**^**F NMR** (376 MHz, C_6_D_6_): δ −79.27 ppm (br.s). Anal. calcd for C_84_H_55_Fe_2_O_6_F_6_N_1_: C 72.06; H 3.96, N 1.00; Found C 71.93, H 4.10, N 1.05. Zero-field ^57^Fe Mössbauer (90 K) δ = 0.49 mm s^–1^, |Δ*E*_Q_| = 1.85 mm s^–1^.

#### 3.8.3 [Fe_2_(^Ph^Dbf)_2_(μ-NC_10_H_13_)] (**2b**)

Upon lyophilization, a blue powder was obtained (**2b**) in 89% yield (20 mg). Crystals suitable for X-ray diffraction were grown from a hexane solution with drops of trifluorotoluene at −35 °C. ^**1**^**H NMR** (600 MHz, C_6_D_6_): δ 34.50 (br s), 13.84 (br s), 9.33 (br s), 8.13 (br s), 8.01 (br.s). 7.56 (br.s), 6.77 (br.s), 4.01 (br.s), 2.10 (br.s), −31.10 (br.s). Anal. calcd for C_86_H_65_Fe_2_O_6_N_1_: C 78.24; H 4.96, N 1.06; Found C 76.16, H 5.10, N 1.11.

#### 3.8.4 [Fe_2_(^Ph^Dbf)_2_(μ-NHC_8_H_3_F_6_)(NHC_8_H_3_F_6_)] (**3**)

Complex **2a** (100 mg, 0.071 mmol) was dissolved in 3 mL of benzene. Two drops of 1,4-cyclohexadiene were added to the solution. The solution was heated at 80 °C overnight in an oil bath (5 h for 10 mg reaction) before lyophilization to afford a dark blue powder in 92% yield (128 mg). It was noted that this reaction requires more time if it is too diluted. Crystals suitable for X-ray diffraction were grown from a hexane solution at −35 °C. ^**1**^**H NMR** (600 MHz, C_6_D_6_): δ 45.39 (br.s), 21.17 (br.s), 21.00 (br.s), 20.33 (br.s), 18.00 (br.s), 17.08 (br.s) 16.63 (br.s), 14.25 (br.s), 13.81 (br.s), 12.68 (br.s), 10.28 (br.s), 5.91 (br.s) 5.83 (br.s), 5.46 (br.s), 5.42 (br.s) 4.55 (br.s), 3.66 (br.s), 0.31 (br.s), −1.10 (br.s), −7.77 (br.s) −12.02 (br.s), −13.93 (br.s), −14.42 (br.s) −22.16 (br.s), 3.66 (br.s). ^**19**^**F NMR** (376 MHz, C_6_D_6_): δ −119.47 ppm (br.s). Anal. calcd for C_92_H_60_Fe_2_O_6_F_12_N_2_: C 67.83; H 3.71, N 1.72; Found C 67.83, H 4.44, N 1.09. Zero-field ^57^Fe Mössbauer (90 K) δ = 0.49 mm s^–1^, |Δ*E*_Q_| = 1.78 mm s^–1^; EPR (toluene, 80 K): *g*_eff_ = 4.27.

*Note: This complex is believed to exist in solution with the dimeric [Fe_2_(^Ph^Dbf)_2_] (**1**) starting material as this complex should be generated upon rearrangement. Complex **3** is highly soluble in all solvents, including hexanes and pentane, making further purification challenging. Therefore, we believe that this explains the deviation in the calculated vs experimental EA values.

#### 3.8.5 [Fe_2_(^Ph^Dbf)_2_(μ-NHC_8_H_3_F_6_)(OC_19_H_15_)] (**4**)

Complex **2a** (15 mg, 0.011 mmol) was dissolved in 5 mL of benzene. Triphenylmethanol (1 equiv, 2.8 mg) was dissolved in minimal benzene and added to **2a**. The solution was stirred at room temperature for 5 min before being lyophilized to afford a brown powder in quantitative yield (>95%; 17 mg). Crystals suitable for X-ray diffraction were grown from a hexane solution at −35 °C. ^**1**^**H NMR** (600 MHz, C_6_D_6_): δ 14.38 (br.s). ^**19**^**F NMR** (376 MHz, C_6_D_6_): δ −99.57 ppm (br s), −103.77 (br.s). Anal. calcd for C_103_H_71_Fe_2_O_7_F_6_N_1_: C 75.23; H 4.35, N 0.85; Found C 72.60, H 4.51, N 0.93. Zero-field ^57^Fe Mössbauer (90 K) 50% δ = 0.47 mm s^–1^, |Δ*E*_Q_| = 2.15 mm s^–1^, 50% δ = 0.51 mm s^–1^, |Δ*E*_Q_| = 1.34 mm s^–1^. EPR (toluene, 80 K): *g*_eff_ = 4.28.

*Note: Complexes **3** and **4** will degrade if stored at room temperature or in solution over the course of a few days. Samples will degrade rapidly upon exposure to oxygen.

#### 3.8.6 [Fe_2_(^Ph^Dbf)_2_(μ-NHC_8_H_3_F_6_)(Py-R)] (**6a**), (**6b**)

##### 3.8.6.1 Procedure A

Complex **2a** (20 mg, 0.014 mmol) was dissolved in minimal benzene. An aliquot of pyridine or 4-(trifluoromethyl)pyridine stock solution (1 equiv, 0.074 mmol) in benzene was added to **1a** and stirred for 5 min at room temperature. The complex was triturated with hexanes five times to remove all excess pyridine and lyophilized in benzene to afford a colored powder: (**6a**)—blue, (**6b**)—blue in quantitative yield (>95%, 21 mg) and quantitative yield (>95%, 22 mg), respectively.

##### 3.8.6.2 Procedure B

Complex **5a** (20 mg, 0.0027 μmol) or **5b** (20 mg, 0.0023 μmol) was dissolved in minimal benzene. An aliquot of azide stock solution (0.5 equiv, 0.085 mmol) in benzene was added to **1a** and stirred for 5 min at room temperature until bubbling was no longer observed. The complex was lyophilized to afford a colored powder ((**6a**)—blue, (**6b**)—blue) in quantitative yield (>95%, 22 mg; crude yield for **6a**—see note below) and quantitative yield (>95%, 22, 21 mg), respectively.

*Note: Procedure A is preferred and used for reactivity studies with complexes (**6a**/**6b**). When procedure B is used with monomeric starting material **5a** an unidentified species at −105 ppm is present in the ^19^F NMR spectrum alongside the expected peak at −74 ppm. The −105 ppm species is not present if procedure A is used or if excess pyridine is added to **2a**, so it is not believed to be a monomeric imido species, but another species formed upon formation of the pyridine-bound imido when the monomeric starting material is utilized. This same peak is not observed if **5b** is utilized as a starting material.

*Note: These complexes (**6a**, **6b**) will degrade into organic materials if stored at room temperature or in solution over the course of a few days. Samples will degrade rapidly when exposed to oxygen.

#### 3.8.7 [Fe_2_(^Ph^Dbf)_2_(μ-NHC_8_H_3_F_6_)(NC_5_H_5_)] (**6a**)

A blue powder in >95% yield (22 mg) was obtained.^1^H NMR (600 MHz, C_6_D_6_): δ 22.75 (br.s), 22.75 (br.s), 17.38 (br.s), 14.56 (br.s), 9.49 (br.s), 9.25 (br.s), 8.81 (br.s), 8.54 (br.s), 8.30 (br.s), 8.09 (br.s), 8.02 (br.s), 7.83 (br.s), 7.75 (br.s), 7.72 (br.s), 7.70 (br.s), 7.63 (br.s), 7.43 (br.s), 7.33 (br.s), 7.31 (br.s), 7.29 (br.s), 6.57 (br.s), 6.46 (br.s), 5.06 (br.s). ^19^F NMR (376 MHz, C_6_D_6_): δ −75.31 (br.s). Anal. calcd for C_89_H_61_Fe_2_O_6_F_6_N_2_: C 72.22; H 4.15, N 1.89; Found C 71.4, H 4.38, N 1.98. Zero-field ^57^Fe Mössbauer (90 K) δ = 0.47 mm s^–1^, |Δ*E*_Q_| = 1.89 mm s^–1^.

#### 3.8.8 [Fe_2_(^Ph^Dbf)_2_(μ-NHC_8_H_3_F_6_)(NC_6_H_4_F_3_)] (**6b**)

^**1**^**H NMR** (400 MHz, C_6_D_6_): A blue powder in >95% yield (22 mg) was obtained. δ 20.82 (br.s), 9.45 (br.s), 8.75 (br.s), 8.44 (br.s), 8.08 (br.s), 7.72 (br.s), 7.71 (br.s), 7.48 (br.s), 7.36 (br.s), 6.57 (br.s), 4.87 (br.s). ^**19**^**F NMR** (376 MHz, C_6_D_6_): δ −65.09 (br s), −73.07 (br.s). Anal. calcd for C_90_H_59_Fe_2_O_6_F_6_N_1_: C 69.82; H 3.91, N 1.81; Found C 65.24, H 3.92, N 1.81.

### 3.9 Stoichiometric Reactions

#### 3.9.1 For Zero-Field ^57^Fe Mössbauer analysis

Under an inert atmosphere, solid Fe_2_(^Ph^Dbf)_2_ (**1**) was dissolved in minimal benzene. The desired azide (1 equiv) in minimal benzene was added to the solution and stirred for 5–10 min at room temperature. The reaction mixture was then lyophilized, and the powder was shipped overnight. Samples were packed in tape-sealed GC–MS vials inside a tape-sealed scintillation vial or a sealed and taped pressure vial packed with dry ice. The samples were then analyzed via zero-field ^57^Fe Mössbauer spectroscopy.

#### 3.9.2 For ^1^H and ^19^F NMR Analysis

Under an inert atmosphere, a solution of the desired azide (1 equiv) in benzene-*d*_6_ was layered onto a frozen solution of Fe_2_(^Ph^Dbf)_2_ (**1**) in benzene-*d*_6_ in an NMR tube. The reaction mixture was thawed immediately prior to acquisition of the initial spectrum. Once the resulting complexes were determined to be stable, the reactions were repeated at room temperature.

#### 3.9.3 For EPR Analysis

Under an inert atmosphere, 2 mg of solid compound was weighed out and diluted in toluene or benzene if unwanted HAA was a concern (**2a**, **2b**, **6a**, and **6b**). This solution was then added to an EPR tube and used for data acquisition. This was repeated for all stable species.

A toluene solution of the desired O–H/C–H bond reagent (1 equiv) was added to a solution of Fe_2_^Ph^Dbf_2_(μ*-*NC_6_H_3_-3,5-(CF_3_)_2_) (**2a**) in minimal toluene in an EPR tube. These samples were immediately used for data acquisition. This was repeated for all stoichiometric reactions.

#### 3.9.4 Styrene Aziridination

Under inert atmosphere, solid Fe_2_(^Ph^Dbf)_2_ (**1**) was weighed out in a vial and a styrene solution of 3,5-bis(trifluoromethyl)phenyl azide was added. The reaction mixture was stirred until bubbling was no longer observed (∼30 min). The solution was concentrated *in vacuo* and eluted through a neutral alumina gel using 10:1 DCM:methanol as eluent to remove paramagnetic materials. Solvent was removed and the remaining product was dried overnight in a vacuum oven at 50 °C. Formation of 1-(3,5-bis(trifluoromethyl)phenyl)-2-phenylaziridine upon reaction with styrene was confirmed via ^1^H and ^19^F NMR. Spectral data were consistent with previously reported characterization of the product.^26^ Yield was determined via an internal standard of 1,2-difluorobenzene. Internal standard was added directly to the NMR sample (0.8 mL of deuterated solvent) with a microsyringe. Yield (95%, 25 mg).

### 3.10 Reactions with Fe_2_(^Ph^Dbf)_2_(μ-NC_6_H_3_-3,5-(CF_3_)_2_) (**2a**) with O–H Substrates

#### 3.10.1 Reactions with 2-Hydroxy-2-azaadamantane

Stoichiometric 2-hydroxy-2-azaadamantane was added to a frozen benzene-*d*_6_ solution of [Fe_2_(^Ph^Dbf)_2_(μ-NC_8_H_3_F_6_)] (**2a**) in an NMR tube. An initial ^1^H and ^19^F NMR spectra were taken upon thawing. Low-temperature EPR spectra were obtained immediately after thawing a frozen toluene solution of [Fe_2_(^Ph^Dbf)_2_(μ-NC_8_H_3_F_6_)] (**2a**) and 2-hydroxy-2-azaadamantane (1 equiv).

#### 3.10.2 Reactions with 2,4,6-Tri-*tert*-Butylphenol

Stoichiometric 2,4,6-tri-*tert*-butylphenol was added to a frozen benzene-*d*_6_ solution [Fe_2_(^Ph^Dbf)_2_(μ-NC_8_H_3_F_6_)] (**2a**) in a J-Young tube and heated overnight at 80 °C. An initial ^1^H and ^19^F NMR spectra were taken upon thawing. Low- and room-temperature EPR spectra were obtained in a benzene solution. This reaction does not go to completion, but the formation of [Fe_2_(^Ph^Dbf)_2_(μ-NHC_8_H_3_F_6_)(NHC_8_H_3_F_6_)] (**3**) is observed in the ^19^F NMR and organic radical in the EPR spectra.

#### 3.10.3 Reactions with Triphenylmethanol

Stoichiometric triphenylmethanol was added to a frozen benzene-*d*_6_ solution [Fe_2_(^Ph^Dbf)_2_(μ-NC_8_H_3_F_6_)] (**2a**) in an NMR tube. ^1^H and ^19^F NMR spectra were taken after 5 min of stirring, and complete color change was noted (brown). This reaction reached completion to form [Fe_2_(^Ph^Dbf)_2_(μ-NHC_8_H_3_F_6_)(OC_19_H_15_)] (**4**). Low-temperature EPR spectra were obtained for a frozen toluene solution of the isolated product [Fe_2_(^Ph^Dbf)_2_(μ-NHC_8_H_3_F_6_)(OC_19_H_15_)] (**4**).

### 3.11 Reactions with Fe_2_(^Ph^Dbf)_2_(μ-NC_6_H_3_-3,5-(CF_3_)_2_) (**2a**) with C–H-Bond-Containing Substrates

#### 3.11.1 Reactions with 9*H*-Fluorene

Excess substrate was dissolved in minimal deuterated benzene [Fe_2_(^Ph^Dbf)_2_(μ-NC_8_H_3_F_6_)] (**2a**) (<20 mg scale) in a J-Young tube. An initial ^1^H and ^19^F NMR spectra were taken and the reaction mixture was then heated at 80 °C overnight. Reactions with 9*H*-fluorene do not completely convert to [Fe_2_(^Ph^Dbf)_2_(μ-NHC_8_H_3_F_6_)(NHC_8_H_3_F_6_)] (**3**) likely due to the steric bulk of the substrate.

#### 3.11.2 Reactions with 1,4-Cyclohexadiene

Excess substrate was added to a benzene-*d*_6_ solution [Fe_2_(^Ph^Dbf)_2_(μ-NC_8_H_3_F_6_)] (**2a**) in a J-Young tube (<20 mg scale) or sealed pressure vessel (>20 mg scale). An initial ^1^H and ^19^F NMR spectra were taken and the reaction mixture was then heated at 80 °C overnight. These reactions do not reach completion to form an organic product, but the formation of [Fe_2_(^Ph^Dbf)_2_(μ-NHC_8_H_3_F_6_)(NHC_8_H_3_F_6_)] (**3**) is observed in the ^19^F NMR spectra.

#### 3.11.3 Reactions with Toluene

Reaction was done neat in toluene with [Fe_2_(^Ph^Dbf)_2_(μ-NC_8_H_3_F_6_)] (**2a**) (<20 mg scale) in an NMR tube. Overall volume was approximately 0.8 mL. An initial ^1^H and ^19^F NMR spectra were taken, and the reaction was either left at room temperature or heated (J-Young tube used). Reactions with toluene convert fully to [Fe_2_(^Ph^Dbf)_2_(μ-NHC_8_H_3_F_6_)(NHC_8_H_3_F_6_)] (**3**), but do not proceed to make organic product after 48 h in the ^19^F NMR at high temperature. Samples were checked by GC–MS to confirm the formation of 3,5-bis(trifluoromethyl)aniline in GC–MS after the reaction was quenched with methanol.

### 3.12 Reactions with [Fe_2_(^Ph^Dbf)_2_(μ-NHC_8_H_3_F_6_)(NC_5_H_5_)] (6a) and [Fe_2_(^Ph^Dbf)_2_(μ-NHC_8_H_3_F_6_)(NC_6_H_5_F_3_)] (**6b**) with C–H-Bond-Containing Substrates

#### 3.12.1 Reactions with 1,4-Cyclohexadiene

Excess substrate was added to a benzene-*d*_6_ solution [Fe_2_(^Ph^Dbf)_2_(μ-NHC_8_H_3_F_6_)(NC_5_H_5_)] (**6a**) or [Fe_2_(^Ph^Dbf)_2_(μ-NHC_8_H_3_F_6_)(NC_6_H_5_F_3_)] (**6b**) in a J-Young tube. An initial ^1^H and ^19^F NMR spectra were taken and the reaction mixture was then heated at 100 °C overnight. Upon removal of 1,4-cyclohexadiene under vacuum, samples were checked by GC–MS to confirm the formation of 3,5-bis(trifluoromethyl)aniline.

#### 3.12.2 Reactions with Toluene

Excess substrate was added to a benzene-*d*_6_ solution [Fe_2_(^Ph^Dbf)_2_(μ-NHC_8_H_3_F_6_)(NC_5_H_5_)] (**6a**) in a J-Young tube. An initial ^1^H and ^19^F NMR were taken and the reaction mixture was then heated at 100 °C overnight. The ^19^F NMR showed consumption of the starting material and formation of the expected functionalized product, 3,5-*N*-benzyl-3,5-bis(trifluoromethyl)aniline. Upon removal of the C–H substrate under vacuum, organic products were identified via GC–MS and ^1^H and ^19^F NMR.^64^ Small quantities of bibenzyl and 3,5-bis(trifluoromethyl)aniline were detected by GC–MS in addition to 3,5-*N*-benzyl-3,5-bis(trifluoromethyl)aniline. Solvent was removed, and the remaining product was dried overnight in a vacuum oven at 50 °C. Yield was determined via internal standard of 1,2-difluorobenzene. Internal standard was added directly to the NMR sample (0.8 mL of deuterated solvent) with a microsyringe. Yield (80%, 2.1 mg)

### 3.13 Reactions with [Fe_2_(^Ph^Dbf)_2_(μ-NHC_8_H_3_F_6_)(NC_5_H_5_)] (**3**)

#### 3.13.1 Reactions with Radicals (^•^CPh_3_ and ^•^AdNO)

Complex [Fe_2_(^Ph^Dbf)_2_(μ-NHC_8_H_3_F_6_)(NC_5_H_5_)] (**3**) was dissolved in minimal benzene-*d*_6_, and 1 equiv of the desired reagent in minimal benzene-*d*_6_ was layered on top of the frozen solution. ^1^H and ^19^F NMR were obtained immediately after thawing. Gomberg’s dimer was synthesized according to the reported procedure.^35^

#### 3.13.2 Reactions with 4-Trifluoromethylpyridine

Complex [Fe_2_(^Ph^Dbf)_2_(μ-NHC_8_H_3_F_6_)(NC_5_H_5_)] (**3**) was dissolved in minimal benzene-*d*_6_ and 0.9 equiv of 4-trifluoromethylpyridine was layered on top of the frozen solution. Less than 1 equiv of 4-trifluoromethylpyridine was utilized to ensure the reaction did not go to completion in case a slight excess of pyridine was present from error in the measurement. ^1^H and ^19^F NMR spectra were obtained immediately after thawing.

## Supporting Information Available

^1^H and ^19^F (where appropriate) NMR, IR, and EPR spectra (80 K) for all complexes synthesized; SQUID magnetometry data and fit for **2a**; ^1^H NMR spectra for reactions of **1** and **5a** with all aryl azides; ^19^F NMR for all reactions of **1**, **6a**, and **6b** with styrene, 1,4-cyclohexadiene, C–H, and O–H bond substrates and radical substrates; X-ray crystallography tables and figures for **2a**, **2b**, **3**, **4**, and **6a** (PDF)
