# Supplementary material for: Lewis Base-Enhanced C–H Bond Functionalization Mediated by a Diiron Imido Complex
Source: Inorg Chem. 2025 Jan 24;64(5):2217–31. doi: 10.1021/acs.inorgchem.4c03922 (PMC11815839; doi:10.1021/acs.inorgchem.4c03922)
Supplement: Supplementary file 1 — ic4c03922_si_001.pdf [file ic4c03922_si_001.pdf]

Lewis Base Enhanced C-H Bond Functionalization Mediated by a Diiron Imido Complex

Reilly K. Gwinn,*[,1] Trevor P. Latendresse,[2] Owen N. Beck,[1] Carla Slebodnick,[1] Nicholas J. Mayhall,[1] Claire E. Casaday,[2] and Diana A. Thornton*[,1]

[1] Department of Chemistry, Virginia Polytechnic Institute and State University, Blacksburg, VA 24061

[2] Department of Chemistry and Chemical Biology, Harvard University, Cambridge, MA 02138

Table of Contents

Zero-field ^{57}Fe Mössbauer of relevant species 6

Figure S-1. Zero-field ^{57}Fe Mössbauer of [$Fe_2(^{Ph}Dbf)_2(\mu{-}NC_8H_3F_6)$] (2) collected at 90 K. 6

Figure S-2. Zero-field ^{57}Fe Mössbauer of [$Fe_2(^{Ph}Dbf)_2(\mu$-$NHC_8H_3F_6)(NHC_8H_3F_6)$] (3) collected at 90 K. 7

Figure S-3. Zero-field ^{57}Fe Mössbauer of [$Fe_2(^{Ph}Dbf)_2(\mu$-$NHC_8H_3F_6)(OC_{19}H_{15})$ (4) collected at 90 K. 8

Figure S-4. Zero-field ^{57}Fe Mössbauer of [$Fe_2(^{Ph}Dbf)_2(\mu$-$NHC_8H_3F_6)(NC_5H_5)$] (6a) collected at 90 K. 9

Table S-1. Calculated and experimental ^{57}Fe Mossbauer isomer shifts and $|\Delta E_Q|$ values. 10

Figure S-5. Magnetization data for [$Fe_2(^{Ph}Dbf)_2(\mu{-}NC_8H_3F_6)$] (2a). 11

Figure S-6. Magnetization data for [$Fe_2(^{Ph}Dbf)_2(\mu{-}NC_8H_3F_6)$] (2a). 12

Figure S-8. Magnetization data for [$Fe_2(^{Ph}Dbf)_2(\mu{-}NC_8H_3F_6)$] (2a) at 0.1 and 1.0 T. 14

Figure S-9. ^{1}H NMR (600 MHz) spectrum for paramagnetic [$Fe_2(^{Ph}Dbf)_2(\mu{-}NC_8H_3F_6)$] (2a) 15

Figure S-10. ^{19}F NMR (376 MHz) spectrum for paramagnetic [$Fe_2(^{Ph}Dbf)_2(\mu{-}NC_8H_3F_6)$] (2a) 16

Figure S-11. ^{1}H NMR (600 MHz) spectrum for paramagnetic [$Fe_2(^{Ph}Dbf)_2(\mu{-}NC_8H_{13})$] (2b). 17

Figure S-12. ^{1}H NMR (600 MHz) spectrum for paramagnetic [$Fe_2(^{Ph}Dbf)_2(\mu{-}NHC_8H_3F_6)(NHC_8H_3F_6)$] (3). 18

Figure S-13. ^{19}F NMR (376 MHz) spectrum for paramagnetic [$Fe_2(^{Ph}Dbf)_2(\mu{-}NHC_8H_3F_6)(NHC_8H_3F_6)$] (3) referenced to trifluorotoluene (-62.74 ppm). 19

Figure S-14. ^{1}H NMR (600 MHz) spectrum for paramagnetic [$Fe_2(^{Ph}Dbf)_2(\mu{-}NHC_8H_3F_6)(OC_{19}H_{15})$] (4). 20

Figure S-15. ^{19}F NMR (376 MHz) spectrum for paramagnetic [$Fe_2(^{Ph}Dbf)_2(\mu{-}NHC_8H_3F_6)$ $(OC_{19}H_{15})$] (4) 21

Figure S-16. ^{1}H NMR (600 MHz) spectrum for paramagnetic [$Fe_2(^{Ph}Dbf)_2(\mu{-}NC_8H_3F_6)(NC_5H_5)$] (6a). 22

Figure S-17. ^{19}F NMR (376 MHz) spectrum for paramagnetic [$Fe_2(^{Ph}Dbf)_2(\mu{-}NC_8H_3F_6)(NC_5H_5)$] (6a) 23

Figure S-18. ^{1}H NMR (600 MHz) spectrum for paramagnetic [$Fe_2(^{Ph}Dbf)_2(\mu{-}NC_8H_3F_6)(NC_6H_4F_3)$] (6b) 24

Figure S-19. ^{19}**F NMR (376 MHz) spectrum for paramagnetic** $[Fe_2(^{Ph}Dbf)_2(\mu{-}NC_8H_3F_6)(NC_6H_4F_3)]$ **(6b)**.......... 25

Figure S-20. ^{1}**H NMR (600 MHz) spectra for paramagnetic** $[Fe_2(^{Ph}Dbf)_2(\mu{-}NC_8H_3F_6)(NC_5H_5)]$ **(6a, *bottom*) and** $[Fe_2(^{Ph}Dbf)_2(\mu{-}NC_8H_3F_6)(NC_6H_4F_3)]$ **(6b, *top*)**.......... 26

Figure S-21. ^{19}**F NMR (376 and 564 (*top*) MHz) spectra for** $[Fe_2(^{Ph}Dbf)_2(\mu{-}NC_8H_3F_6)]$ **2a upon addition of 1 equivalent (*bottom*), 2 equivalent (*middle*), excess pyridine (*middle*), and for complex** $[Fe(^{Ph}Dbf)(NC_5H_5)_2]$ **5a upon addition of 0.5 equivalents of 3,5-bis(trifluoromethyl)phenylazide.** ... 27

Figure S-22. Integrated ^{19}**F NMR (564 MHz, benzene-*d*$_6$) spectrum for the reaction of** $[Fe_2(^{Ph}Dbf)_2(\mu{-}NC_8H_3F_6)]$ **2a with 2 equivalents of 4-trifluoromethylpyridine**.......... 28

Figure S-23. Stacked ^{19}**F NMR (564 MHz, benzene-*d*$_6$) spectra for the reaction of** $[Fe_2(^{Ph}Dbf)_2(\mu{-}NC_8H_3F_6)]$ **2a (*bottom*) with 1 (*middle*) and 2 (*top*) equivalents of 4-trifluoromethylpyridine.**.......... 29

Figure S-24. Frozen benzene EPR (80 K) spectrum of $[Fe_2(^{Ph}Dbf)_2(\mu{-}NC_8H_3F_6)]$ **(2a)**.......... 30

Figure S-25. Frozen benzene EPR (80 K) spectrum of $[Fe_2(^{Ph}Dbf)_2(\mu{-}NC_{10}H_{13})]$ **(2b)**.......... 31

Figure S-26. Frozen toluene EPR (80 K) spectrum of $[Fe_2(^{Ph}Dbf)_2(\mu{-}NHC_8H_3F_6)(NHC_8H_3F_6)]$ **(3)** 32

Figure S-27. Stacked frozen toluene EPR spectra at 80 K and 115 K of $[Fe_2(^{Ph}Dbf)_2(\mu{-}NHC_8H_3F_6)(NHC_8H_3F_6)]$ **(3)**.......... 33

Figure S-28. Frozen toluene EPR (80 K) spectrum of the reaction of $[Fe_2(^{Ph}Dbf)_2(\mu{-}NHC_8H_3F_6)(OC_{19}H_{15})]$ **(4)**.......... 34

Figure S-29. Frozen benzene EPR (80 K) spectrum of the generation of $Fe_2(^{Ph}Dbf)_2(\mu{-}NC_8H_3F_6)(NC_5H_5)]$ **(6a)**.......... 35

Figure S-30. Frozen toluene EPR (80 K) spectrum of the reaction of $[Fe_2(^{Ph}Dbf)_2(\mu{-}NC_8H_3F_6)]$ **(2a) with 1 equivalent of 2-hydroxy-2-adamantane.**.......... 36

Figure S-31. Frozen toluene EPR (80 K) spectrum of 9-azabicyclo[3.3.1]nonane-N-oxyl........... 37

Figure S-32. RT benzene EPR spectrum of the reaction of $[Fe_2(^{Ph}Dbf)_2(\mu{-}NC_8H_3F_6)]$ **(2a) with 1 equivalent 2,4,6-*tert*-butylphenol.**.......... 38

Table S-2. Experimental g_{eff} **values for Fe(III) complexes.**.......... 39

Figure S-33. Infrared Spectrum of liquid 3,5-bis(trifluoromethyl)phenyl azide........... 40

Figure S-34. Infrared Spectrum of solid $[Fe_2(^{Ph}Dbf)_2(\mu{-}NC_8H_3F_6)]$ **(2a).**.......... 41

Figure S-35. Infrared Spectrum of liquid 4-*tert*-butylphenyl azide........... 42

Figure S-36. Infrared Spectrum of solid $[Fe_2(^{Ph}Dbf)_2(\mu{-}NC_{10}H_{13})]$ **(2b).**.......... 43

Figure S-37. Infrared Spectrum of solid $[Fe_2(^{Ph}Dbf)_2(\mu{-}NHC_8H_3F_6)(NHC_8H_3F_6)]$ **(3).**.......... 44

Figure S-38. Infrared Spectrum of liquid $[Fe_2(^{Ph}Dbf)_2(\mu{-}NHC_8H_3F_6)(NHC_8H_3F_6)]$ **(3).**.......... 45

Figure S-39. Stacked infrared spectra of solid (red) and liquid (black) samples of $[Fe_2(^{Ph}Dbf)_2(\mu{-}NHC_8H_3F_6)(NHC_8H_3F_6)]$ **(3)**.......... 46

Figure S-40. Infrared Spectrum of solid $[Fe_2(^{Ph}Dbf)_2(\mu{-}NHC_8H_3F_6)(OC_{19}H_{15})]$ **(4).**.......... 47

Figure S-41. Infrared Spectrum of solid $[Fe_2(^{Ph}Dbf)_2(\mu{-}NC_8H_3F_6)(NC_5H_5)]$ **(6a).**.......... 48

Figure S-42. Infrared Spectrum of solid $[Fe_2(^{Ph}Dbf)_2(\mu{-}NC_8H_3F_6)(NC_6H_4F_3)]$ **(6b).**.......... 49

Table S-3. Reactions of 1 and 5a with aryl azides. 50
Figure S-43. ^{1}H NMR (600 Hz) spectrum of the reaction of [Fe_2(^{Ph}Dbf)$_2$] (1) with 4-nitrophenyl azide 51
Figure S-44. ^{1}H NMR (600 Hz) spectrum of the reaction of [Fe_2(^{Ph}Dbf)$_2$] (1) with 2,4,6-trimethylphenyl azide 52
Figure S-45. ^{1}H NMR (600 Hz) spectrum of the reaction of [Fe(^{Ph}Dbf)(NC_5H_5)$_2$] (5a) with 4-*tert*-butylphenylazide 53
Figure S-46. ^{1}H NMR (600 Hz) spectrum of the reaction of [Fe(^{Ph}Dbf)(NC_5H_5)$_2$] (5a) with 4-nitrophenylazide 54
Figure S-47. ^{1}H NMR (600 Hz) spectrum of the reaction of [Fe(^{Ph}Dbf)(NC_5H_5)$_2$] (5a) with 2,4,6-trimethylphenyl azide 55
Figure S-48. ^{1}H NMR (600 Hz) spectra of the reaction of 1 and 4-*tert*-butylphenylazide (*bottom*), [Fe_2(^{Ph}Dbf)$_2$(μ−$NC_8H_3F_6$)] (2b) and 1 equivalent of pyridine (*middle*) and [Fe(^{Ph}Dbf)(NC_5H_5)$_2$] (5a) with 4-*tert*-butylphenyl azide (*top*). 56
Figure S-49. ^{1}H NMR (600 Hz) spectra of the reaction of 1 and 2,4,6-trimethylphenyl azide (*bottom,* 376 MHz), [Fe_2(^{Ph}Dbf)$_2$(μ−$NC_8H_3F_6$)] (2b) and 1 equivalent of pyridine (*middle,* 564 MHz) and [Fe(^{Ph}Dbf)(NC_5H_5)$_2$] (5a) with 4-*tert*-butylphenyl azide (*top,* 564 MHz). 57
Figure S-50. ^{1}H NMR (600 MHz) spectrum of the reaction of [Fe_2(^{Ph}Dbf)$_2$] (1) with 10 equivalents 2,6-diisopropylphenyl azide at room temperature. 58
Figure S-51. ^{1}H NMR (600 MHz) spectrum of the reaction of [Fe_2(^{Ph}Dbf)$_2$] (1) with 1 equivalent of 2,6-diisopropylphenylazide after 18 hours at 80 °C. 59
Figure S-52. ^{1}H NMR (600 MHz) spectrum of the initial reaction of [Fe_2(^{Ph}Dbf)$_2$] (1) with 10 equivalents 2,6-diisopropylphenyl azide after 24 hours at 80 °C. 60
Figure S-53. ^{1}H NMR (600 MHz) spectrum of the organic product 2-isopropyl-6-(prop-1-en-2-yl)aniline resulting from the reaction of [Fe_2(^{Ph}Dbf)$_2$] (1) with 10 equivalents 2,6-diisopropylphenyl azide after 24 hours at 80 °C. 61
Figure S-54. ^{1}H (600 MHz) and ^{19}F (564 MHz, *inlay left*) NMR spectra of the organic product, 1-(3,5-bis(trifluoromethyl)phenyl-2-phneylaziridine, of the reaction of [Fe_2(^{Ph}Dbf)$_2$] (1) with styrene. 62
Figure S-55. ^{19}F NMR (564 MHz) spectrum for paramagnetic [Fe_2(^{Ph}Dbf)$_2$(μ−$NC_8H_3F_6$)] (2a) reaction with 2-hydroxy-2-azaadaamantane. 63
Figure S-56. ^{19}F NMR (564 MHz) spectrum for paramagnetic [Fe_2(^{Ph}Dbf)$_2$(μ−$NC_8H_3F_6$)] (2a) reaction with 9-azabicyclo[3.3.1]nonane-N-oxyl at room temperature. 64
Figure S-57. ^{19}F NMR (376 MHz) spectrum for paramagnetic [Fe_2(^{Ph}Dbf)$_2$(μ−$NC_8H_3F_6$)] (2a) reaction with 2,4,6-tri-*tert*-butylphenol 65
Figure S-58. ^{19}F NMR (376 MHz) spectra for paramagnetic [Fe_2(^{Ph}Dbf)$_2$(μ−$NC_8H_3F_6$)] (2a) (bottom) reaction with 1,4-cyclohexadiene 66
Figure S-59. ^{19}F NMR (565 MHz) spectrum for paramagnetic [Fe_2(^{Ph}Dbf)$_2$(μ−$NC_8H_3F_6$)] (2a) reaction with *9H*-fluorene 67

Figure S-60. ^{19}**F NMR (565 MHz) spectrum for paramagnetic [Fe_2(^{Ph}Dbf)$_2$(μ−$NC_8H_3F_6$)] (2a) reaction with triphenylmethane** **68**

Figure S-61. ^{19}**F NMR (376 MHz) spectrum (*unlocked in toluene*) for paramagnetic [Fe_2(^{Ph}Dbf)$_2$(μ−$NC_8H_3F_6$)] (2a) reaction with toluene at room temperature.** **69**

Figure S-62. Stacked ^{19}**F NMR (376 MHz) spectra (*unlocked in toluene*) for paramagnetic [Fe_2(^{Ph}Dbf)$_2$(μ−$NC_8H_3F_6$)] (2a) reaction with toluene at 80 °C** **70**

Figure S-63. Stacked ^{19}**F NMR (376 MHz) spectra (*unlocked in toluene*) for paramagnetic [Fe_2(^{Ph}Dbf)$_2$(μ−$NC_8H_3F_6$)] (2a) reaction with toluene** **71**

Figure S-64. ^{1}**H (600 MHz) and** ^{19}**F (564 MHz, *inlay*) NMR spectra of the organic product of the reaction of Fe_2(^{Ph}Dbf)$_2$(μ−$NC_8H_3F_6$)(NC_5H_5)] (6a) reaction with 1,4-cyclohexadiene** **72**

Figure S-65. ^{19}**F NMR (376 MHz (*bottom*) and 564 MHz; C_6D_6) spectra of the reaction of [Fe_2(^{Ph}Dbf)$_2$(μ−$NC_8H_3F_6$)(NC_5H_5)] (6a) reaction with 1,4-cyclohexadiene at room temperature for 1h, 60 °C overnight, and 80 °C overnight** **73**

Figure S-66. ^{19}**F NMR (376 MHz (*bottom*) and 564 MHz; C_6D_6) spectra of the reaction of [Fe_2(^{Ph}Dbf)$_2$(μ−$NC_8H_3F_6$)($NC_6H_5F_3$)] (6b) reaction with 1,4-cyclohexadiene** **74**

Figure S-67. ^{19}**F NMR (564 MHz; C_6D_6) spectrum of the reaction of [Fe_2(^{Ph}Dbf)$_2$(μ−$NC_8H_3F_6$)($NC_6H_4F_3$)] (6b) reaction with 1,4-cyclohexadiene** **75**

Figure S-68. ^{19}**F NMR (564 MHz; C_6D_6) spectrum of the reaction of [Fe_2(^{Ph}Dbf)$_2$(μ−$NC_8H_3F_6$)($NC_6H_4F_3$)] (6b) reaction with 1,4-cyclohexadiene at 90 °C after 36 hours.** **76**

Figure S-69. ^{19}**F NMR (564 MHz; C_6D_6) spectrum of the reaction of [Fe_2(^{Ph}Dbf)$_2$(μ−$NHC_8H_3F_6$)($NHC_6H_5F_3$)] (3) reaction with 0.9 equivalents of 4-trifluoromethylpyridine.** **77**

Figure S-70. Stacked ^{19}**F NMR (564 MHz; C_6D_6) spectra of the reaction of [Fe_2(^{Ph}Dbf)$_2$(μ−$NC_8H_3F_6$)($NC_6H_4F_3$)] (6b) reaction with 1,4-cyclohexadiene at 90 °C after 36 hours (*bottom*), complex 3 with 0.9 equivalents of *p*-CF3Py (*middle*), and monomer 5b with 1,4-cyclohexadiene at 90 °C after 24 hours (*top*).** **78**

Figure S-71. ^{19}**F NMR (376 MHz (*bottom*) and 564 MHz; C_6D_6) spectra of the reaction of [Fe_2(^{Ph}Dbf)$_2$(μ−$NC_8H_3F_6$)($NC_6H_4F_3$)] (6b) reaction with 1,4-cyclohexadiene at room temperature for 1h, 60 °C overnight, and 80 °C overnight** **79**

Figure S-72. Stacked ^{19}**F NMR (376 MHz) spectra (*unlocked in toluene*) for paramagnetic [Fe_2(^{Ph}Dbf)$_2$(μ−$NC_8H_3F_6$)(NC_5H_5)] (6a) reaction with toluene** **80**

Figure S-73. Stacked ^{19}**F NMR (376 MHz) spectra (*unlocked in toluene*) for paramagnetic [Fe_2(^{Ph}Dbf)$_2$(μ−$NC_8H_3F_6$)(NC_5H_5)] (6a) reaction with toluene** **81**

Figure S-74. Stacked ^{19}**F NMR (564 MHz, C_6D_6) spectra of the reaction of paramagnetic [Fe(^{Ph}Dbf) (μ-$NHC_8H_3F_6$)($NHC_8H_3F_6$)] (3) reaction with 9-azabicyclo[3.3.1]nonane-N-oxyl (*top*).** **82**

Figure S-75. Stacked ^{19}**F NMR (564 MHz, C_6D_6) spectra of the reaction of paramagnetic [Fe(^{Ph}Dbf) (μ-$NHC_8H_3F_6$)($NHC_8H_3F_6$)] (3, *bottom*) reaction with Gombergs's dimer (*top*).** **83**

Figure S-76. Stacked ^{19}**F NMR (564 MHz, C_6D_6) spectra of complex 4 (*bottom*) and all proposed asymmetric bridging amide complexes from reactions of complex 3 with organic (*middle)* and oxyl**

(*middle*) radicals, as well as species generated in the reaction of 6b with 1,4-cyclohexadiene at 90 °C (*top*)....84

X-Ray Diffraction Techniques....85

Table S-4. X-ray diffraction experimental details....87

Figure S-77. Solid-state molecular structure for [Fe_2(^{Ph}Dbf)$_2$(μ−$NC_8H_3F_6$)] (2a)....92

Figure S-78. Solid-state molecular structure for [Fe_2(^{Ph}Dbf)$_2$(μ−$NC_{10}H_{13}$)] (2b)....93

Figure S-79. Truncated solid-state molecular structure for [Fe_2(^{Ph}Dbf)$_2$(μ-$NHC_8H_3F_6$)($NHC_8H_3F_6$)] (3)....94

Figure S-80. Solid-state molecular structure for [Fe_2(^{Ph}Dbf)$_2$(μ-$NHC_8H_3F_6$)($OC_{19}H_{15}$)] (4)....95

Figure S-81. Solid-state molecular structure for [Fe_2(^{Ph}Dbf)$_2$(μ-$NHC_8H_3F_6$)(NC_5H_5)] (6a)....96

Figure S-82. Space fill model for [Fe_2(^{Ph}Dbf)$_2$(μ−$NC_8H_3F_6$)] (2a)....97

Figure S-83. Space fill model for [Fe_2(^{Ph}Dbf)$_2$(μ−$NC_{10}H_{13}$)] (2b)....98

Figure S-84. Space fill model for [Fe_2(^{Ph}Dbf)$_2$(μ−$NHC_8H_3F_6$)((μ−$NHC_8H_3F_6$)] (3)....99

Figure S-85. Space fill model for [Fe_2(^{Ph}Dbf)$_2$(μ−$NHC_8H_3F_6$)($OCPh_3$)] (4)....100

Figure S-86. Space fill model for [Fe_2(^{Ph}Dbf)$_2$(μ−$NHC_8H_3F_6$)(NC_5H_5)] (6a)....101

Figure S-87. Polyhedral model for [Fe_2(^{Ph}Dbf)$_2$(μ−$NC_8H_3F_6$)] (2a)....102

Table S-5. Selected bond distances and angles for complexes 2a, 2b, and 6a....103

Table S-6. Selected bond distances and angles for complexes 3 and 4....103

Table S-7. Bond metrics for 3 and 4 and average bond lengths and angles calculated from reported structures in the CSD for related bridging aryl imido[8-10], bridging aryl bis(imido)[11-15], terminal aniline[8, 16-23] and bridging[14, 24, 25] and terminal[26-31] aryl amide complexes. All values were calculated in Excel from data obtained from the .cif files for each complex. Average bond lengths were calculated using the average function in excel and rounded to the least number of significant figures for each sample pool. In Excel, error for each measurement was recorded and used to calculate using the propagation of error formula: $\sigma x\mathrm{x} = \ i = \sigma nni2N$....104

Figure S-88. Gas Chromatography Mass Spectroscopy Trace and spectra for the reaction of 2a in Toluene heated at 80 °C....106

Figure S-89. Gas Chromatography Mass Spectroscopy Trace and spectra for the reaction of 6a in Toluene heated at 100 °C....107

Figure S-90. Gas Chromatography Mass Spectroscopy Trace and spectra for the reaction of 2a in 2,6-diisopropylphenyl azide heated at 80 °C....108

Table S-9. Coupling constants for 2a, 6a, 3, and 4....109

References....110

Zero-field ^{57}Fe Mössbauer of relevant species

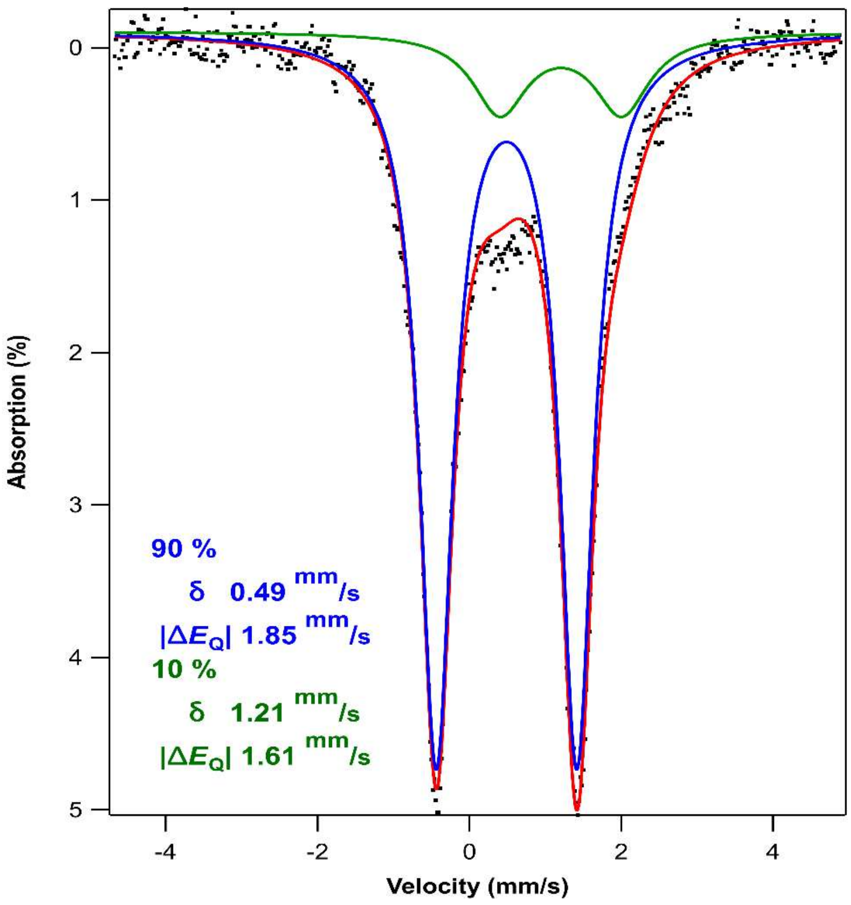

Figure S-1. Zero-field ^{57}Fe Mössbauer of [$Fe_2(^{Ph}Dbf)_2(\mu{-}NC_8H_3F_6)$] (**2**) collected at 90 K. Isomer shift and quadrupole splitting are referenced to Fe foil at room temperature. Parameters calculated via single-point DFT: 100 % δ = 0.51 mm/s, ΔE_Q = 1.427 mm/s.

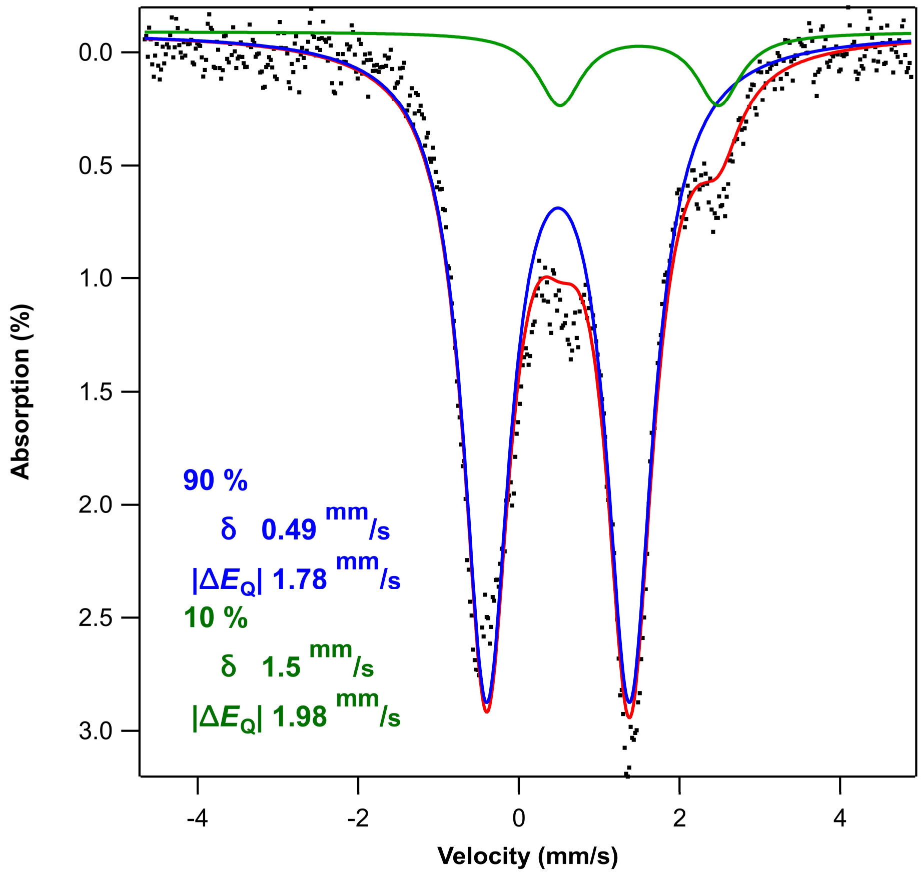

Figure S-2. Zero-field [57]Fe Mössbauer of [$Fe_2(^{Ph}Dbf)_2(\mu\text{-}NHC_8H_3F_6)(NHC_8H_3F_6)$] (**3**) collected at 90 K. Isomer shift and quadrupole splitting are referenced to Fe foil at room temperature. Parameters calculated via single-point DFT: 50 % δ = 0.44 mm/s, ΔE_Q = 1.556 mm/s; 50 % δ = 0.43 mm/s, ΔE_Q = -1.88 mm/s

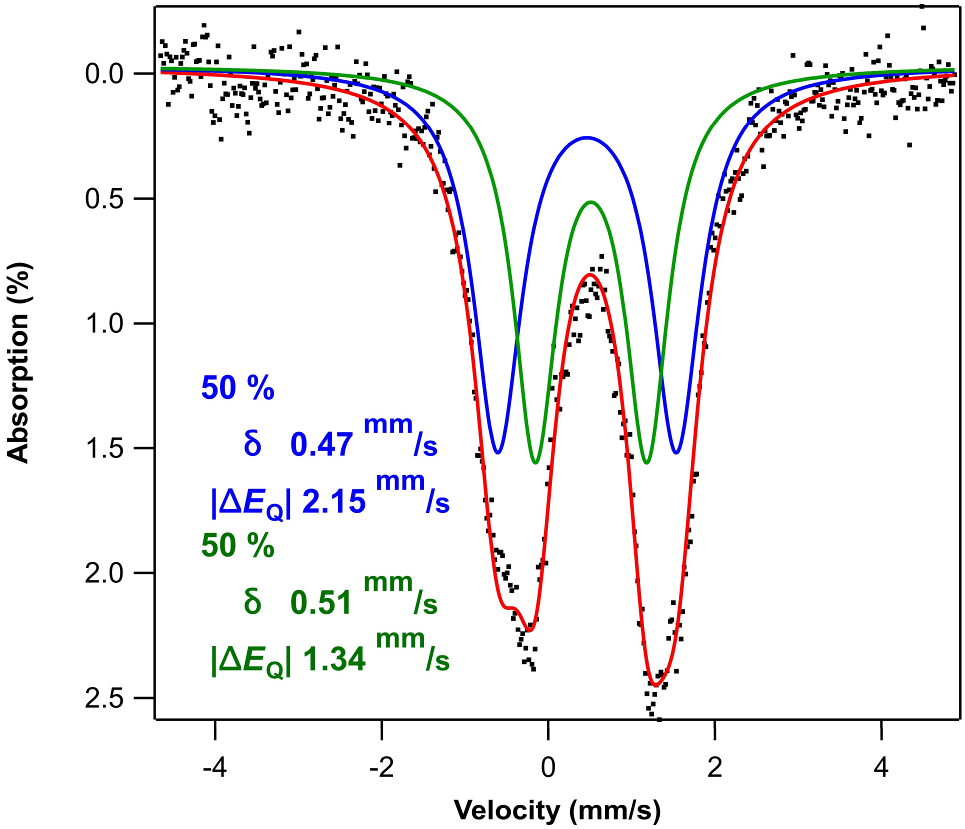

Figure S-3. Zero-field ^{57}Fe Mössbauer of $[Fe_2(^{Ph}Dbf)_2(\mu\text{-}NHC_8H_3F_6)(OC_{19}H_{15})$ (**4**) collected at 90 K. Isomer shift and quadrupole splitting are referenced to Fe foil at room temperature. Parameters calculated via single-point DFT: 50 % δ = 0.44 mm/s, ΔE_Q = –1.556 mm/s; 50 % δ = 0.43 mm/s, ΔE_Q = –1.556 mm/s.

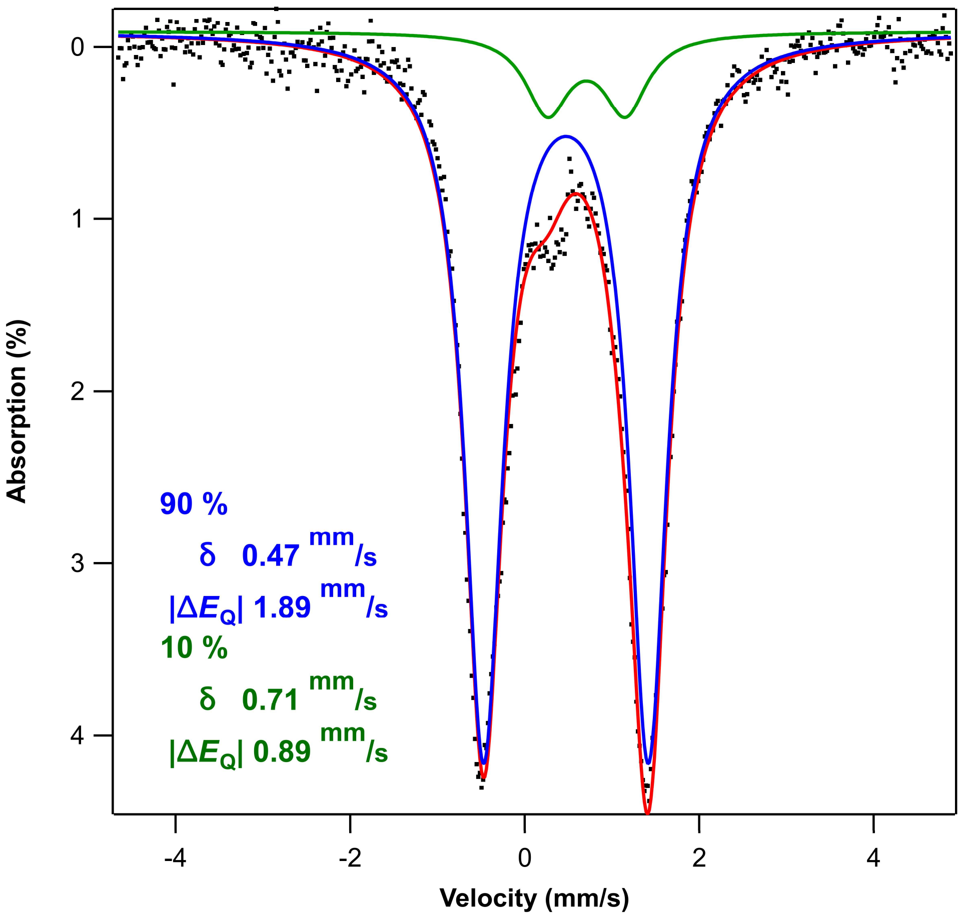

Figure S-4. Zero-field ^{57}Fe Mössbauer of $[Fe_2(^{Ph}Dbf)_2(\mu\text{-}NHC_8H_3F_6)(NC_5H_5)]$ (**6a**) collected at 90 K. Isomer shift and quadrupole splitting are referenced to Fe foil at room temperature.

Table S-1. Calculated and experimental ^{57}Fe Mossbauer isomer shifts and $|\Delta E_Q|$ values.

Complex	Calculated		Experimental	
	Isomer Shift	ΔE_Q	Isomer Shift	$|\Delta E_Q|$
**2a**	0.51	1.427	0.49	1.85
**3**	0.44	1.556	0.49	1.78
	0.43	-1.88	–	–
**4**	0.44	-1.556	0.51	1.34
	0.43	-1.556	0.47	2.15
**6a**	-	-	0.47	1.89

Magnetization Data

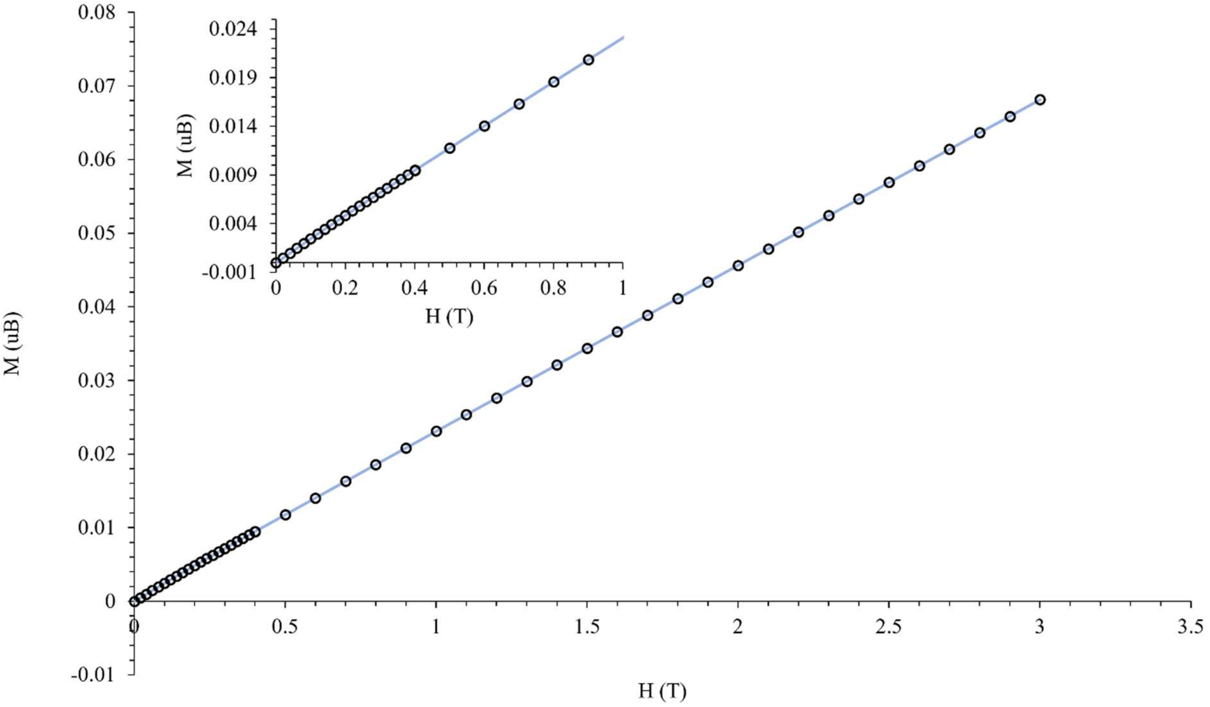

Figure S-5. Magnetization data for $[Fe_2(^{Ph}Dbf)_2(\mu{-}NC_8H_3F_6)]$ (**2a**). Magnetization versus field collected at 100 K – linear plot reflects the sample is free from ferromagnetic impurities; (inset) M (μB) vs. *H* at low fields.

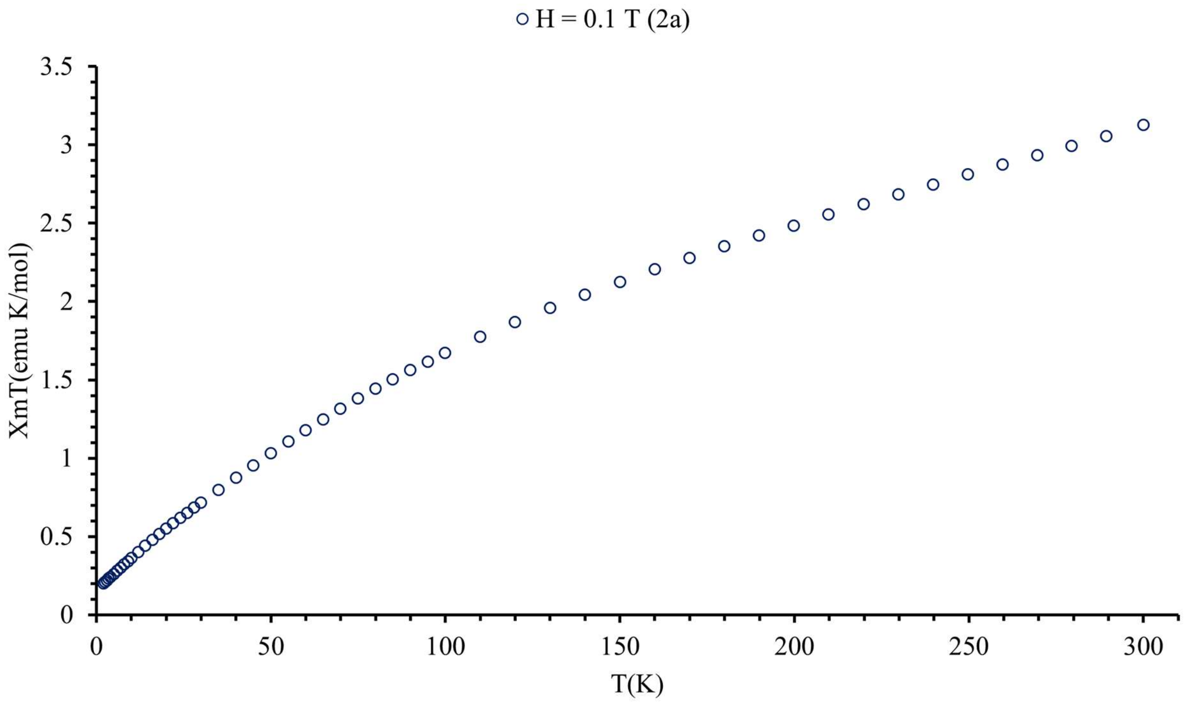

Figure S-6. Magnetization data for $[Fe_2(^{Ph}Dbf)_2(\mu{-}NC_8H_3F_6)]$ (**2a**). Variable temperature susceptibility data collected at 0.1 T, with $\chi_M T$ = 3.06 cm^3K/mol at 290 K.

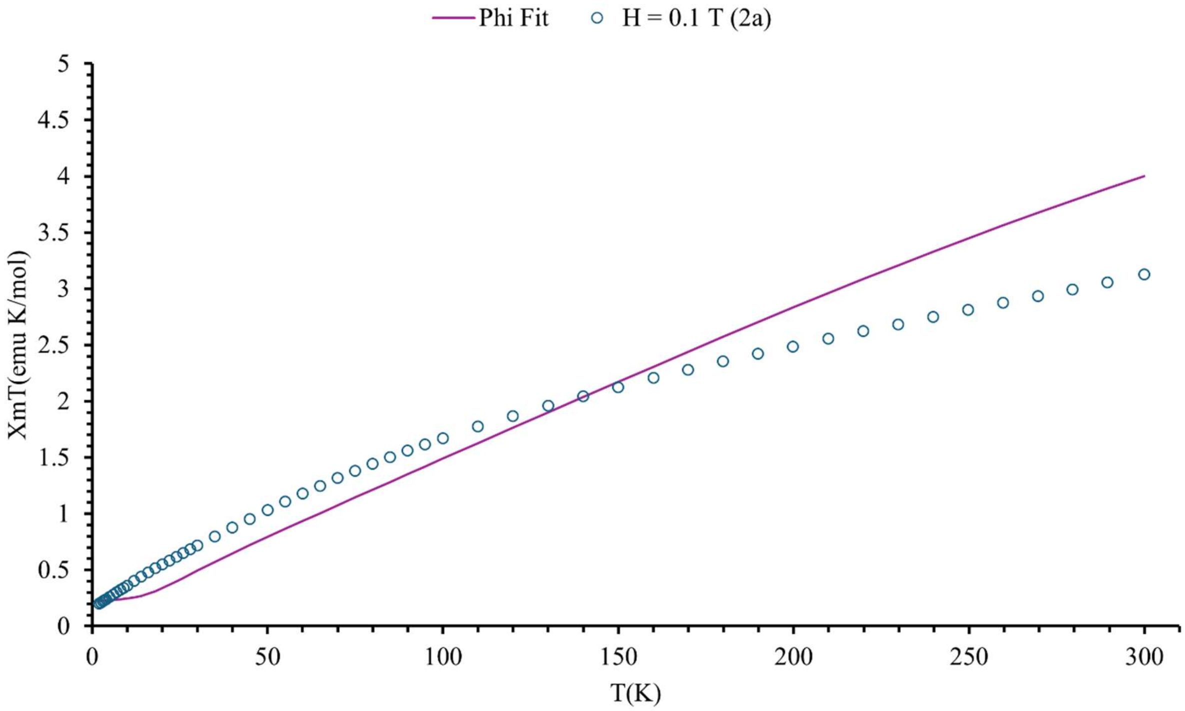

Figure S-7. Magnetization data for $[Fe_2(^{Ph}Dbf)_2(\mu-NC_8H_3F_6)]$ (**2a**) with magnetization fit. Variable temperature susceptibility data collected at 1.0 T, with $\chi_M T$ = 2.87 cm^3K/mol at 290 K. Magnetization fit parameters obtained with PHI[1]: g = 1.90; J = -25 cm^{-1}. This was the best fit obtained with reasonable g values; however, it does not account for the curvature of the data at high temperatures. Currently, we are unable to explain this feature.

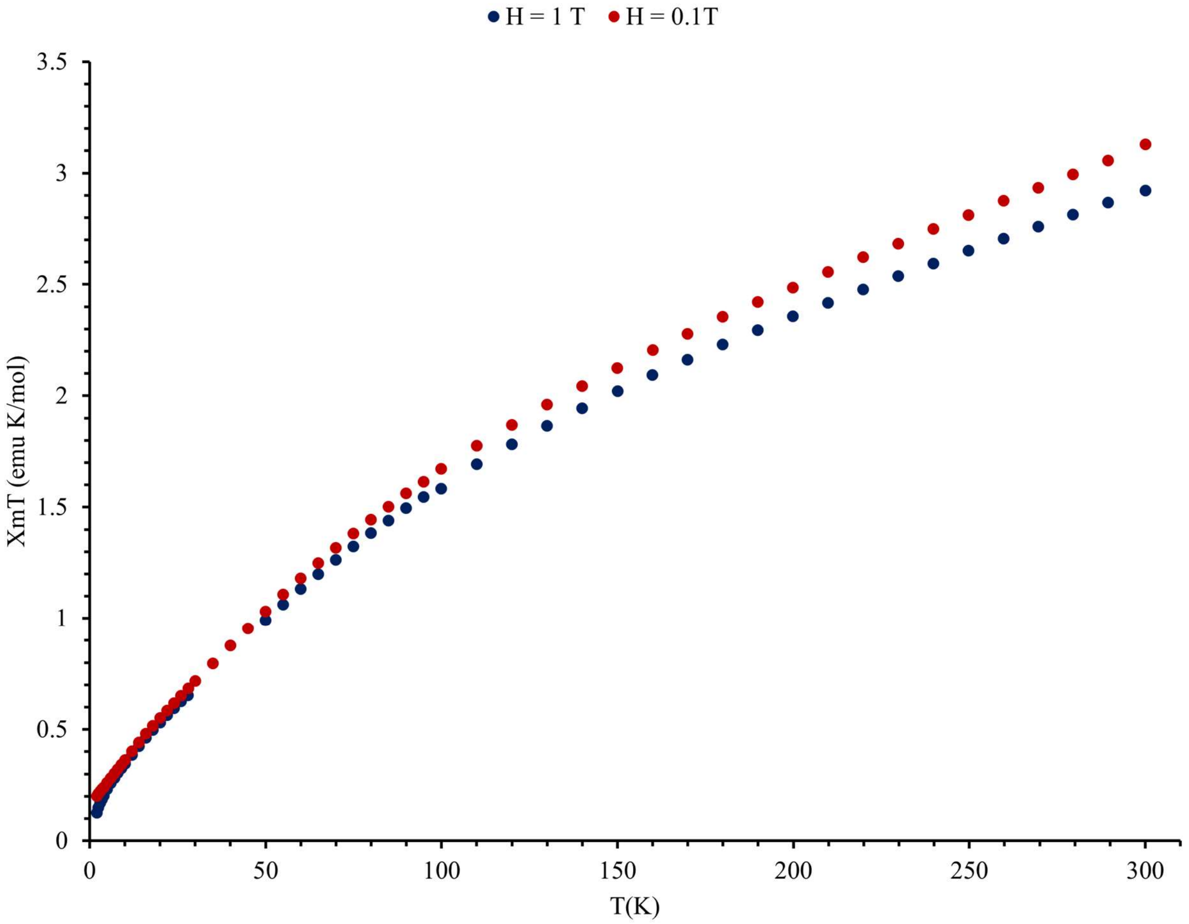

Figure S-8. Magnetization data for [Fe_2(^{Ph}Dbf)$_2$(μ−$NC_8H_3F_6$)] (**2a**) at 0.1 and 1.0 T. Variable temperature susceptibility data collected at 0.1 T and 1.0 T, with $\chi_M T$ = 3.06 cm^3K/mol and 2.87 cm^3K/mol at 290 K.

Nuclear Magnetic Resonance (NMR) Spectra

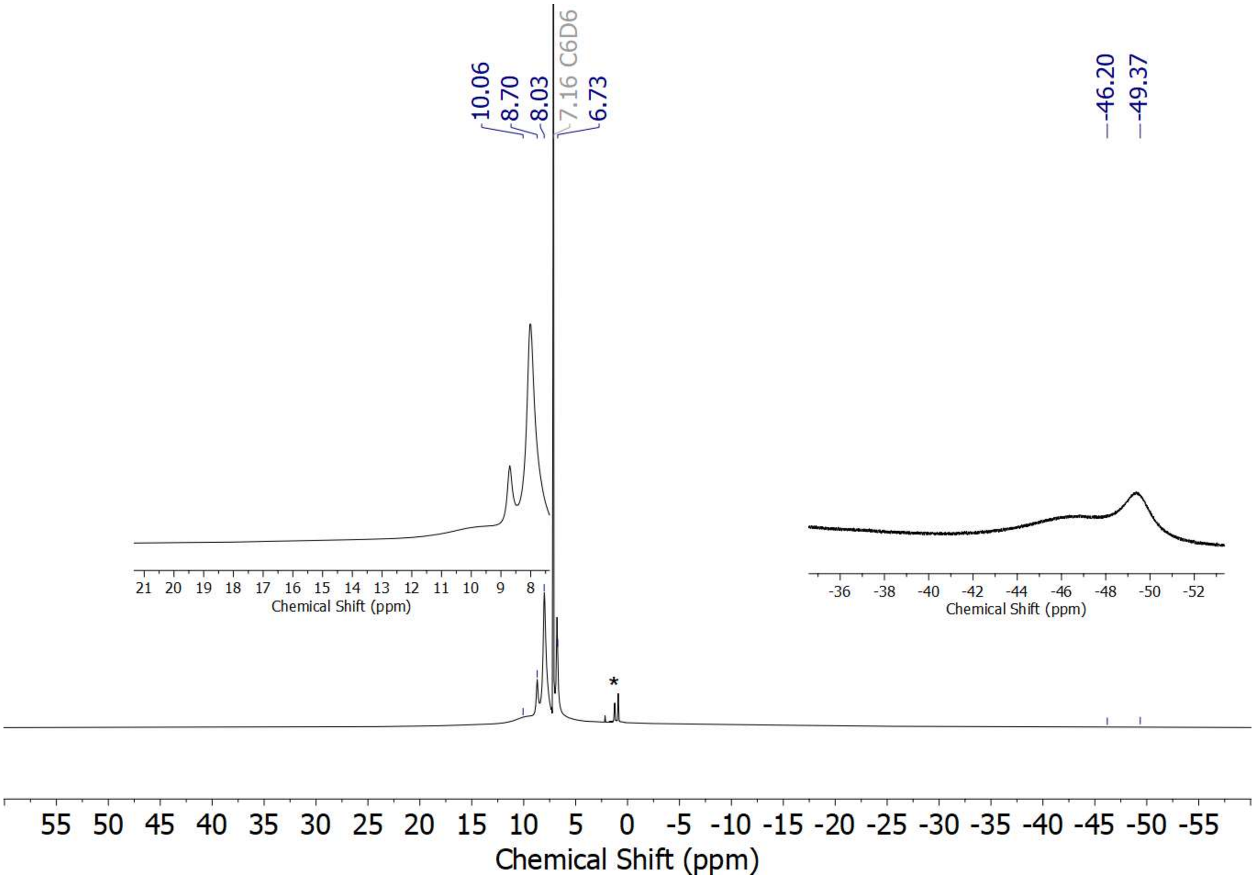

Figure S-9. ^{1}H NMR (600 MHz) spectrum for paramagnetic $[Fe_2(^{Ph}Dbf)_2(\mu{-}NC_8H_3F_6)]$ (**2a**) with an inset of paramagnetic peaks. *indicates minor organic impurities remaining from starting material wash (ligand/hexanes).

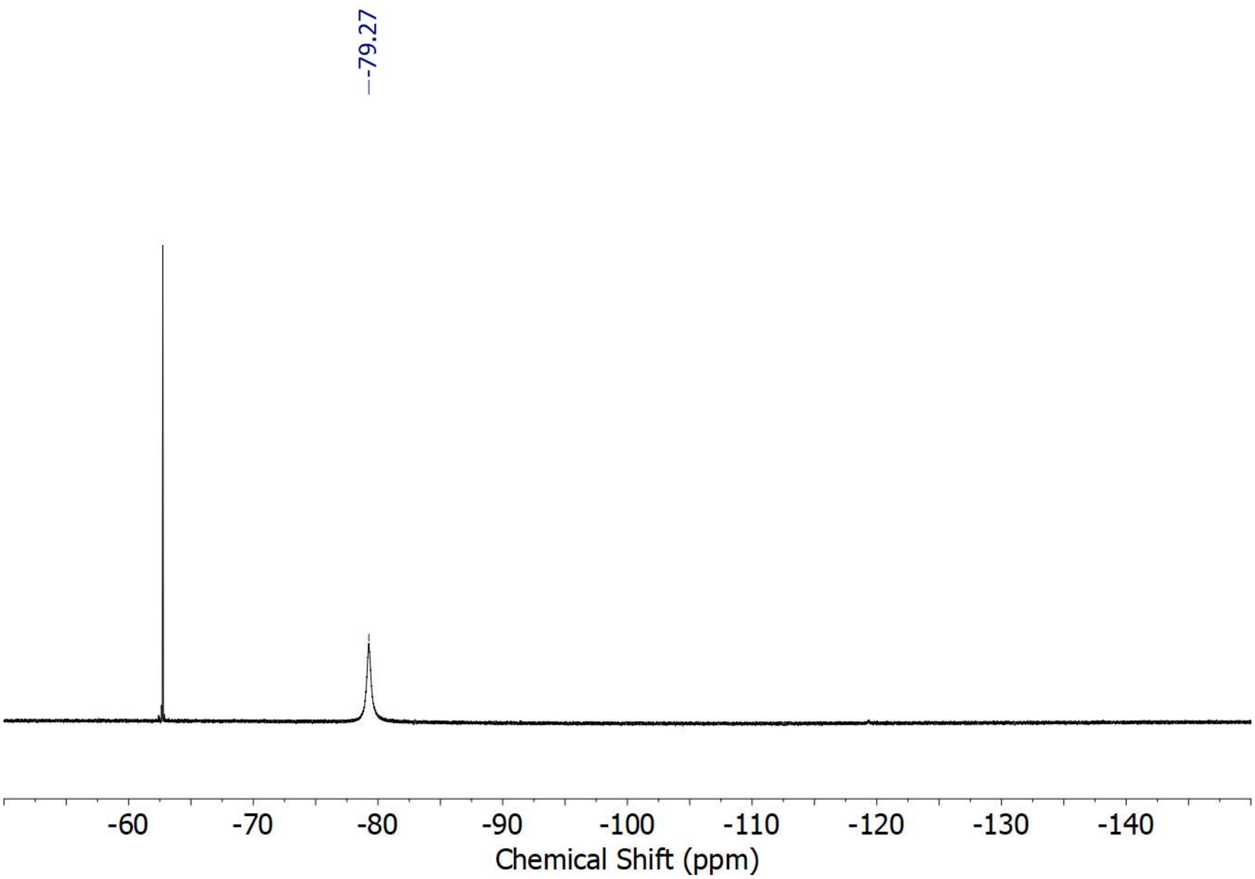

Figure S-10. ^{19}F NMR (376 MHz) spectrum for paramagnetic $[Fe_2(^{Ph}Dbf)_2(\mu{-}NC_8H_3F_6)]$ (**2a**) referenced to trifluorotoluene (-62.74 ppm).

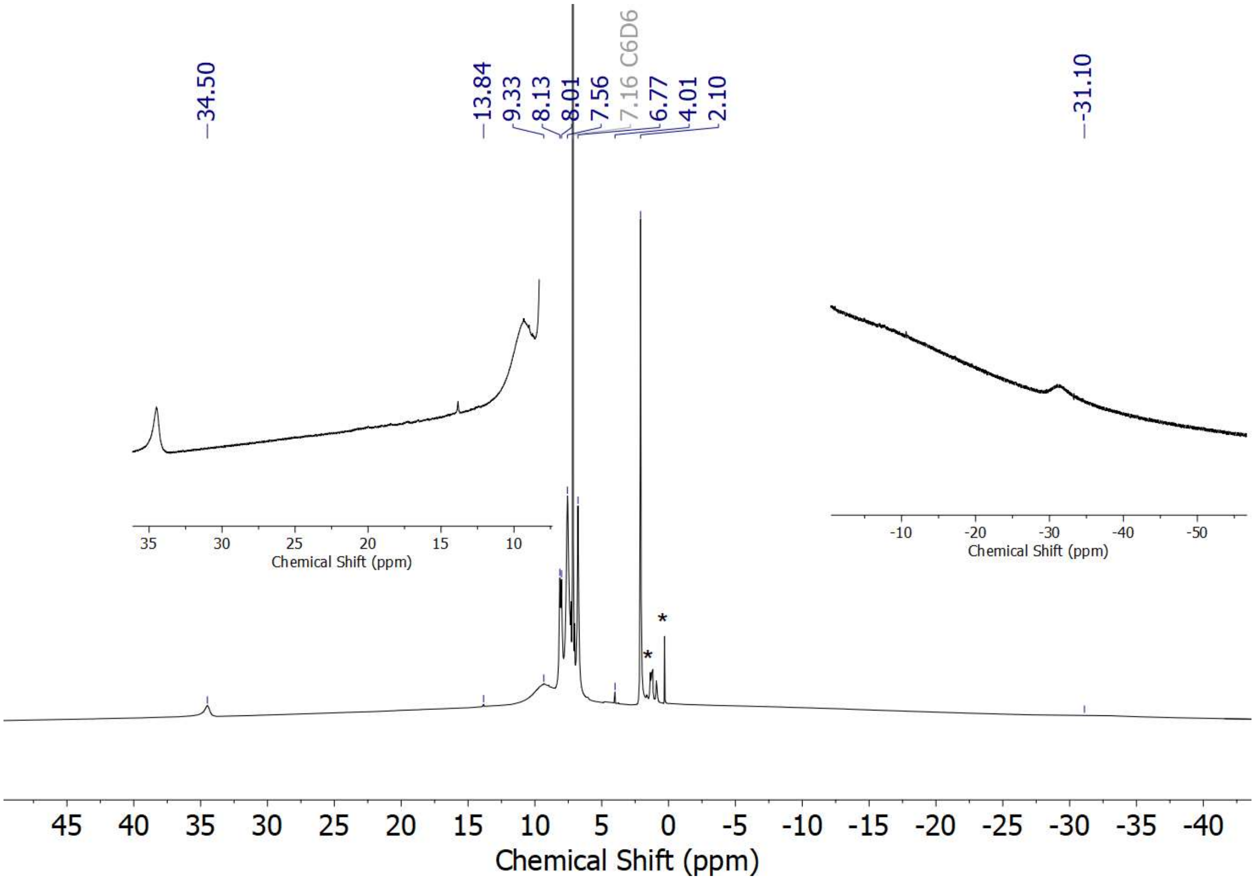

Figure S-11. ^{1}H NMR (600 MHz) spectrum for paramagnetic $[Fe_2(^{Ph}Dbf)_2(\mu{-}NC_8H_{13})]$ (**2b**). *indicates minor organic impurities from hexanes and grease.

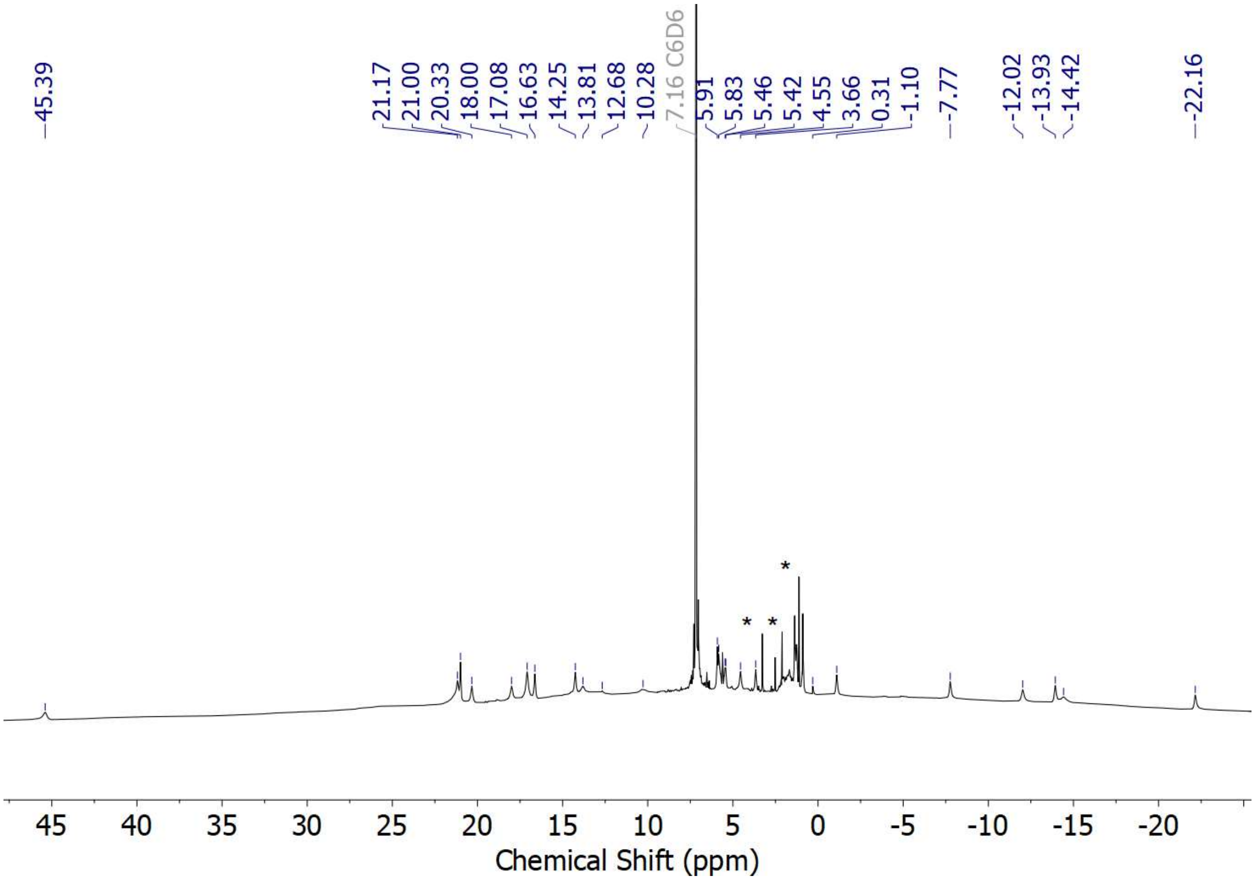

Figure S-12. ^{1}H NMR (600 MHz) spectrum for paramagnetic $[Fe_2(^{Ph}Dbf)_2(\mu{-}NHC_8H_3F_6)(NHC_8H_3F_6)]$ (**3**). * indicates impurity from hexanes, ligand, and 1,4-cyclohexadiene.

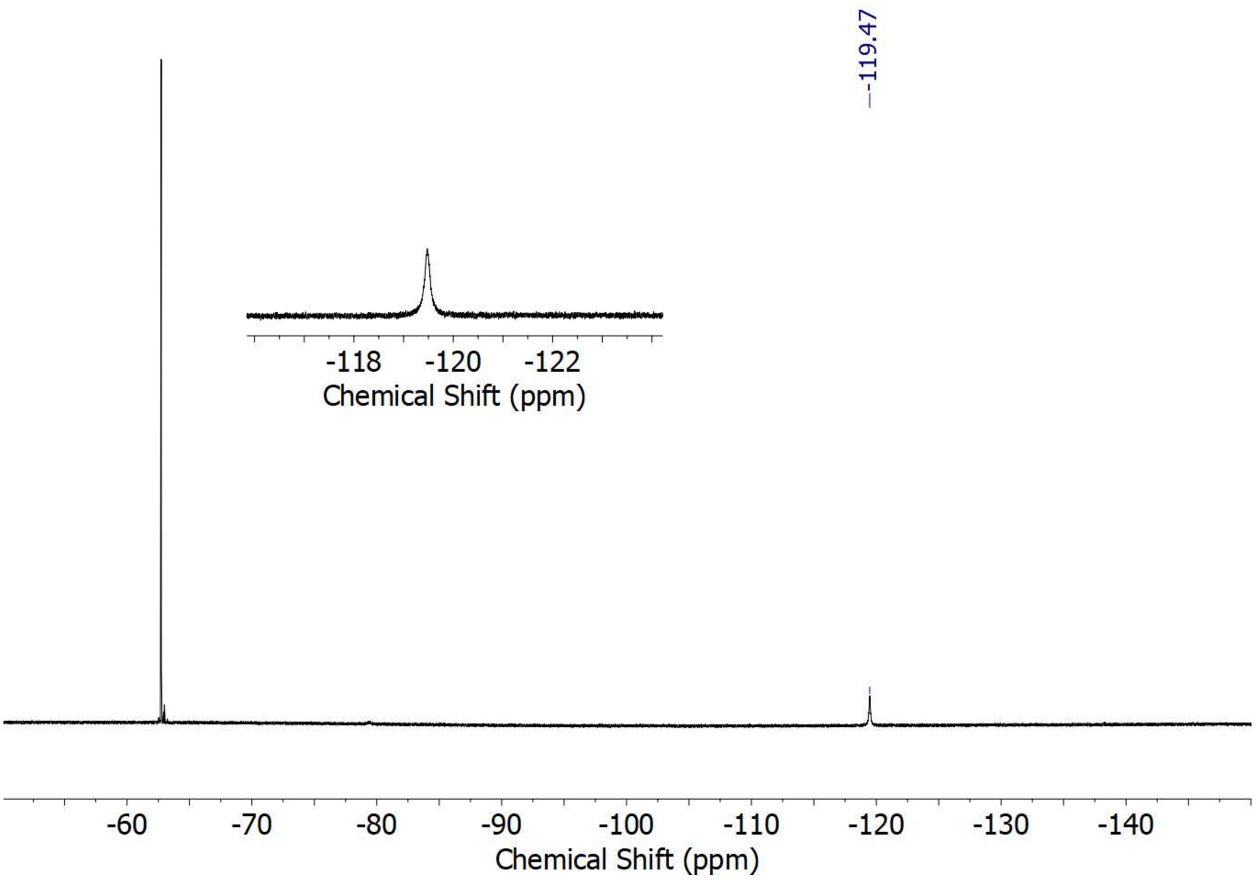

Figure S-13. ^{19}F NMR (376 MHz) spectrum for paramagnetic $[Fe_2(^{Ph}Dbf)_2(\mu{-}NHC_8H_3F_6)(NHC_8H_3F_6)]$ (**3**) referenced to trifluorotoluene (-62.74 ppm).

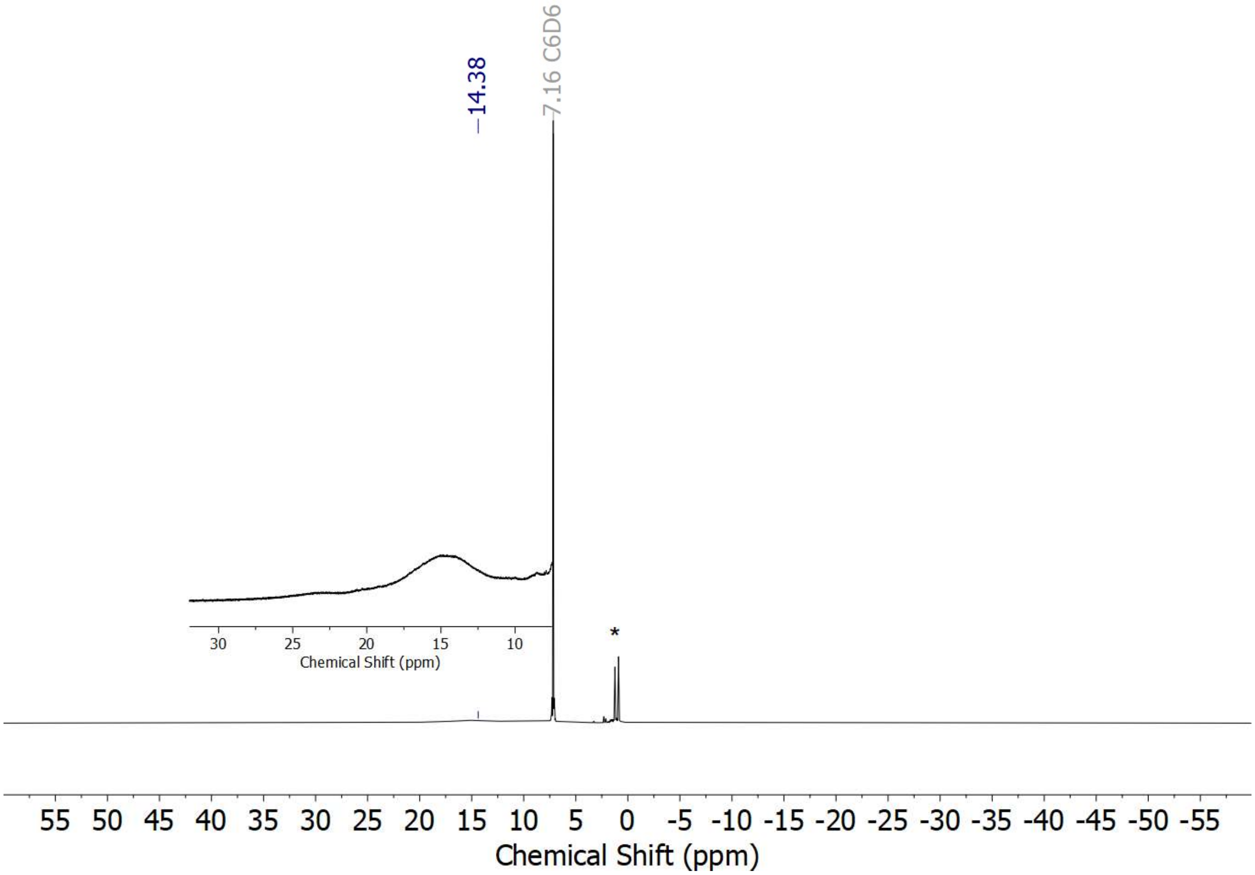

Figure S-14. ^{1}H NMR (600 MHz) spectrum for paramagnetic $[Fe_2(^{Ph}Dbf)_2(\mu{-}NHC_8H_3F_6)(OC_{19}H_{15})]$ (**4**). *indicates minor organic impurities remaining from starting material wash (ligand/hexanes).

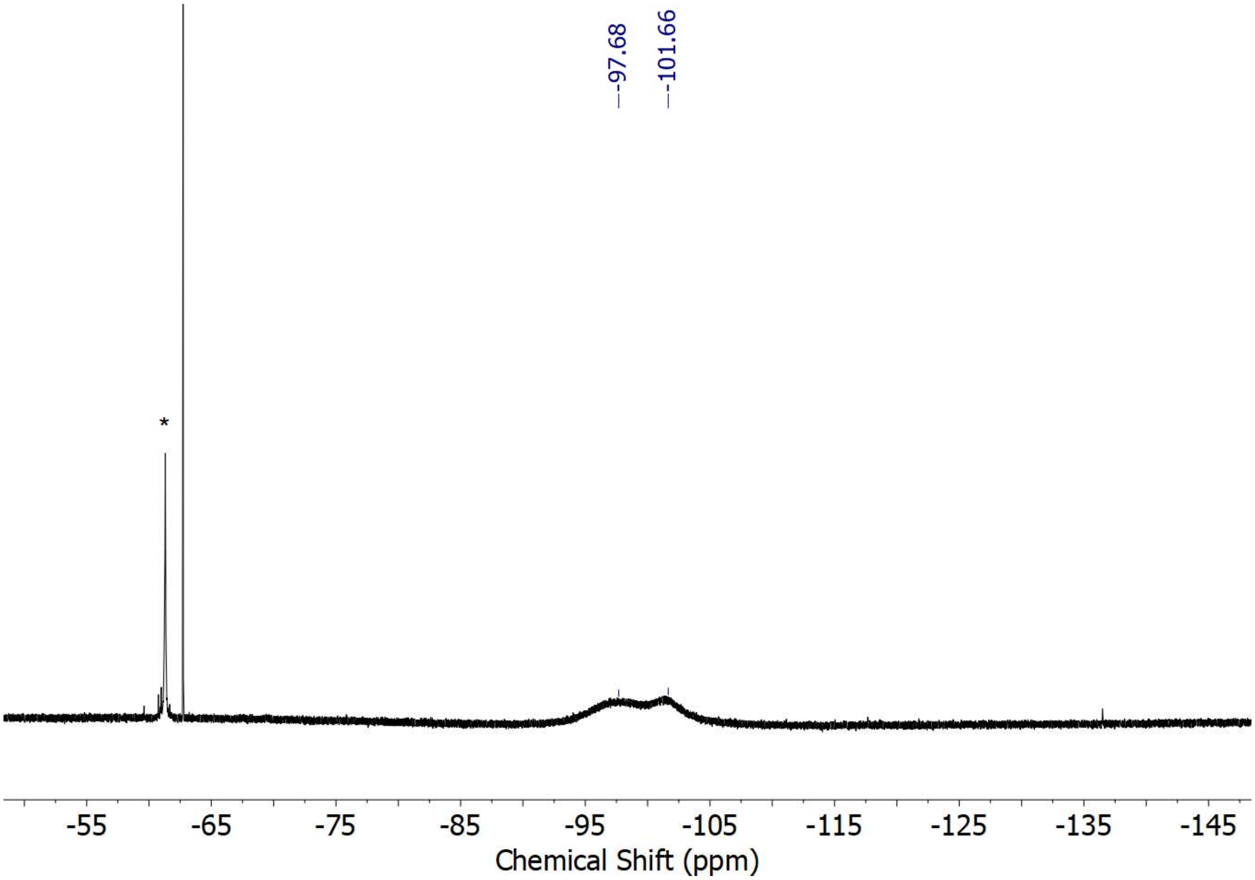

Figure S-15. ^{19}F NMR (376 MHz) spectrum for paramagnetic $[Fe_2(^{Ph}Dbf)_2(\mu{-}NHC_8H_3F_6)(OC_{19}H_{15})]$ (**4**) referenced to trifluorotoluene (-62.74 ppm). * indicates organic impurity (aniline).

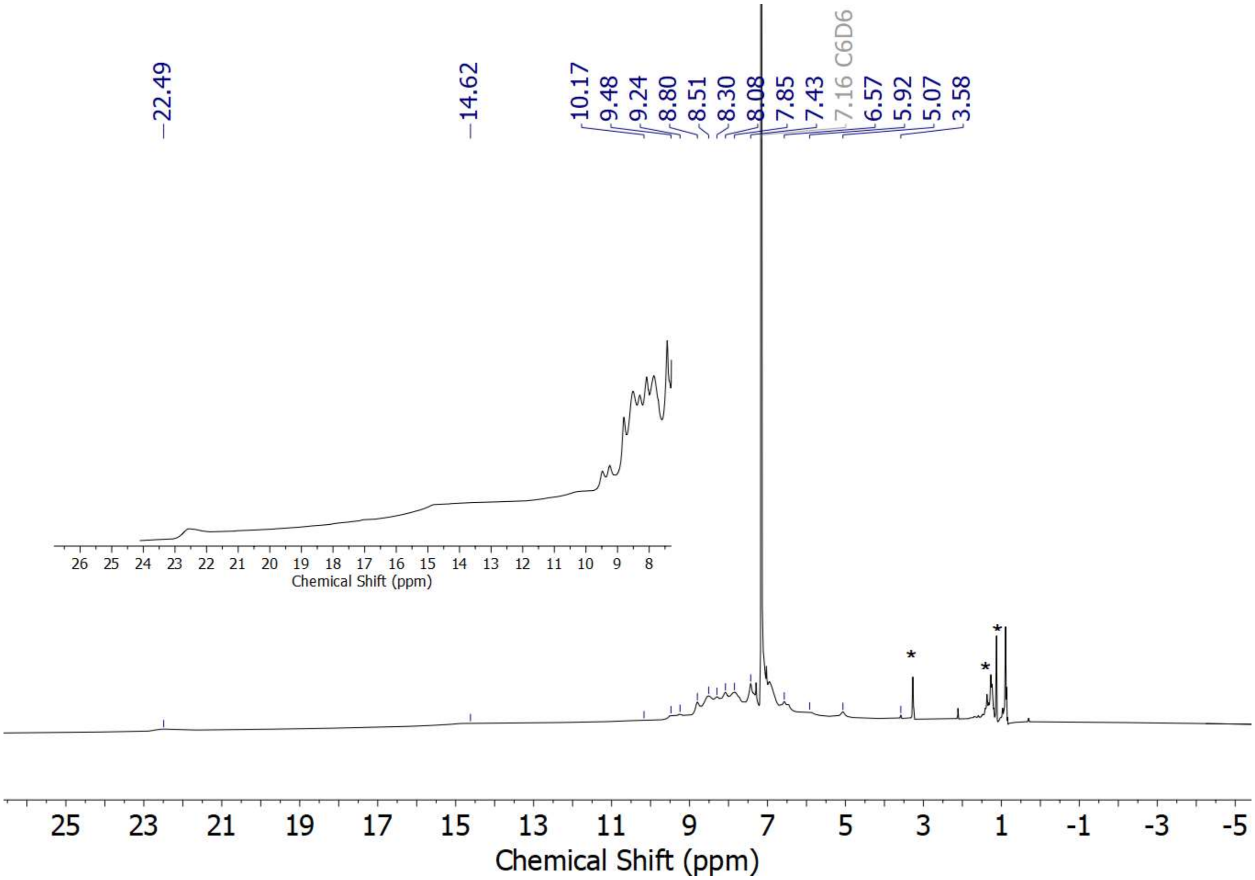

Figure S-16. ^{1}H NMR (600 MHz) spectrum for paramagnetic $[Fe_2(^{Ph}Dbf)_2(\mu{-}NC_8H_3F_6)(NC_5H_5)]$ (**6a**). Inlay of identifying paramagnetic peaks at high chemical shifts. *indicates minor organic impurities remaining from starting material wash (ligand/hexanes).

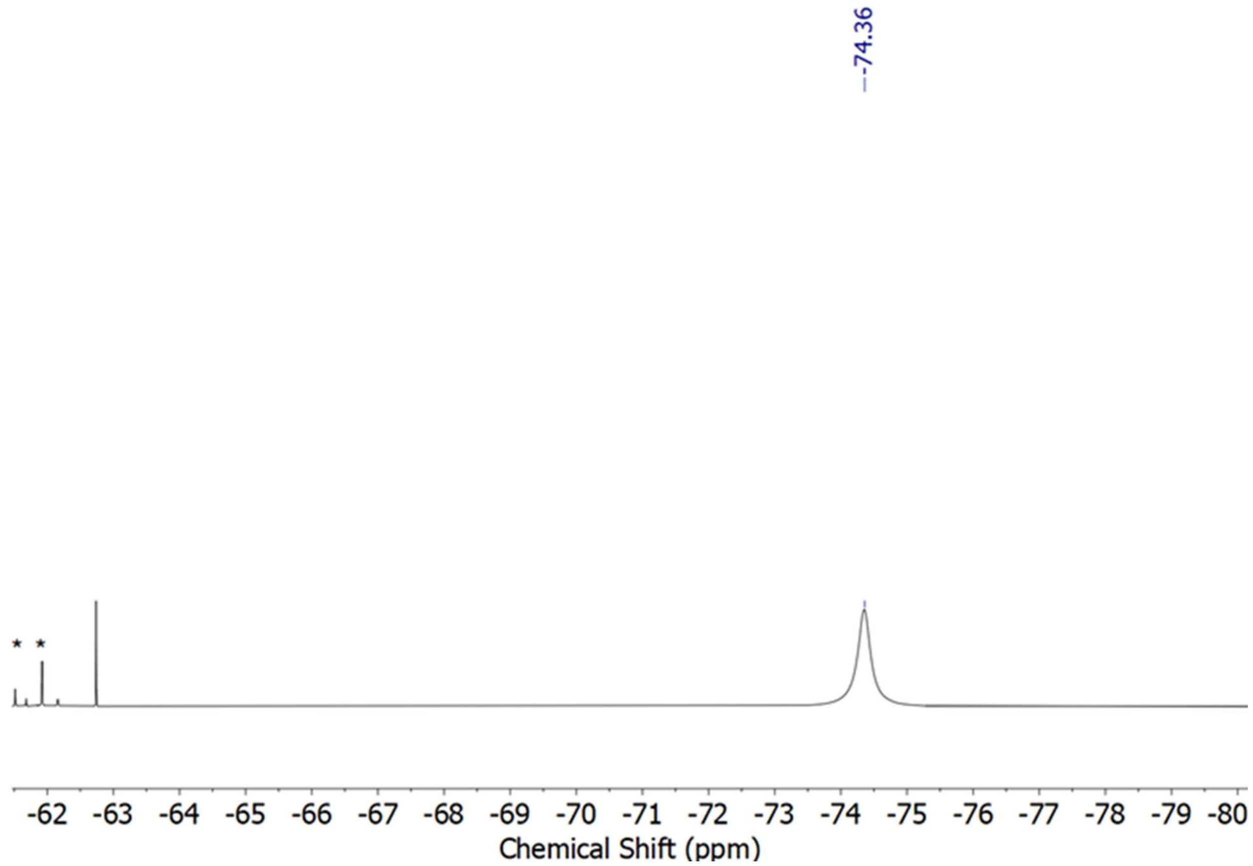

Figure S-17. ^{19}F NMR (376 MHz) spectrum for paramagnetic $[Fe_2(^{Ph}Dbf)_2(\mu{-}NC_8H_3F_6)(NC_5H_5)]$ (**6a**) referenced to trifluorotoluene (-62.74 ppm). * indicates minor organic impurity.

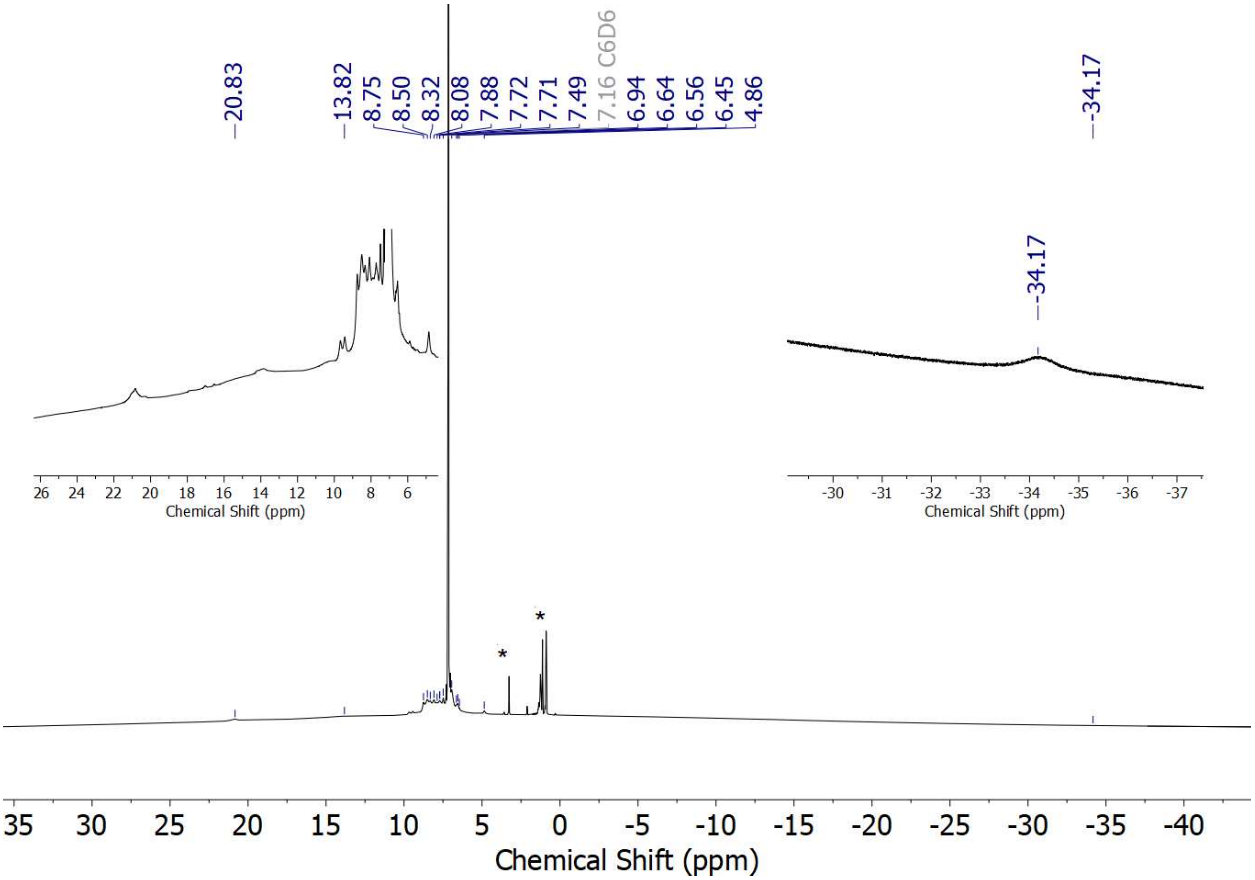

Figure S-18. ^{1}H NMR (600 MHz) spectrum for paramagnetic $[Fe_2(^{Ph}Dbf)_2(\mu{-}NC_8H_3F_6)(NC_6H_4F_3)]$ (**6b**). *indicates minor organic impurities remaining from starting material wash (ligand/hexanes).

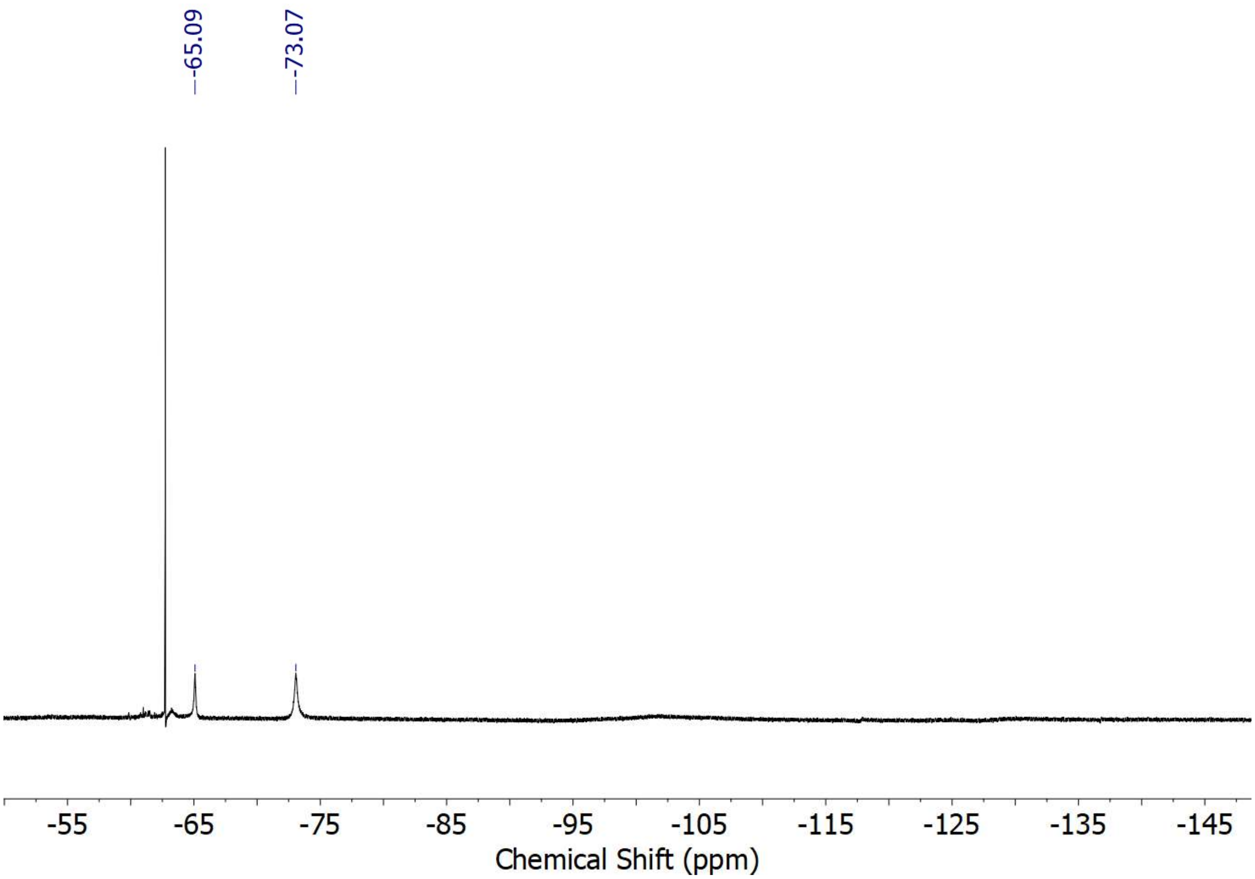

Figure S-19. ^{19}F NMR (376 MHz) spectrum for paramagnetic $[Fe_2(^{Ph}Dbf)_2(\mu-NC_8H_3F_6)(NC_6H_4F_3)]$ (**6b**) referenced to trifluorotoluene (-62.74 ppm).

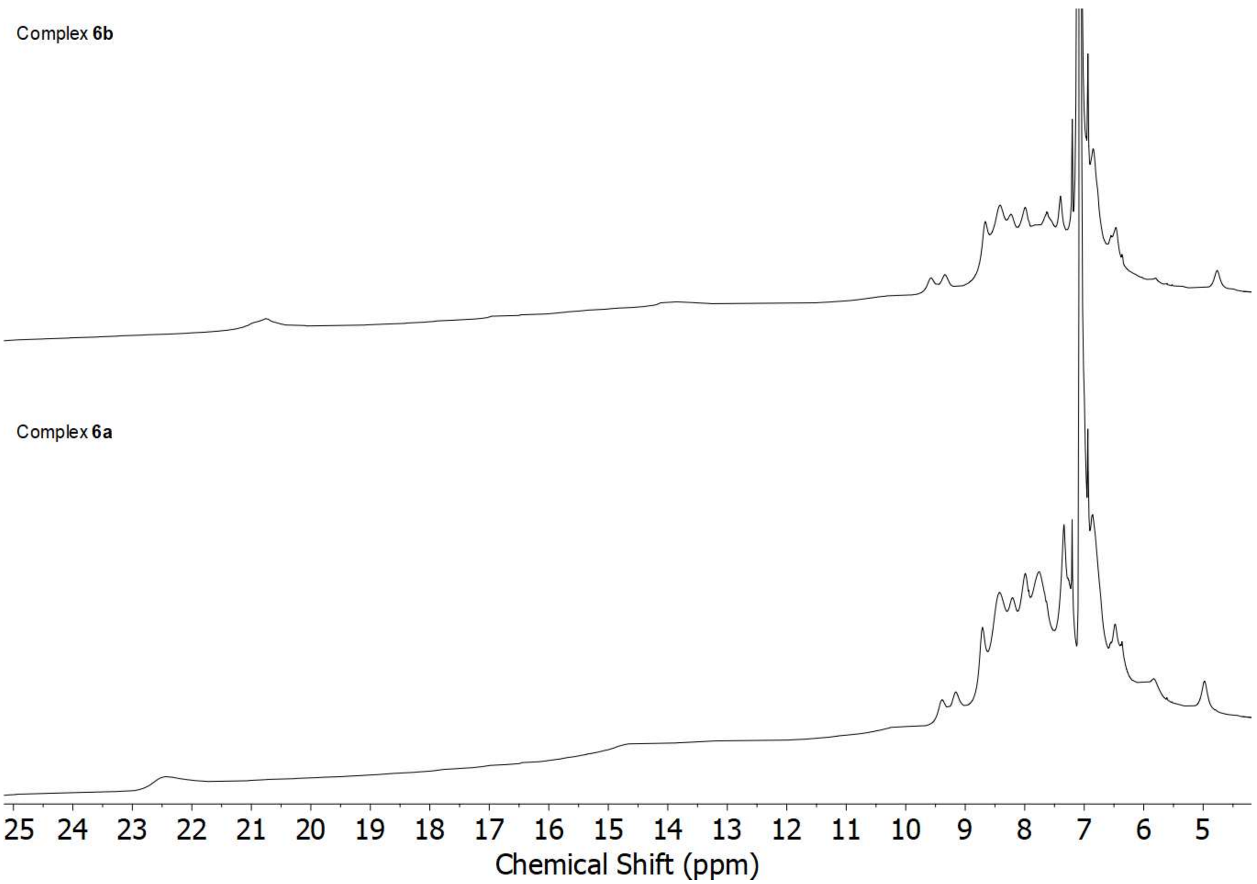

Figure S-20. ^{1}H NMR (600 MHz) spectra for paramagnetic [Fe_2(^{Ph}Dbf)$_2$(μ−$NC_8H_3F_6$)(NC_5H_5)] (**6a**, *bottom*) and [Fe_2(^{Ph}Dbf)$_2$(μ−$NC_8H_3F_6$)($NC_6H_4F_3$)] (**6b**, *top*). Spectra zoomed in from 4.8 ppm to 25 ppm for clarity. The full spectrum for each complex is located above.

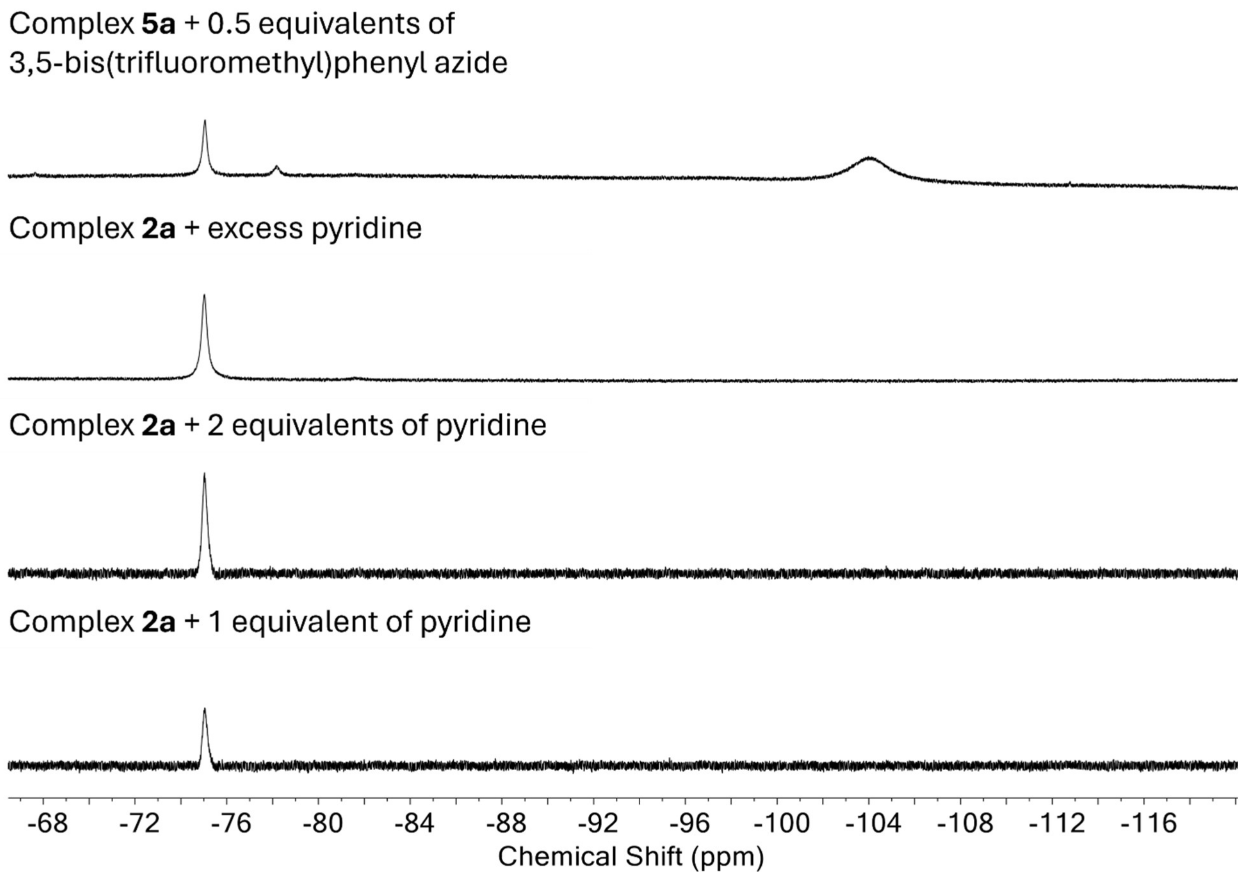

Figure S-21. ^{19}F NMR (376 and 564 (*top*) MHz) spectra for [Fe_2(^{Ph}Dbf)$_2$(μ–$NC_8H_3F_6$)] **2a** upon addition of 1 equivalent (*bottom*), 2 equivalent (*middle*), excess pyridine (*middle*), and for complex [Fe(^{Ph}Dbf)(NC_5H_5)$_2$] **5a** upon addition of 0.5 equivalents of 3,5-bis(trifluoromethyl)phenylazide. A single species [Fe_2(^{Ph}Dbf)$_2$(μ–$NC_8H_3F_6$)(NC_5H_5)] (**6a**) is observed in ^{19}F NMR upon addition of excess pyridine to [Fe_2(^{Ph}Dbf)$_2$(μ–$NC_8H_3F_6$)] **2a**; however, a second paramagnetic species is formed upon addition of 0.5 eq. of 3,5-bis(trifluoromethyl)phenylazide to [Fe(^{Ph}Dbf)(NC_5H_5)$_2$] **5a**. This suggests the formation of the second paramagnetic species in the ^{19}F NMR can be avoided by accessing the Py bound imido through the addition of pyridine directly to complex [Fe_2(^{Ph}Dbf)$_2$(μ–$NC_8H_3F_6$)] **2a**.

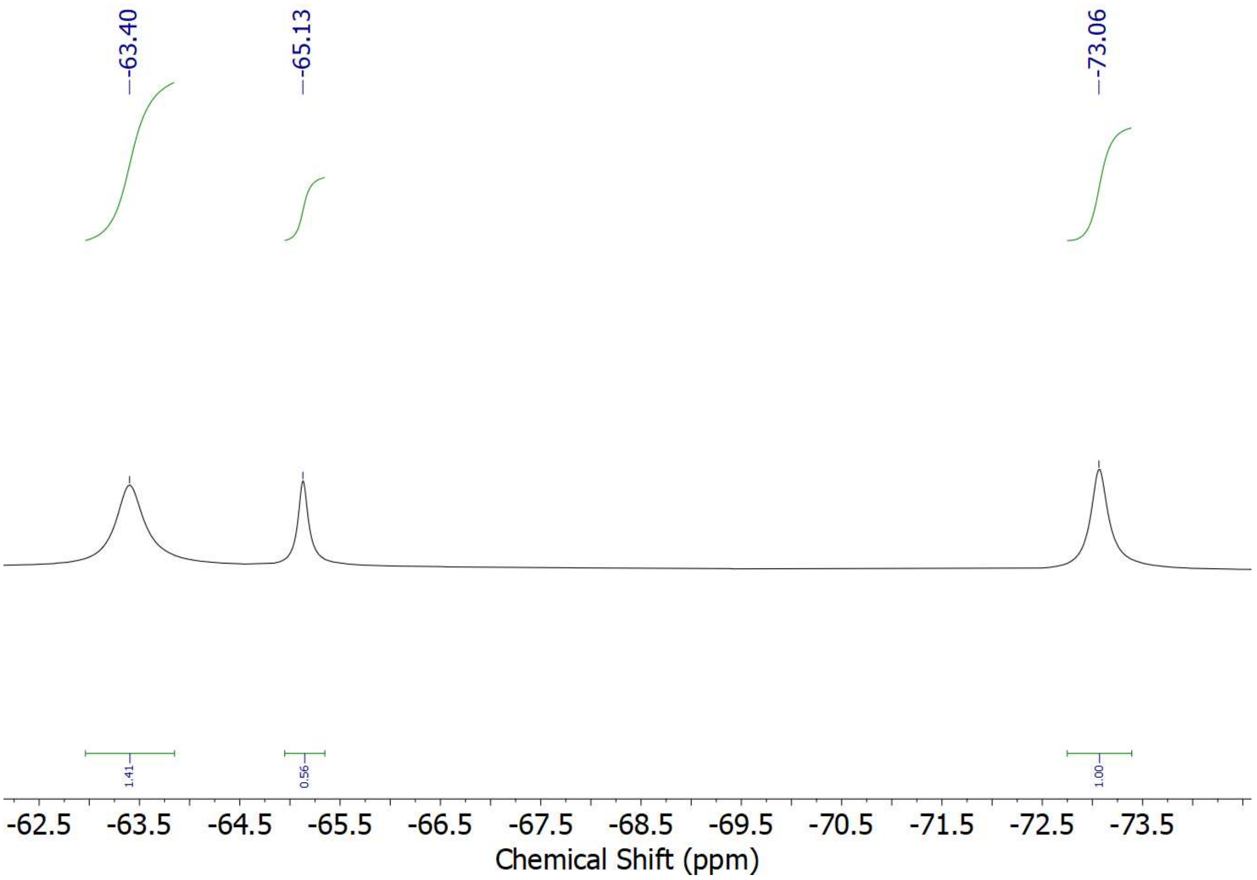

Figure S-22. Integrated ^{19}F NMR (564 MHz, benzene-d_6) spectrum for the reaction of $[Fe_2(^{Ph}Dbf)_2(\mu{-}NC_8H_3F_6)]$ **2a** with 2 equivalents of 4-trifluoromethylpyridine. Peak at -73.06 ppm corresponds to the two trifluoromethyl groups on the aryl azide ligand and the peak at -65.13 ppm corresponds to the single trifluoromethyl group on the substituted pyridine ligand on $[Fe_2(^{Ph}Dbf)_2(\mu{-}NC_8H_3F_6)(NC_6H_4F_3)]$ **6b**. These peaks integrate to the expected ratio of 2:1 for a complex with one pyridine ligand bound. The peak at -63.40 ppm represents free 4-trifluoromethyl pyridine. This excess pyridine can be removed via trituration with hexanes, followed by lyophilization of the complex in benzene.

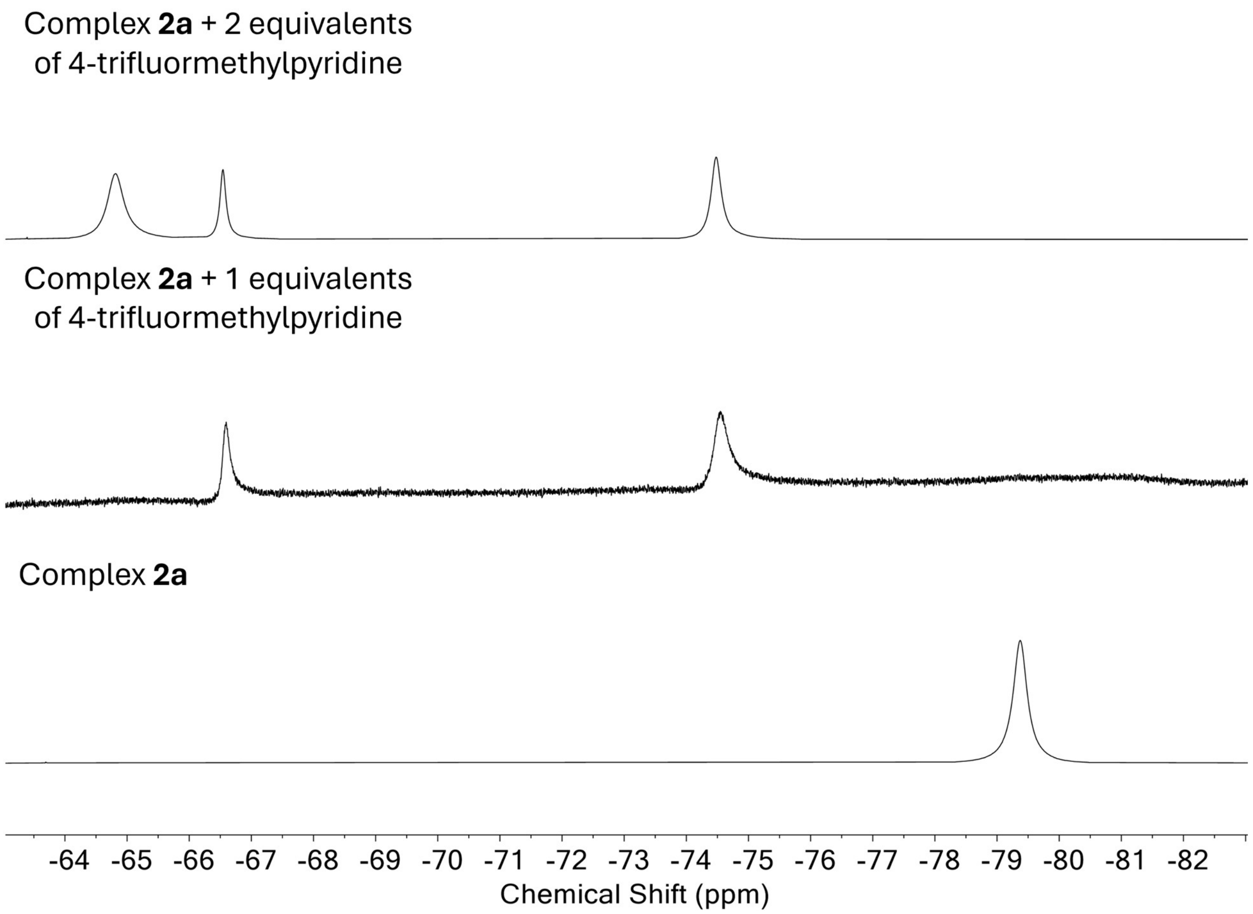

Figure S-23. Stacked ^{19}F NMR (564 MHz, benzene-d_6) spectra for the reaction of $[Fe_2(^{Ph}Dbf)_2(\mu{-}NC_8H_3F_6)]$ **2a** (*bottom*) with 1 (*middle*) and 2 (*top*) equivalents of 4-trifluoromethylpyridine. Complex $[Fe_2(^{Ph}Dbf)_2(\mu{-}NC_8H_3F_6)(NC_6H_4F_3)]$ **6b** is formed upon addition of 1 equivalent of pyridine (*middle*) and $[Fe_2(^{Ph}Dbf)_2(\mu{-}NC_8H_3F_6)]$ **2a** is completely consumed; however upon addition of a second equivalent of pyridine, $[Fe_2(^{Ph}Dbf)_2(\mu{-}NC_8H_3F_6)(NC_6H_4F_3)]$ **6b** and excess 4-trifluoromethylpyridine are observed.

Electronic Paramagnetic Resonance (EPR) Spectra

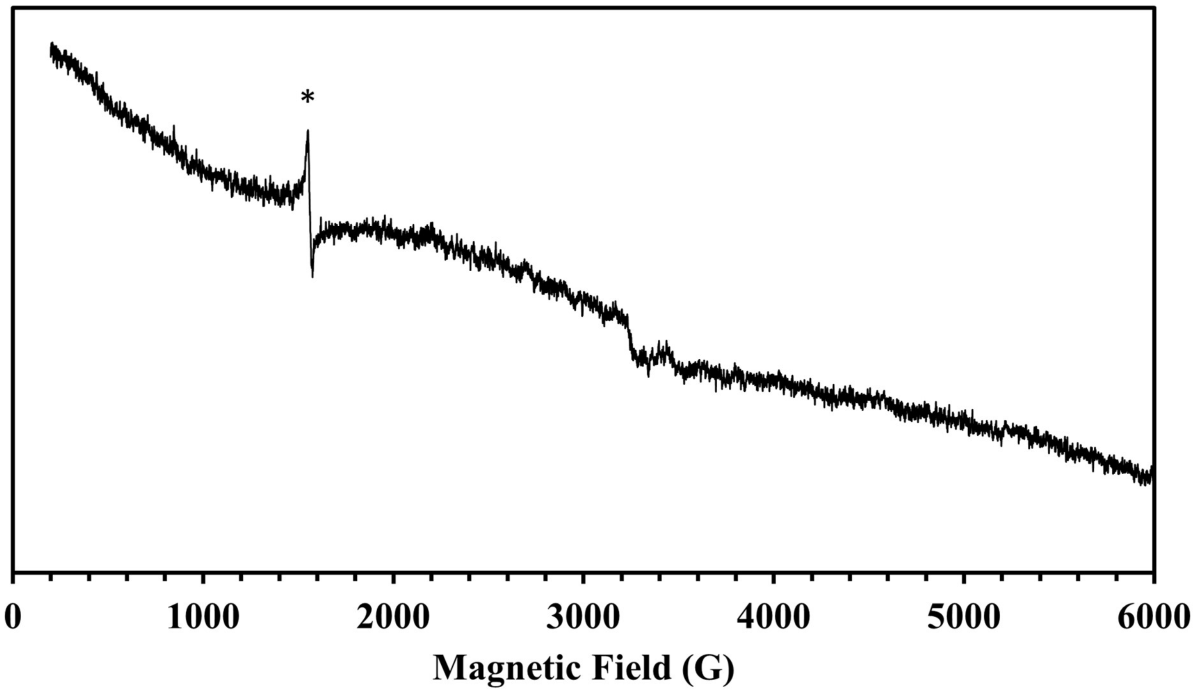

Figure S-24. Frozen benzene EPR (80 K) spectrum of [Fe_2(^{Ph}Dbf)$_2$(μ−$NC_8H_3F_6$)] (**2a**). No signal was expected for **2a** due to the observed antiferromagnetic coupling between the iron centers. * indicates minor Fe(III) impurity with low intensity suspected to be the bis(amide) [Fe_2(^{Ph}Dbf)$_2$(μ−$NHC_8H_3F_6$)($NHC_8H_3F_6$)] **3**, due to the similar g-value and shape. Benzene was chosen as the solvent to avoid any HAA from occurring.

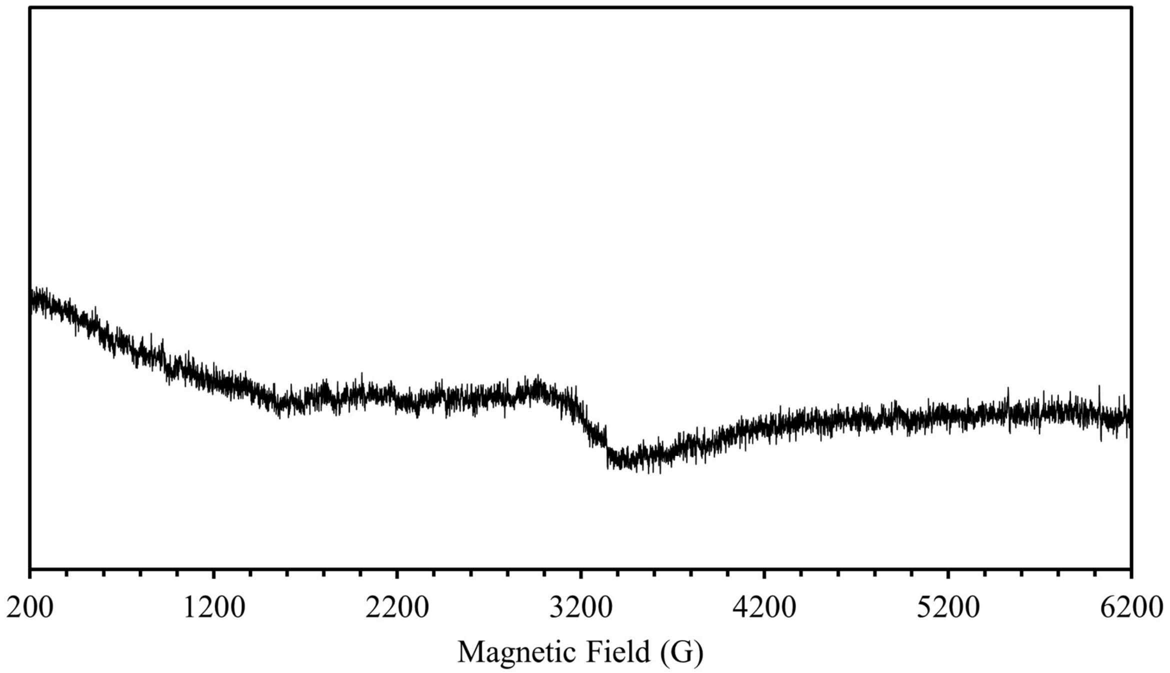

Figure S-25. Frozen benzene EPR (80 K) spectrum of $[Fe_2(^{Ph}Dbf)_2(\mu{-}NC_{10}H_{13})]$ (**2b**). Benzene was chosen as the solvent to avoid any HAA from occurring.

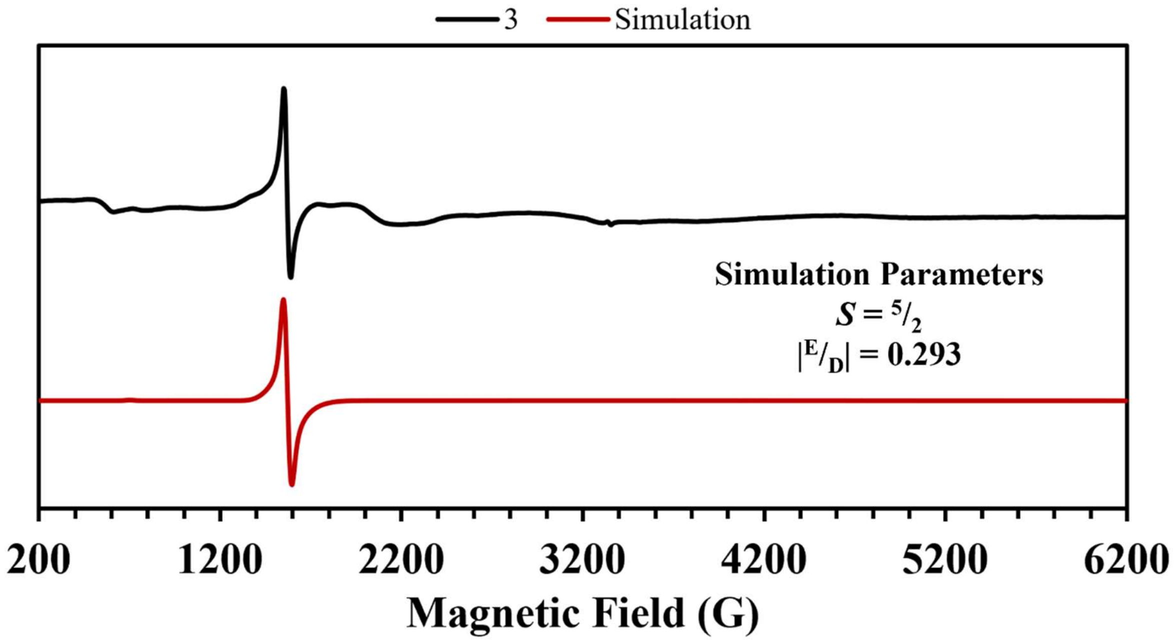

Figure S-26. Frozen toluene EPR (80 K) spectrum of $[Fe_2(^{Ph}Dbf)_2(\mu{-}NHC_8H_3F_6)(NHC_8H_3F_6)]$ (**3**) (g_{eff} = 4.27). The red line represents a simulation with VisualRhombo.[2]

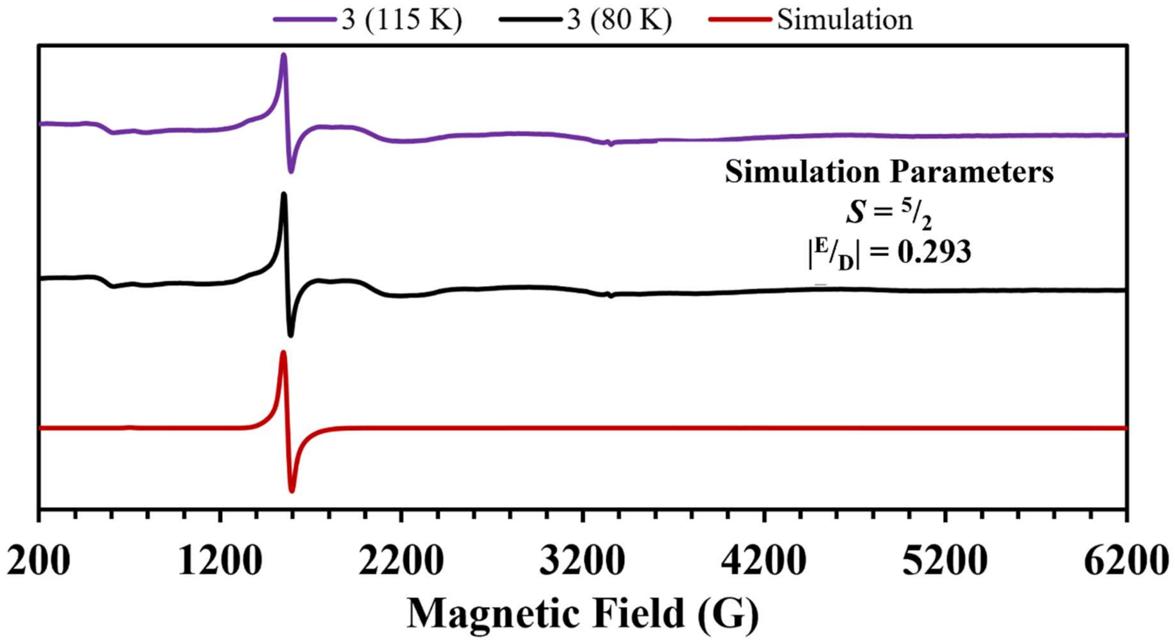

Figure S-27. Stacked frozen toluene EPR spectra at 80 K and 115 K of $[Fe_2(^{Ph}Dbf)_2(\mu{-}NHC_8H_3F_6)(NHC_8H_3F_6)]$ (**3**) (g_{eff} =4.27). The red line represents a simulation with VisualRhombo.[2]

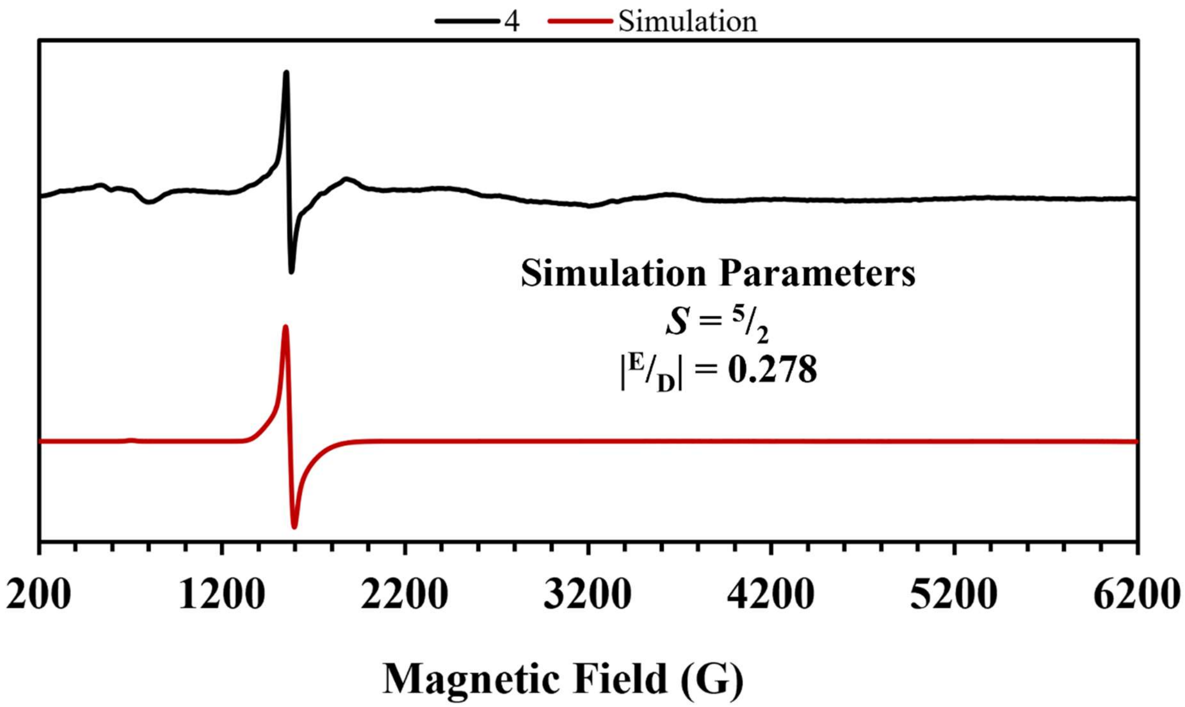

Figure S-28. Frozen toluene EPR (80 K) spectrum of the reaction of $[Fe_2(^{Ph}Dbf)_2(\mu{-}NHC_8H_3F_6)(OC_{19}H_{15})]$ (**4**), g_{eff} = 4.28. The red line represents a simulation with VisualRhombo.[2]

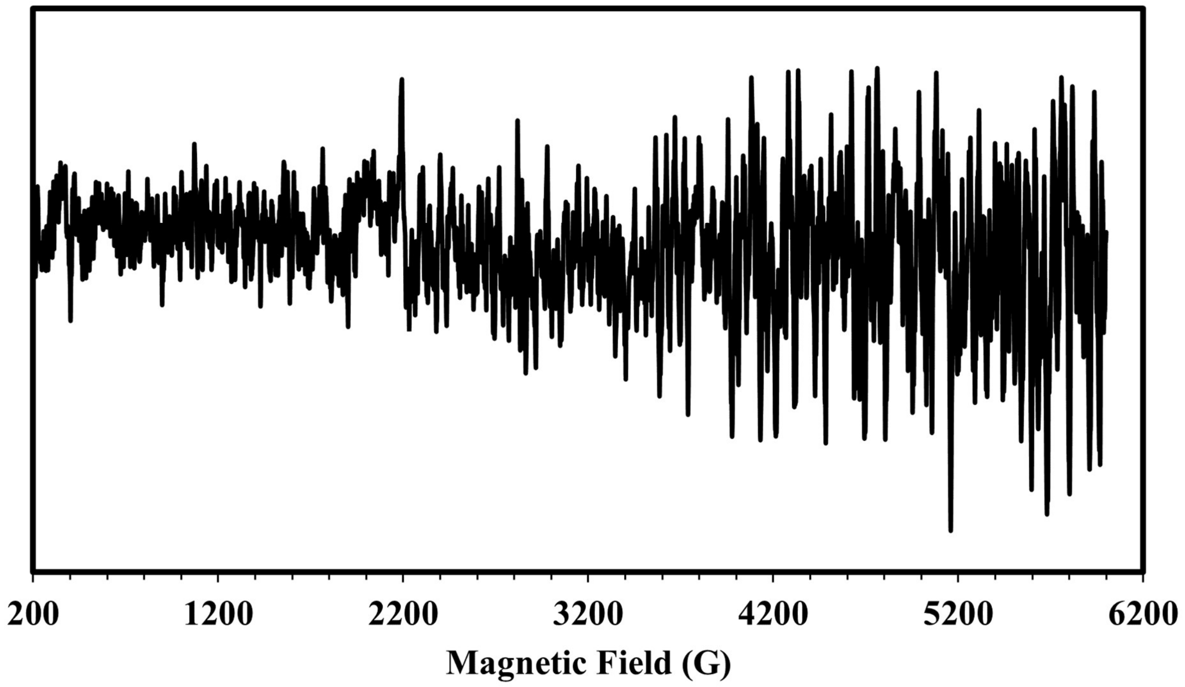

Figure S-29. Frozen benzene EPR (80 K) spectrum of the generation of $Fe_2(^{Ph}Dbf)_2(\mu{-}NC_8H_3F_6)(NC_5H_5)]$ (**6a**) via a reaction of $[Fe_2(^{Ph}Dbf)_2(\mu{-}NC_8H_3F_6)]$ (**2a**) with 1 equivalent of pyridine.

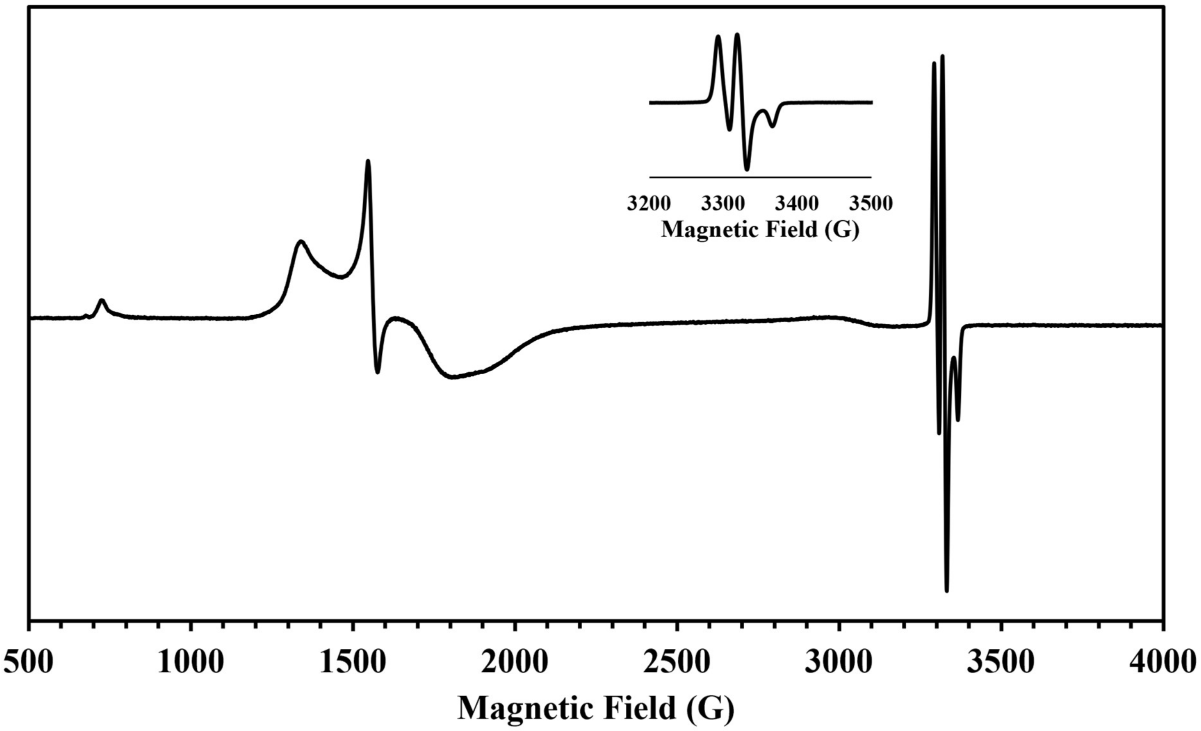

Figure S-30. Frozen toluene EPR (80 K) spectrum of the reaction of $[Fe_2(^{Ph}Dbf)_2(\mu{-}NC_8H_3F_6)]$ (**2a**) with 1 equivalent of 2-hydroxy-2-adamantane. Inlay of the zoomed organic region that corresponds to the hyperfine split signal from the AdNO radical. Paramagnetic species are assumed to be the bis(amide) $[Fe_2(^{Ph}Dbf)_2(\mu{-}NHC_8H_3F_6)(NHC_8H_3F_6)]$ (**3**) and the AdNO radical bound imido that are generated in the ^{19}F NMR as this reaction proceeds.

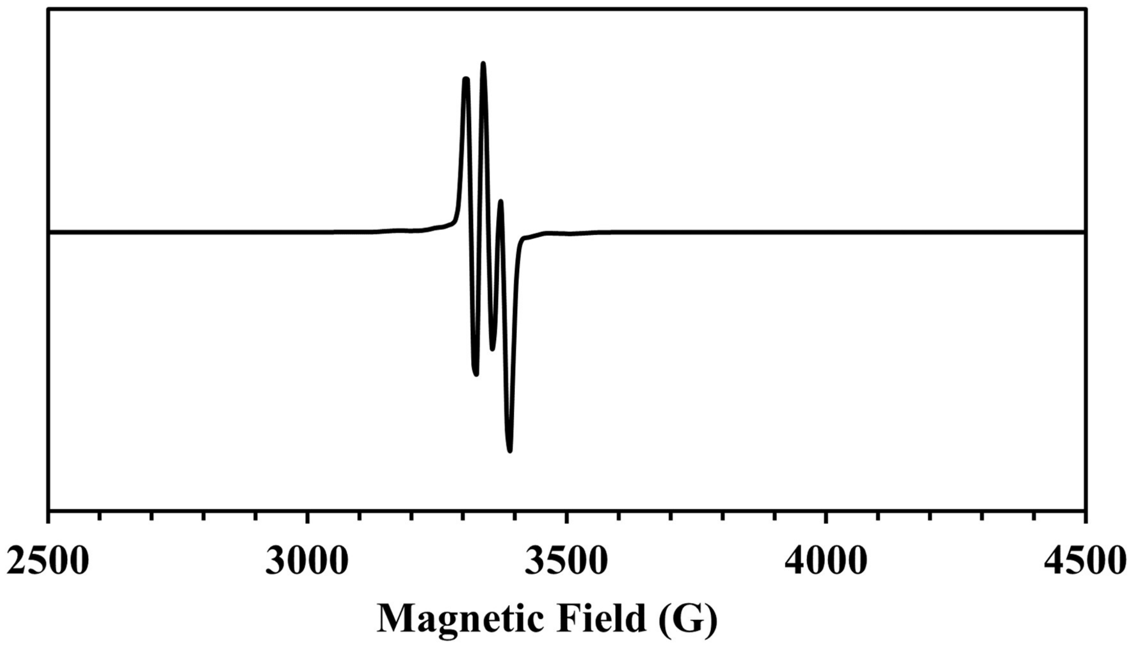

Figure S-31. Frozen toluene EPR (80 K) spectrum of 9-azabicyclo[3.3.1]nonane-N-oxyl.

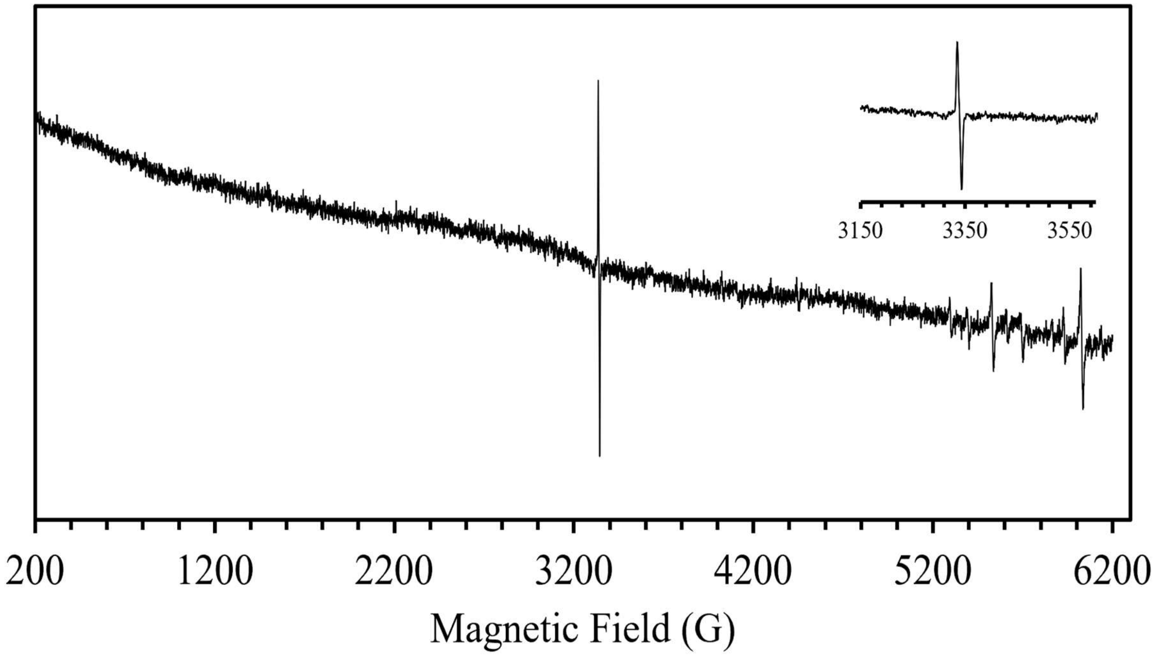

Figure S-32. RT benzene EPR spectrum of the reaction of [Fe_2(^{Ph}Dbf)$_2$(μ−$NC_8H_3F_6$)] (**2a**) with 1 equivalent 2,4,6-*tert*-butylphenol. The signal with a g_{eff} = 2.033 indicates an organic radical formed in solution implying HAA from the substrate had occurred.

Table S-2. Experimental g_{eff} values for Fe(III) complexes.

Complex	$\|^{E}/_{D}\|$ (Simulated)	g_{eff}
2a	-	Silent
2b	-	Silent
3	0.293	4.27
4	0.278	4.28
6a	-	Silent

IR Spectra

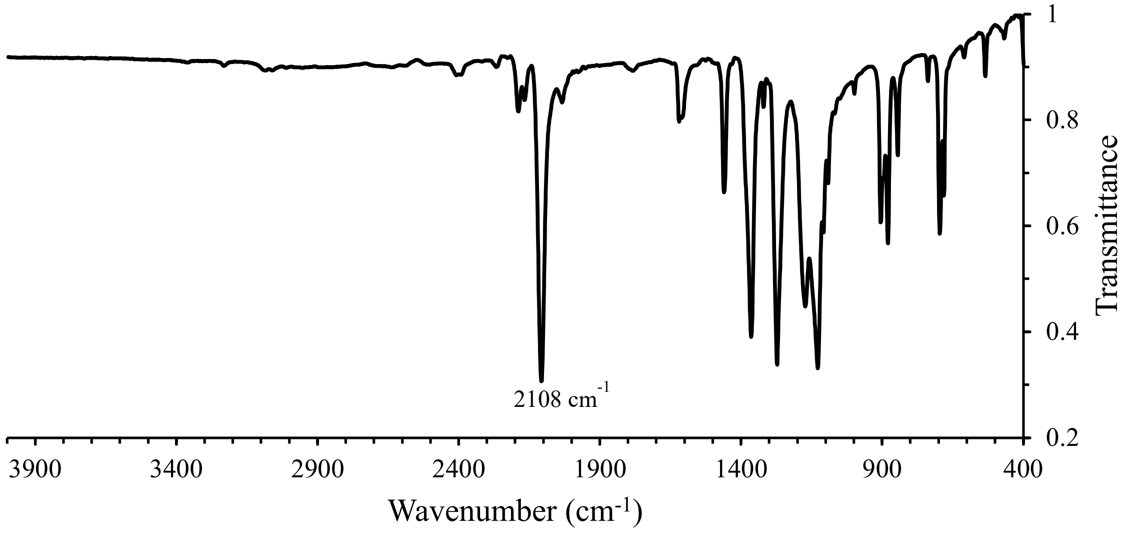

Figure S-33. Infrared Spectrum of liquid 3,5-bis(trifluoromethyl)phenyl azide. The azide stretch is marked at 2108 cm^{-1}.

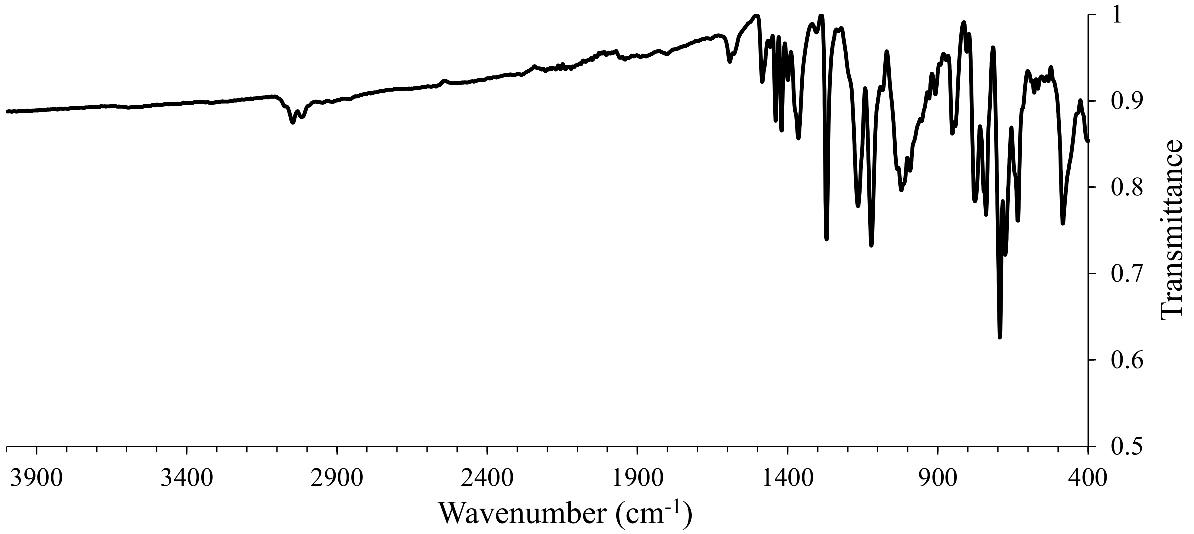

Figure S-34. Infrared Spectrum of solid [Fe_2(^{Ph}Dbf)$_2$(μ–$NC_8H_3F_6$)] (**2a**).

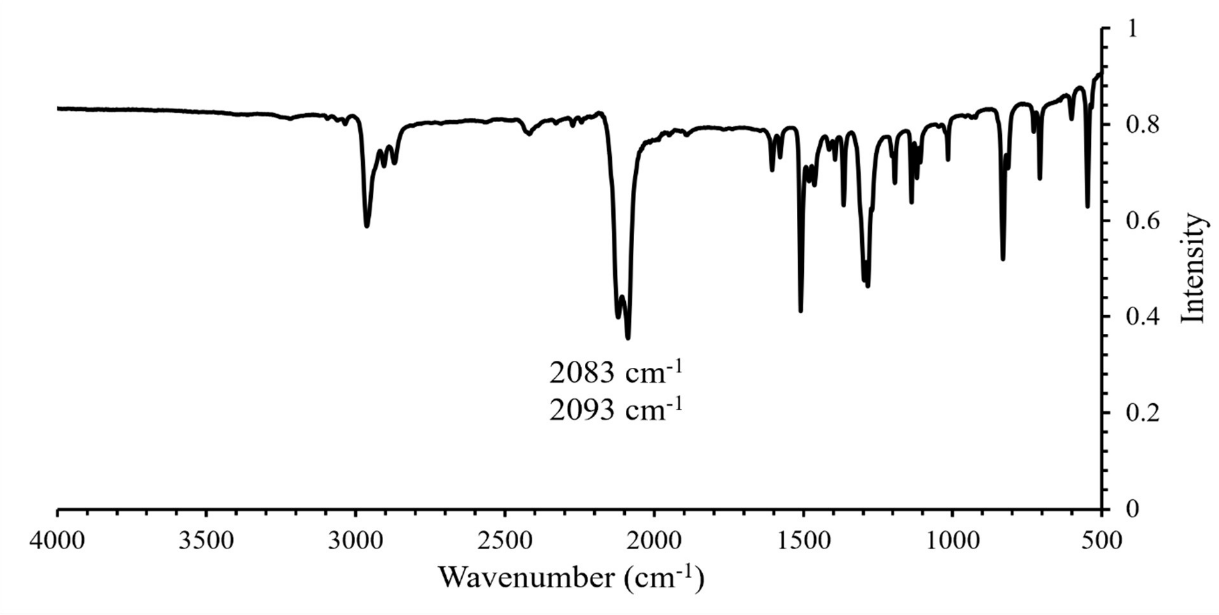

Figure S-35. Infrared Spectrum of liquid 4-*tert*-butylphenyl azide.

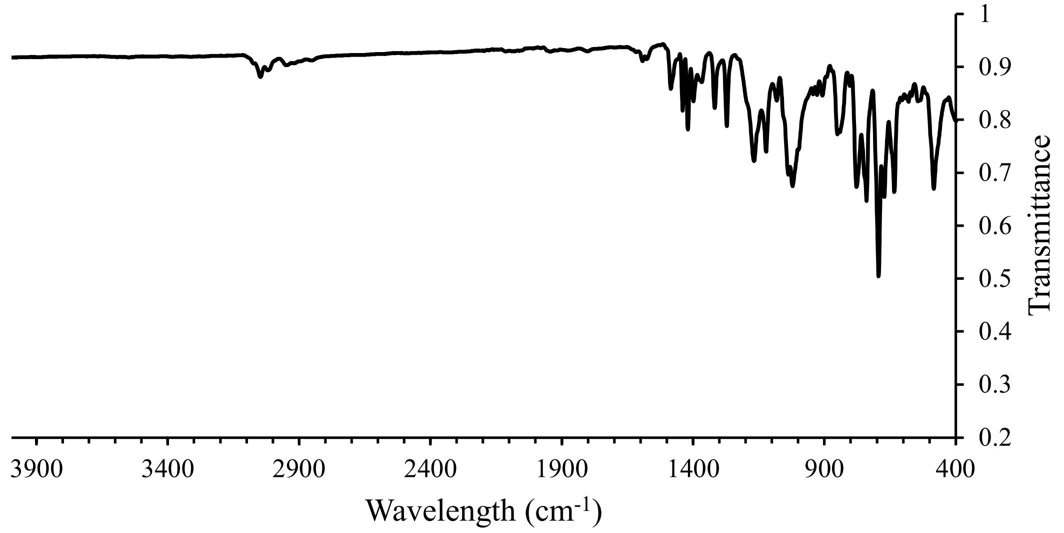

Figure S-36. Infrared Spectrum of solid [Fe_2(^{Ph}Dbf)$_2$(μ–$NC_{10}H_{13}$)] (**2b**).

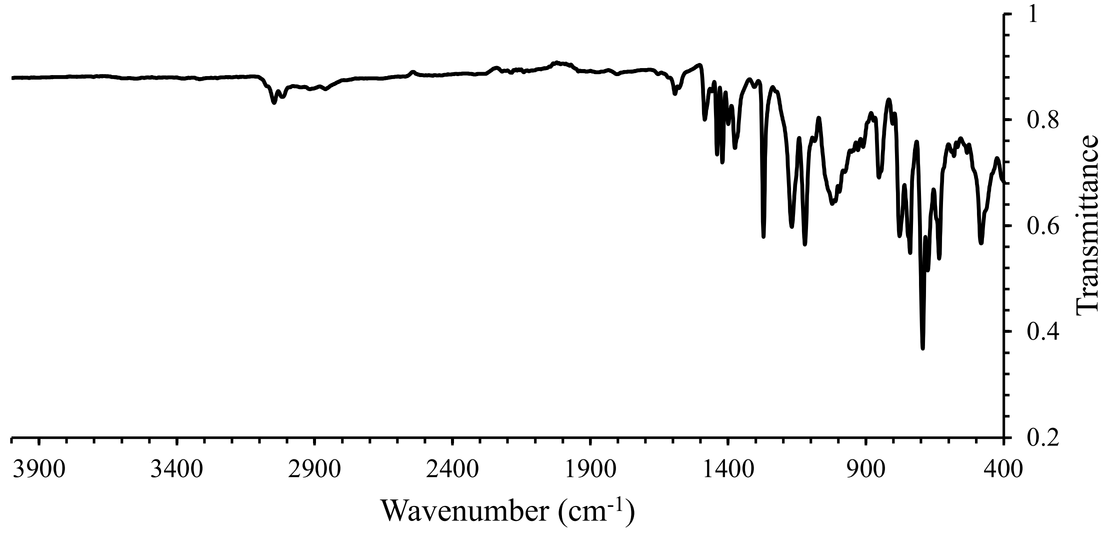

Figure S-37. Infrared Spectrum of solid [Fe_2(^{Ph}Dbf)$_2$(μ−$NHC_8H_3F_6$)($NHC_8H_3F_6$)] (**3**).

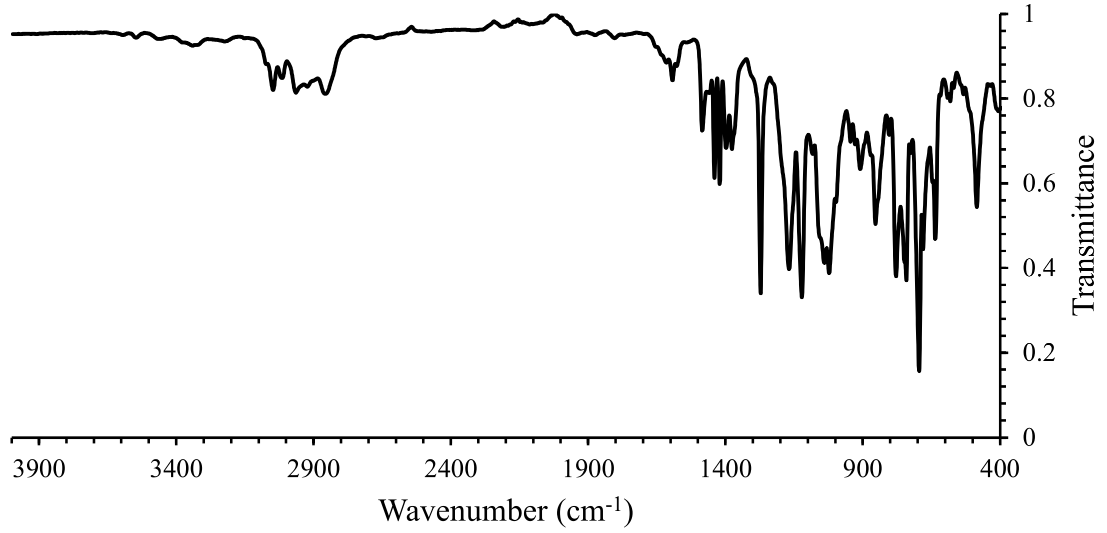

Figure S-38. Infrared Spectrum of liquid [Fe_2(^{Ph}Dbf)$_2$(μ−$NHC_8H_3F_6$)($NHC_8H_3F_6$)] (**3**).

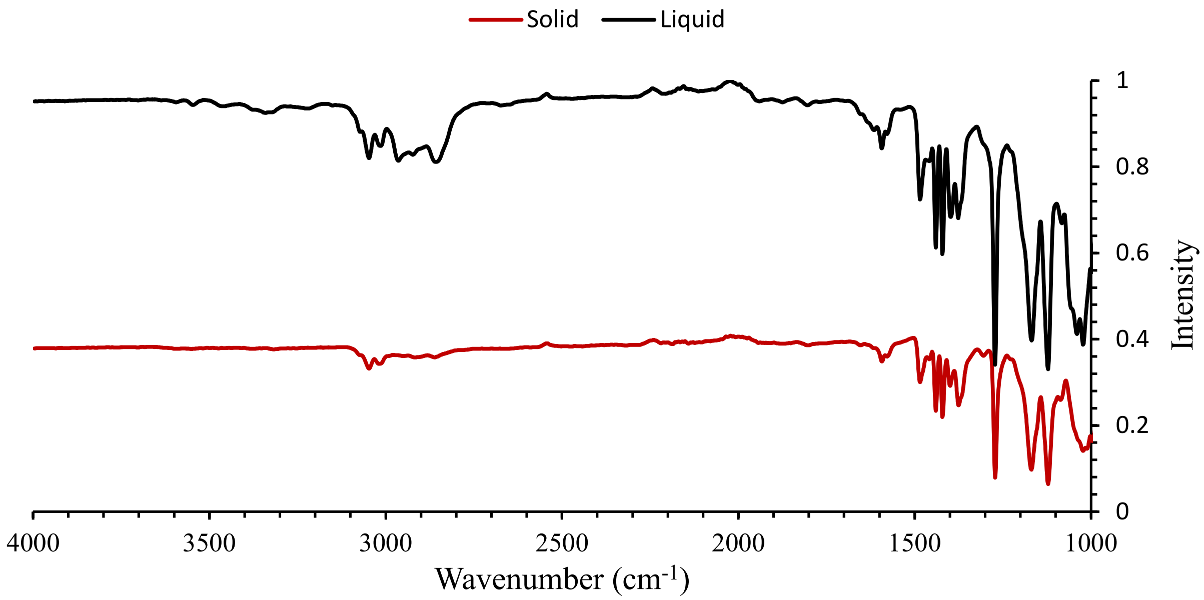

Figure S-39. Stacked infrared spectra of solid (red) and liquid (black) samples of $[Fe_2(^{Ph}Dbf)_2(\mu{-}NHC_8H_3F_6)(NHC_8H_3F_6)]$ (**3**) from 1000-4000 cm^{-1}.

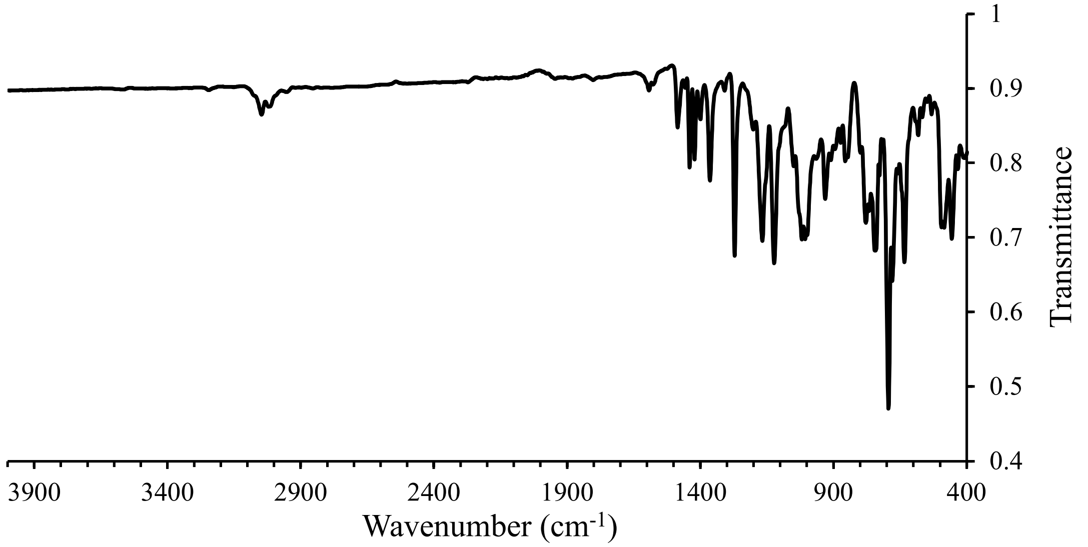

Figure S-40. Infrared Spectrum of solid $[Fe_2(^{Ph}Dbf)_2(\mu{-}NHC_8H_3F_6)(OC_{19}H_{15})]$ (**4**).

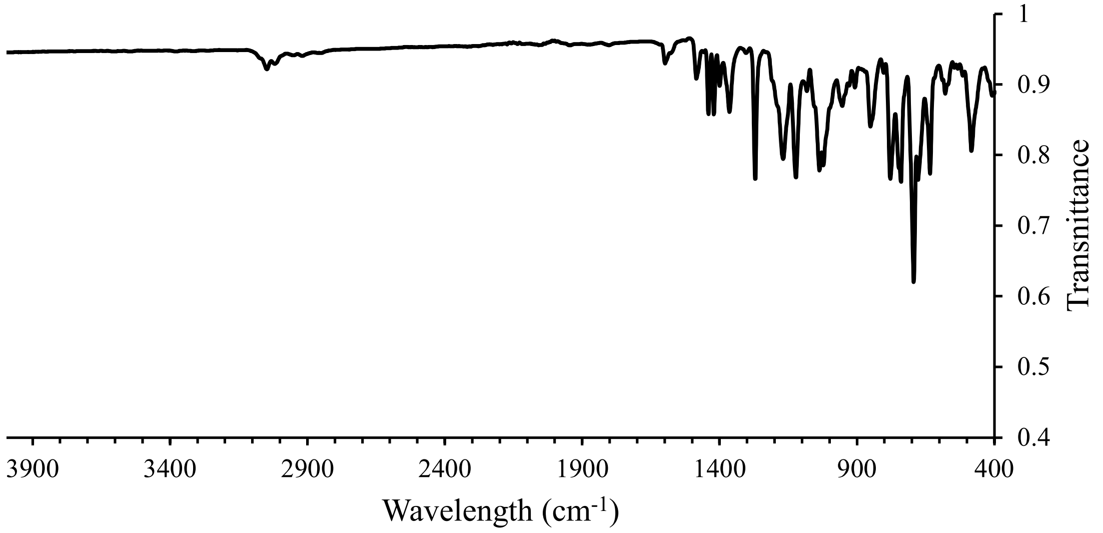

Figure S-41. Infrared Spectrum of solid [Fe_2(^{Ph}Dbf)$_2$(μ–$NC_8H_3F_6$)(NC_5H_5)] (**6a**).

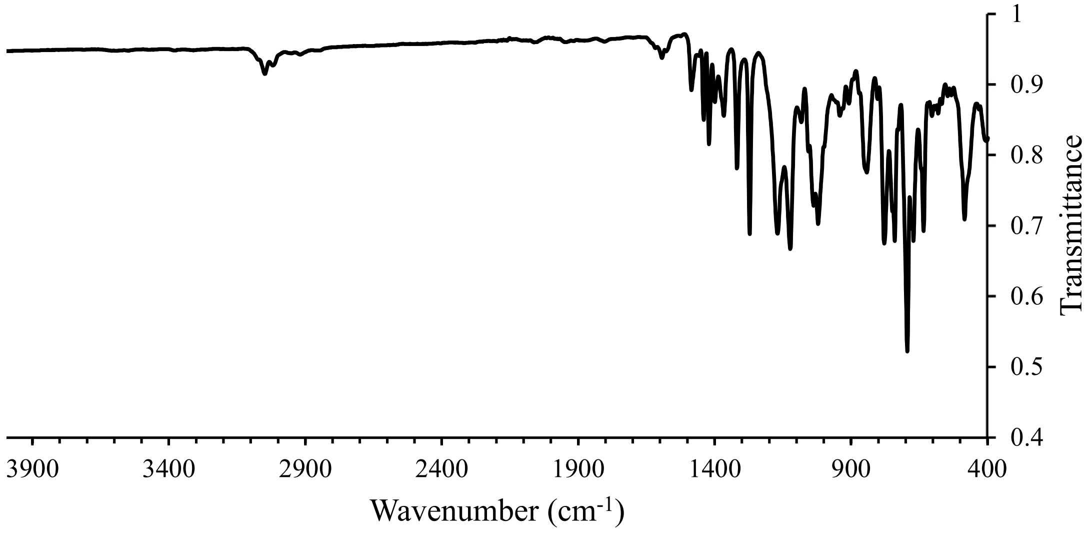

Figure S-42. Infrared Spectrum of solid [Fe_2(^{Ph}Dbf)$_2$(μ–$NC_8H_3F_6$)($NC_6H_4F_3$)] (**6b**).

Nitrene Capture Substrates

Table S-3. Reactions of **1** and **5a** with aryl azides.

Azide	Complex	Isolable Species	Time
3,5-bis(trifluoromethyl)phenyl*	1	Y	Instantaneous
	5a	Y	Instantaneous
4-*tert*-butylphenyl*	1	Y	Instantaneous
	5a	Y	Instantaneous
4-nitrophenyl	1	Y	Instantaneous
	5a	Y	Instantaneous
2,4,6-trimethylphenyl	1	Y	Overnight
	5a	Y	Instantaneous
Phenyl	1	N	–
	5a	N	–

Reagents with * were confirmed via X-ray diffraction and all other species were identified with ^{1}H NMR and IR spectra.

Reactions of 1 with other Aryl Azides

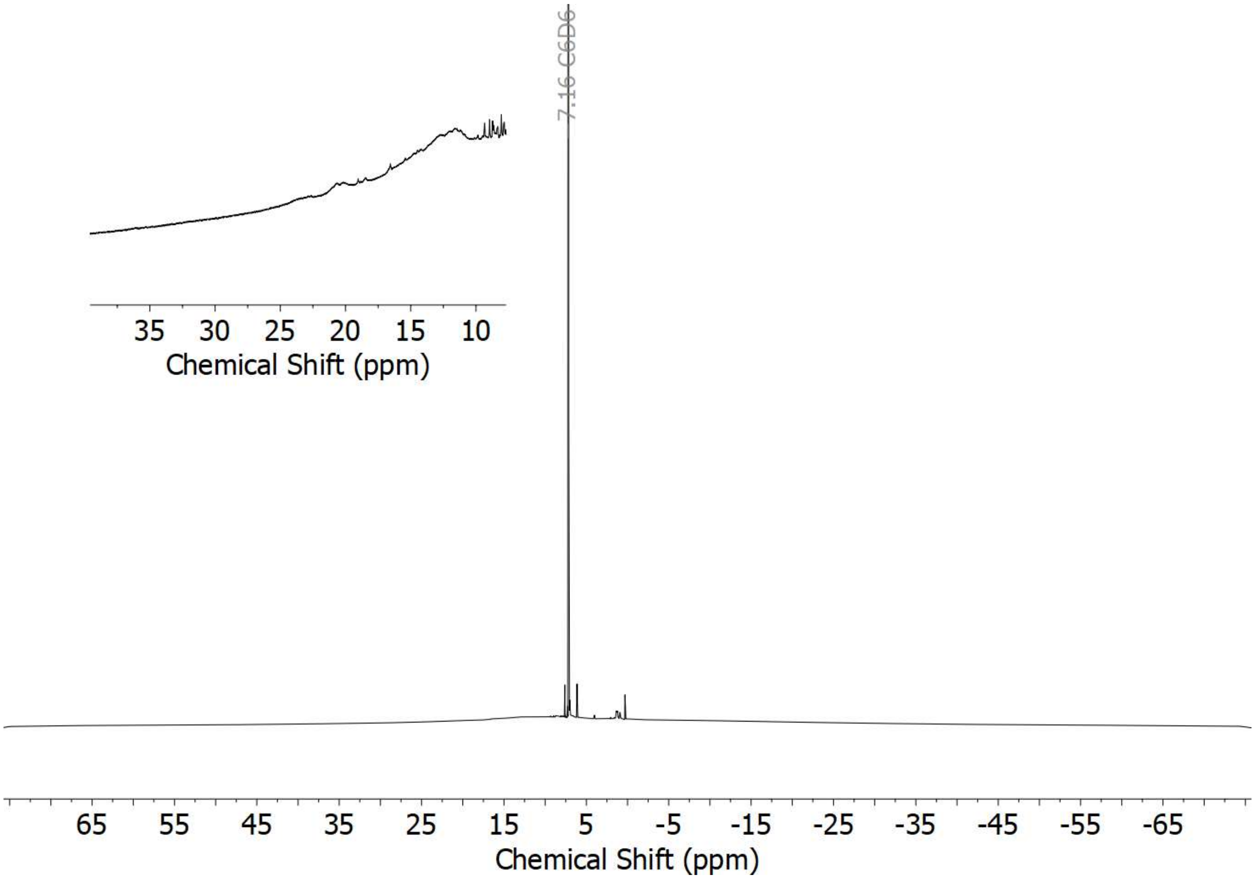

Figure S-43. ^{1}H NMR (600 Hz) spectrum of the reaction of [Fe_2(^{Ph}Dbf)$_2$] (**1**) with 4-nitrophenyl azide to form a new paramagnetic species (red).

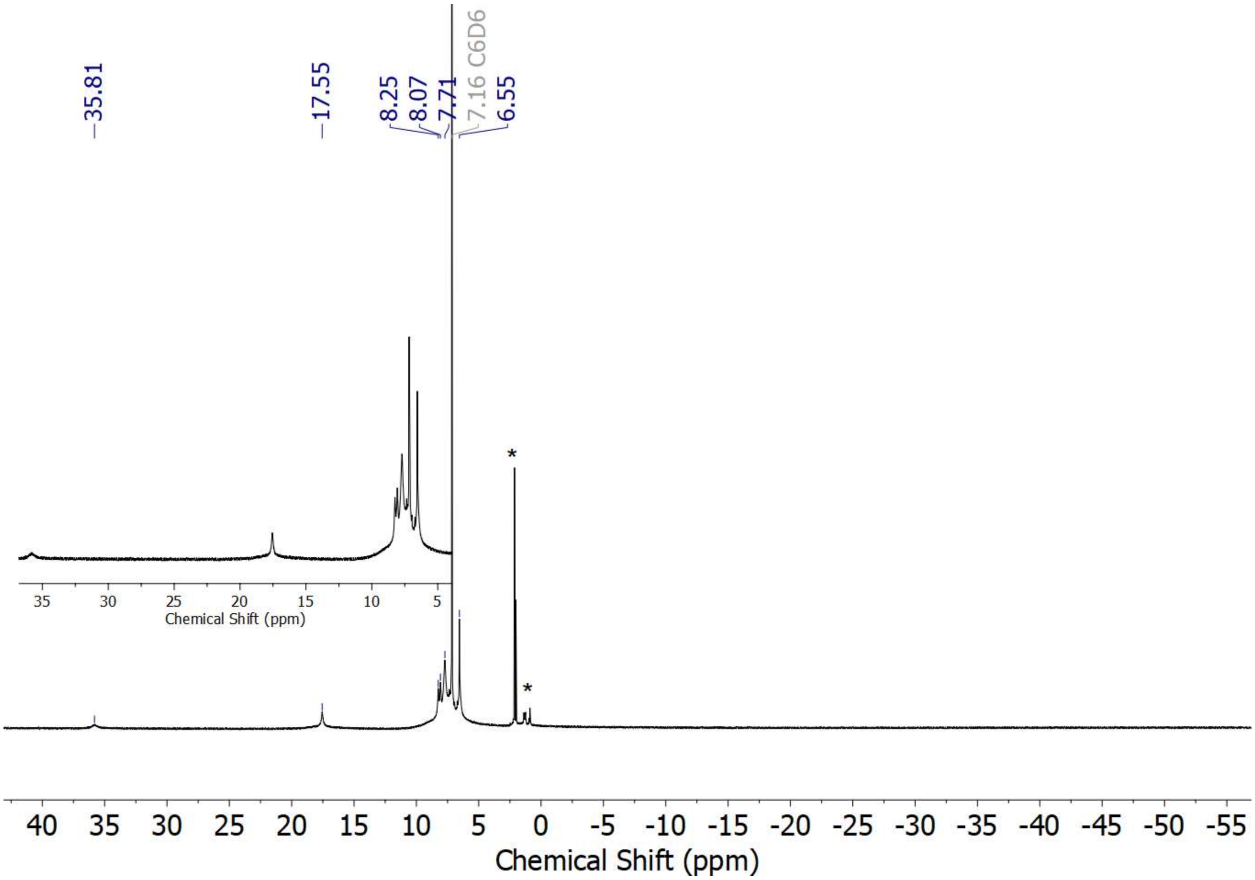

Figure S-44. ^{1}H NMR (600 Hz) spectrum of the reaction of [Fe_2(^{Ph}Dbf)$_2$] (**1**) with 2,4,6-trimethylphenyl azide to form a new paramagnetic species (yellow tinted). * indicates minor impurity from hexanes or excess 2,4,6-trimethylphenyl azide.

Reactions of 5a with other Azides

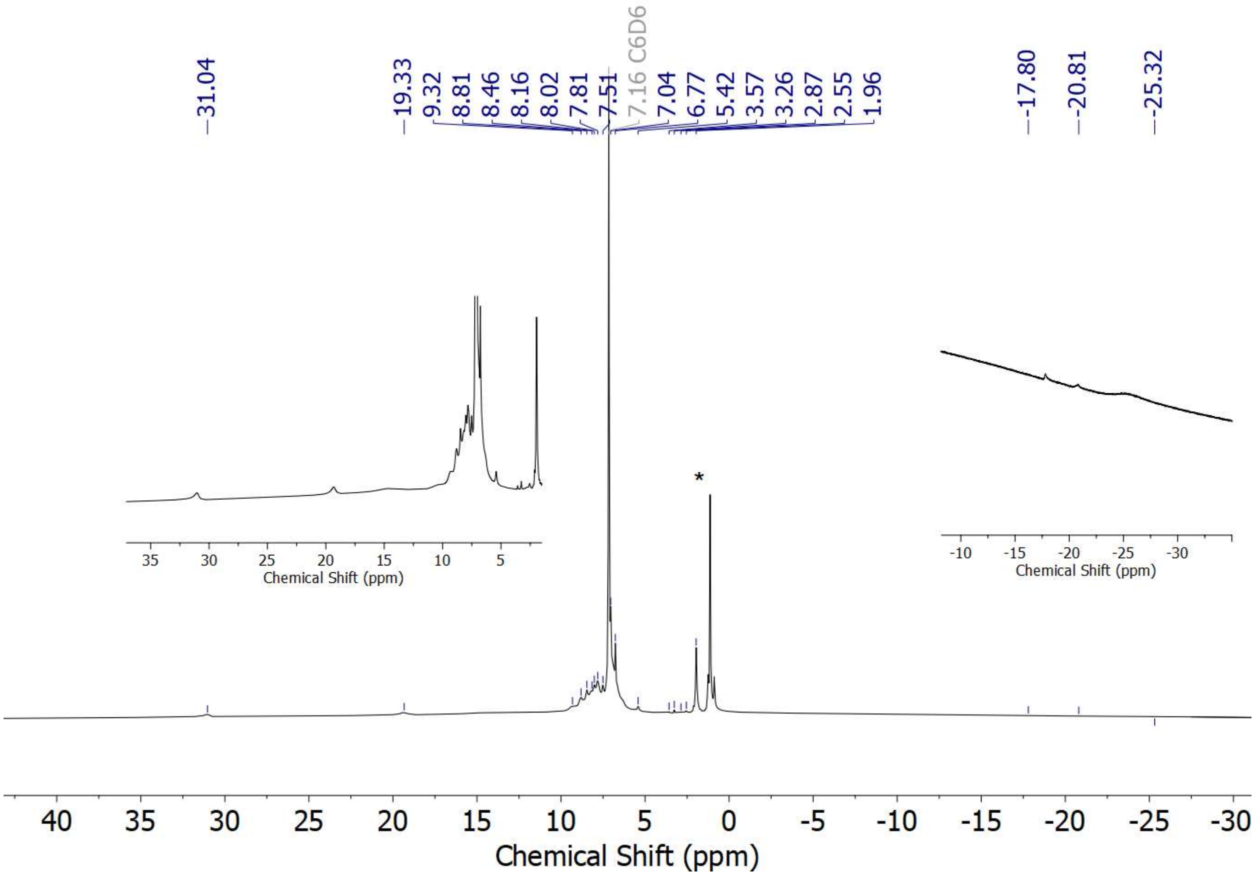

Figure S-45. ^{1}H NMR (600 Hz) spectrum of the reaction of [Fe(^{Ph}Dbf)(NC_5H_5)$_2$] (**5a**) with 4-*tert*-butylphenylazide to form a new paramagnetic species (dark green). Inlaid spectra are of paramagnetic peaks at positive chemical shifts (*left*) and negative chemical shifts (*right*). * indicates minor impurity from excess azide, hexanes, and ligand.

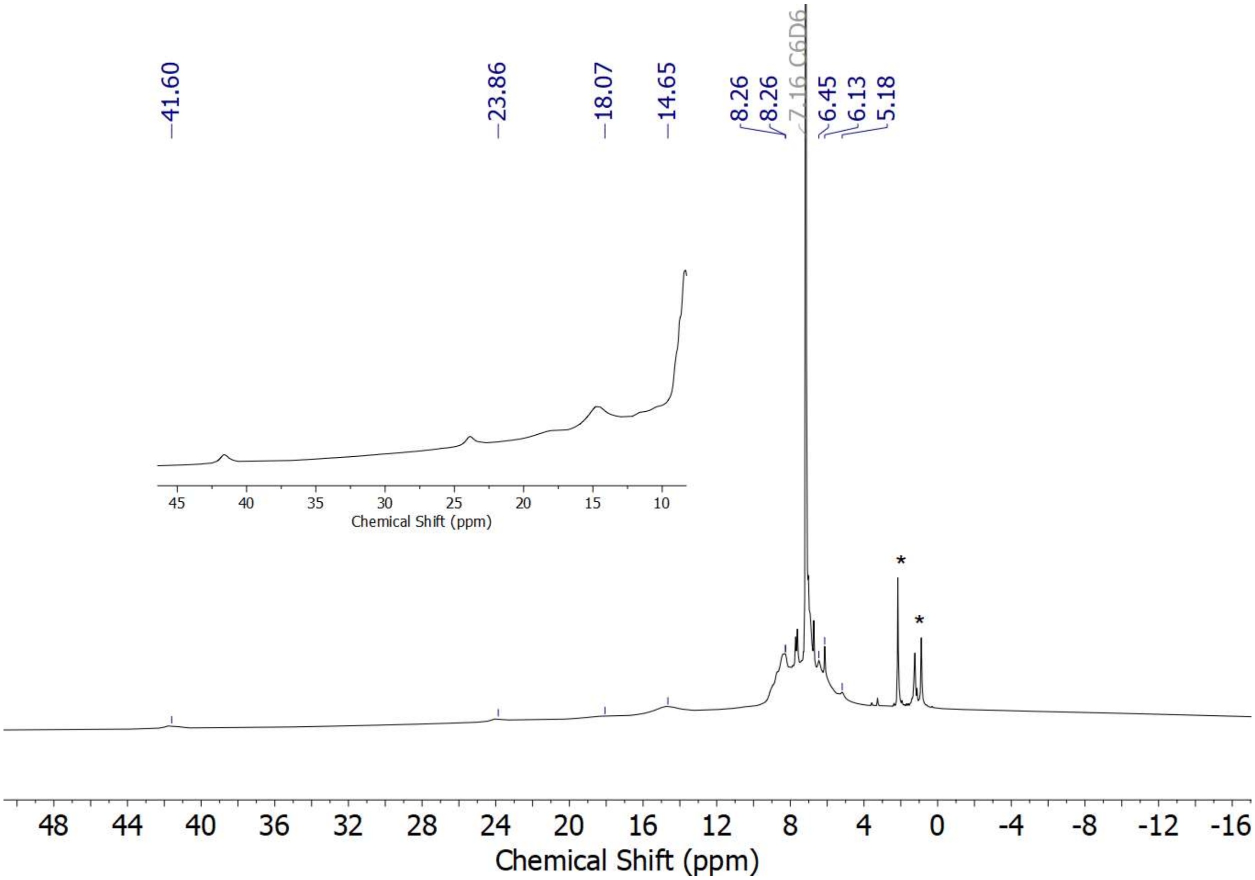

Figure S-46. ^{1}H NMR (600 Hz) spectrum of the reaction of [Fe(^{Ph}Dbf)($NC_5H_5)_2$] (**5a**) with 4-nitrophenylazide to form a new paramagnetic species (dark green). Inlay of paramagnetic peaks at high chemical shifts (*left*) and around the residual solvent peak (*right*). * indicates minor organic impurities from free ligand and hexanes.

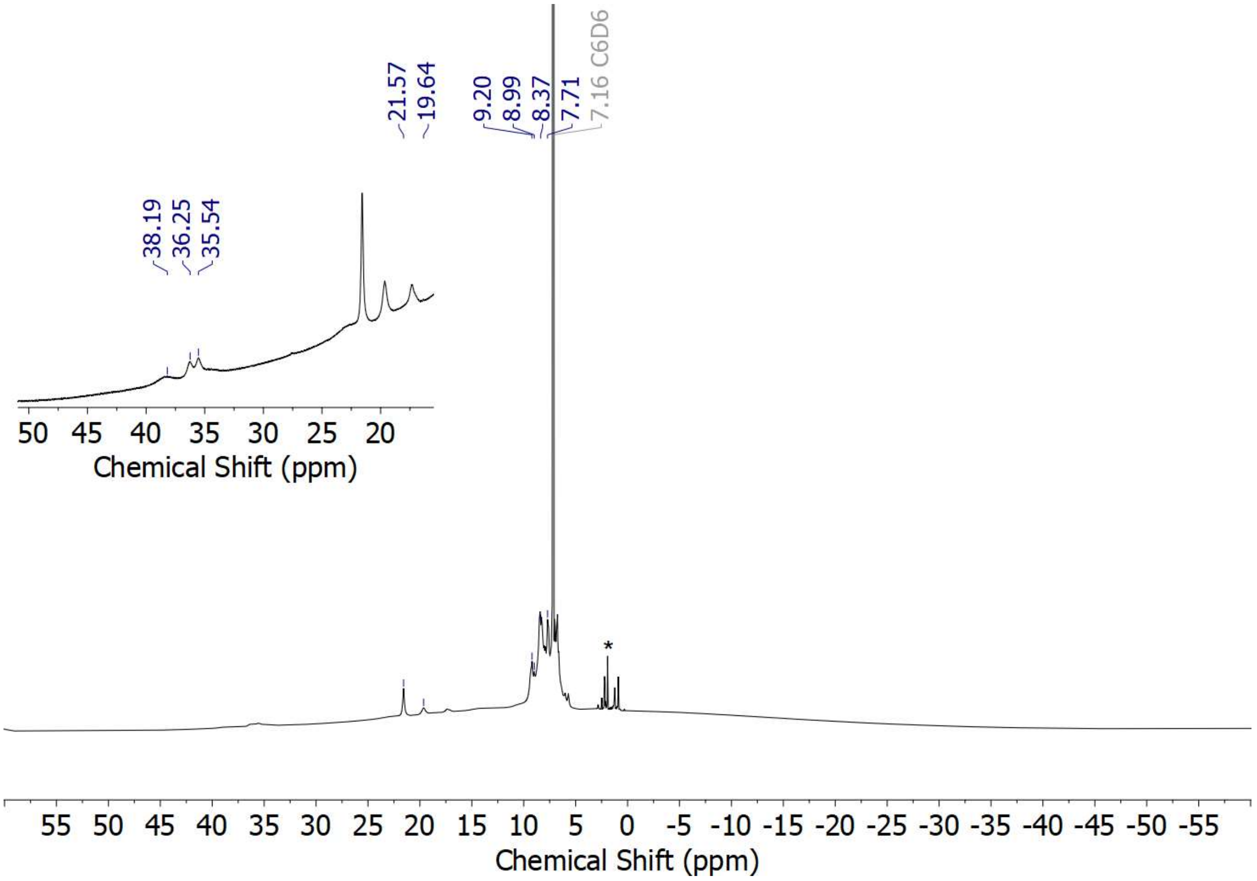

Figure S-47. ^{1}H NMR (600 Hz) spectrum of the reaction of [Fe(^{Ph}Dbf)(NC_5H_5)$_2$] (**5a**) with 2,4,6-trimethylphenyl azide to form a new species (dark red).

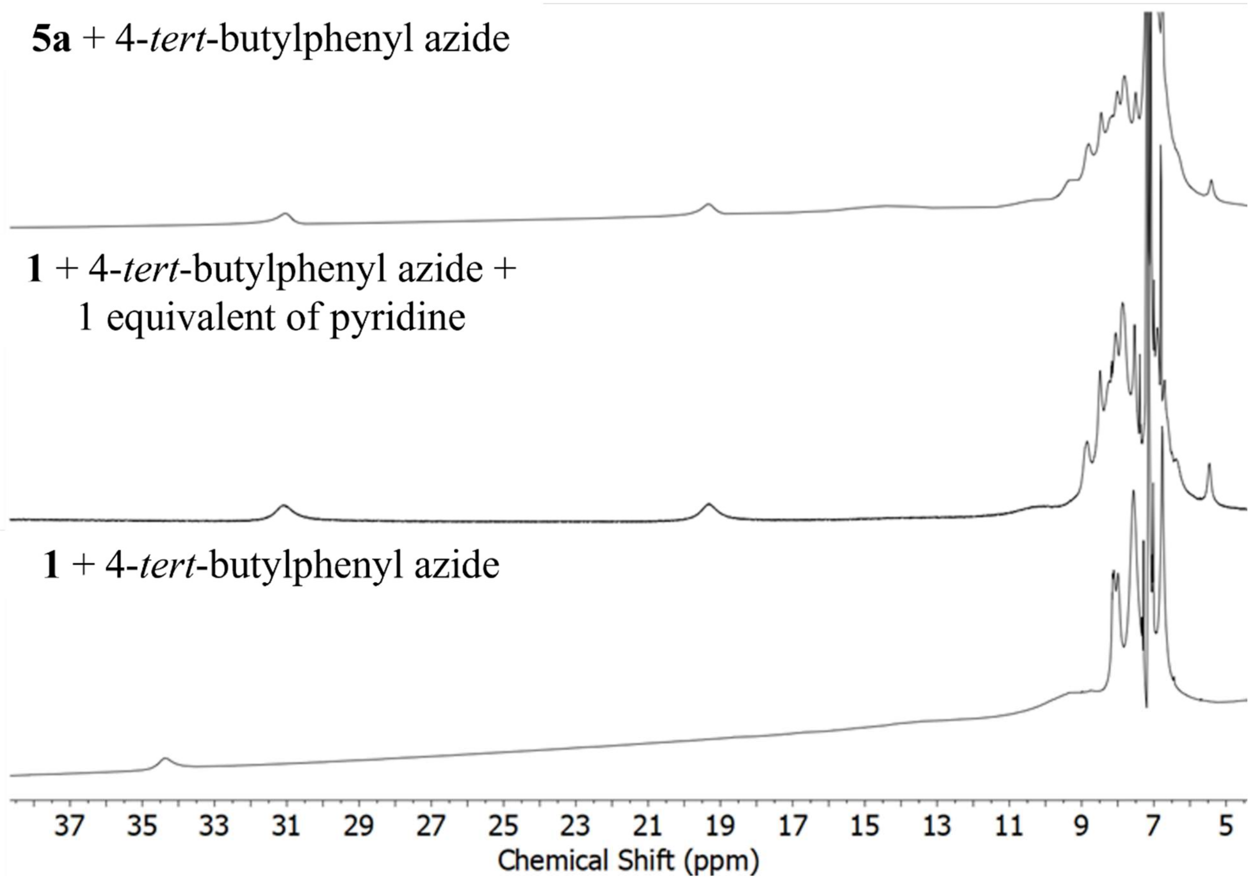

Figure S-48. ^{1}H NMR (600 Hz) spectra of the reaction of **1** and 4-*tert*-butylphenylazide (*bottom*), [Fe_2(^{Ph}Dbf)$_2$(μ–$NC_8H_3F_6$)] (**2b**) and 1 equivalent of pyridine (*middle*) and [Fe(^{Ph}Dbf)(NC_5H_5)$_2$] (**5a**) with 4-*tert*-butylphenyl azide (*top*). Spectra suggests the formation of the same species via both reaction pathways. Spectra are shown from 5 ppm to 40 ppm to eliminate the organic impurity peaks (hexanes/xs. azide) for clarity.

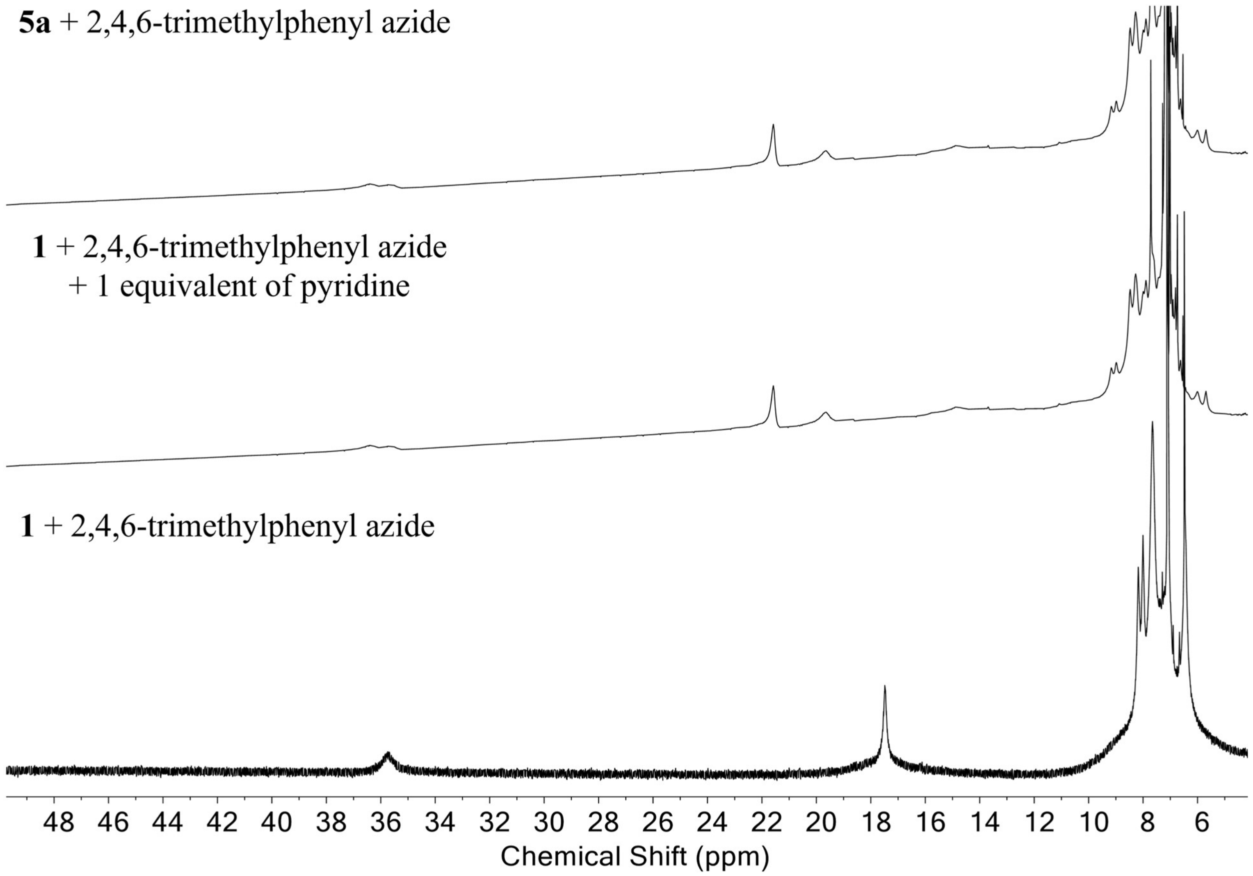

Figure S-49. ^{1}H NMR (600 Hz) spectra of the reaction of **1** and 2,4,6-trimethylphenyl azide (*bottom,* 376 MHz), [Fe_2(^{Ph}Dbf)$_2$(μ–$NC_8H_3F_6$)] (**2b**) and 1 equivalent of pyridine (*middle,* 564 MHz) and [Fe(^{Ph}Dbf)(NC_5H_5)$_2$] (**5a**) with 4-*tert*-butylphenyl azide (*top,* 564 MHz). Spectra suggests the formation of the same species via both reaction pathways to form the pyridine bound diiron imido and this species has a ^{1}H NMR spectrum distinct from the symmetric diiron imido. Upon addition of 2,4,6-trimethylphenyl azide to **1**, the solution needs to sit for 24 hours before any change is noted in the ^{1}H NMR; however, upon addition to 1 eq. of pyridine to that reaction, the reaction mixture turns red slowly over the course of five minutes, indicating complete conversion to the new species. Spectra are shown from 5 ppm to 47 ppm to eliminate the organic impurity peaks (hexanes/xs. azide) for clarity.

Intramolecular dehydrogenation of 2,6-diisopropylphenyl azide reaction

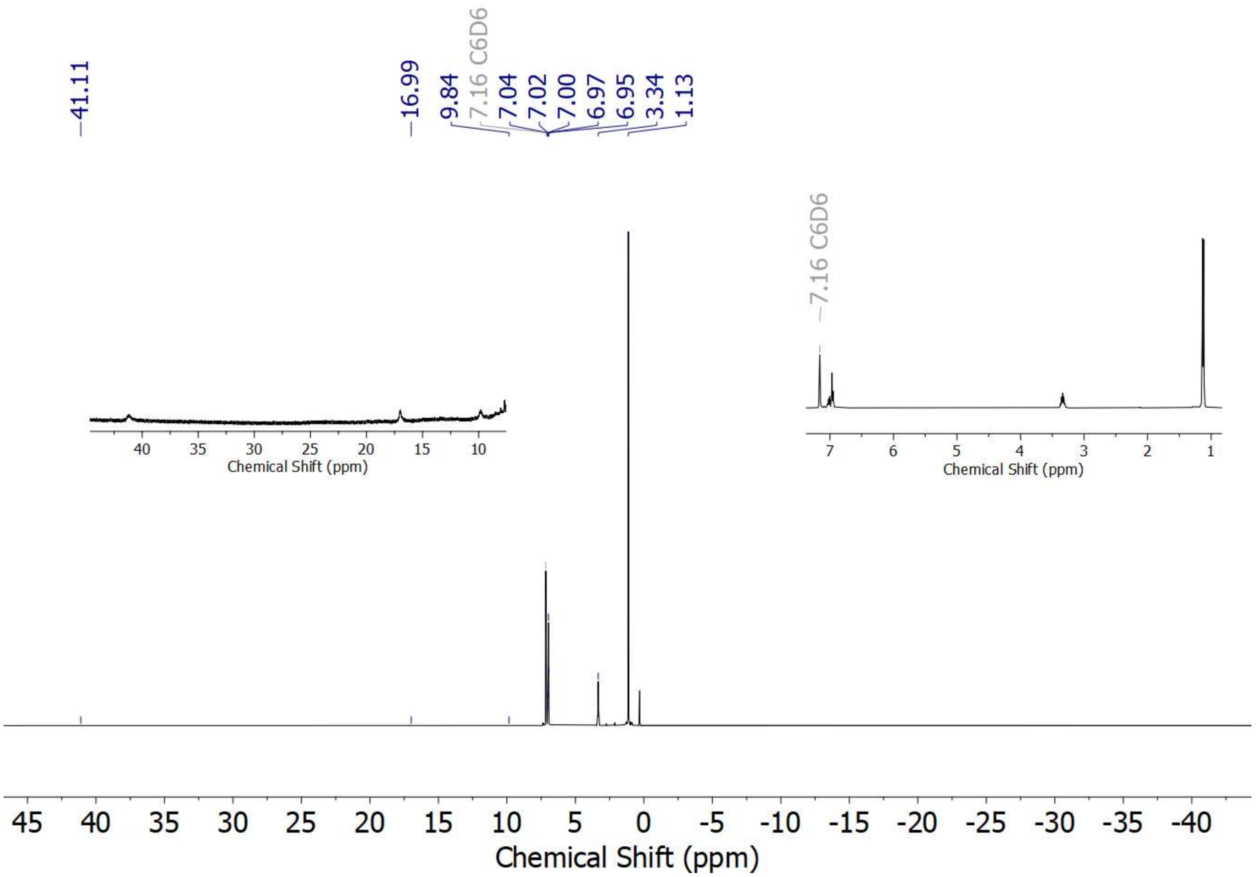

Figure S-50. ^1H NMR (600 MHz) spectrum of the reaction of $[Fe_2(^{Ph}Dbf)_2]$ (**1**) with 10 equivalents 2,6-diisopropylphenyl azide at room temperature. Inlays indicates identifying proton peaks for the starting complex (*left*) and organic starting material (*right*).

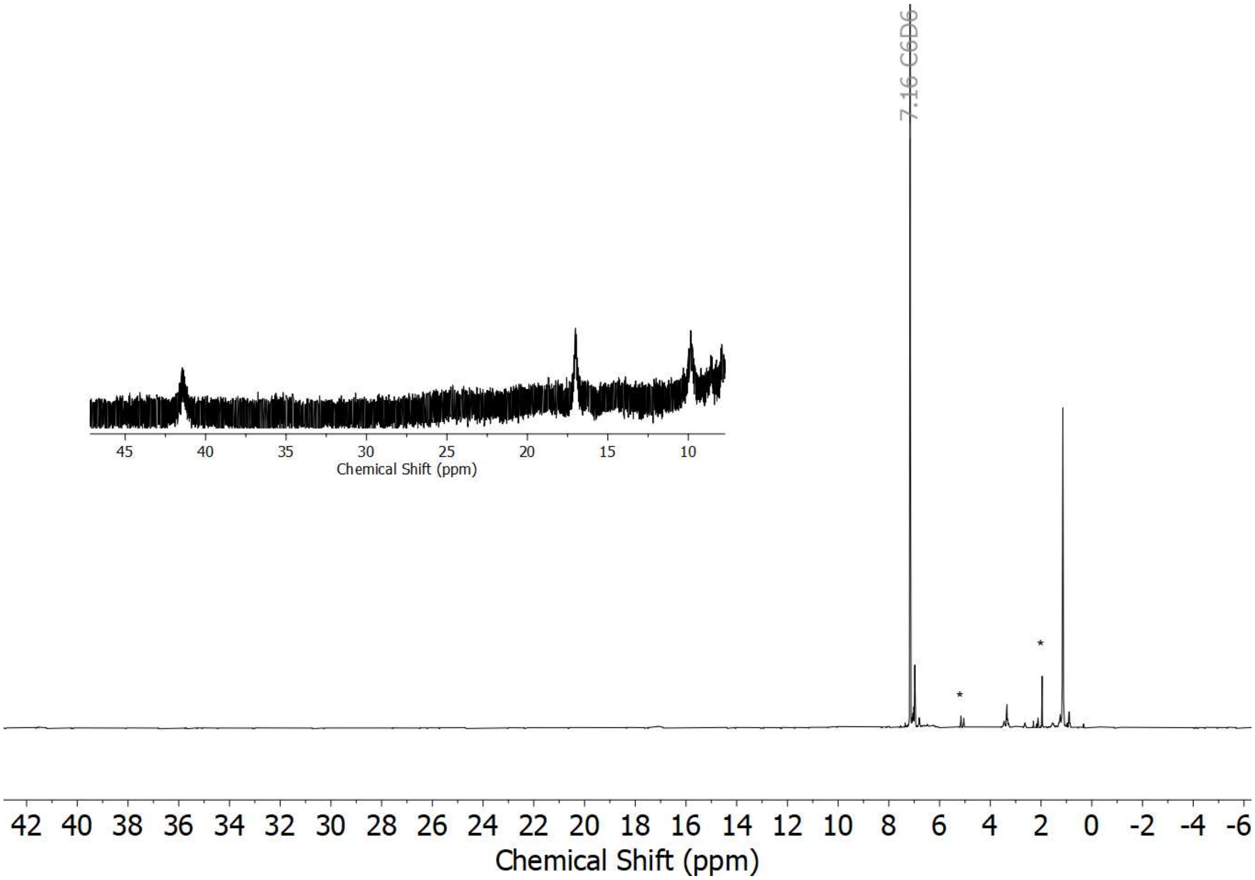

Figure S-51. ^{1}H NMR (600 MHz) spectrum of the reaction of [Fe_2(^{Ph}Dbf)$_2$] (**1**) with 1 equivalent of 2,6-diisopropylphenylazide after 18 hours at 80 ˚C. The inlay of the paramagnetic region indicates presence of the starting material [Fe_2(^{Ph}Dbf)$_2$] (**1**). No new paramagnetic peaks were identified. * Indicates diamagnetic peaks unique to the dehydrogenated product 2-isopropyl-6-(prop-1-en-2-yl)aniline.

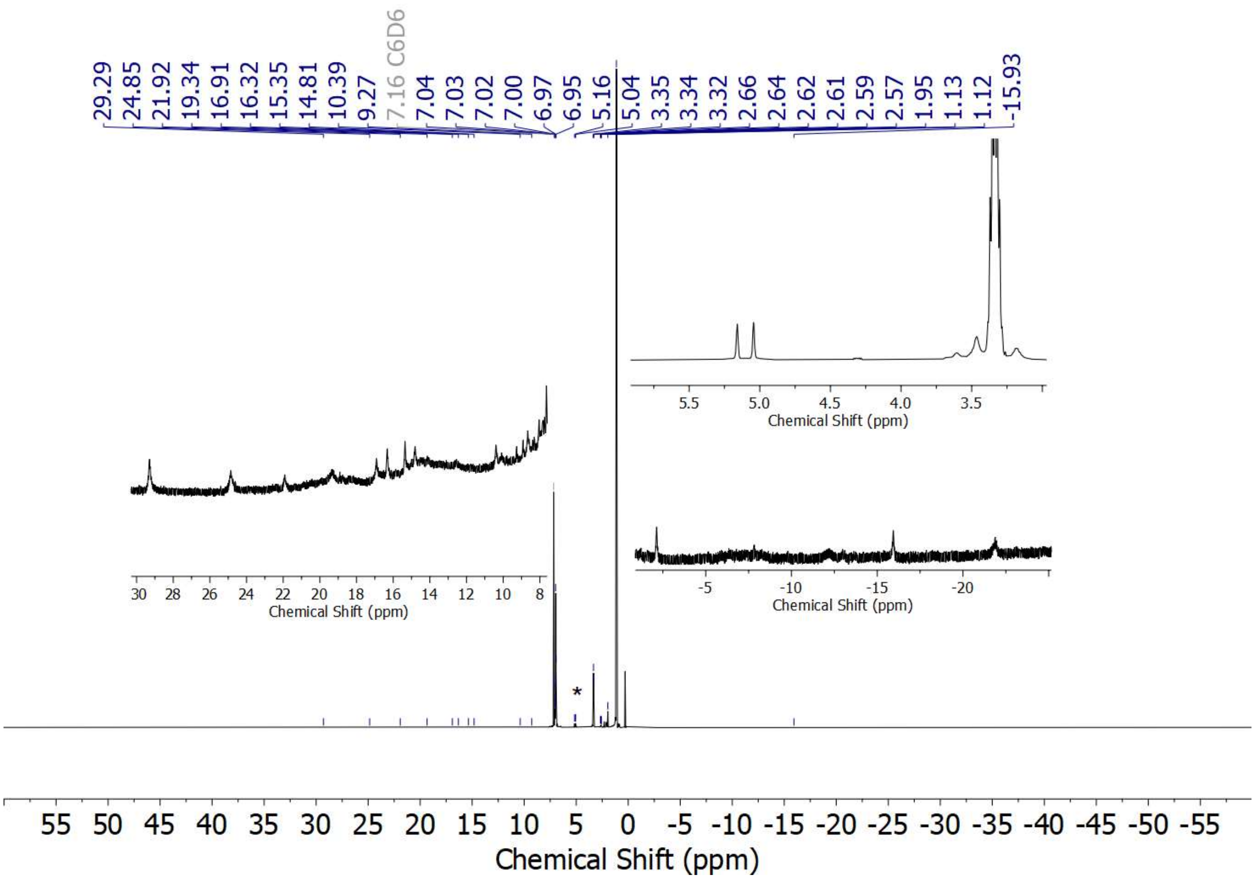

Figure S-52. ^{1}H NMR (600 MHz) spectrum of the initial reaction of [$Fe_2(^{Ph}Dbf)_2$] (**1**) with 10 equivalents 2,6-diisopropylphenyl azide after 24 hours at 80 ˚C. Inlays indicate new paramagnetic peaks (*left*) and organic starting material (*right, bottom*), and the identifying diamagnetic peak for the corresponding olefin (*right, top)* 2-isopropyl-6-(prop-1-en-2-yl)aniline.

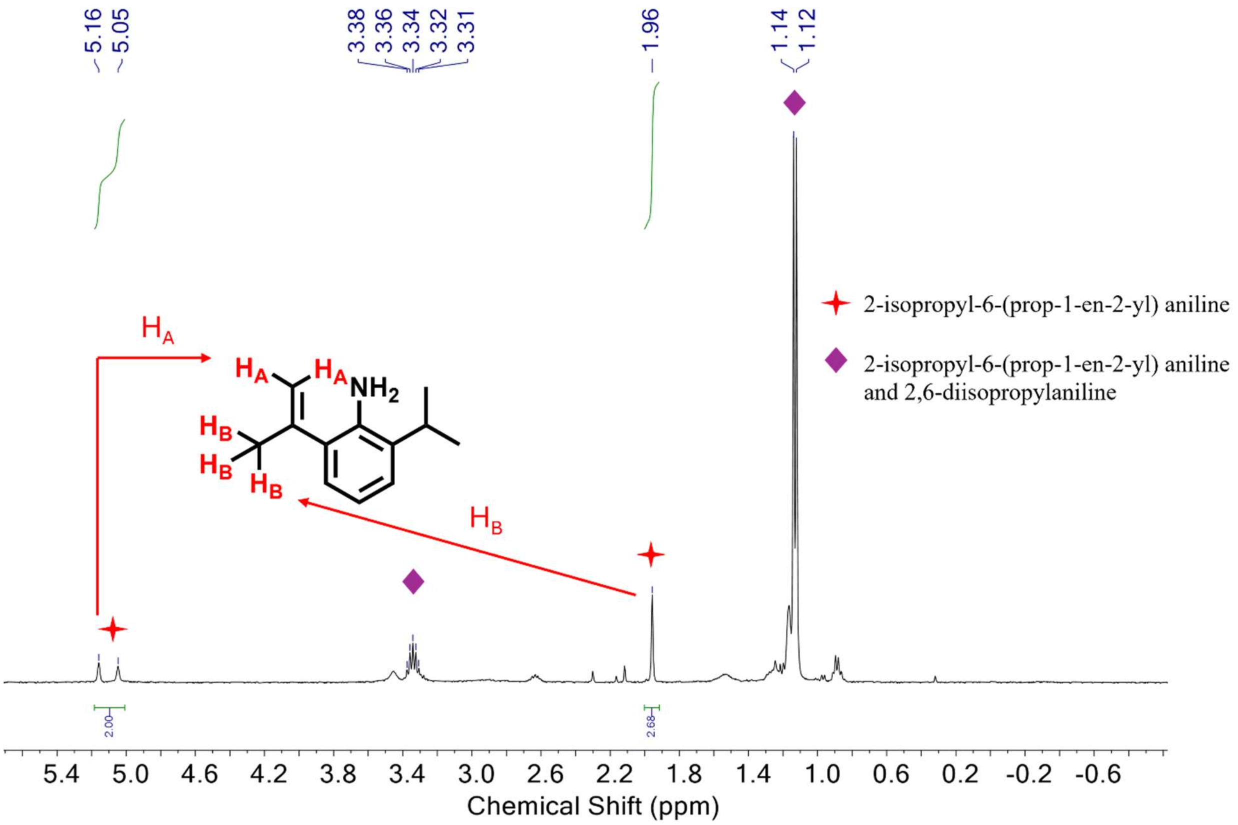

Figure S-53. ^{1}H NMR (600 MHz) spectrum of the organic product 2-isopropyl-6-(prop-1-en-2-yl)aniline resulting from the reaction of $[Fe_2(^{Ph}Dbf)_2]$ (**1**) with 10 equivalents 2,6-diisopropylphenyl azide after 24 hours at 80 °C. Identifying peaks are integrated to 2:3, respectively, as expected for the dehydrogenated isopropyl group.

Styrene aziridination reaction

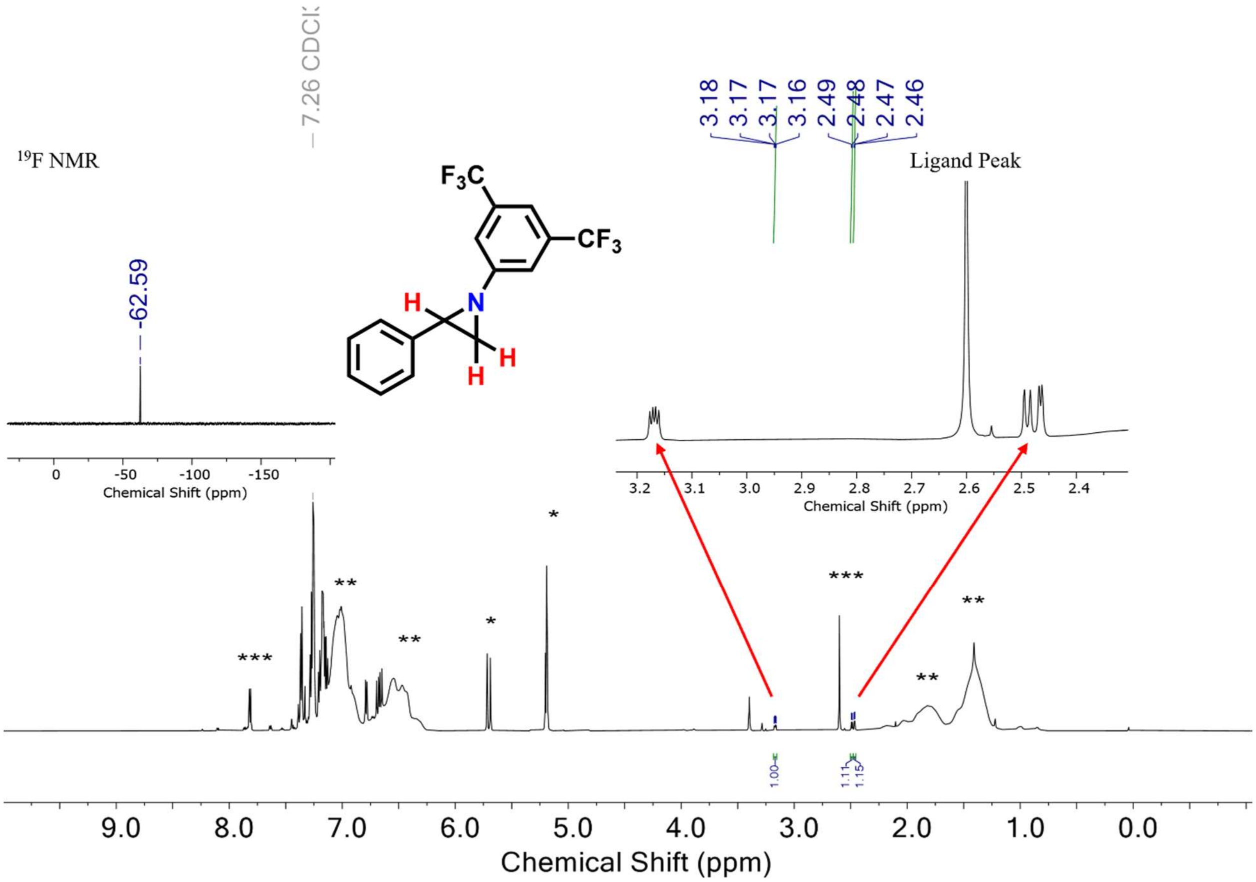

Figure S-54. ^{1}H (600 MHz) and ^{19}F (564 MHz, *inlay left*) NMR spectra of the organic product, 1-(3,5-bis(trifluoromethyl)phenyl-2-phneylaziridine, of the reaction of $[Fe_2(^{Ph}Dbf)_2]$ (**1**) with styrene. The product, 1-(3,5-bis(trifluoromethyl)phenyl-2-phenylaziridine, was identified by the two peaks corresponding to the aziridine protons marked in red (*inlay right*) in addition to the ^{19}F NMR peak, as well as GC-MS. Aromatic peaks are not marked as this compound was not isolated pure. Peaks from the ^{Ph}Dbf ligand (***), excess styrene (*), and polymerized styrene (**) are all marked accordingly.

Reactions with [Fe_2(^{Ph}Dbf)$_2$(μ−$NC_8H_3F_6$)] (2a) with O–H and C–H Bond Substrates

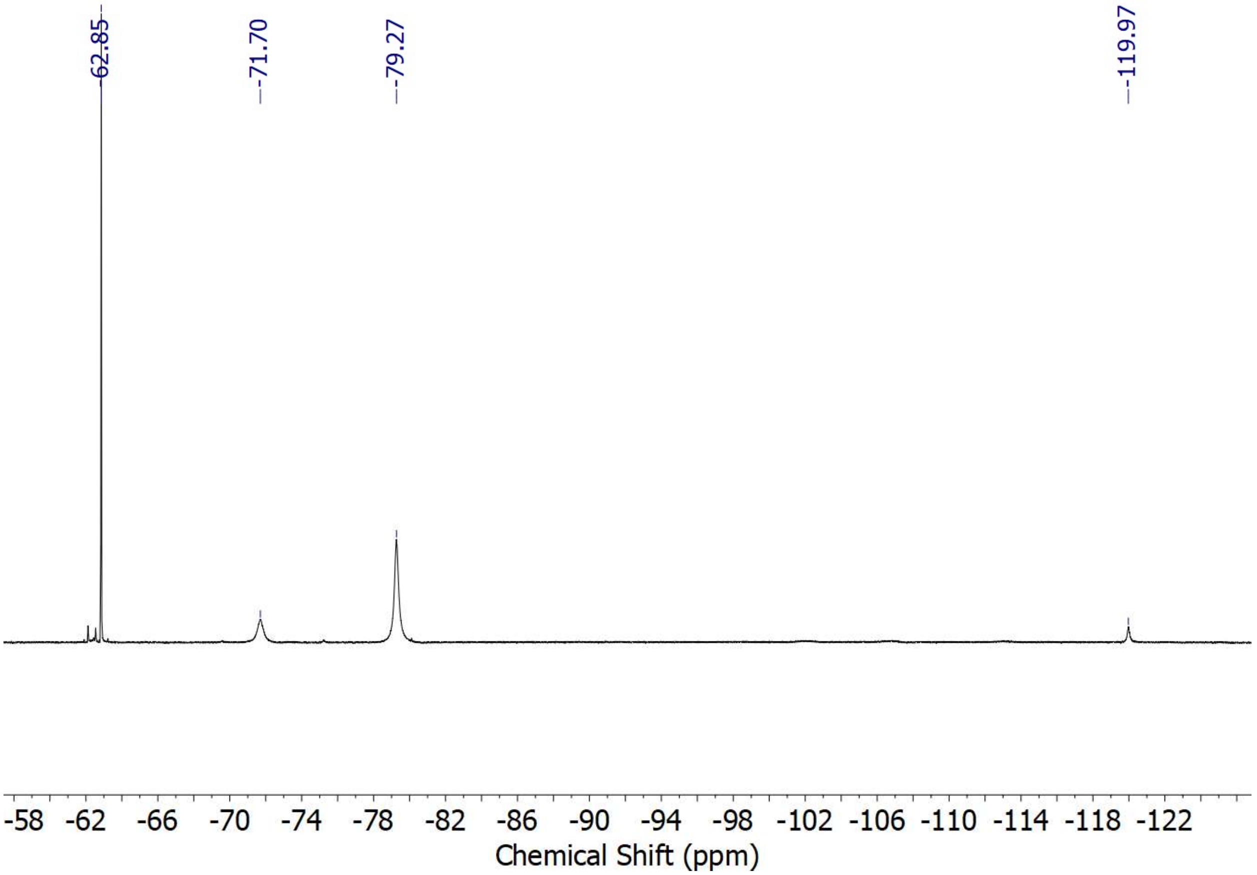

Figure S-55. ^{19}F NMR (564 MHz) spectrum for paramagnetic [Fe_2(^{Ph}Dbf)$_2$(μ−$NC_8H_3F_6$)] (**2a**) reaction with 2-hydroxy-2-azaadaamantane. Peaks at -71.10 ppm and -119.97 ppm represent the product of the imido with 9-azabicyclo[3.3.1]nonane-N-oxyl (see Figure S-56 below) and [Fe_2(^{Ph}Dbf)$_2$(μ−$NHC_8H_3F_6$)($NHC_8H_3F_6$)] (**3**). Peak in the organic region (-62.58 ppm) indicates the formation of aniline upon HAA.

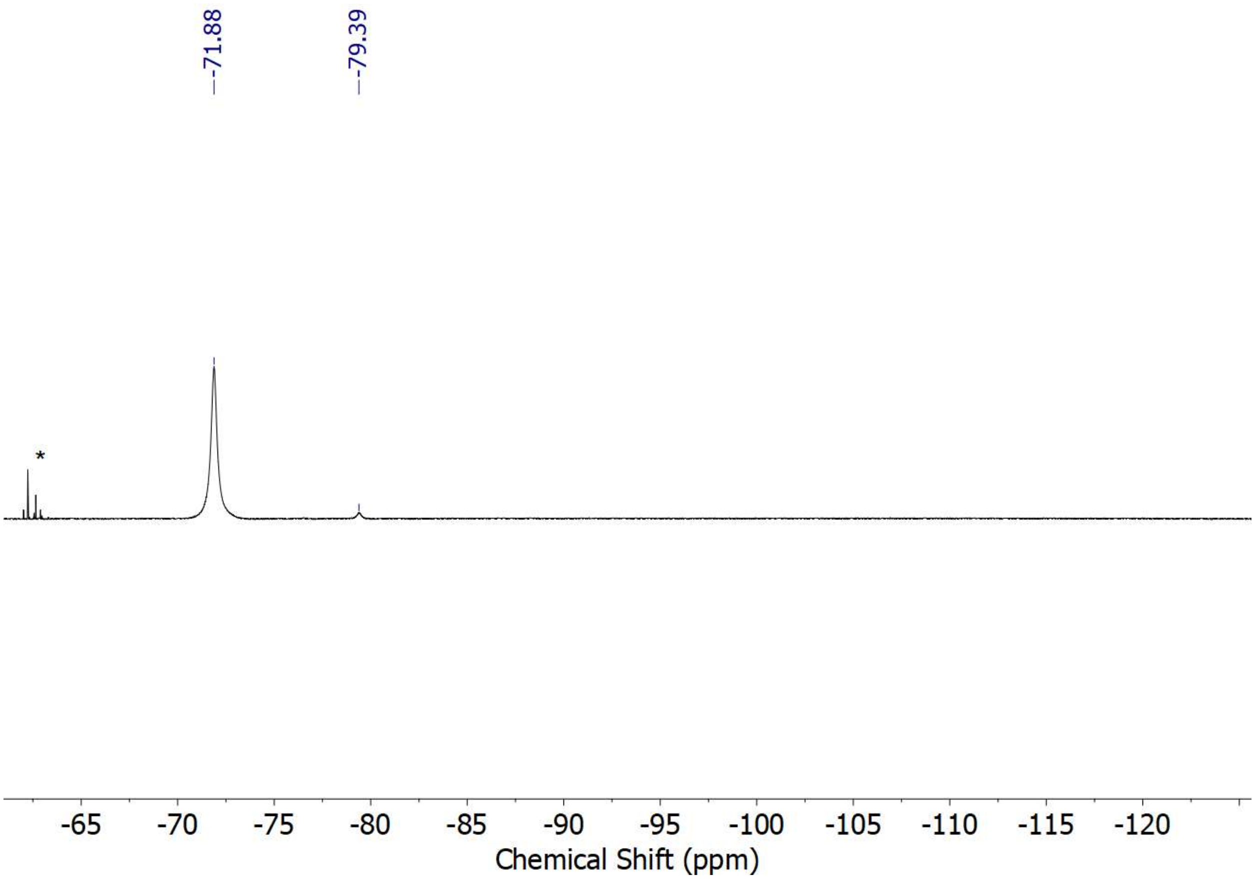

Figure S-56. ^{19}F NMR (564 MHz) spectrum for paramagnetic $[Fe_2(^{Ph}Dbf)_2(\mu{-}NC_8H_3F_6)]$ (**2a**) reaction with 9-azabicyclo[3.3.1]nonane-N-oxyl at room temperature. Peak at -71.88 ppm represents the radical bound imido species and peak at -79.39 ppm represents minor unconverted imido complex (< 1%).   * indicates minor organic impurity.

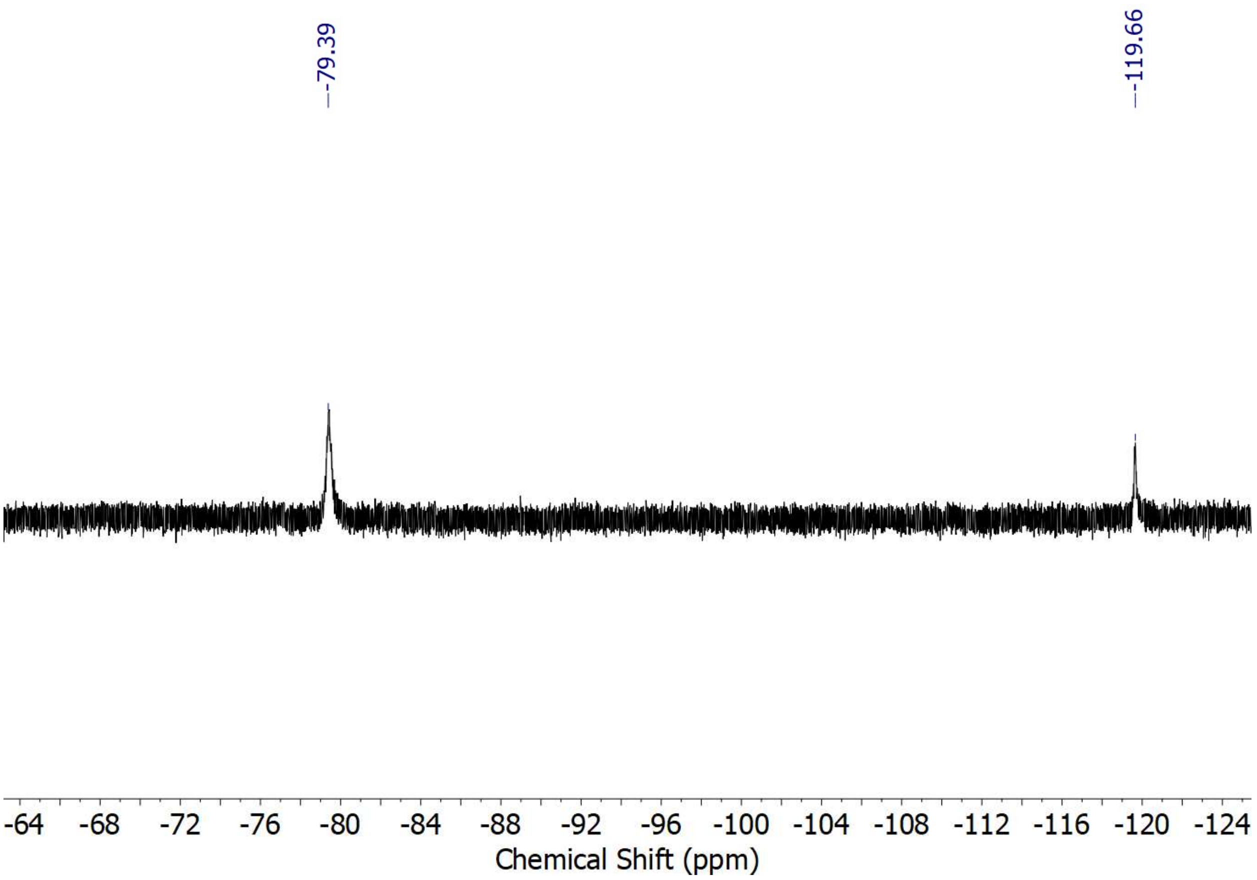

Figure S-57. ^{19}F NMR (376 MHz) spectrum for paramagnetic $[Fe_2(^{Ph}Dbf)_2(\mu{-}NC_8H_3F_6)]$ (**2a**) reaction with 2,4,6-tri-*tert*-butylphenol overnight at 80 °C. Formation of $[Fe_2(^{Ph}Dbf)_2(\mu{-}NHC_8H_3F_6)(NHC_8H_3F_6)]$ (**3**) is observed.

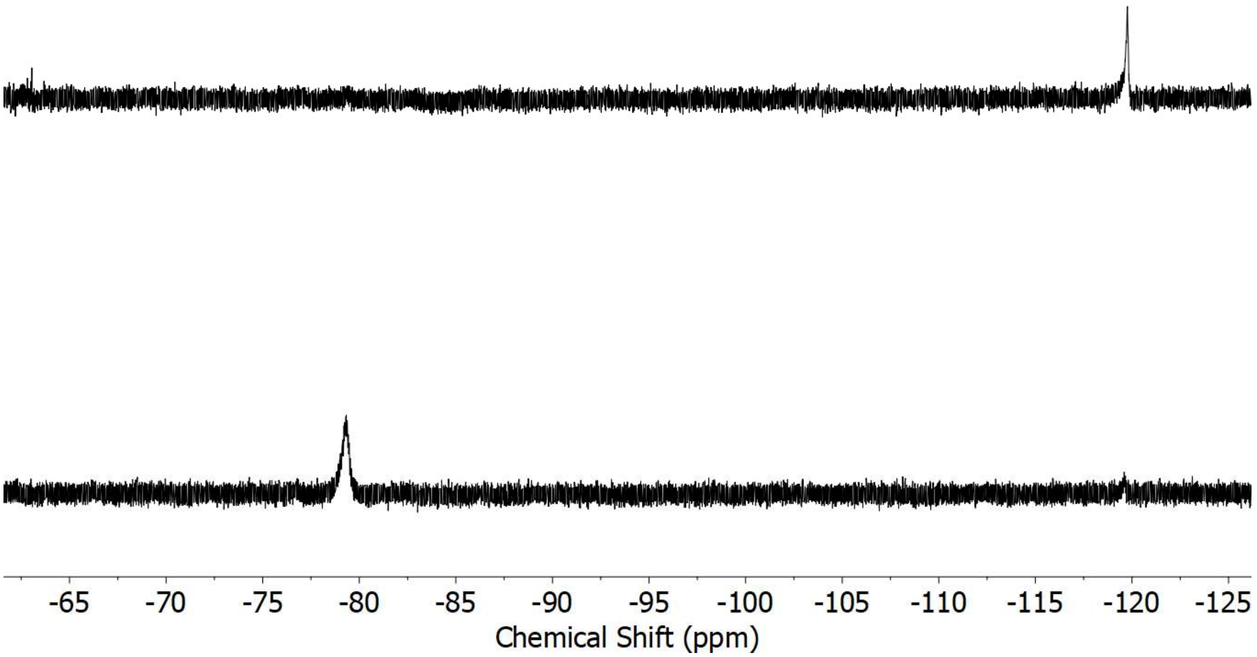

Figure S-58. ^{19}F NMR (376 MHz) spectra for paramagnetic [$Fe_2(^{Ph}Dbf)_2(\mu{-}NC_8H_3F_6)$] (**2a**) (bottom) reaction with 1,4-cyclohexadiene for 5 hours at 80 ˚C (10 mg scale). Complete conversion to [$Fe_2(^{Ph}Dbf)_2(\mu{-}NHC_8H_3F_6)(NHC_8H_3F_6)$] (**3**) is observed (*top*).

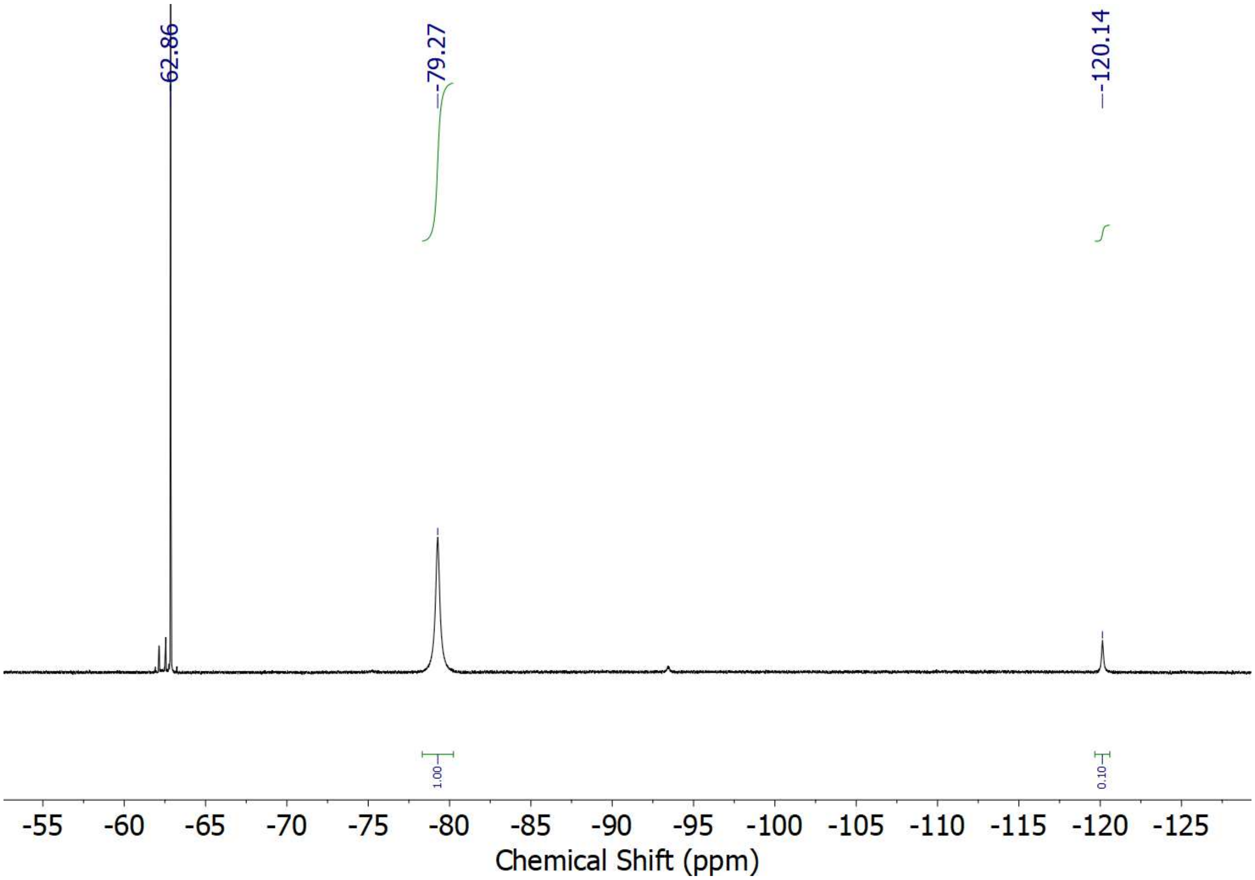

Figure S-59. ^{19}F NMR (565 MHz) spectrum for paramagnetic $[Fe_2(^{Ph}Dbf)_2(\mu{-}NC_8H_3F_6)]$ (**2a**) reaction with *9H*-fluorene overnight at 80 ˚C. Formation of $[Fe_2(^{Ph}Dbf)_2(\mu{-}NHC_8H_3F_6)(NHC_8H_3F_6)]$ (**3**) is observed. We propose this is due to the steric bulk of the substrate. The peak at -62.28 ppm is excess 3,5-bis(trifluoromethyl)phenyl azide.

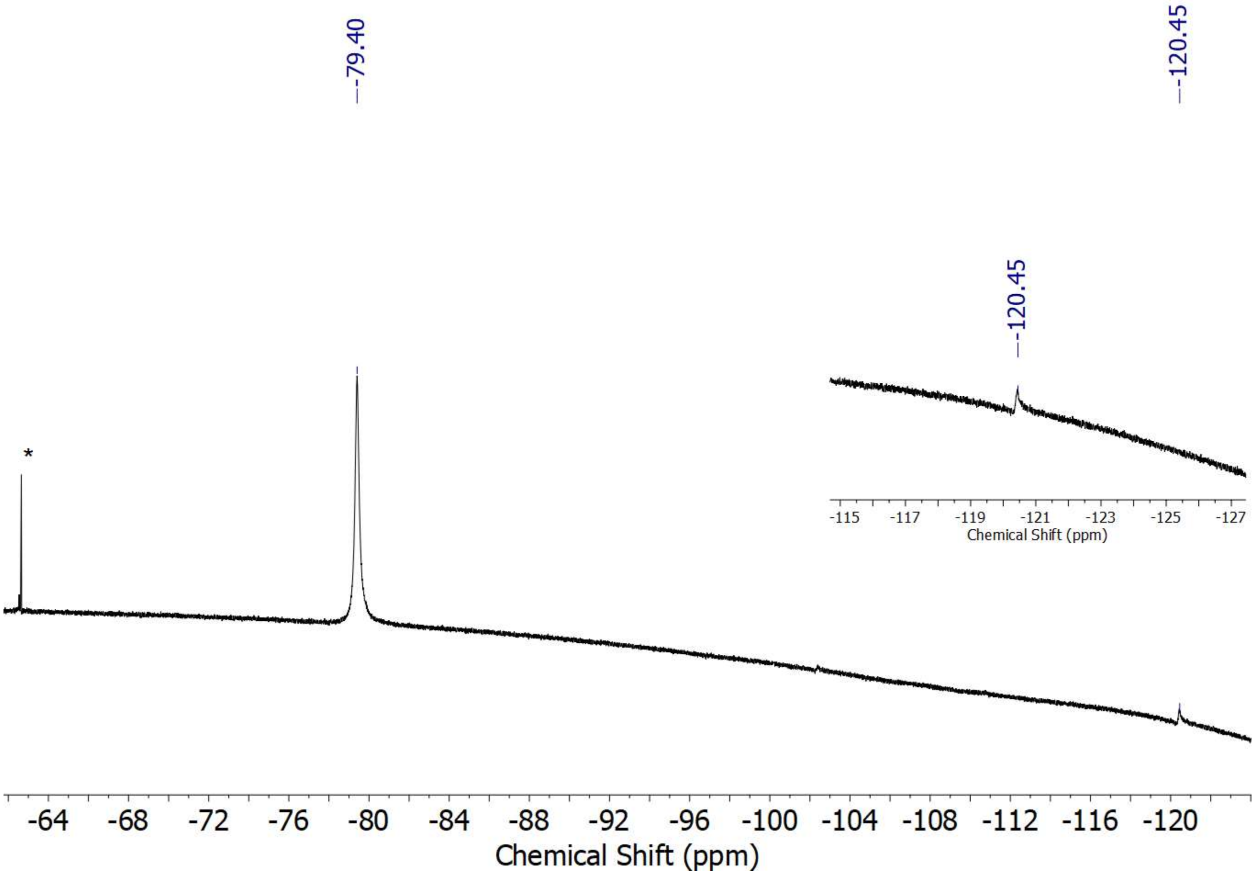

Figure S-60. ^{19}F NMR (565 MHz) spectrum for paramagnetic [Fe_2(^{Ph}Dbf)$_2$(μ−$NC_8H_3F_6$)] (**2a**) reaction with triphenylmethane (BDE = 79.0 Kcal/mol)[3] over 48 hours at 80 ˚C. Formation of [Fe_2(^{Ph}Dbf)$_2$(μ−$NHC_8H_3F_6$)($NHC_8H_3F_6$)] (**3**) is observed in very small amounts. We propose this is due to the steric bulk of the substrate. The impurity indicated by * is excess 3,5-bis(trifluormethyl)phenyl azide.

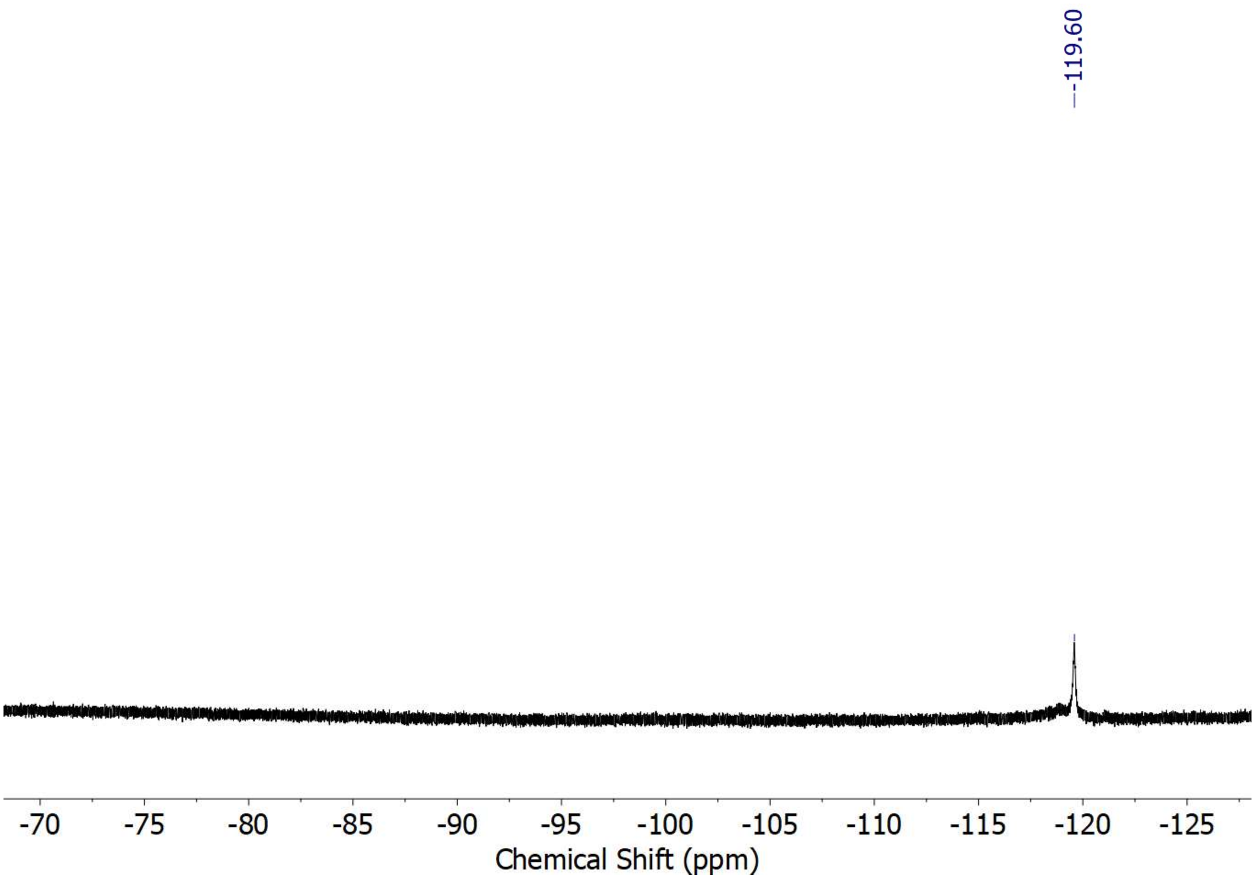

Figure S-61. ^{19}F NMR (376 MHz) spectrum (*unlocked in toluene*) for paramagnetic $[Fe_2(^{Ph}Dbf)_2(\mu{-}NC_8H_3F_6)]$ (**2a**) reaction with toluene at room temperature. after 1 hour. Formation of $[Fe_2(^{Ph}Dbf)_2(\mu{-}NHC_8H_3F_6)(NHC_8H_3F_6)]$ (**3**) is observed. No significant organic product is formed after extended periods of time.

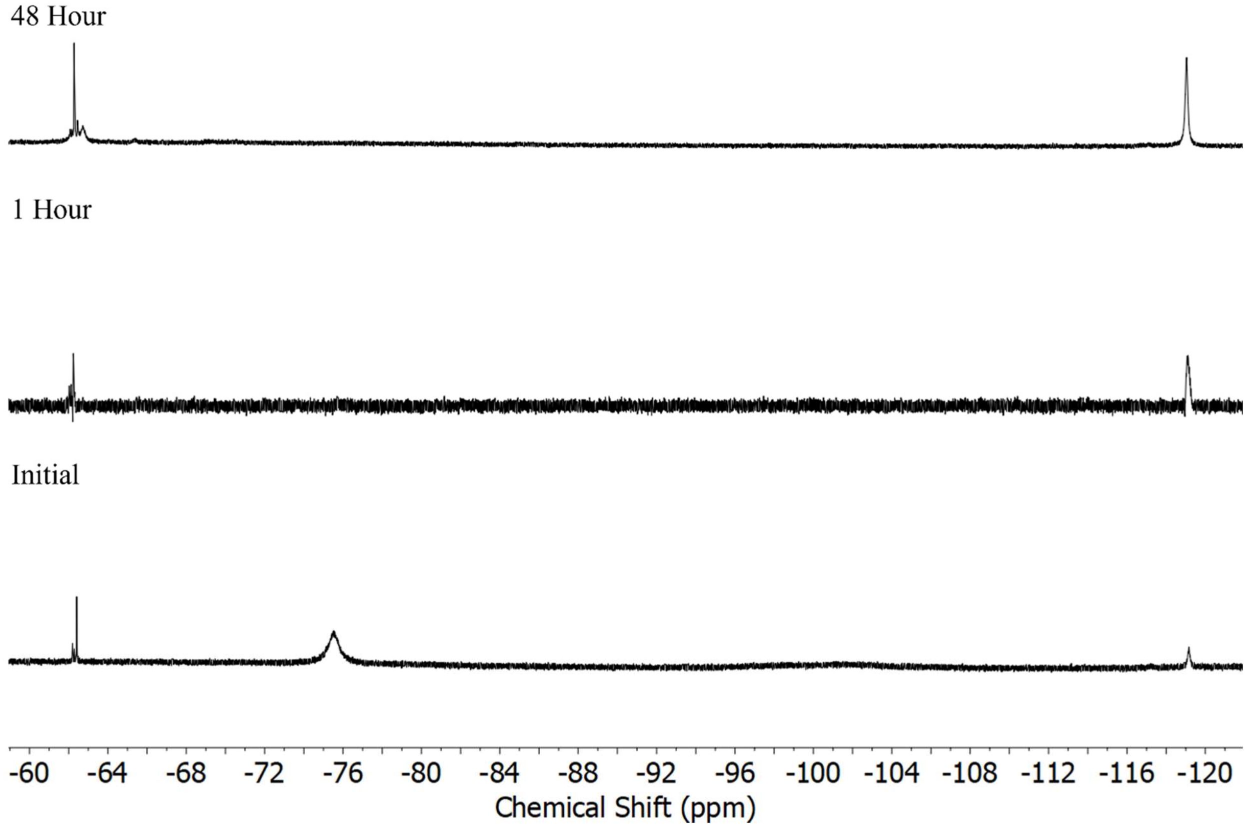

Figure S-62. Stacked ^{19}F NMR (376 MHz) spectra (*unlocked in toluene*) for paramagnetic $[Fe_2(^{Ph}Dbf)_2(\mu{-}NC_8H_3F_6)]$ (**2a**) reaction with toluene at 80 ˚C over 72 hours. Formation of $[Fe_2(^{Ph}Dbf)_2(\mu{-}NHC_8H_3F_6)(NHC_8H_3F_6)]$ (**3**) is observed. No significant organic product is formed as reaction is heated after the initial formation of $[Fe_2(^{Ph}Dbf)_2(\mu{-}NHC_8H_3F_6)(NHC_8H_3F_6)]$ (**3**). Aniline and bibenzyl were identified in GC-MS as organic products, suggesting HAA from toluene, but only trace amount of aminated product (*N*-benzyl-3-5-bis(trifluoromethyl)aniline) was observed.

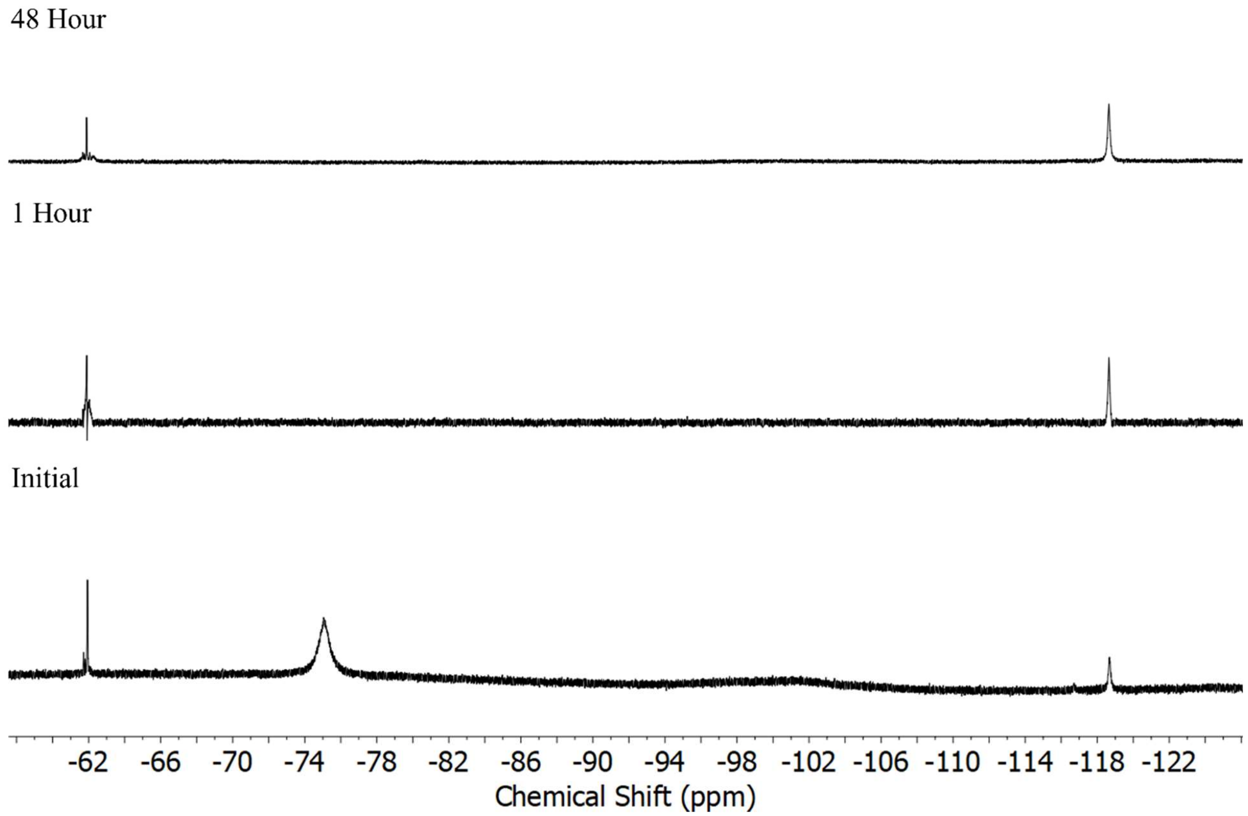

Figure S-63. Stacked ^{19}F NMR (376 MHz) spectra (*unlocked in toluene*) for paramagnetic $[Fe_2(^{Ph}Dbf)_2(\mu{-}NC_8H_3F_6)]$ (**2a**) reaction with toluene at 100 °C over 72 hours. Formation of $[Fe_2(^{Ph}Dbf)_2(\mu{-}NHC_8H_3F_6)(NHC_8H_3F_6)]$ (**3**) is observed. No significant organic product is formed as reaction is heated after the initial formation of $[Fe_2(^{Ph}Dbf)_2(\mu{-}NHC_8H_3F_6)(NHC_8H_3F_6)]$ (**3**). Aniline and bibenzyl were identified in GC-MS as organic products, suggesting HAA from toluene, but only trace amount of aminated product (*N*-benzyl-3-5-bis(trifluoromethyl)aniline) was observed.

Reactions with [Fe_2(^{Ph}Dbf)$_2$(μ−$NC_8H_3F_6$)(NC_5H_5)] (6a) and [Fe_2(^{Ph}Dbf)$_2$(μ−$NC_8H_3F_6$)($NC_6H_5F_3$)] (6b) with C−H Bond Containing Substrates

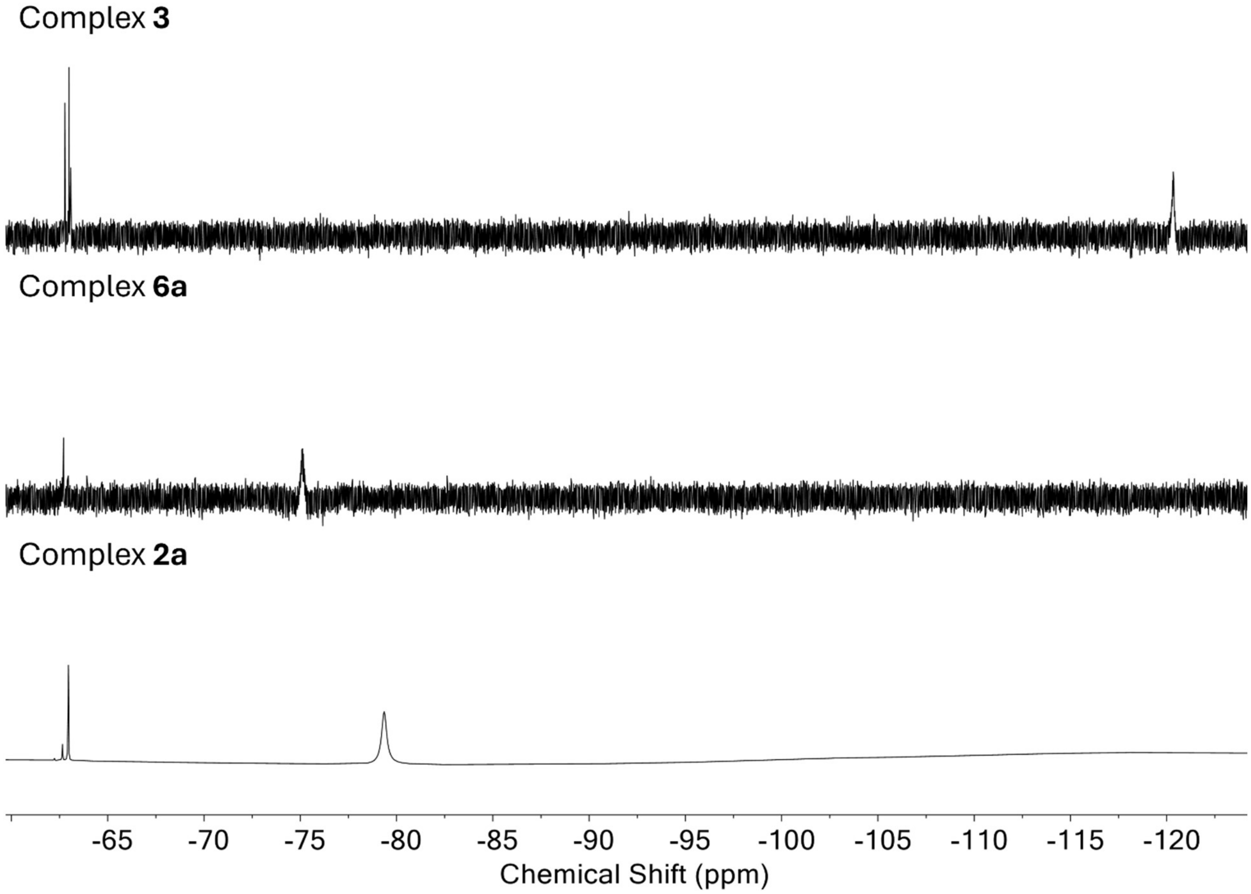

Figure S-64. ^{1}H (600 MHz) and ^{19}F (564 MHz, *inlay*) NMR spectra of the organic product of the reaction of Fe_2(^{Ph}Dbf)$_2$(μ−$NC_8H_3F_6$)(NC_5H_5)] (**6a**) reaction with 1,4-cyclohexadiene at 80 ˚C overnight. Complex **6a** (*middle*) was formed via the addition of 1 equivalent of pyridine to complex **2a** (*bottom*) Complex **3** (*top*) is observed to form over time when **6a** is heated in the presence of 1,4-cyclohexadiene. Peaks in the organic region are excess azide (<2%) and minor organic product (3,5-bis(trifluoromethyl)aniline) forming.

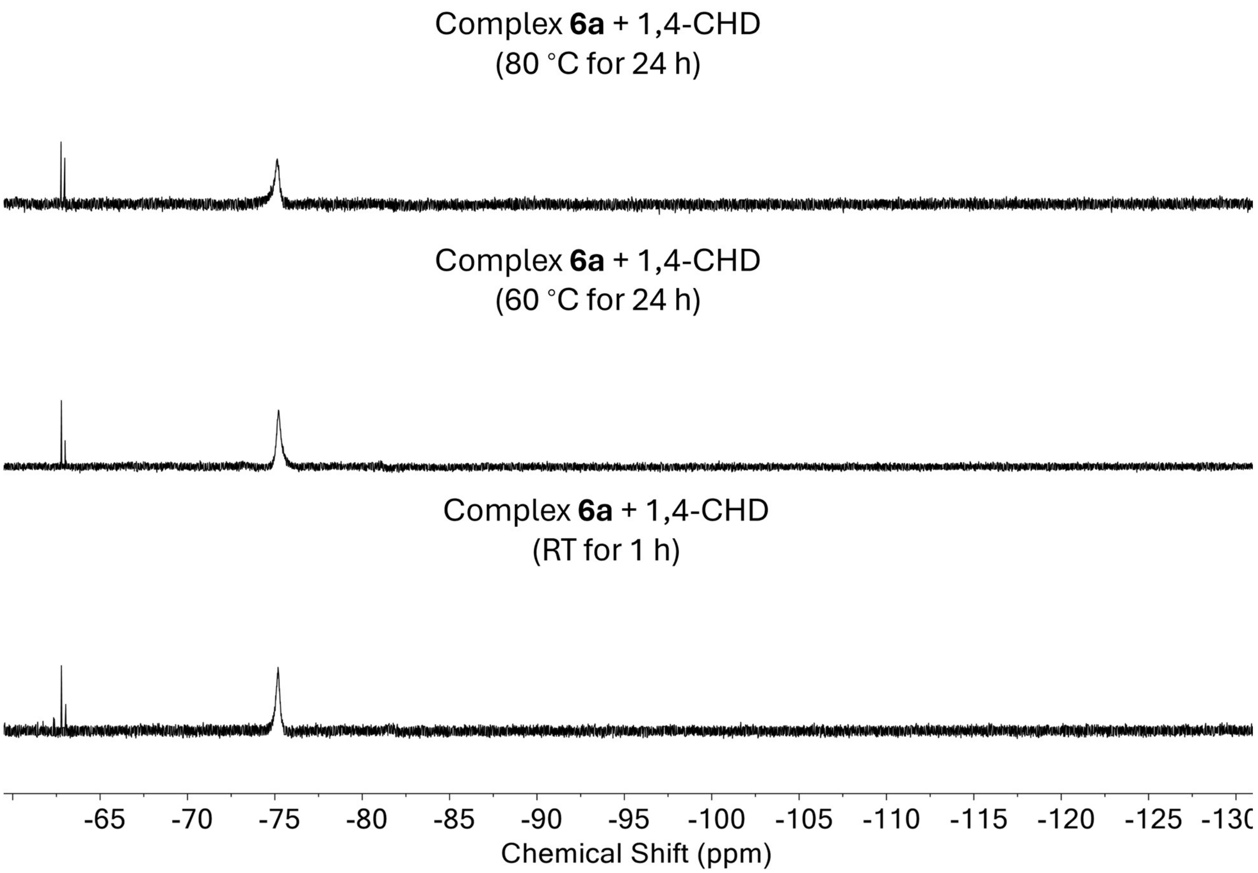

Figure S-65. ^{19}F NMR (376 MHz (*bottom*) and 564 MHz; C_6D_6) spectra of the reaction of $[Fe_2(^{Ph}Dbf)_2(\mu{-}NC_8H_3F_6)(NC_5H_5)]$ (**6a**) reaction with 1,4-cyclohexadiene at room temperature for 1h, 60 ˚C overnight, and 80 ˚C overnight. Complex **6a** remains stable at all temperatures under 90 ˚C.

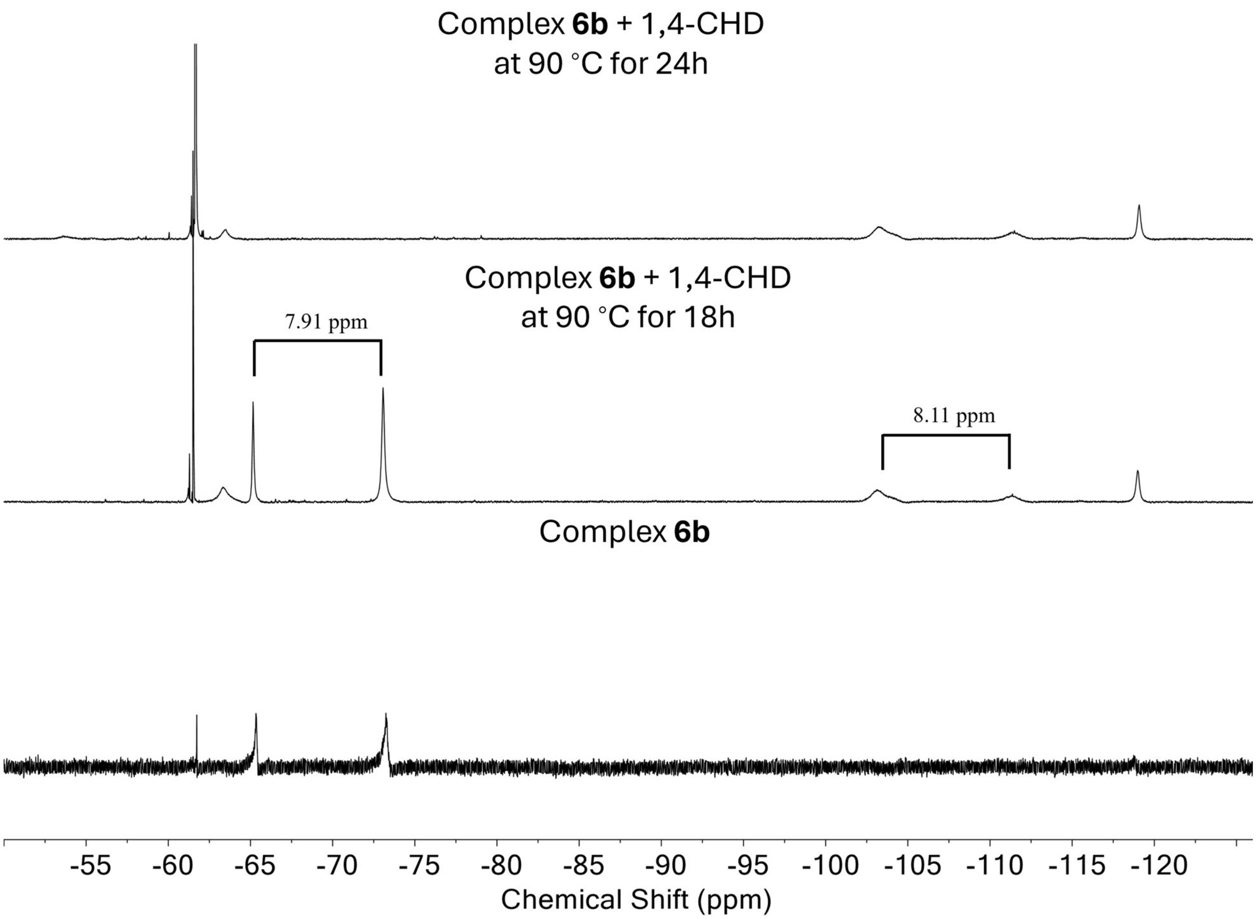

Figure S-66. ^{19}F NMR (376 MHz (*bottom*) and 564 MHz; C_6D_6) spectra of the reaction of $[Fe_2(^{Ph}Dbf)_2(\mu{-}NC_8H_3F_6)(NC_6H_5F_3)]$ (**6b**) reaction with 1,4-cyclohexadiene at 90 ˚C. Complex $[Fe_2(^{Ph}Dbf)_2(\mu{-}NC_8H_3F_6)(NC_6H_4F_3)]$ **6b** (*bottom*) was formed via the addition of 1 equivalent of pyridine to complex **2a** (*bottom*) and heated at 90 ˚C for 18 hours. Complex **3** (*middle*) is observed to form over time along with free pyridine at -64 ppm. Additionally, two peaks are observed, likely corresponding to the same species (CF_3 groups on both the aryl imido and pyridine ligands – 2 peaks expected akin to **6b**). The first of the two peaks is reminiscent in shape and chemical shift (-103.16 ppm) of that of complex $[Fe_2(^{Ph}Dbf)_2(\mu{-}NC_8H_3F_6)(OC_{19}H_{15})]$ **4**, suggesting this may be the elusive proposed active species, the asymmetric bridging amide complex with one bound 4-trifluormethylpyridine ligand represented by the peak at ~ -111.33 ppm. After 24 hours, complex **6b** is consumed (*top*), although the new paramagnetic species, complex **3**, and free pyridine are all observed. Peaks in the organic region correspond to the organic product (3,5-bis(trifluoromethyl)aniline) forming.

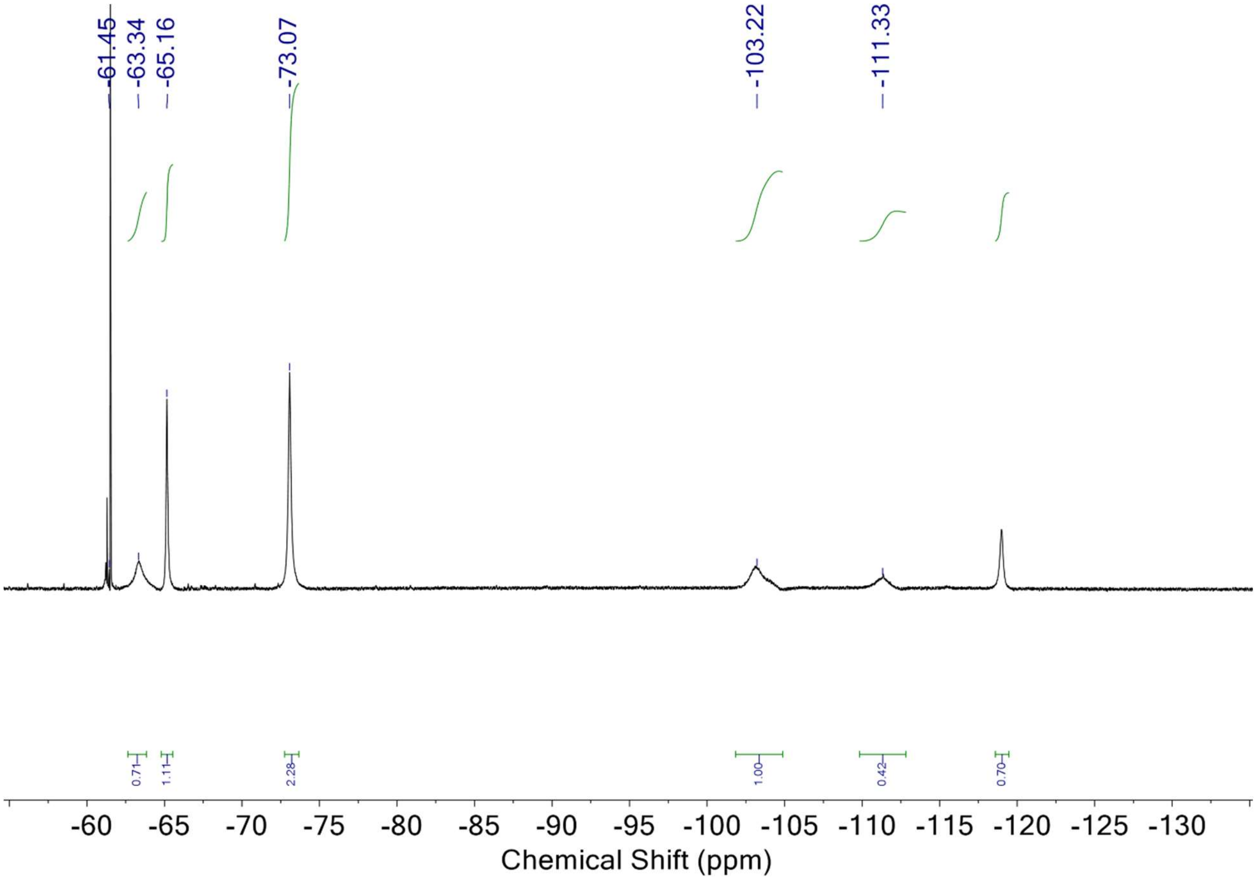

Figure S-67. ^{19}F NMR (564 MHz; C_6D_6) spectrum of the reaction of $[Fe_2(^{Ph}Dbf)_2(\mu{-}NC_8H_3F_6)(NC_6H_4F_3)]$ (**6b**) reaction with 1,4-cyclohexadiene at 90 ˚C after 24 hours. The integration of the peaks report a 1:1 ratio of free pyridine to bis(amide) complex **3**, a 2:1 ratio expected for complex **6b**, and a 2:1 ratio of the new paramagnetic peaks at -103.22 ppm and -111.33 ppm. Peak in the organic region correspond to the organic product (3,5-bis(trifluoromethyl)aniline) forming.

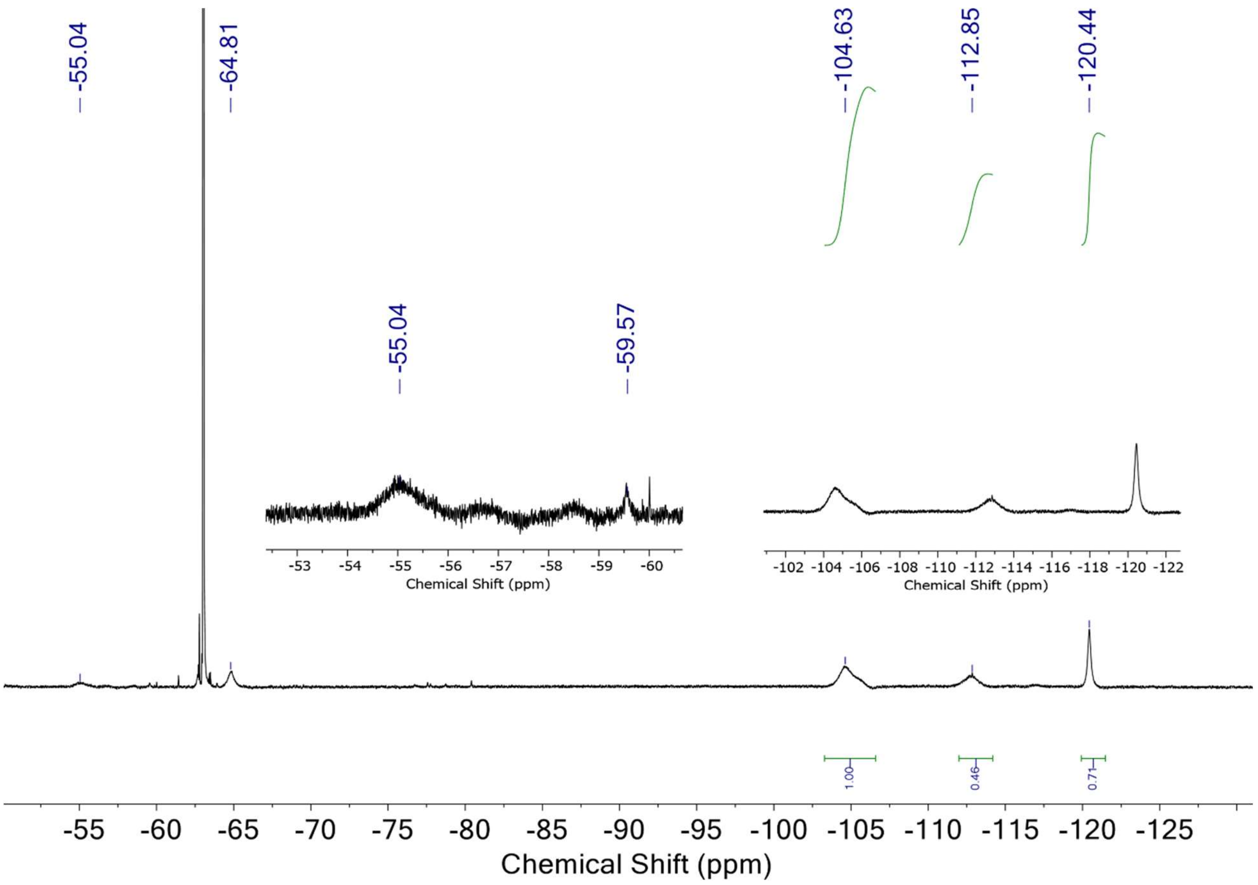

Figure S-68. ^{19}F NMR (564 MHz; C_6D_6) spectrum of the reaction of [Fe_2(^{Ph}Dbf)$_2$(μ−$NC_8H_3F_6$)($NC_6H_4F_3$)] (**6b**) reaction with 1,4-cyclohexadiene at 90 ˚C after 36 hours. Notably, after consumption of complex **6b**, the unidentified paramagnetic species, complex **3**, and free 4-trifluormethylpyrifine are all that remain in solution. A new paramagnetic species can be noted at -55.04 ppm but is not identified. This species is not present in the NMR at 24h (Figure S68 and 69).

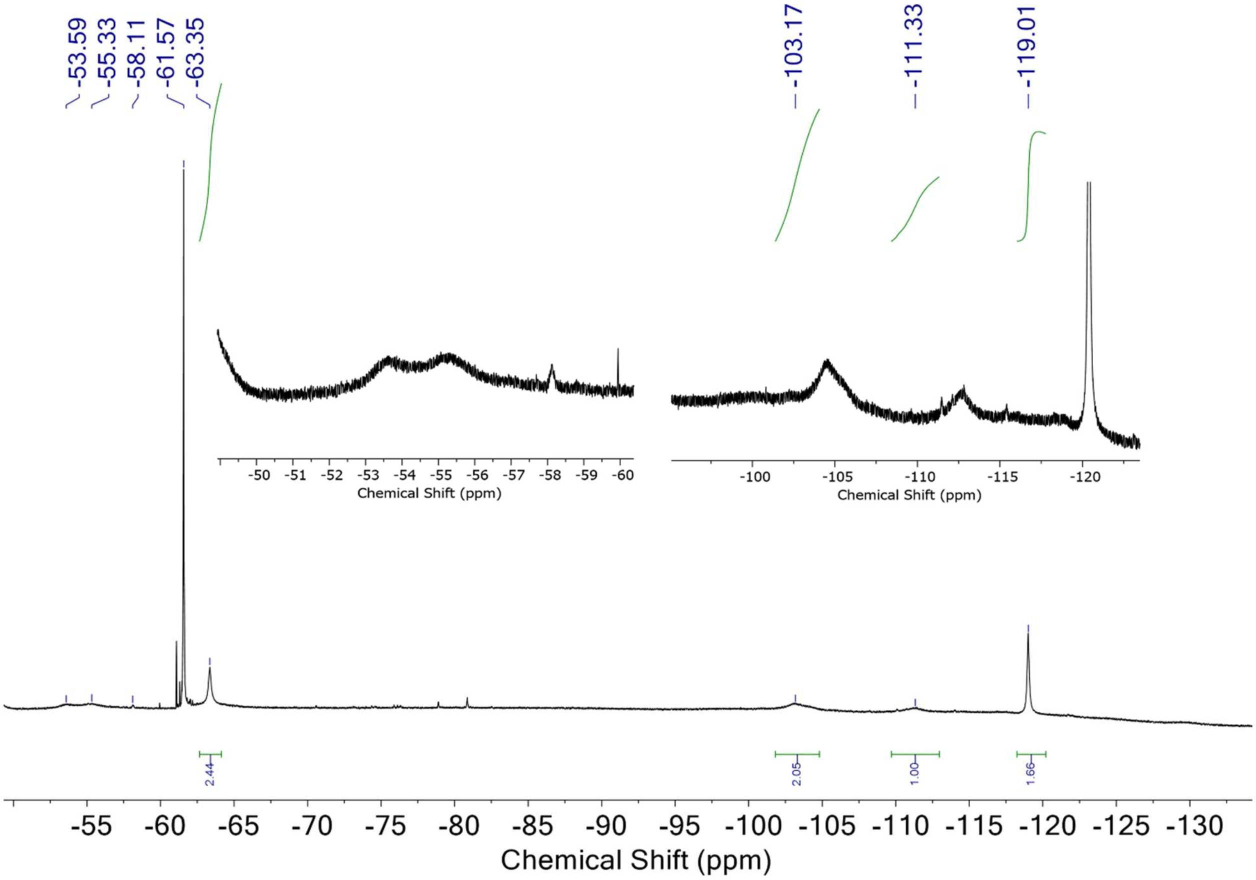

Figure S-69. ^{19}F NMR (564 MHz; C_6D_6) spectrum of the reaction of $[Fe_2(^{Ph}Dbf)_2(\mu{-}NHC_8H_3F_6)(NHC_6H_5F_3)]$ (**3**) reaction with 0.9 equivalents of 4-trifluoromethylpyridine. Notably, the same unidentified paramagnetic species, complex **3**, and free 4-trifluormethylpyrifine remain in solution. Additionally, a new paramagnetic species is generated at -55 ppm, although this species is observed to form after the proposed pyridine bound bridging amide species forms, as seen in **Figure S-70**.

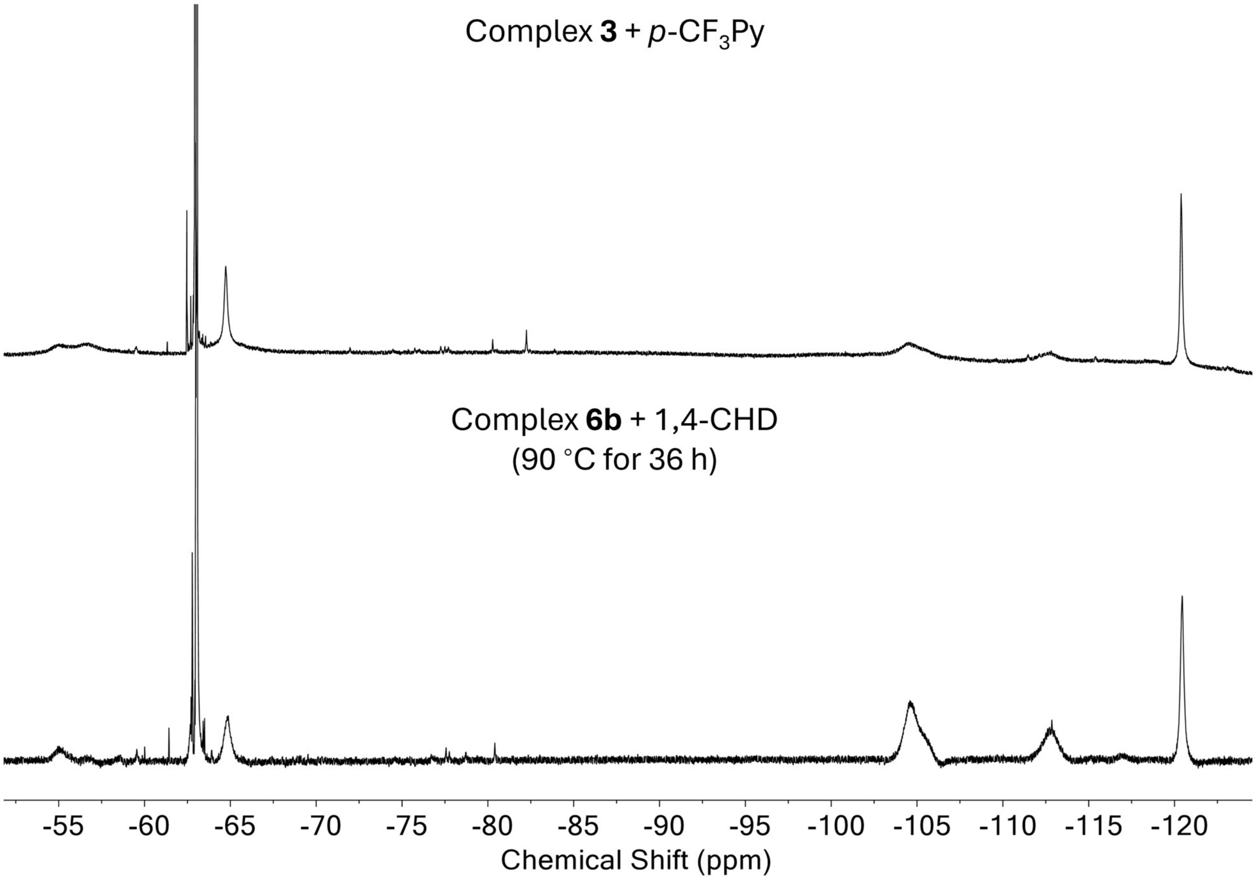

Figure S-70. Stacked ^{19}F NMR (564 MHz; C_6D_6) spectra of the reaction of [Fe_2(^{Ph}Dbf)$_2$(μ−$NC_8H_3F_6$)($NC_6H_4F_3$)] (**6b**) reaction with 1,4-cyclohexadiene at 90 ˚C after 36 hours (*bottom*), complex **3** with 0.9 equivalents of *p*-CF3Py (*middle*), and monomer **5b** with 1,4-cyclohexadiene at 90 ˚C after 24 hours (*top*). Notably, the same species observed after consumption of the imido **6b** in the reaction of **6b** with 1,4-cyclohexadiene at 90 ˚C (36 h) are observed upon addition of 0.9 equivalents of 4-trifluoromethylpyridine. Due to the broadness of these signals, there is some baseline noise that is unavoidable; however, species that are less than 2% of solution are not marked as they are likely baseline noise.

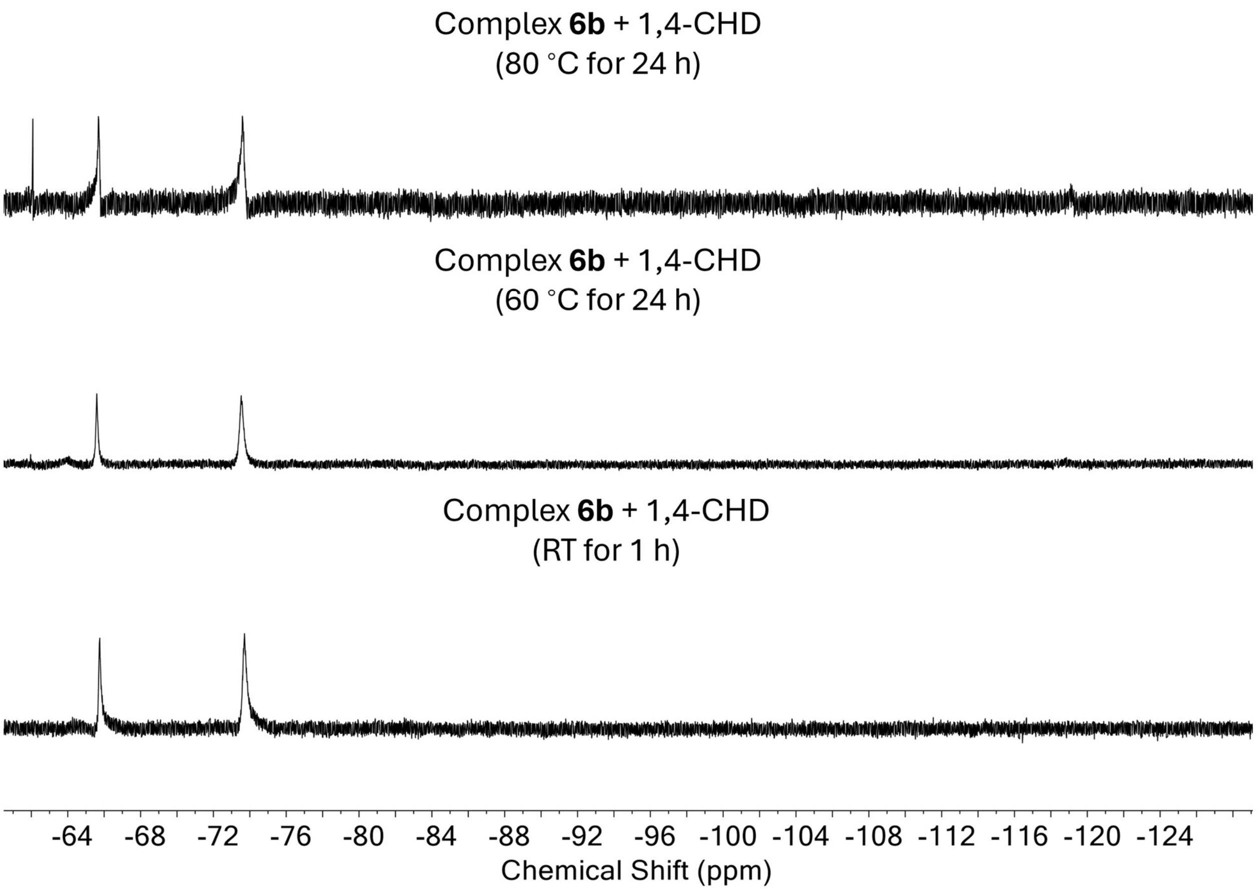

Figure S-71. ^{19}F NMR (376 MHz (*bottom*) and 564 MHz; C_6D_6) spectra of the reaction of [Fe_2(^{Ph}Dbf)$_2$(μ–$NC_8H_3F_6$)($NC_6H_4F_3$)] (**6b**) reaction with 1,4-cyclohexadiene at room temperature for 1h, 60 ˚C overnight, and 80 ˚C overnight. Complex **6b** remains stable at all temperatures under 90 ˚C.

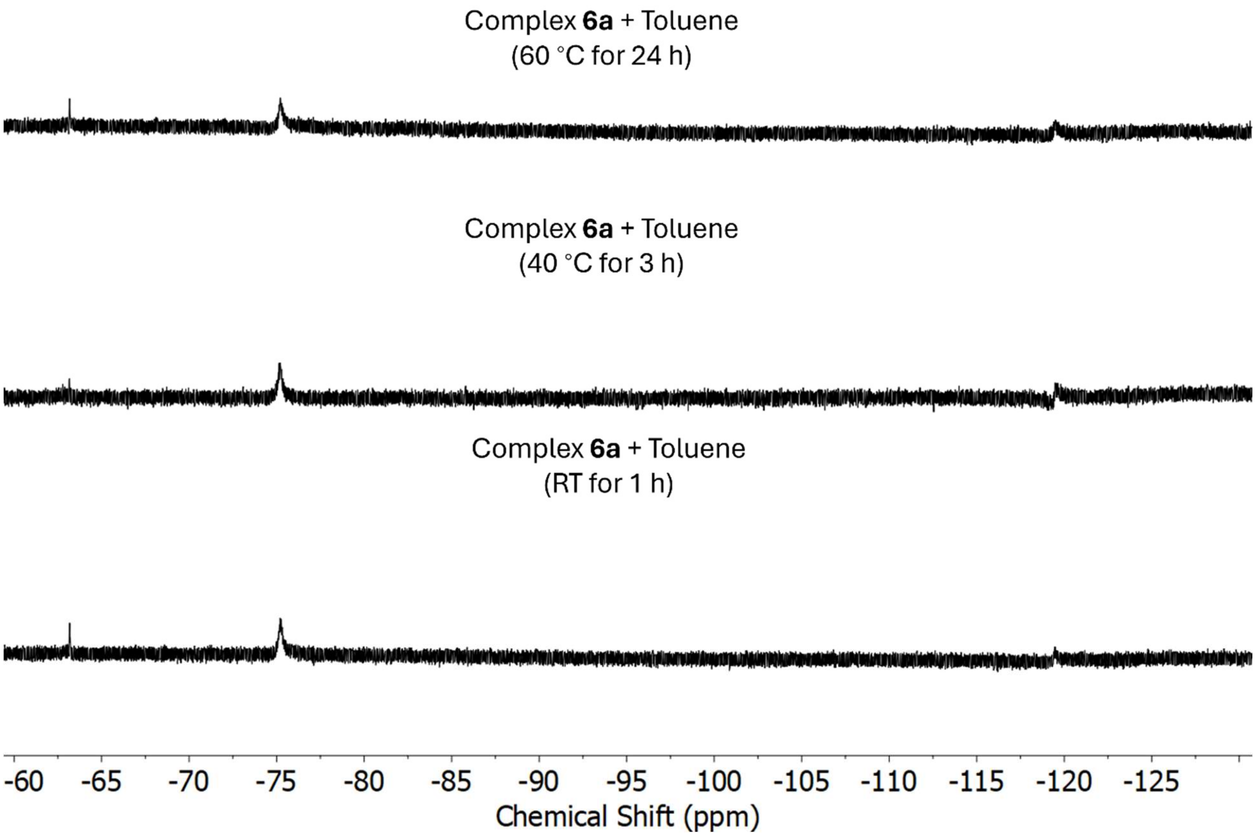

Figure S-72. Stacked ^{19}F NMR (376 MHz) spectra (*unlocked in toluene*) for paramagnetic $[Fe_2(^{Ph}Dbf)_2(\mu{-}NC_8H_3F_6)(NC_5H_5)]$ (**6a**) reaction with toluene Complex **6a** remains stable at temperatures under 80 ˚C.

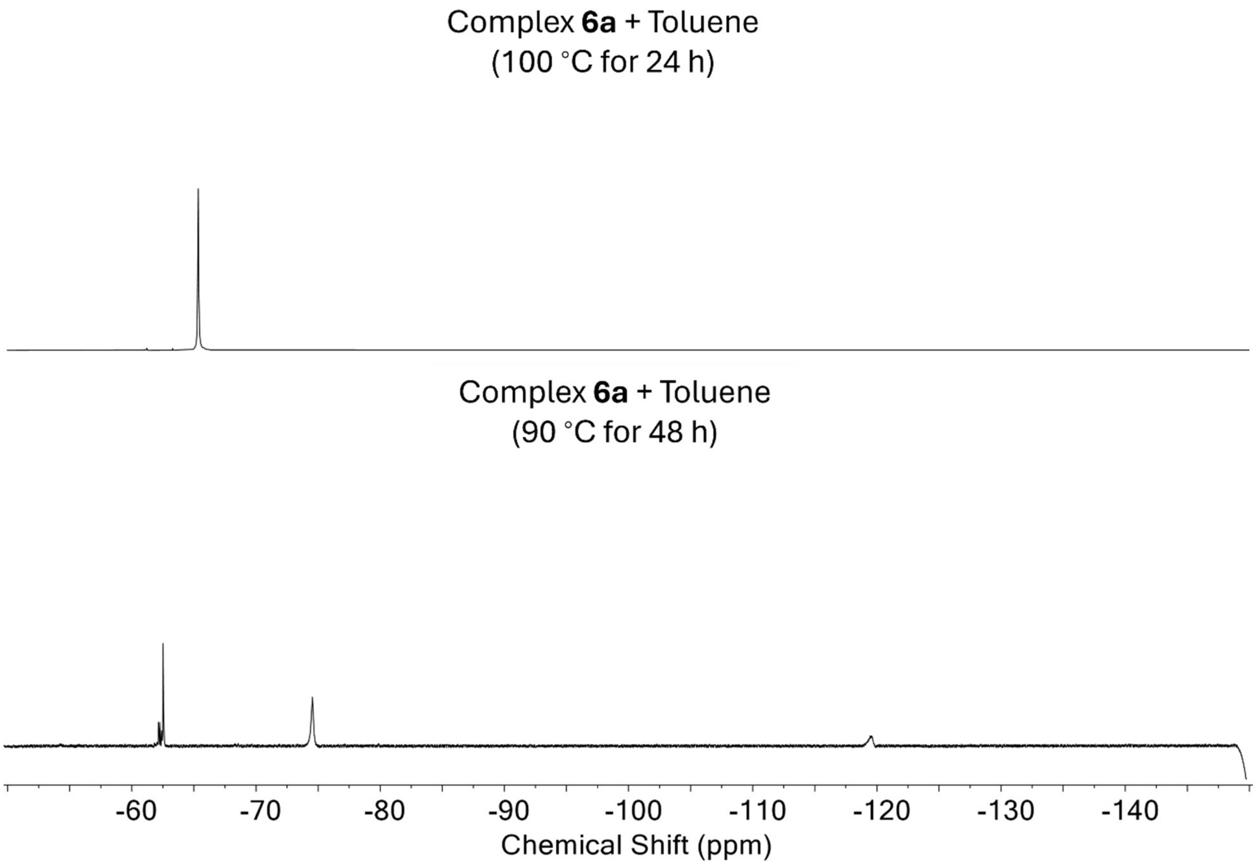

Figure S-73. Stacked ^{19}F NMR (376 MHz) spectra (*unlocked in toluene*) for paramagnetic $[Fe_2(^{Ph}Dbf)_2(\mu{-}NC_8H_3F_6)(NC_5H_5)]$ (**6a**) reaction with toluene Complex **6a** begins HAA at 90 ˚C but does not generate significant amounts of complex **3** after 48h (*bottom*). When heated at 100 ˚C complex **6a** goes to completion to generate the aminated product aminated product 3,5- *N*-benzyl-3-5-bis(trifluoromethyl)aniline.

Reactions with [Fe(^{Ph}Dbf) (μ-$NHC_8H_3F_6$)($NHC_8H_3F_6$)] (3) with Radical Substrates

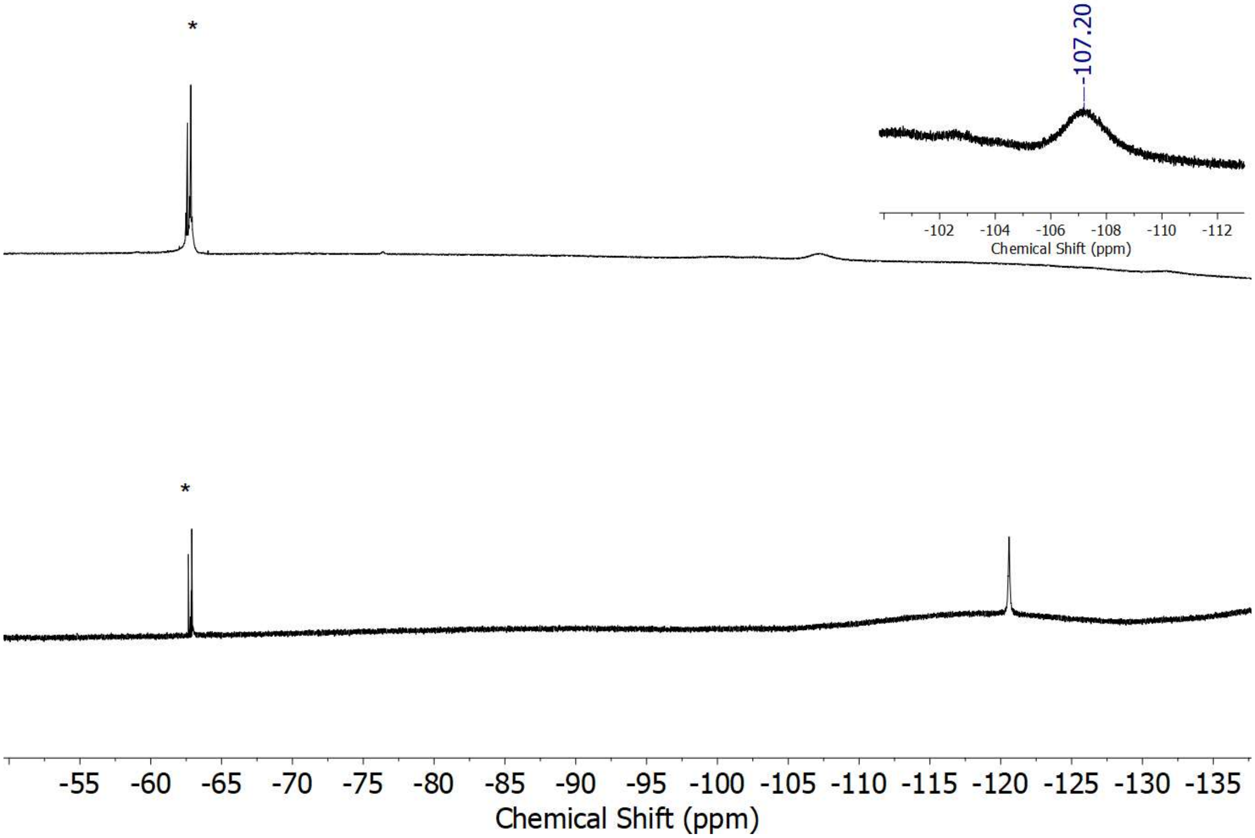

Figure S-74. Stacked ^{19}F NMR (564 MHz, C_6D_6) spectra of the reaction of paramagnetic [Fe(^{Ph}Dbf) (μ-$NHC_8H_3F_6$)($NHC_8H_3F_6$)] (**3**) reaction with 9-azabicyclo[3.3.1]nonane-N-oxyl (*top*). The reaction of [Fe(^{Ph}Dbf)(μ-$NHC_8H_3F_6$)($NHC_8H_3F_6$)] (**3**) (*bottom*) with 9-azabicyclo[3.3.1]nonane-N-oxyl (*top*) indicated the formation of a single new species with a chemical shift of -107.20 ppm (*inlay*), which is reminiscent of the bridging amide alkoxide (**4**) that forms upon the reaction of **2a** with triphenylmethanol. Therefore, we propose that this is the analogous bridging amide radical bound species. Likewise, this species is also stable at room temperature like complex **4**, suggesting that complex **3** cannot promote radical recombination in the absence of pyridine, as unwanted radical binding to the bridging amide species occurs. The ^{19}F NMR spectra of complex **3** used for this reaction (*bottom*) does indicate that some decomposition into the corresponding 3,5-bis(trifluoromethyl)aniline is observed (indicated by the *). Although the formation of this organic product is unavoidable, it does not interfere with the reaction, as indicated by the ^{19}F NMR.

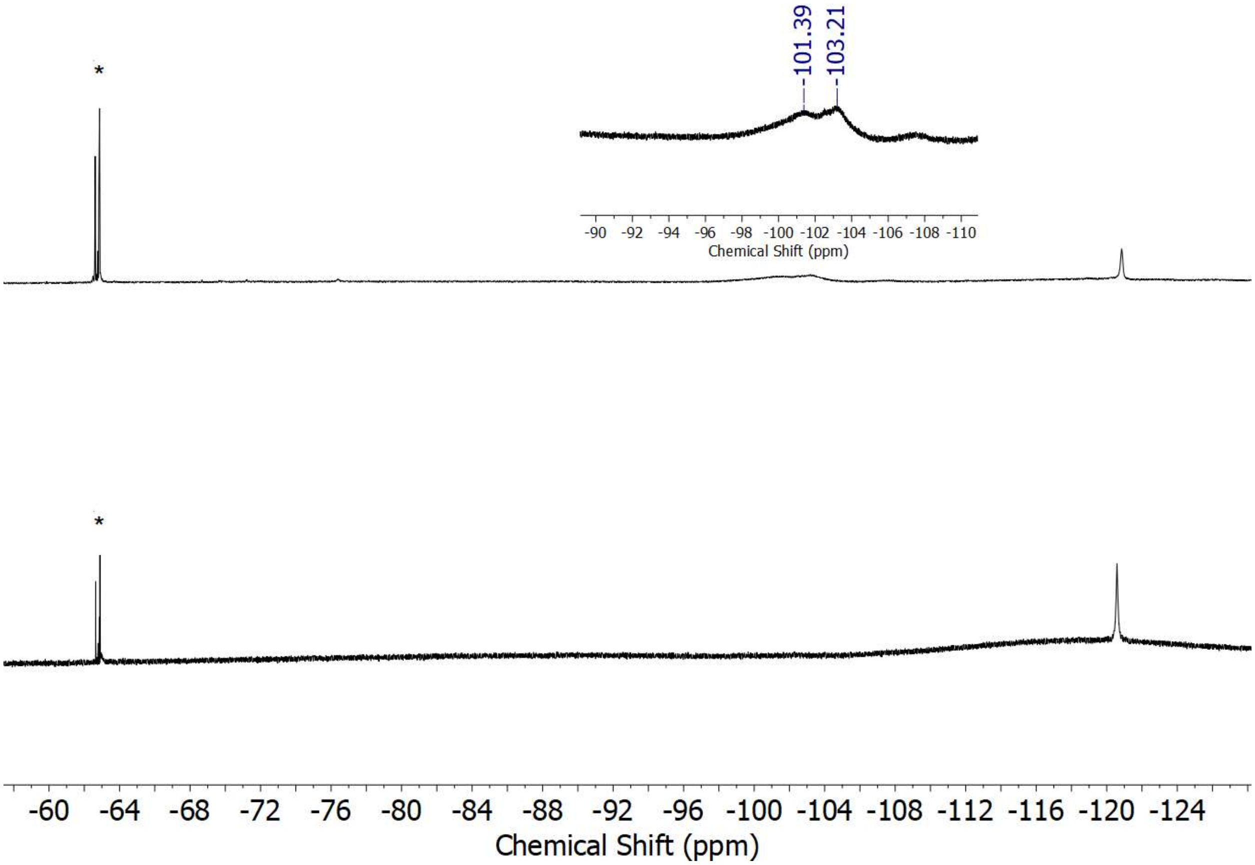

Figure S-75. Stacked ^{19}F NMR (564 MHz, C_6D_6) spectra of the reaction of paramagnetic [Fe(^{Ph}Dbf) (μ-$NHC_8H_3F_6$)($NHC_8H_3F_6$)] (**3**, *bottom*) reaction with Gombergs's dimer (*top*). The reaction of [Fe(^{Ph}Dbf) (μ-$NHC_8H_3F_6$)($NHC_8H_3F_6$)] (**3**) reaction with 9-azabicyclo[3.3.1]nonane-N-oxyl (*top*) indicated the formation of a single new species with a chemical shift of -107.20 ppm (*inlay*), which is reminiscent of the bridging amide alkoxide (**4**) that forms upon the reaction of **2a** with triphenylmethanol. Therefore, we propose that this is the analogous bridging amide radical bound species. Likewise, this species is also stable at room temperature like complex **4**, suggesting that complex **3** cannot promote radical recombination in the absence of pyridine, as unwanted radical binding to the bridging amide species occurs. Furthermore, this reaction does not consume the starting material, complex **3**, likely due to the steric bulk of this substrate. This agrees with studies that show that reactions of complex **2a** with bulky C–H bond substrates (2,4,6-trimethylphenol, *9H*–Fluorene, and triphenylmethane) do not go to completion. The ^{19}F NMR spectra of complex **3** used for this reaction (*bottom*) does indicate that some decomposition into the corresponding 3,5-bis(trifluoromethyl)aniline is observed (indicated by the *). Although the formation of this organic product is unavoidable, it does not interfere with the reaction, as indicated by the ^{19}F NMR.

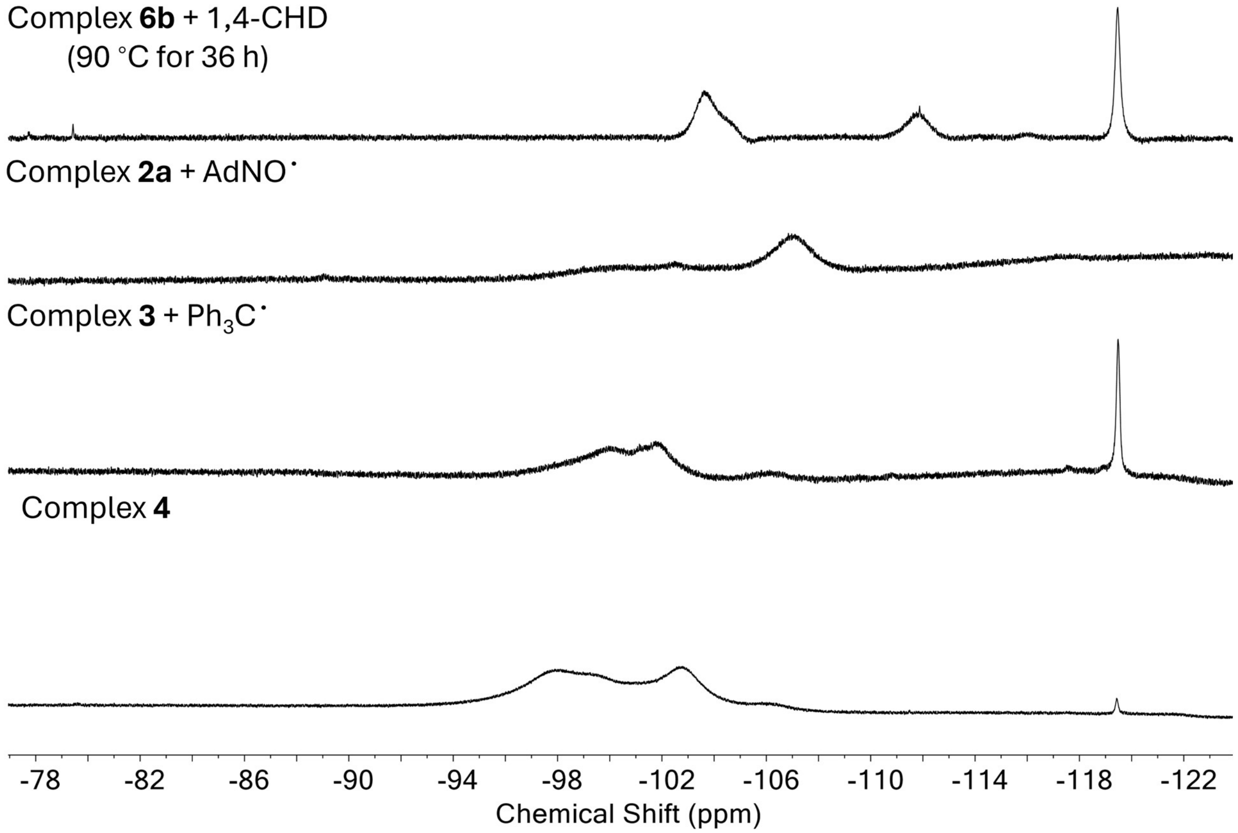

Figure S-76. Stacked ^{19}F NMR (564 MHz, C_6D_6) spectra of complex **4** (*bottom*) and all proposed asymmetric bridging amide complexes from reactions of complex **3** with organic (*middle)* and oxyl (*middle*) radicals, as well as species generated in the reaction of **6b** with 1,4-cyclohexadiene at 90 ˚C (*top*).

X-Ray Diffraction Techniques.

[Fe_2(^{Ph}Dbf)$_2$(μ−$NC_8H_3F_6$)] (2a): A blue irregular shaped crystal (0.35 x 0.24 x 0.10 mm^3) was centered on the goniometer of a Rigaku Oxford Diffraction Synergy-S diffractometer equipped with a HyPix6000HE detector and operating with MoKα radiation. The data collection routine, unit cell refinement, and data processing were carried out with the program CrysAlisPro.[4] The trigonal space group R-3 was assigned. The structure was solved using SHELXT[5] and refined using SHELXL[6] via Olex2[7]. The final refinement model involved anisotropic displacement parameters for non-hydrogen atoms and a riding model for all hydrogen atoms Disordered solvent could not be resolved; therefore, the solvent mask feature of Olex2 was used. A total of 1806 e$^-$ (2 hexanes/ASU; 1800 e$^-$ per unit cell) was subtracted from 10368 Å^3 total void space.

(Deposition #: 2364575)

[Fe_2(^{Ph}Dbf)$_2$(μ−$NC_{10}H_{13}$)] (2b): A blue irregular shaped crystal (0.22 × 0.010 × 0.05 mm^3) was centered on the goniometer of a Rigaku Oxford Diffraction Synergy-S diffractometer equipped with a HyPix6000HE detector and operating with MoKα radiation. The data collection routine, unit cell refinement, and data processing were carried out with the program CrysAlisPro.[4] The trigonal space group R-3 was assigned. The structure was solved using SHELXT[5] and refined using SHELXL[6] via Olex2.[7] The final refinement model involved anisotropic displacement parameters for non-hydrogen atoms and a riding model for all hydrogen atoms. Due to poor data quality (Rint = 0.16, <I>/<sigI> = 6.2 to max resolution = 0.84) this structure was used primarily for identification. Disordered solvent could not be resolved; therefore, the solvent mask feature of Olex2 was used. A total of 1800 e$^-$ (2 hexanes/ASU; 1800 e$^-$ per unit cell) was subtracted from 11559 Å^3 total void space.

(Deposition #: 2364577)

[Fe_2(^{Ph}Dbf)$_2$(μ-$NHC_8H_3F_6$)($NHC_8H_3F_6$)] (3): A prism shaped blue crystal (0.07 x 0.12 x 0.22) was centered on the goniometer of a Rigaku Oxford Diffraction Synergy-S diffractometer equipped with a HyPix6000HE detector and operating with CuKα radiation. The data collection routine, unit cell refinement, and data processing were carried out with the program CrysAlisPro.[4] The monoclinic space group $P2_1/n$ was assigned. The structure was solved using SHELXT[5] and refined using SHELXL[6] via Olex2[7]. The final refinement model involved anisotropic displacement parameters for non-hydrogen atoms and a riding model for all hydrogen atoms. This structure was used primarily for identification. The assignment of the N-H hydrogens was confirmed via experimental data and thorough comparison of bond metrics (Fig. SI-XX) with reported bond metrics for bridging and terminal imido, amide, and aniline diiron complexes using the reported structures in the CSD.

(Deposition #: 2364574)

$[Fe_2(^{Ph}Dbf)_2(\mu\text{-}NHC_8H_3F_6)(OC_{19}H_{15})]$ (4): An irregular shaped blue crystal (0.51 × 0.3 × 0.16 mm^3) was centered on the goniometer of a Rigaku Oxford Diffraction Synergy-S diffractometer equipped with a HyPix6000HE detector and operating with MoKα radiation. The data collection routine, unit cell refinement, and data processing were carried out with the program CrysAlisPro.[4] The orthorhombic space group *Fdd*2 was assigned. The structure was solved using SHELXT[5] and refined using SHELXL[6] via Olex2[7]. The final refinement model involved anisotropic displacement parameters for non-hydrogen atoms and a riding model for all hydrogen atoms. Due to poor data quality (Rint = 0.1986, <I>/<sigI> = 6.4 to max resolution = 0.84) this structure was used primarily for identification. Disordered solvent could not be resolved; therefore, the solvent mask feature of Olex2 was used. A total of 596 e^- was subtracted from 10248 Å^3 total void space. This was assigned as one trifluorotoluene/ASU corresponding to 592 e^- per unit cell.

(Deposition #: 2364573)

$[Fe_2(^{Ph}Dbf)_2(\mu\text{-}NHC_8H_3F_6)(NC_5H_5)]$ (6a): A prism shaped blue crystal (0.07 x 0.12 x 0.22 mm^3) was centered on the goniometer of a Rigaku Oxford Diffraction Synergy-S diffractometer equipped with a HyPix6000HE detector and operating with CuKα radiation. The data collection routine, unit cell refinement, and data processing were carried out with the program CrysAlisPro.[4] The triclinic space group *P-1* was assigned. The structure was solved using SHELXT[5] and refined using SHELXL[6] via Olex2[7]. The final refinement model involved anisotropic displacement parameters for non-hydrogen atoms and a riding model for all hydrogen atoms. Disordered solvent could not be resolved; therefore, the solvent mask feature of Olex2 was used. A total of 275 e^- was subtracted from 1009 Å^3 total void space. This was assigned as two trifluorotoluene/ASU corresponding to 296 e^- per unit cell.

(Deposition #: 2364576)

Table S-4. X-ray diffraction experimental details

	$[Fe_2(^{Ph}Dbf)_2(\mu{-}NC_8H_3F_6)]$ (**2a**)	$[Fe_2(^{Ph}Dbf)_2(\mu{-}NC_{10}H_{13})]$ (**2b**)
**Empirical Formula**	$C_{84}H_{55}F_6Fe_2NO_6$ [2 Hexanes]	$C_{86}H_{65}Fe_2NO_6$ [2 Hexanes]
**Formula Weight**	1572.33	1492.43
**Temp. (K)**	100.15	100.15
**Radiation**	Mo	Mo
**Crystal System**	Trigonal	Trigonal
**Space Group**	R-3	R-3
**a (Å)**	31.7788(5)	38.171(2)
**b (Å)**	31.7788(5)	38.171(2)
**c (Å)**	42.3830(8)	29.7234(10)
**α (°)**	90	90
**β (°)**	90	90
**γ (°)**	120	120
**Volume (Å^3)**	37067.8(14)	37468(4)
**Z**	18	18
**ρ_{calc} (g/cm^3)**	1.268	1.191
**μ (mm^{-1})**	0.420	0.403
**F(000)**	14760.0	14184.0
**Crystal size (mm^3)**	0.35 x 0.24 x 0.1	0.22 × 0.10 × 0.05
**2Θ range for data collection (°)**	5.294 – 61.014	5.118 – 50.054
**Index Ranges**	$-43 \leq h \leq 45$	$-44 \leq h \leq 42$
	$-45 \leq k \leq 45$	$-43 \leq k \leq 45$
	$-54 \leq l \leq 60$	$-35 \leq l \leq 35$

**Reflections Collected**	104954	56342
**Independent Reflections**	25153	14684
**Data/Restraints/Parameters**	25153/0/892	14684/0/859
**GOF**	1.044	0.944
**Final R indexes [I>=2σ (I)]**	R_1 = 0.0622, wR2 = 0.1727	R_1 = 0.0714, wR2 = 0.1539
**Final R indexes [all data]**	R_1 = 0.0976, wR2 = 0.1891	R_1 = 0.1551, wR2 = 0.1845
**Largest diff. peak/hole/e (Å^{-3})**	1.57/-0.71	0.33/-0.30

Table S-4 continued. X-ray diffraction experimental details

	$[Fe_2(^{Ph}Dbf)_2(\mu\text{-}NHC_8H_3F_6)(NHC_8H_3F_6)]$ (**3**)	$[Fe_2(^{Ph}Dbf)_2(\mu\text{-}NHC_8H_3F_6)(OC_{19}H_{15})]$ (**4**)
**Empirical Formula**	$C_{76}H_{52}Fe_2O_6$	$C_{106.5}H_{73.5}F_{7.5}Fe_2NO_7$
	[2 Benzene]	[0.5 $C_7H_5F_3$]
**Formula Weight**	1785.33	1733.36
**Temp. (K)**	100	100.15
**Radiation**	Cu	Mo
**Crystal System**	Monoclinic	Orthorhombic
**Space Group**	$P2_1/n$	Pbca
**a (Å)**	13.2783(3)	27.6157(8)
**b (Å)**	39.4242(9)	24.3842(6)
**c (Å)**	16.7936(4)	55.758(2)
**α (°)**	90	90
**β (°)**	110.726(3)	90
**γ (°)**	90	90

**Volume (Å^3)**	8222.3(4)	37547(2)
**Z**	4	16
**ρ_{calc} (g/cm^3)**	1.442	1.227
**μ (mm^{-1})**	3.578	0.379
**F(000)**	3672.0	14320.0
**Crystal size (mm^3)**	0.02 x 0.04 x 0.26	0.51 × 0.3 × 0.16
**2Θ range for data collection (°)**	6.058 – 133.202	4.918 – 50.054
**Index Ranges**	$-15 \leq h \leq 15$	$-31 \leq h \leq 32$
	$-46 \leq k \leq 43$	$-22 \leq k \leq 29$
	$-19 \leq l \leq 19$	$-66 \leq l \leq 62$
**Reflections Collected**	53520	155052
**Independent Reflections**	14471	33085
**Data/Restraints/Parameters**	14909/0/1143	33085/0/2143
**GOF**	1.022	0.959
**Final R indexes [I>=2σ (I)]**	R_1 = 0.0538, wR2 = 0.1296	R_1 = 0.0871, wR2 = 0.2184
**Final R indexes [all data]**	R_1 = 0.0800 wR2 = 0.1409	R_1 = 0.1607, wR2 = 0.2573
**Largest diff. peak/hole/e (Å^{-3})**	0.79/-0.83	0.70/-0.48

Table S-4 continued. X-ray diffraction experimental details

	$[Fe_2(^{Ph}Dbf)_2(\mu\text{-}NHC_8H_3F_6)(NHC_5H_5)]$ (**6a**)
**Empirical Formula**	$C_{89}H_{49}Fe_2O_6N_2F_6$ [2 Trifluorotoluene]
**Formula Weight**	1770.30
**Temp. (K)**	100
**Radiation**	Cu
**Crystal System**	Triclinic
**Space Group**	P-1
**a (Å)**	13.25640(10)
**b (Å)**	13.90670(10)
**c (Å)**	23.4096(2)
**α (°)**	94.0170(10)
**β (°)**	96.6780(10)
**γ (°)**	105.4990(10)
**Volume (Å^3)**	4107.35(6)
**Z**	2
**ρ_{calc} (g/cm^3)**	1.431
**μ (mm^{-1})**	3.577
**F(000)**	1818.0
**Crystal size (mm^3)**	0.07 x 0.12 x 0.22
**2Θ range for data collection (°)**	6.632 – 155.08
**Index Ranges**	$-15 \leq h \leq 16$<br>$-17 \leq k \leq 17$

	$-29 \leq l \leq 29$
**Reflections Collected**	98938
**Independent Reflections**	17190
**Data/Restraints/Parameters**	17190/0/946
**GOF**	1.098
**Final R indexes [I>=2σ (I)]**	R_1 = 0.0512, wR2 = 0.1485
**Final R indexes [all data]**	R_1 = 0.0544 wR2 = 0.1513
**Largest diff. peak/hole/e ($Å^{-3}$)**	0.72/-0.70

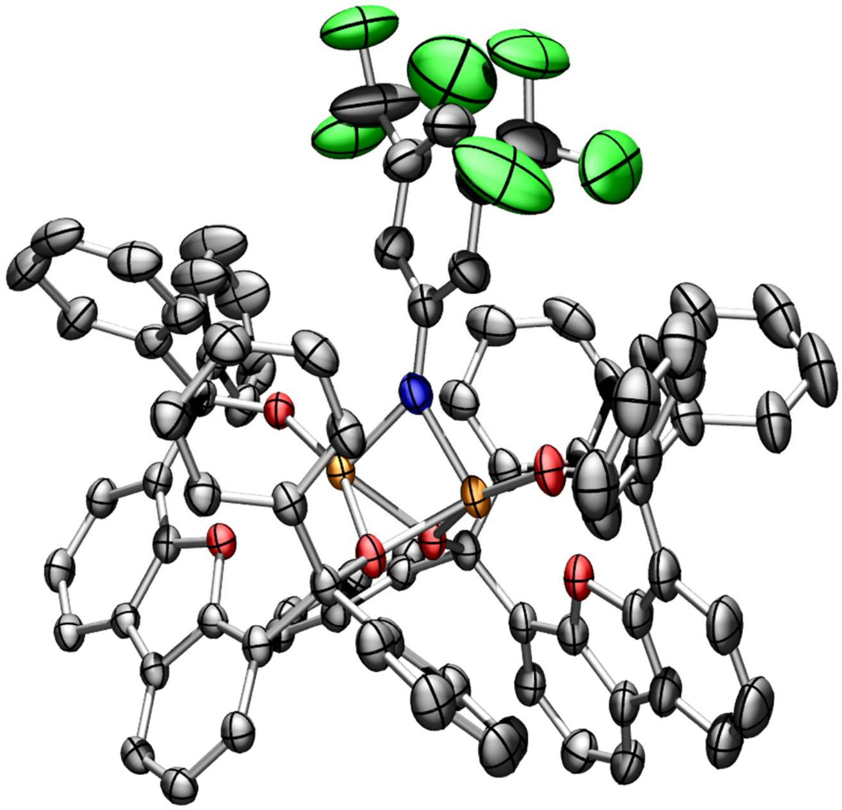

Figure S-77. Solid-state molecular structure for [Fe_2(^{Ph}Dbf)$_2$(μ–$NC_8H_3F_6$)] (**2a**) with anisotropic displacement ellipsoids at 50% probability level. Hydrogen atoms and benzene solvent in the unit cell are omitted for clarity. Color scheme: Fe – orange, O – red, N – blue, C – gray, F – green.

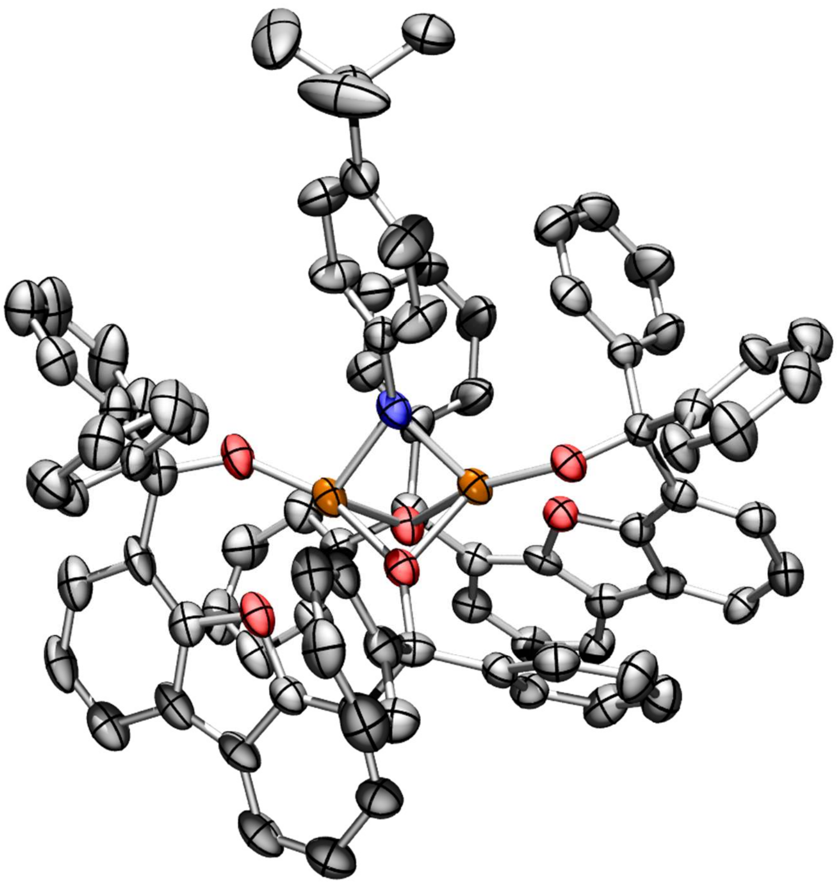

Figure S-78. Solid-state molecular structure for $[Fe_2(^{Ph}Dbf)_2(\mu-NC_{10}H_{13})]$ (**2b**) with anisotropic ellipsoids at 50% probability level. Hydrogen atoms and benzene solvent in the unit cell are omitted for clarity. Color scheme: Fe – orange, O – red, N – blue, C – gray.

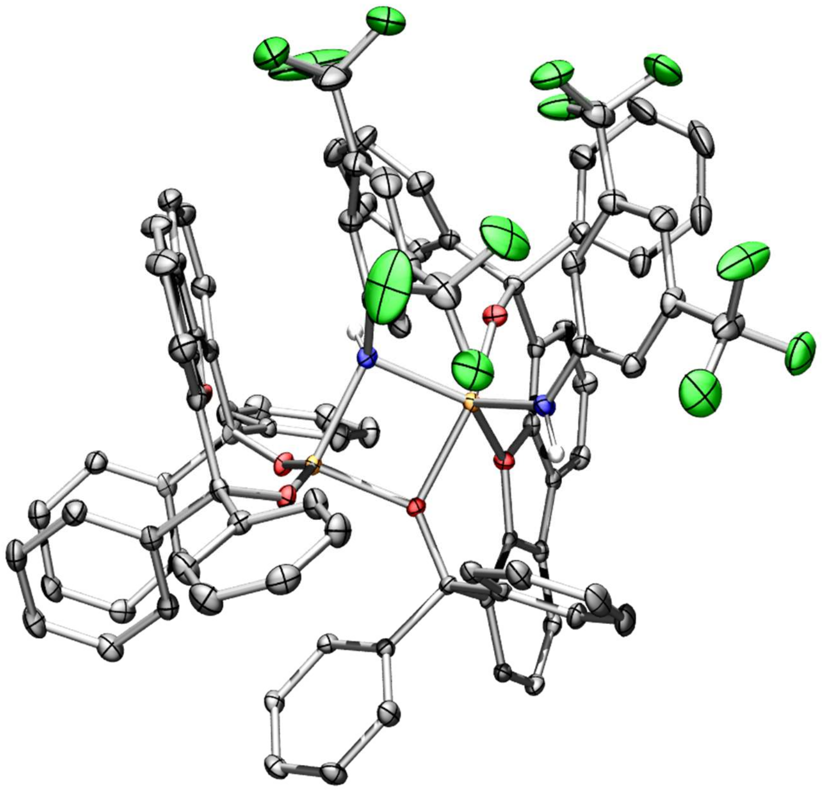

Figure S-79. Truncated solid-state molecular structure for $[Fe_2(^{Ph}Dbf)_2(\mu\text{-}NHC_8H_3F_6)(NHC_8H_3F_6)]$ (**3**) with anisotropic ellipsoids at 50% probability level. Hydrogen atoms (except NH) and benzene solvent in the unit cell are omitted for clarity. Color scheme: Fe – orange, O – red, N – blue, C – gray, F – green.

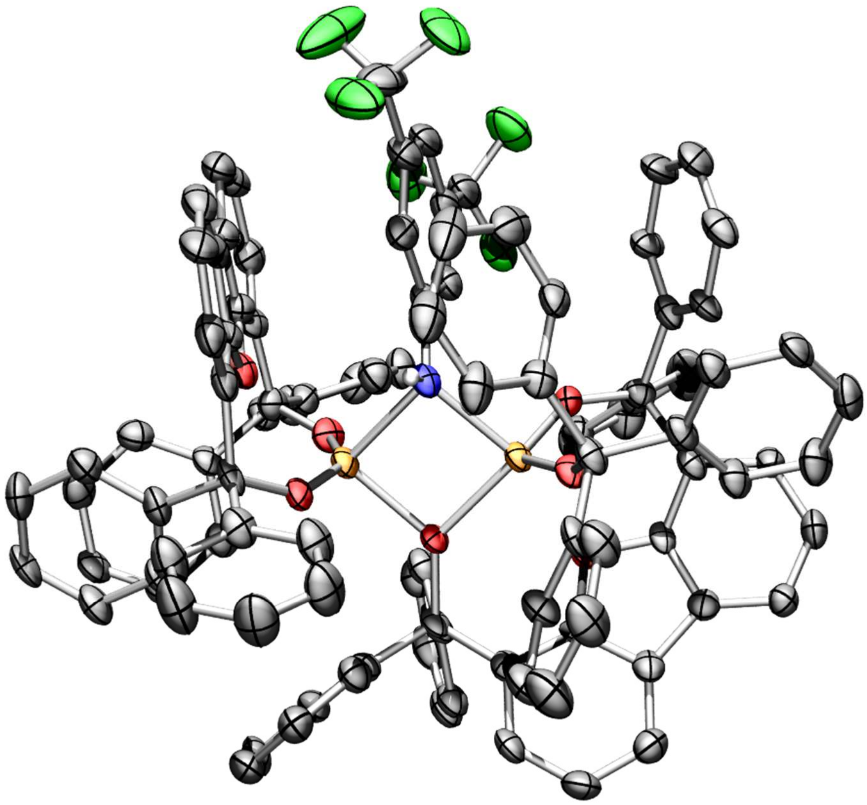

Figure S-80. Solid-state molecular structure for $[Fe_2(^{Ph}Dbf)_2(\mu\text{-}NHC_8H_3F_6)(OC_{19}H_{15})]$ (**4**) with anisotropic ellipsoids at 50% probability level. Hydrogen atoms (except NH) and benzene solvent in the unit cell are omitted for clarity. Color scheme: Fe – orange, O – red, N – blue, C – gray, F – green.

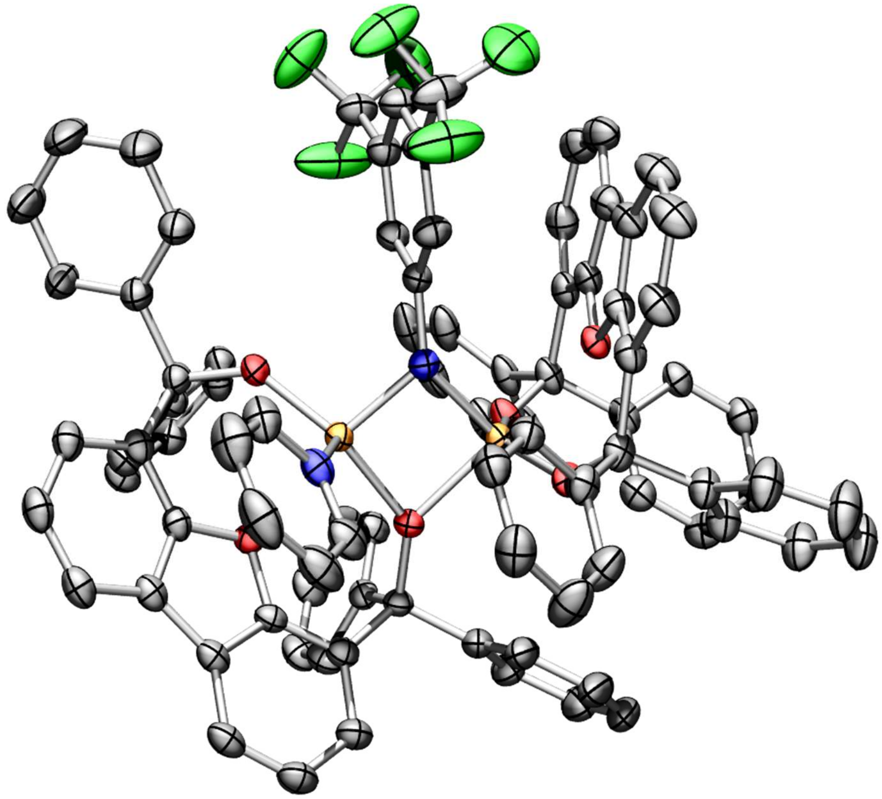

Figure S-81. Solid-state molecular structure for $[Fe_2(^{Ph}Dbf)_2(\mu\text{-}NHC_8H_3F_6)(NC_5H_5)]$ (**6a**) with anisotropic ellipsoids at 50% probability level. Hydrogen atoms and benzene solvent in the unit cell are omitted for clarity. Color scheme: Fe – orange, O – red, N – blue, C – gray, F – green.

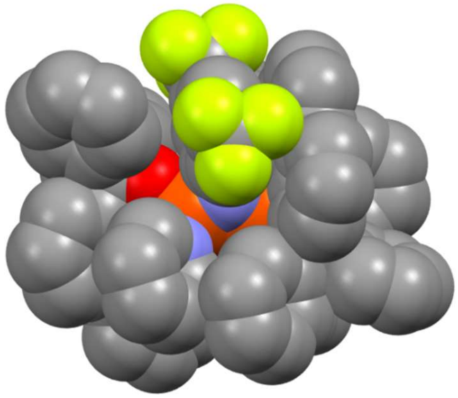

Figure S-82. Space fill model for [Fe_2(^{Ph}Dbf)$_2$(μ−$NC_8H_3F_6$)] (**2a**). Hydrogen atoms were omitted for clarity. Color scheme: Fe – orange, O – red, N – blue, C – gray, F – green.

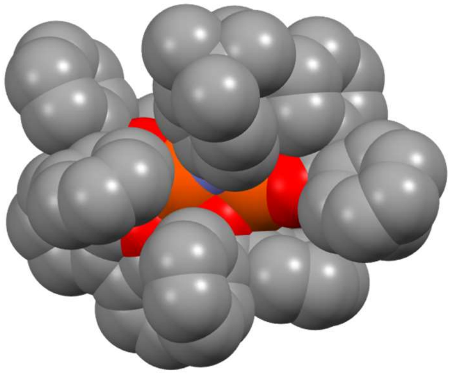

Figure S-83. Space fill model for $[Fe_2(^{Ph}Dbf)_2(\mu{-}NC_{10}H_{13})]$ (**2b**). Hydrogen atoms were omitted for clarity. Color scheme: Fe – orange, O – red, N – blue, C – gray, F – green.

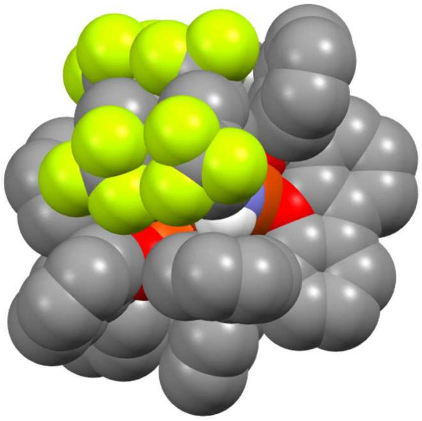

Figure S-84. Space fill model for [Fe_2(^{Ph}Dbf)$_2$(μ−$NHC_8H_3F_6$)((μ−$NHC_8H_3F_6$)] (**3**). Hydrogen atoms were omitted for clarity apart from the amide N–H bonds. The right most NHCH ligand is the terminally bound ligand. Color scheme: Fe – orange, O – red, N – blue, C – gray, F – green.

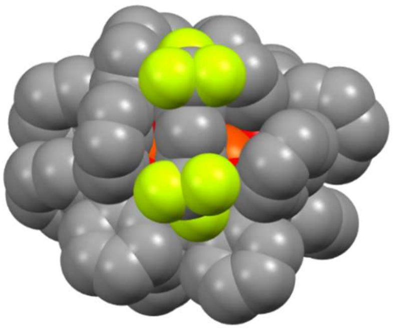

Figure S-85. Space fill model for $[Fe_2(^{Ph}Dbf)_2(\mu-NHC_8H_3F_6)(OCPh_3)]$ (**4**). Hydrogen atoms were omitted for clarity. Color scheme: Fe – orange, O – red, N – blue, C – gray, F – green.

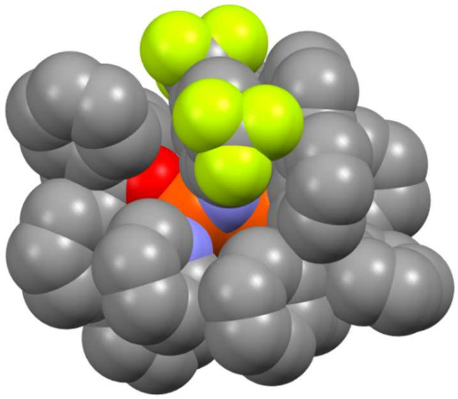

Figure S-86. Space fill model for $[Fe_2(^{Ph}Dbf)_2(\mu{-}NHC_8H_3F_6)(NC_5H_5)]$ (**6a**). Hydrogen atoms were omitted for clarity. Color scheme: Fe – orange, O – red, N – blue, C – gray, F – green.

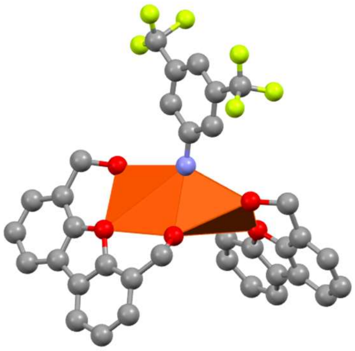

Figure S-87. Polyhedral model for $[Fe_2(^{Ph}Dbf)_2(\mu{-}NC_8H_3F_6)]$ (**2a**) Hydrogen atoms and phenyl substituents on the ^{Ph}Dbf ligand are excluded for clarity. Color scheme: Fe – orange, O – red, N – blue, C – gray, F – green.

Table S-5. Selected bond distances and angles for complexes **2a**, **2b**, and **6a**.

Metrics	**2a**	**2b**	**6a**
Fe_1–Fe_2	2.5180(4)	2.5269(9)	2.8124(4)
Fe_1–N_1	1.887(2)	1.898(4)	1.8618(17)
Fe_2–N_1	1.905(2)	1.884(4)	1.8927(17)
N_1–C_1	1.379(3)	1.398(6)	1.385(3)
Fe_1–O_1	2.0856 (13)	2.047(3)	1.9674(13)
Fe_1–O_3	2.0002(15)	1.979(3)	-
Fe_1–N_{Py}	-	-	2.1064(17)
N_{Py}–C_{13}	-	-	1.349(3)
Fe_1–N_1–Fe_2	83.20(9)	83.85(16)	97.01(8)
Fe_1–N_1–C_1	139.87(17)	134.0(4)	124.08(13)
Fe_2–N_1–C_1	136.90(16)	138.9(3)	134.31(14)
Fe_1–O_3–Fe_2	76.79(6)	76.34(10)	88.13(5)
Fe_1–O_1–Fe_2	-	-	88.13(5)

Table S-6. Selected bond distances and angles for complexes **3** and **4**.

Metrics	**3**	**4**
Fe_1–Fe_2	3.0690(7)	3.0484(11)
Fe_1–N_1	2.096(3)	2.064(4)
Fe_2–N_1	2.048(3)	2.053(4)
Fe_1–N_2	1.914(3)	-
Fe_1–N_1–Fe_2	95.53(11)	95.53(18)
Fe_1–N_1–C_1	112.8(2)	122.2(3)
Fe_2–N_1–C_1	127.8(2)	120.8(4)
Fe_1–N_2–C_9	133.0(2)	-

Table S-7. Bond metrics for **3** and **4** and average bond lengths and angles calculated from reported structures in the CSD for related bridging aryl imido[8-10], bridging aryl bis(imido)[11-15], terminal aniline[8, 16-23] and bridging[14, 24, 25] and terminal[26-31] aryl amide complexes. All values were calculated in Excel from data obtained from the .cif files for each complex. Average bond lengths were calculated using the average function in excel and rounded to the least number of significant figures for each sample pool. In Excel, error for each measurement was recorded and used to calculate using the propagation of error formula: $\frac{\sigma_x}{x} = \frac{\sqrt{\sum_{i=\sigma_n}^{n} i^2}}{N}$.

Bond Metrics	**3**	**4**	**Diiron Imido**	**Diiron Bis(Imido)**	**Terminal Amides**	**Diiron Amides**	**Terminal Anilines**
Fe_n– $N_{(Bridging)}$	2.096(3)<br>2.048(3)	2.064(4)<br>2.053(4)	1.879(4)	1.876(1)	–	2.017(1)	–
Fe_1–$N_{(Terminal)}$	1.914(3)	–	–	–	1.927(2)	–	2.14(1)
Fe_1–N_1–Fe_2	95.53(11)	95.53(18)	122.4(3)	83.58(3)	–	87.1(1)	–
Fe_n–$N_{(bridging)}$–C	112.8(2)<br>127.8(2)	122.2(3)<br>120.8(4)	118.7(1)	137.72(5)	–	120.8(1)	–
Fe_n–$N_{(terminal)}$–C	133.0(2)	–	–	–	134.1(7)	–	118.3(5)

Table S-8. Calculated Spin Densities for Complexes **2a**, **6a**, **3** and **4**.

Complex	Theory Level	Basis Set	Spin Density $N_{imido/amide}$
**2a**	B3LYP	LANL2DZ	0.794
	B3LYP	def2-SV(P)	−
	B3LYP	def2-SV(P)/TZVP	−
**6a**	B3LYP	LANL2DZ	0.789
	B3LYP	def2-SV(P)	−
	B3LYP	def2-SV(P)/TZVP	−
**3**	B3LYP	LANL2DZ	0.281, 0.361
	wB97X-D	LANL2DZ	0.278, 0.351
**4**	B3LYP	LANL2DZ	0.284
	wB97X-D	LANL2DZ	0.278

Gas Chromatography Mass Spectroscopy Traces

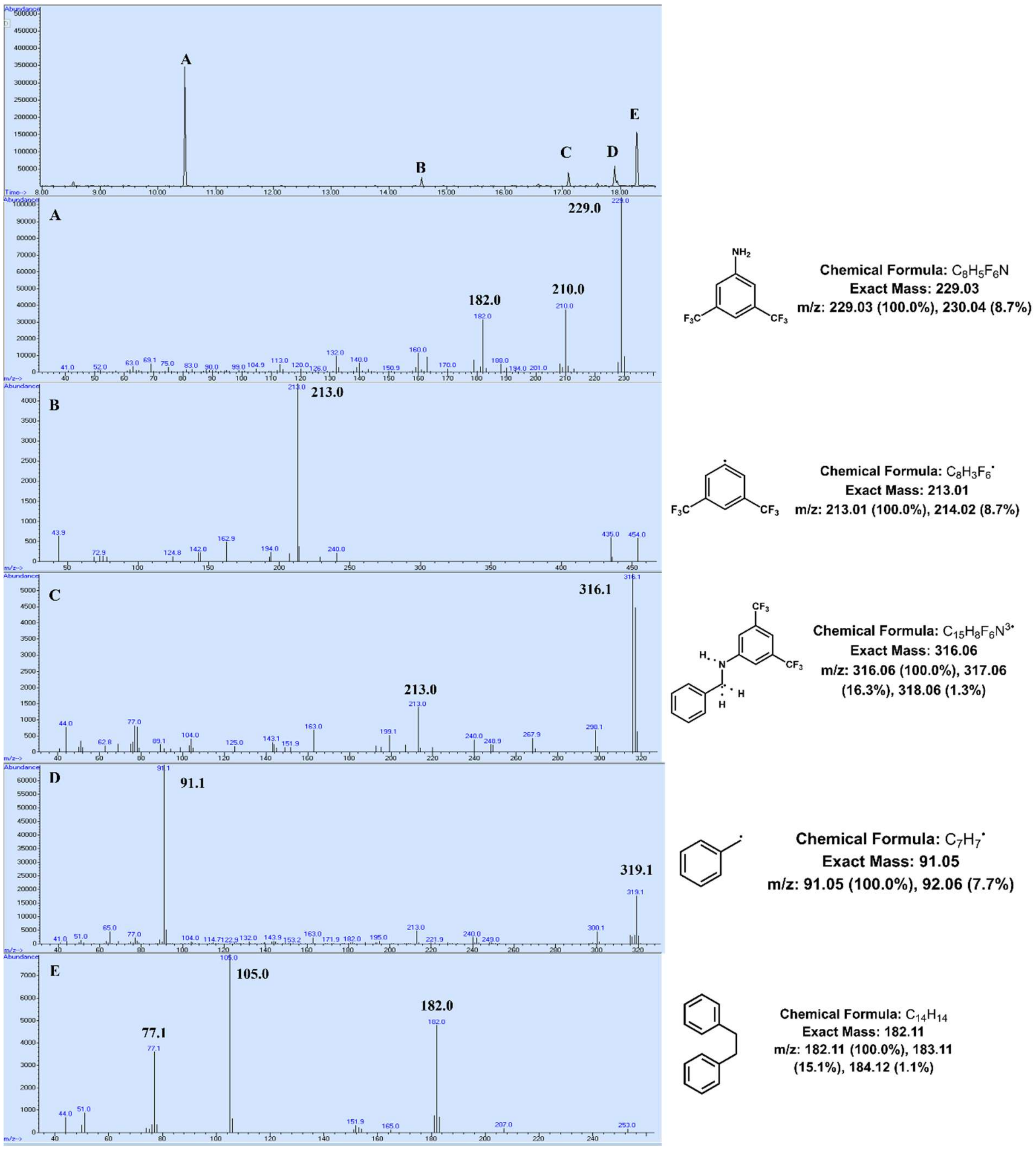

Figure S-88. Gas Chromatography Mass Spectroscopy Trace and spectra for the reaction of **2a** in Toluene heated at 80 ˚C for several days. Reaction was quenched with methanol and solvent was removed in vacuo. Paramagnetic materials were removed via a celite plug and organic materials eluted in 10:1 DCM:Methanol. The major product can be identified as 3,5-bis(trifluoromethyl)aniline (***A and B***), accompanied by bibenzyl (**E**) as expected if HAA from toluene occurred. Interestingly, a small quantity of the aminated product (**C**) was detected, as well as the benzylic radical fragment (**D**) resulting from the fragmentation of the aminated product.

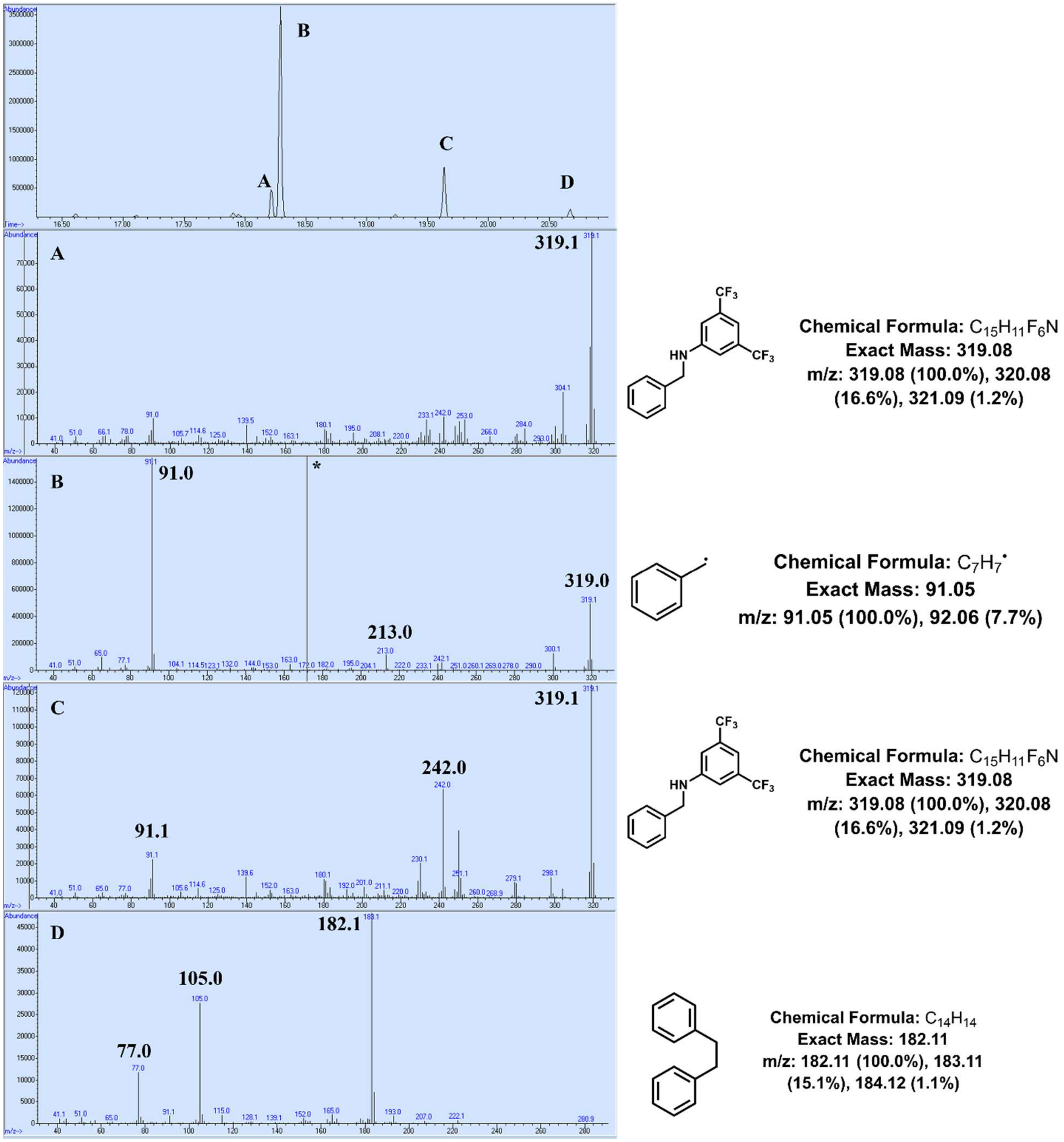

Figure S-89. Gas Chromatography Mass Spectroscopy Trace and spectra for the reaction of **6a** in Toluene heated at 100 ˚C . Reaction was quenched with methanol and solvent was removed in vacuo. Paramagnetic materials were removed via a celite plug and organic materials eluted in 10:1 DCM:Methanol. The major product can be identified as the aminated product (***A** and **C***), and a small amount of bibenzyl (**D**). * indicates the cursor line accidentally captured in the screen clipping.

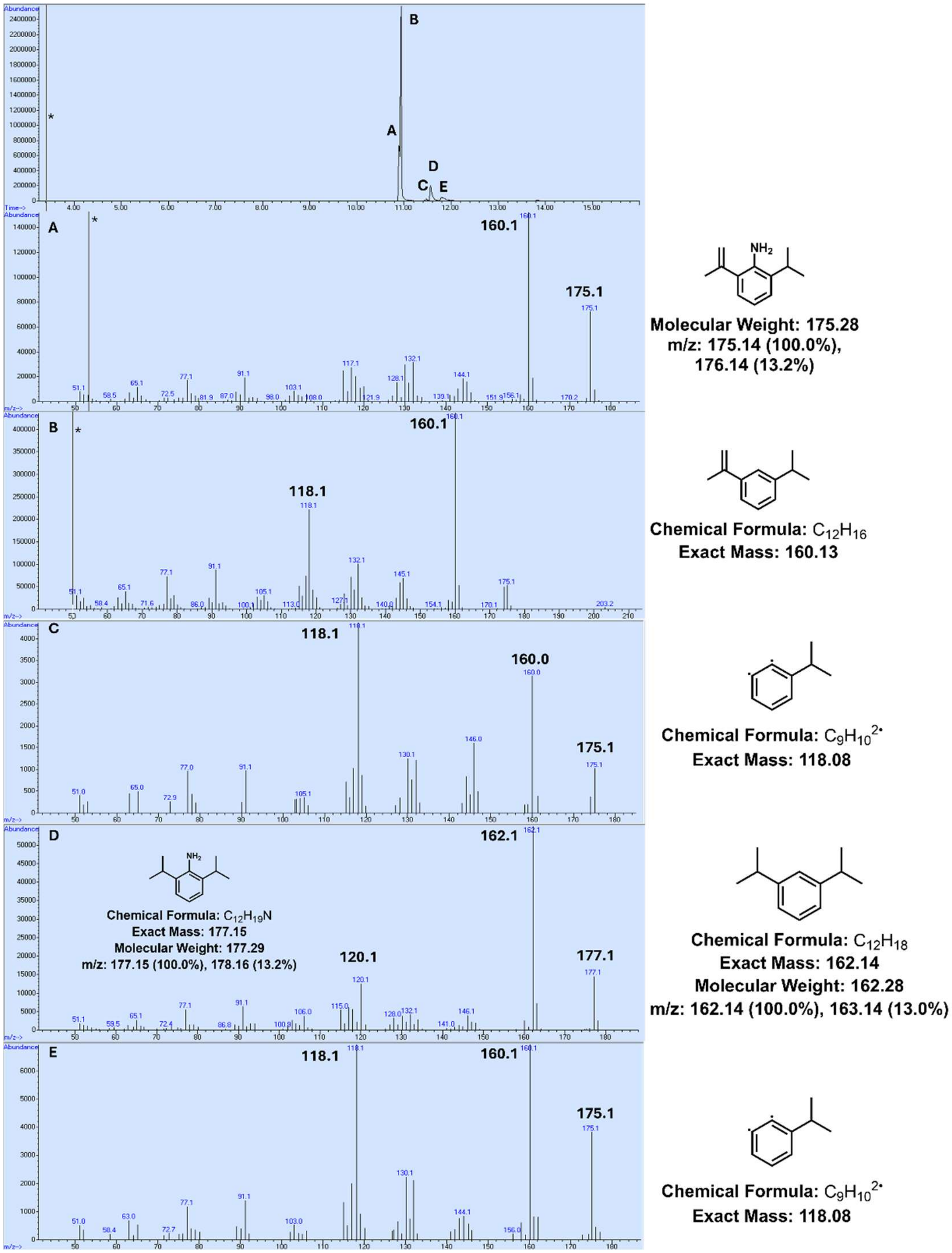

Figure S-90. Gas Chromatography Mass Spectroscopy Trace and spectra for the reaction of **2a** in 2,6-diisopropylphenyl azide heated at 80 ˚C . Reaction was quenched with methanol and solvent was removed in vacuo. Paramagnetic materials were removed via a celite plug and organic materials eluted in 10:1 DCM:Methanol. The major product can be identified as the dehydrogenated product or fragments resulting from the ionization of the product (***A***, ***B***, ***C**, and* ***E***), and some 2,6-diisopropylaniline (**D**). * indicates the cursor line accidentally captured in the screen clipping.

Table S-9. Coupling constants for **2a**, **6a**, **3**, and **4**.

2a

Functional	Basis Set	E(Ms=0, BS), hartrees	E(Ms=11), hartrees	<S^2> (Ms=0, BS)	<S^2> (Ms=11)	J (cm^{-1})
B3LYP	def2-SV(P)	-6862.207477	-6862.20058	4.7885	30.0304	**-60.0**
B3LYP	def2-SV(P)/TZVP	-6862.557208	-6862.55001	4.7995	30.0332	**-62.6**
B3LYP	LANL2DZ	-4584.228142	-4584.221904	4.789469301	30.03585451	**-54.2**
wB97X-D	def2-SV(P)	-6860.87088	-6860.866578	4.848	30.0362	**-37.5**
wB97X-D	def2-SV(P)/TZVP	-6861.218445	-6861.213753	4.8583	30.0386	**-40.9**
wB97X-D	LANL2DZ	-4582.885203	-4582.881617	4.851595151	30.04250168	**-31.2**

6a

Functional	Basis Set	E(Ms=0, BS), hartrees	E(Ms=11), hartrees	<S^2> (Ms=0, BS)	<S^2> (Ms=11)	J (cm^{-1})
B3LYP	def2-SV(P)	-7110.270319	-7110.260887	4.8132	30.0303	**-82.1**
B3LYP	def2-SV(P)/TZVP	-7110.621899	-7110.612562	4.8245	30.0341	**-81.3**
B3LYP	LANL2DZ	-4832.423557	-4832.414458	4.820578183	30.03520059	**-79.2**
wB97X-D	def2-SV(P)	-7108.876424	-7108.868965	4.8675	30.0322	**-65.1**
wB97X-D	def2-SV(P)/TZVP	-7109.226069	-7109.218652	4.8775	30.0356	**-64.7**
wB97X-D	LANL2DZ	-4831.021725	-4831.014437	4.875198884	30.03775533	**-63.6**

3

Functional	Basis Set	E(Ms=0, BS), hartrees	E(Ms=11), hartrees	<S^2> (Ms=0, BS)	<S^2> (Ms=11)	J(cm^{-1})
B3LYP	LANL2DZ	-5545.670955	-5545.670518	4.997604083	30.03257763	**-3.8**
wB97X-D	LANL2DZ	-5544.086083	-5544.086028	5.013565385	30.0364495	**-0.5**

4

Functional	Basis Set	E(Ms=0, BS), hartrees	E(Ms=11), hartrees	<S^2> (Ms=0, BS)	<S^2> (Ms=11)	J(cm^{-1})
B3LYP	LANL2DZ	-5392.747403	-5392.746484	4.985677987	30.02612854	**-8.1**
wB97X-D	LANL2DZ	-5391.217638	-5391.217186	5.001211069	30.02788458	**-4.0**

References

(1) Chilton, N. F.; Anderson, R. P.; Turner, L. D.; Soncini, A.; Murray, K. S. PHI: A powerful new program for the analysis of anisotropic monomeric and exchange-coupled polynuclear d- and f-block complexes. *J. Comput. Chem.* **2013**, *34* (13), 1164-1175.
(2) Hagen, W. R. Wide zero field interaction distributions in the high-spin EPR of metalloproteins. *Mol. Phys.* **2007**, *105* (15-16), 2031-2039.
(3) Luo, Y.-R. *Handbook of Bond Dissociation Energies in Organic Compounds (1st ed.)*; CRC Press, 2002.
(4) *CrysAlisPro Software System, Rigaku Oxford Diffraction*; Rigaku Corporation: Oxford, UK, 2021.
(5) Sheldrick, G. M. SHELXT – Integrated space-group and crystal-structure determination. *Acta Crystallogr., Sec. A: Found. Crystallogr.* **2015**, *71* (1), 3-8.
(6) Sheldrick, G. M. Crystal structure refinement with SHELXL. *Acta Crystallogr., Sec. C: Cryst. Struct. Commun.* **2015**, *71* (Pt 1), 3-8.
(7) O. V. Dolomanov, L. J. B., R. J. Gildea, J. A. K. Howard and H. Puschmann. OLEX2: a complete structure solution, refinement and analysis program. *J. Appl. Crystallogr.* **2009**, *42*, 339-341.
(8) Iovan, D. A.; Betley, T. A. Characterization of Iron-Imido Species Relevant for *N*-Group Transfer Chemistry. *J. Am. Chem. Soc.* **2016**, *138* (6), 1983-1993.
(9) Nichols, P. J.; Fallon, G. D.; Murray, K. S.; West, B. O. Synthesis and magnetic properties of .mu.-organoimido-bridged iron(III) salicylaldimine compounds. Structure of (.mu.-p-tolylimido)bis[(N,N'-ethane-1,2-diylbis(salicylaldiminato))iron(III)] ([Fe(salen)]2N(Tol)). *Inorg. Chem.* **1988**, *27* (16), 2795-2800.
(10) Zart, M. K.; Powell, D.; Borovik, A. S. Synthesis and structures of dimeric iron(III)-Oxo and -imido complexes containing intramolecular hydrogen bonds. *Inorganica Chim. Acta* **2007**, *360* (7), 2397-2402.
(11) Bellow, J. A.; Yousif, M.; Cabelof, A. C.; Lord, R. L.; Groysman, S. Reactivity Modes of an Iron Bis(alkoxide) Complex with Aryl Azides: Catalytic Nitrene Coupling vs Formation of Iron(III) Imido Dimers. *Organometallics* **2015**, *34* (12), 2917-2923.
(12) Bellows, S. M.; Arnet, N. A.; Gurubasavaraj, P. M.; Brennessel, W. W.; Bill, E.; Cundari, T. R.; Holland, P. L. The Mechanism of N–N Double Bond Cleavage by an Iron(II) Hydride Complex. *J. Am. Chem. Soc.* **2016**, *138* (37), 12112-12123.
(13) Hashimoto, T.; Hoshino, R.; Hatanaka, T.; Ohki, Y.; Tatsumi, K. Dinuclear Iron(0) Complexes of *N*-Heterocyclic Carbenes. *Organometallics* **2014**, *33* (4), 921-929.
(14) Takemoto, S.; Ogura, S.-I.; Yo, H.; Hosokoshi, Y.; Kamikawa, K.; Matsuzaka, H. Diiron Amido−Imido Complex $[(Cp^*Fe)_2(\mu_2\text{-NHPh})(\mu_2\text{-NPh})]$: Synthesis and a Net Hydrogen Atom Abstraction Reaction To Form a Bis(imido) Complex. *Inorg. Chem.* **2006**, *45* (13), 4871-4873.
(15) Zhang, Q.; Xiang, L.; Deng, L. Dinuclear Iron–Imido Complexes with *N*-Heterocyclic Carbene Ligation: Synthesis, Structure, and Redox Reactivity. *Organometallics* **2012**, *31* (12), 4537-4543.
(16) Abucayon, E. G.; Chu, J.-M.; Ayala, M.; Khade, R. L.; Zhang, Y.; Richter-Addo, G. B. Insight into the preferential N-binding *versus* O-binding of nitrosoarenes to ferrous and ferric heme centers. *Dalton Trans.* **2021**, *50* (10), 3487-3498.
(17) Dhifaoui, S.; McHiri, C.; Quatremare, P.; Marvaud, V.; Bujacz, A.; Nasri, H. Molecular structure, magnetic properties, cyclic voltammetry of the low-spin iron(III) Bis(4-ethylaniline)

complex with the para-chloro substituted meso-tetraphenylporphyrin. *J. Mol. Struct.* **2018**, *1153*, 353-359.
(18) Dickman, M. H. Bis(1,2-diaminobenzene-N)-bis(1,1,1,5,5,5-hexa-fluoro-pen-tane-2,4-dion-ato-O,O')-iron(II), -cobalt(II) and -nickel(II) at low temperature. *Acta Cryst.* **2000**, *C56*, 58-60.
(19) Eckert, N. A.; Smith, J. M.; Lachicotte, R. J.; Holland, P. L. Low-Coordinate Iron(II) Amido Complexes of β-Diketiminates: Synthesis, Structure, and Reactivity. *Inorg. Chem.* **2004**, *43* (10), 3306-3321.
(20) González Guillén, A.; Oszajca, M.; Łasocha, W. Synthesis, characterization and thermal properties of organic-inorganic hybrid layered materials based on metal sulfates and aromatic diamines: M(diamine)xSO4, (M = Fe, Co; x = 1, 2). *Polyhedron* **2021**, *206*, 115319.
(21) M'thiruaine, C. M.; Friedrich, H. B.; Nyawade, E. A.; Omondi, B. Syntheses and structural characterization of 2,4,6-trimethylaniline complexes of iron carbonyls. *Inorganica Chim. Acta* **2014**, *423*, 550-554.
(22) Wang, J.-J.; Chen, X.; Zhang, B.-S.; Li, C.-B.; Wang, Y.-F. Experimental (X-ray, TGA) and computation (NBO, AIM) studies of Iron(II) complex with thiabendazole and 5-aminoisophthalate. *J. Mol. Struct.* **2021**, *1245*, 131100.
(23) Zdilla, M. J.; Verma, A. K.; Lee, S. C. Reactivity of a Sterically Hindered Fe(II) Thiolate Dimer with Amines and Hydrazines. *Inorg. Chem.* **2008**, *47* (23), 11382-11390.
(24) Stephens, J. C.; Khan, M. A.; Nicholas, K. M. Cyclopentadienyliron complexes of nitrosobenzene: Preparation, structure and reactivity with olefins. *J. Organomet. Chem.* **2005**, *690* (21), 4727-4733.
(25) Tung, C.-Y.; Tseng, Y.-T.; Lu, T.-T.; Liaw, W.-F. Insight into the Electronic Structure of Biomimetic Dinitrosyliron Complexes (DNICs): Toward the Syntheses of Amido-Bridging Dinuclear DNICs. *Inorg. Chem.* **2021**, *60* (21), 15846-15873.
(26) Brown, S. D.; Peters, J. C. Hydrogenolysis of [PhBP3]Fe⁝N-p-tolyl: Probing the Reactivity of an Iron Imide with H2. *J. Am. Chem. Soc.* **2004**, *126* (14), 4538-4539.
(27) Mankad, N. P.; Müller, P.; Peters, J. C. Catalytic N−N Coupling of Aryl Azides To Yield Azoarenes via Trigonal Bipyramid Iron−Nitrene Intermediates. *J. Am. Chem. Soc.* **2010**, *132* (12), 4083-4085.
(28) Smith, J. M.; Lachicotte, R. J.; Holland, P. L. NN Bond Cleavage by a Low-Coordinate Iron(II) Hydride Complex. *J. Am. Chem. Soc.* **2003**, *125* (51), 15752-15753.
(29) Spasyuk, D. M.; Carpenter, S. H.; Kefalidis, C. E.; Piers, W. E.; Neidig, M. L.; Maron, L. Facile hydrogen atom transfer to iron(III) imido radical complexes supported by a dianionic pentadentate ligand. *Chem. Sci.* **2016**, *7* (9), 5939-5944.
(30) Sun, C.; Oswald, V. F.; Hill, E. A.; Ziller, J. W.; Borovik, A. S. Investigation of iron–ammine and amido complexes within a C_3-symmetrical phosphinic amido tripodal ligand. *Dalton Trans.* **2021**, *50* (32), 11197-11205.
(31) Wang, X.; Mo, Z.; Xiao, J.; Deng, L. Monomeric Bis(anilido)iron(II) Complexes with N-Heterocyclic Carbene Ligation: Synthesis, Characterization, and Redox Reactivity toward Aryl Halides. *Inorg. Chem.* **2013**, *52* (1), 59-65.
